# Surface Immunoproteomics Reveals Potential Biomarkers in *Alicyclobacillus acidoterrestris*

**DOI:** 10.3389/fmicb.2018.03032

**Published:** 2018-12-04

**Authors:** Yiheng Shi, Tianli Yue, Yipei Zhang, Jianping Wei, Yahong Yuan

**Affiliations:** ^1^College of Food Science and Engineering, Northwest A&F University, Yangling, China; ^2^Laboratory of Quality and Safety Risk Assessment for Agro-Products, Ministry of Agriculture, Yangling, China; ^3^National Engineering Research Center of Agriculture Integration Test, Yangling, China

**Keywords:** immunoproteomics, immunogenic proteins, *Alicyclobacillus acidoterrestris*, cell surface proteins, biomarkers

## Abstract

*Alicyclobacillus acidoterrestris* is a major putrefying bacterium that can cause pecuniary losses in the global juice industry. Current detection approaches are time-consuming and exhibit reduced specificity and sensitivity. In this study, an immunoproteomic approach was utilized to identify specific biomarkers from *A. acidoterrestris* for the development of new detection methods. Cell surface-associated proteins were extracted and separated by 2-D (two-dimensional) gel electrophoresis. Immunogenic proteins were detected by Western blot analysis using antisera against *A. acidoterrestris*. Twenty-two protein spots exhibiting immunogenicity were excised and eighteen of the associated spots were successfully identified by matrix-assisted laser desorption/ionization time-of-flight tandem mass spectrometry (MALDI-TOF/TOF MS). These proteins were observed to be involved in energy and carbohydrate metabolism, transmembrane transport, response to oxidative stress, polypeptide biosynthesis, and molecule binding activity. This is the first report detailing the identification of cell surface-associated antigens of *A. acidoterrestris*. The identified immunogenic proteins could serve as potential targets for the development of novel detection methods.

## Introduction

*Alicyclobacillus acidoterrestris* is a thermo-acidophilic, aerobic, spore-forming, Gram-positive bacterium which is capable of growing at a temperature range of 25–60°C, and a pH range of 2.5–6.0 (Chang et al., 2013). The spores that are produced by *A. acidoterrestris* can germinate and grow at pH < 4 and are capable of resisting heat treatment at 90°C for 16 to 23 min, a more stringent heat treatment regimen than the conventional pasteurization treatments used in juice processing (Steyn et al., 2011; Celenk and Ayse Handan, 2015). *A. acidoterrestris* can use vanillin and tyrosine as precursors in the synthesis of guaiacol, an organic compound that emits a “phenolic” odor (Uchida and Silva, 2017). The latter compound triggers spoilage of fruit juices and acidic beverages, resulting in significant economic losses to the juice industry (Oteiza et al., 2015; Fernandez et al., 2017). According to a survey conducted by the European Fruit Juice Association (AIJN) in 2005, about 45% of the 68 fruit processing industries experienced *Alicyclobacillus* related problems, including 33% undergoing problems more than once (Steyn et al., 2011). Current detection methods are either labor-intensive and time-consuming or highly technique-requiring (Chang and Kang, 2004; Concina et al., 2010; Pérez-Cacho et al., 2011; Wang et al., 2012). Although immunoassays have been developed for many years, the effectiveness of immunoassays largely depends on the quality of antibody. Bacteria with high homology could express similar antigens especially in gram-positive bacteria that teichoic-acid could serve as an important surface antigen in all species (Pasquina et al., 2013). So, finding species-specific biomarkers and preparing their corresponding antibodies could lead to the development of more accurate methods for the detection of *A. acidoterrestris*.

As a combination of immunology and proteomics, immunoproteomics has been successfully utilized in pathogenic microorganism for revealing the pathogenesis and elucidating novel diagnostic biomarkers (Hernandez-Haro et al., 2015; Ray et al., 2016; Chen et al., 2018). Thus, immunoproteomics can be used as a preliminary step for specific antibody and immunoassay development to facilitate the detection of foodborne microbiology accurately and rapidly possible (Xiong et al., 2012; Rasmussen et al., 2016). However, up until now limited research has been performed to investigate the use of this technique in detecting non-pathogenic bacteria capable of contaminating food matrices and products.

For most bacteria, the cell wall proteins are often exposed at the surface and exhibit strain specificity (Yu et al., 2012). Thus, based on these properties, cell surface exposed proteins have already served as drug targets and for vaccine and pathogen-specific immunoassay techniques development (Rodriguez-Ortega et al., 2006). The aim of this study was to resolve the cell wall proteins of *A. acidoterrestris* using an immunoproteomics approach to find species-specific biomarkers for immunodetection of this bacterium. Type strain *A. acidoterrestris* DSM3923 was used in this research. Cell wall proteins were extracted and separated by 2-D gel electrophoresis. Protein spots on gels exhibiting immunogenicity were identified and these proteins were chosen as biomarkers for future immunoassay development. We expect that we can monitor the *A. acidoterrestris* in real time from orchard to table in the future based on our findings. Therefore, it could reduce or prevent economic losses to the juice industry resulting from spoilage caused by metabolic products of *A. acidoterrestris.*

## Materials and Methods

### Ethics Statement

This study was carried out in accordance with the recommendations of the Animal Ethics Procedures and Guidelines of the People’s Republic of China. All efforts were exerted to minimize the suffering of animals. The animal experiments were approved by the Ethics Committee of Northwest A&F University.

### Strain and Growth Conditions

*Alicyclobacillus acidoterrestris* DSM 3923 used in this study was purchased from the Deutsche Sammlung von Mikroorganismen und Zellkulturen (DSMZ) and stored at -80°C. The culture was grown in AAM broth (Yamazaki et al., 1996) with some modifications (yeast extract 2.0 g, glucose 2.0 g, (NH_4_)_2_SO_4_ 0.4 g, MgSO_4_7H_2_O 1.0 g, KH_2_PO_4_ 1.2 g, CaCl_2_ 0.38 g, distilled water 1000 mL, pH 4.0) on a shaker at 45°C.

### Preparation of Immunized Sera

Antisera against whole *A. acidoterrestris* DSM 3923 cells were obtained by immunizing rabbits as previously described (Wang et al., 2012). Briefly, two New Zealand white male rabbits (Xi’an Jiaotong University Health Science Center, China) were subcutaneously immunized with formaldehyde-inactivated *A. acidoterrestris* DSM 3923 at a dose of 1 × 10^8^ CFU/rabbit mixed with an equal volume of Freund’s complete adjuvant (Sigma, United States). Four booster immunizations were administered every 2 weeks using the same concentration of bacterial cells mixed with Freund’s incomplete adjuvant (Sigma, United States). One week after the final booster immunization, the rabbits were anesthetized with absolute ether and sacrificed to collect blood samples. The blood samples were incubated at 37°C for 1–2 h followed by overnight incubation at 4°C. The samples were subsequently centrifuged at 4500 *g* for 15–20 min at 4°C and the sera were collected and divided into 1 mL-aliquots and stored at -20°C until further required. Next, the isolated antisera were purified by MabSelect SuRe (GE Healthcare, United States) and stored at -20°C until further required.

### Preparation of Cell Wall Proteins

Cell wall proteins from *A. acidoterrestris* DSM 3923 were isolated according to a method (with some modifications) published by Siegel et al. (1981). Briefly, *A. acidoterrestris* cells grown to the log phase (OD600 = 0.5) were harvested by centrifugation at 10,000 *g* for 10 min at 4°C. The cells were subsequently washed three times with PBS (137 mM NaCl, 2.7 mM KCl, 10 mM Na_2_HPO_4_, 2 mM KH_2_PO_4_) to remove the excess culture medium. The resultant pellets were suspended in extraction buffer (30 mM Tris–HCl (pH 7.5), 3 mM MgCl_2_, 25% sucrose, 125 U/mL mutanolysin, 400 U/mL lysozyme) containing a protease inhibitor cocktail (Roche, Switzerland) and incubated at 37°C for 90 min. The protoplast fraction was removed by centrifugation at 10,000 *g* for 10 min at 4°C and the supernatant containing the cell wall proteins was collected and filtered through a 0.22 μm R.C membrane. The cell wall proteins were precipitated using trichloroacetic acid (TCA)/acetone (Wu et al., 2014). Prechilled (-20°C) 10% TCA/acetone containing 0.1% Dithiothreitol (DTT) was added to the resultant supernatant. Proteins were allowed to precipitate for 1 h at -20°C. The precipitate was washed three times with cold (-20°C) acetone containing 0.1% DTT to remove TCA. The final pellet was air-dried and solubilized in rehydration buffer [7 M urea, 2 M thiourea, 4% (w/v) CHAPS, 65 mM DTT and 0.2% (v/v) Bio-Lyte pH range 3–10 (Bio-Rad, United States)]. Protein concentration was determined by the Bradford assay.

### 2-D Gel Electrophoresis

For the first dimension, isoelectric focusing (IEF) was performed in a PROTEAN^TM^ IEF cell (Bio-Rad, United States). Cell wall protein samples (approximately 400 μg each) were suspended in rehydration buffer containing 0.001% (w/v) bromophenol blue and loaded onto the IEF focusing tray with ReadyStrip^TM^ IPG Strips (17 cm, pH 4–7). The samples were subsequently rehydrated overnight (12 h) at 20°C, and 50 V. IEF was carried out using the following conditions: 250 V for 30 min, 1000 V for 1 h, 8500 V for 5 h and final focusing at 8500 V for a total of 60,000 Vh. The current was limited to 50 μA per IPG strip, and the temperature was kept at 20°C for all of the focusing steps. After the IEF, the strip was equilibrated for 15 min with 5 mL of equilibration buffer (6 M urea, 2% (w/v) SDS, 0.375 M Tris-HCl (pH 8.8), 20% (v/v) glycerol) containing 2% (w/v) DTT and another 15 min in the same buffer containing 2.5% iodoacetamide. Finally, the IPG strip was dipped into running buffer (25 mM Tris base, 192 mM glycine, 0.1% (w/v) SDS, pH 8.3), positioned on top of a 12% polyacrylamide gel and sealed in place with 0.5% agarose. The second-dimension electrophoresis was carried out in PROTEAN II XL Cell (Bio-Rad, United States). The resultant gel was run at 10 mA for 30 min and 25 mA until the bromophenol dye reached the bottom of the gel. Following electrophoresis, the gel was stained using Coomassie Brilliant Blue G-250 and scanned using a GS-800^TM^ Calibrated Densitometer (Bio-Rad, United States). Three independent replicates were performed for each experiment.

### Immunoblot Analysis

Proteins in the gel were transferred onto a 0.45 μm Immobilon-P polyvinylidene difluoride (PVDF) membrane (Merck Millipore, United States) using a Trans-Blot^®^SD Semi-Dry Electrophoretic Transfer Cell (Bio-Rad, United States) at 25 V for 30 min with transfer buffer (25 mM Tris base, 192 mM glycine, 0.02% (w/v) SDS, 20% (v/v) methanol). Next, the membrane was rinsed three times with TBST (20 mM Tris base, 137 mM NaCl, 0.1% Tween-20, pH 7.5) for 5 min and blocked with 5% skimmed milk in TBST for 2 h at room temperature. The membrane was subsequently incubated with 1:2000 diluted antisera at 4°C overnight. Next, the membrane was rinsed five times with TBST for 8 min and incubated with a 1:10000 dilution of goat anti-rabbit IgG horseradish peroxidase (HRP)-conjugated secondary antibody (Jackson, United States) for 1 h at room temperature. Finally, the membrane was washed five times with TBST for 8 min and the excess buffer was dried with filter paper. Immunoreactive spots on the membrane were detected by chemiluminescence (Pierce^TM^ ECL Western Blotting Substrate, Thermo Scientific, United States) according to the manufacturer’s instructions. The blot was visualized using a ChemiDo^TM^ XRS+ system (Bio-Rad, United States).

### In-Gel Digestion

In-gel digestion was performed as previously described (Shevchenko et al., 1996) with some modifications. Briefly, immunoreactive protein spots were selected and manually excised from stained gels. The gel spots were destained with 30% acetonitrile (ACN) containing 100 mM NH_4_HCO_3_ and dehydrated in 100% ACN. The liquid phase was removed. The gel pieces were completely dried in a vacuum centrifuge. Next, the gel pieces were reswollen with 10 ng/μL sequencing-grade trypsin at 4°C for 30 min; the pieces were subsequently incubated at 37°C overnight (approximately 20 h). Excess trypsin solution was removed and peptides were extracted three times with 60% ACN containing 0.1% trifluoroacetic acid (TFA). Then the extracts were pooled and lyophilized.

### MALDI-TOF/TOF MS Analysis

The lyophilized peptides were dissolved in 20% acetonitrile. The samples (1 μL each) were spotted on a MALDI target plate and allowed to air-dry. Next, 0.5 μL of matrix (5 mg/mL α-cyano-4-hydroxycinnamic acid diluted in 0.1% TFA and 50% ACN) were mixed with dried peptides and allowed to air-dry. Sample analysis was carried out using a 4800 Plus MALDI-TOF/TOF MS Analyzer (Applied Biosystems, United States) in positive ion reflector mode with 2 kV accelerating voltage and 355 nm Nd:YAG laser source. Positive ion mode and automatic acquisition mode were used for data collection. A peptide mass fingerprint (PMF) in the 800–4000 Da range was generated. Peaks with a signal-to-noise ratio greater than 50 were selected for MS/MS analysis using a collision energy of 2 kV.

### Bioinformatics Analysis

Data searches were performed using MASCOT 2.2 (Matrix Science, United Kingdom) and Global Proteome Server Explorer (Applied Biosystems) protein identification. The parameters were set as follows: UniProt database, and species restriction to *A. acidoterrestris*; Enzyme, Trypsin; Fixed modifications, Carbamidomethyl (C)s; Dynamical modifications, Oxidation (M); Peptide Mass Tolerance, ± 100 ppm; Fragment Mass Tolerance, ± 0.4 Da; Peptide Charge State, 1+; Max Missed Cleavages, 1. GPS software-reported protein scores with confidence intervals (C.I.%) greater than 95% were considered as successful identifications for proteins. Protein–protein interaction was performed using STRING [version 10.5^1^ (Szklarczyk et al., 2017)] and the protein network was constructed.

### SDS–PAGE and Western Blot Analysis of Identified Proteins

Eight identified antigens, namely, GADPH, Polyamine aminopropyl transferase, Succinate-CoA ligase [ADP-forming] subunit alpha, EF-Tu, 6PGD, Hypothetical protein N007_04435, SOD, AhpC were chosen for antigenicity veridiction. Genomic DNA of DSM 3923 were extracted by QIAamp DNA Mini Kit (Qiagen, Germany) following manufacturer’s instruction. PCR reactions were performed using T100^TM^ Thermal Cycler (Bio-Rad, United States). The PCR products were purified by Gel Extraction Kit (Omega BIO-TEK, United States) before cloned into pET-28a (+) and pET-32a (+) vectors (Novagen, Germany). The constructs were sequenced by ABI 3730XL. Recombinant plasmids were transformed into *E. coli* BL21 (DE3) (Invitrogen^TM^, United States) competent cells for expression. The recombinant proteins were purified by Ni-NTA His Bind Resin (Merck Millipore, United States) and examined by 12% SDS–PAGE. Western blot was performed as described above, apart from the transfer parameters which were changed from 25 V for 30 min into 15 V for 15 min.

## Results

### 2-D Gel Electrophoresis Map of Cell Wall Proteins of *A. acidoterrestris* DSM 3923

**FIGURE 1:**
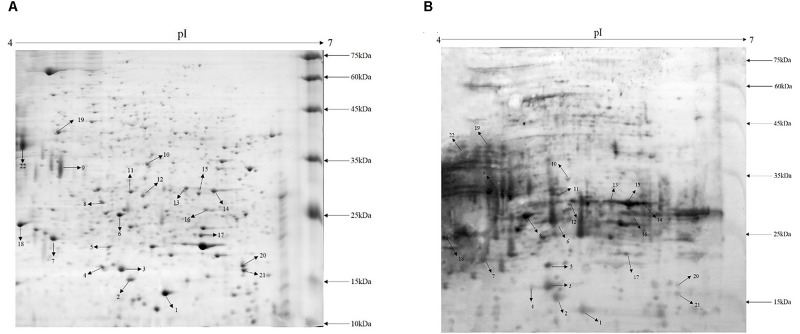
Proteome map and immunoblot profile of cell wall proteins of *A. acidoterrestris.* **(A)** 2-D map of cell wall proteins; **(B)** immunoblot analysis of cell wall proteins.

Cell wall proteins of *A. acidoterrestris* were extracted using enzymolysis and separated by 2-D gel electrophoresis. A total of more than 200 spots in the gel were clearly observed with a molecular mass range from 10 to 75 kDa and pH range from 4 to 7 as shown in Figure 1A. Protein spots were evenly scattered and most of them showed in small light dots. High abundance proteins (big spots) were predominantly distributed in the 10–35 kDa range and low abundance proteins (small spots) in the 35–75 kDa range.

### Immunoblot Analysis of Cell Wall Proteins of *A. acidoterrestris* DSM 3923

Cell wall extracts of *A. acidoterrestris* DSM 3923 separated by 2-D gel electrophoresis were blotted onto a PVDF membrane and visualized using chemiluminescence. Figure 1B shows that a large number of protein spots reacted with the antisera and the strongest reactors were mainly in the 15–35 kDa range. This analysis also indicated that, compared with the 2-D map, highly abundant proteins reacted strongly with antisera while those proteins that were less prevalent reacted with the antisera in a less intense fashion. Furthermore, several intense reaction spots that were not observed clearly on the gel were detected by Western blot, indicating that some proteins that were expressed at reduced levels but were exposed on the cell surface could potently stimulate host immune response (Wang et al., 2013).

### Identification of Cell Wall Protein Immunogens in *A. acidoterrestris* DSM 3923

**FIGURE 2:**
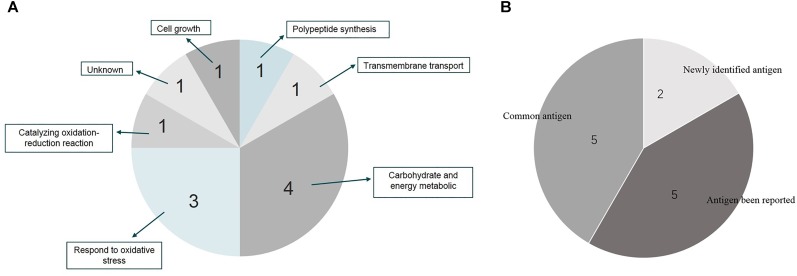
Distribution and function summary of identified antigens from *A. acidoterrestris* cell wall. **(A)** Function characterization of identified antigens; **(B)** antigen description of identified proteins.

**FIGURE 3:**
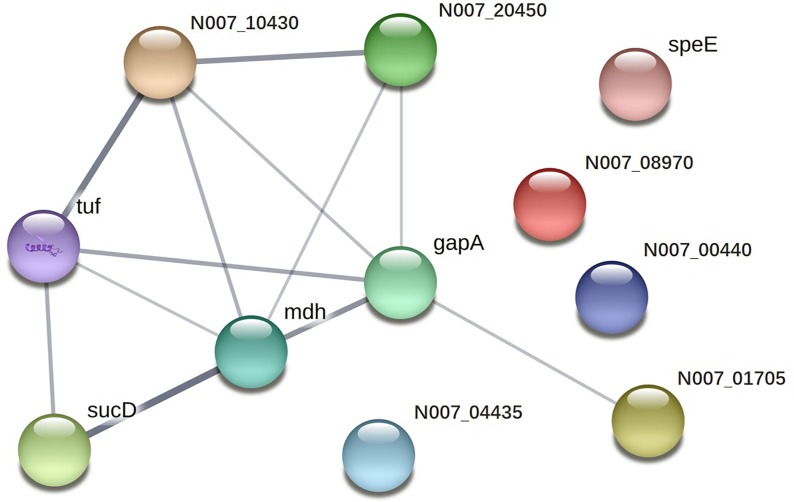
Protein–protein interaction network of immunoreactive proteins.

Twenty-two immunoreactive spots recognized by antisera that correlated with relatively high abundance protein spots in the Coomassie-stained gel were picked for MALDI-TOF/TOF MS identification. All twenty-two immunoreactive spots were excised from the gel and eighteen spots were successfully identified (Table 1). Of the eighteen spots, eight were characterized as three kinds of hypothetical protein (N007_00440, N007_08970, N007_04435). Two spots were identified as the elongation factor Tu (EF-Tu), and the rest were classified as metabolic enzymes (Table 2). These proteins were mainly involved in transmembrane transport of matter, carbohydrate and energy metabolic process, chain elongation in polypeptide synthesis, maintaining redox homeostasis in respond to oxidative stress, and cell growth (Figure 2A and Table 2). Among the identified antigens, two were newly found in our study that never been reported in the previous researches. Five were identified as “common antigen” and the rest five were classified into “antigen been reported” (Figure 2B and Table 2). To further understand the property and correlation of immunogenic proteins, Protein–protein interaction prediction was performed using online database STRING. Figure 3 shows that seven proteins which involved in glycometabolism and oxidative stress could interact with each other. The three hypothetical proteins include two newly identified antigens showed no relationship with others.

**Table 1:** Immunoreactive proteins in *A. acidoterrestris* cell wall identified by MALDI-TOF MS.

Spot no.	Protein name	Accession no.	Gene	Theoretical Mr (kDa)/pI	Protein score	Matched pep.	Coverage %
1	Hypothetical protein N007_00440	T0C5G8	N007_00440	63753/4.64	209	7	19.44
2	Probable thiol peroxidase	T0CJI3	tpx	18634.5/5.23	317	17	87.21
4	Elongation factor Tu	T0D867	tuf	43353/5.09	409	13	38.48
5	Alkyl hydroperoxide reductase subunit C	T0CIG8	N007_20450	20796.5/4.93	367	11	64.71
6	Elongation factor Tu	T0D867	tuf	43353/5.09	596	15	42.78
9	Hypothetical protein N007_08970	T0BYX4	N007_08970	114313.8/4.01	188	8	7.05
10	Malate dehydrogenase	T0BTR2	mdh	33360.7/5.2	381	17	72.12
11	Hypothetical protein N007_04435	T0C6H1	N007_04435	32737.6/5.18	499	14	56.56
12	Succinate-CoA ligase [ADP-forming] subunit alpha	T0C0Z3	sucD	31636.3/5.37	469	15	50.66
13	Polyamine aminopropyl transferase	T0DMQ2	speE	31094.8/5.52	308	16	65.7
14	Hypothetical protein N007_08970	T0BYX4	N007_08970	114313.8/4.01	183	6	4.87
16	Hypothetical protein N007_08970	T0BYX4	N007_08970	114313.8/4.01	129	6	4.7
17	Glyceraldehyde-3-phosphate dehydrogenase	T0BM94	gapA	35675.5/5.78	103	11	35.33
18	Hypothetical protein N007_00440	T0C5G8	N007_00440	63753/4.64	174	9	49.2
19	Hypothetical protein N007_00440	T0C5G8	N007_00440	63753/4.64	142	9	67.38
20	Superoxide dismutase	T0D4I3	N007_10430	22299.1/5.66	280	11	65.35
21	6-Phosphogluconate dehydrogenase	T0CYB3	N007_01705	32612.2/5.57	384	12	41.95
22	Hypothetical protein N007_08970	T0BYX4	N007_08970	114313.8/4.01	117	7	5.48


**Table 2:** Summary of immunoreactive protein spots from *A. acidoterrestris* cell wall extracts.

Spot no.	Characterization	Potential function	Description
1, 18, 19	Hypothetical protein N007_00440	Transmembrane transport	Antigen been reported
9, 14, 16, 22	Hypothetical protein N007_08970	Unknown	Newly identified antigen
4, 6	Elongation factor Tu	Polypeptide synthesis	Common antigen
2	Probable thiol peroxidase	Respond to oxidative stress	Antigen been reported
5	Alkyl hydroperoxide reductase subunit C	Respond to oxidative stress	Antigen been reported
10	Malate dehydrogenase	Carbohydrate and energy metabolic	Common antigen
11	Hypothetical protein N007_04435	Catalyzing oxidation-reduction reaction	Newly identified antigen
12	Succinate-CoA ligase [ADP-forming] subunit alpha	Carbohydrate and energy metabolic	Antigen been reported
13	Polyamine aminopropyl transferase	Cell growth	Antigen been reported
17	Glyceraldehyde-3-phosphate dehydrogenase	Carbohydrate and energy metabolic	Common antigen
20	Superoxide dismutase	Respond to oxidative stress	Common antigen
21	6-Phosphogluconate dehydrogenase	Carbohydrate and energy metabolic	Common antigen


### Validation of Antigenic Property of Recombinant Cell Wall Proteins

**FIGURE 4:**
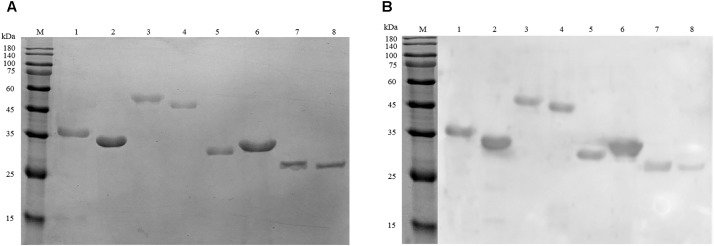
Analysis of recombinant antigen proteins. **(A)** SDS–PAGE of eight purified recombinant proteins. **(B)** Immunoreactivity analysis of recombinant proteins using anti-*A. acidoterrestris* polyclonal antibody. Lanes: M, pre-stain protein molecular weight marker (kDa). 1–8: GADPH, polyamine aminopropyl transferase, succinate-CoA ligase [ADP-forming] subunit alpha, EF-Tu, 6PGD, hypothetical protein N007_04435, SOD, AhpC.

To further evaluate the potential as biomarkers of the identified proteins, eight proteins were cloned and expressed in *E. coli* BL21 (DE3) cell. Figure 4A confirms that the recombinant proteins were successfully expressed. The molecule mass of recombinant proteins matched the data of MS analysis. Western blot demonstrates that the eight proteins could be recognized by anti-*A. acidoterrestris* antisera (Figure 4B).

## Discussion

*Alicyclobacillus acidoterrestris* is a thermo-acidophilic bacterial species that is capable of surviving conventional pasteurization procedures for fruit juices and acidic products. The presence of this bacterium results in product spoilage and economic losses (dos Anjos et al., 2016). However, existing detection methods used to identify this microorganism are not without problems (Chang et al., 2013). It has previously been suggested that bacterial cell wall-associated proteins could serve as potential diagnostic candidates for detection. However, the surface proteome of *A. acidoterrestris* cells has yet to be elucidated. Hence, in the present study, we utilized 2-D gel electrophoresis followed by Western blotting to elucidate new biomarkers for *A. acidoterrestris*.

The use of two kinds of cell wall hydrolases, lysozyme and mutanolysin, in the extraction of cell wall proteins, has facilitated the release proteins attached to the cell wall, thereby giving a more complete picture of the proteome. Figure 1A shows most of protein spots are small and faint, while only a few are large and of high intensity. In gram-positive bacteria, cell walls mainly contain peptidoglycan and teichoic acid,with less than 10% of the exported proteins covalently attached to the bacterial cell wall (Siegel et al., 2016). Thus, the cell wall proteome indicates that most proteins are expressed at low levels. However, a large number of spots could elicit strong reactions against the antisera including many spots which were not clearly observed on the gel. Overall, the data show that the cell wall proteins of *A. acidoterrestris* were successfully extracted and had a high affinity to the antisera. While these proteins were present at low levels, they were sufficiently exposed on the surface to elicit a strong antibody response (from the rabbits) (Wang et al., 2013).

Among the eighteen spots that were successfully identified, three proteins (Hypothetical protein N007_00440, Hypothetical protein N007_08970, Elongation factor Tu) were identified from two or more spots. The presence of the same protein in multiple spots was also observed in the proteomic study of *Sporothrix schenckii* (Messias et al., 2015). The existence of natural isoforms, post-translational modifications, or differential methods of sample preparation might explain these divergences (Wang et al., 2013).

Five of the identified proteins were recognized as “common antigen,” as these proteins have been widely reported in pathogenic bacteria. Typically, 6-phosphogluconate dehydrogenase (6PGD) (spot 21), glyceraldehyde-3-phosphate dehydrogenase (GAPDH) (spot 17) and malate dehydrogenase (MDH) (spot 10) which play a key role in the carbohydrate and energy metabolic pathway so that they can catalyze different substrates to maintain normal function of bacteria (Rendina et al., 1984; Sirover, 1997; Musrati et al., 1998). Consequently, these proteins are the most active in the cellular metabolism and have been identified as immunogenic proteins in many pathogenic bacteria (Severin et al., 2007; Wang et al., 2013; Elfaham et al., 2017). Superoxide dismutase (SOD) (spot 20) were also recognized by the antisera. It can convert superoxide (O_2_^-^) radicals into either ordinary molecular oxygen (O_2_) or hydrogen peroxide (H_2_O_2_) to protect cells from oxidative stress and contributes to the survival of some pathogenic bacteria *in vivo* (Zhang et al., 1991). Thus, it has served as important biomarker in *Cronobacter sakazakii* (Wang et al., 2013). Notably, Elongation factor Tu (EF-Tu) (spot 4, 6) were also identified in our study. This protein is mainly involved in chain elongation during polypeptide synthesis which could bind and transport of aminoacyl-tRNA to the ribosome and interact with GTP in the catalysis of GTP into GDP (Caldas et al., 1998). It is widely distributed in bacteria and has been identified as immunogen in many microorganisms (Fowsantear et al., 2014; Casas et al., 2017).

Five proteins were classified as “antigen been reported,” which means that these proteins have been identified as antigen candidates and served as biomarkers in diagnosis of pathogens in other studies (Stent et al., 2012; Kim et al., 2014; Gomes et al., 2016). These proteins also participate in the metabolic pathway to regulate the function of bacteria. Thiol peroxidase and alkyl hydroperoxide reductase subunit C (AhpC) (spot 5) reduce hydrogen peroxide and peroxynitrite to maintain homeostasis of bacteria and protect cell from oxidative damage (Delaunay et al., 2002; Zuo et al., 2014). They have been identified as important immunogens and vaccine candidates of *Helicobacter pylori* (Stent et al., 2012) and anthrax (Kim et al., 2014), respectively. Succinate-CoA ligase [ADP-forming] (spot 12), also named Succinyl-CoA synthetase, functions in substrate-level phosphorylation in the citric acid cycle (TCA), catalyzing succinate, CoA and ATP or GTP into Succinyl-CoA, phosphate and ADP or GDP, respectively. It has been suggested that this enzyme is a potential antigenic target that could be utilized for the development of a novel diagnostic approach for pathogenic bacterium detection *Bartonella bacilliformis* (Gomes et al., 2016). Moreover, an ATP-binding cassette (ABC) transporter substrate-binding protein analog, hypothetical protein N007_00440 (spot 1, 18, and 19) was recognized as an immunoreactive protein. This protein is mainly involved in transmembrane transport process and is capable of binding substrates outside of the cell and delivering them into cell (Schneider and Hunke, 1998). Members of this family are also involved in the antigen presentation and bacterial pathogenesis and were able to be used as targets to facilitate the detection of pathogenic bacteria (Rodriguez-Ortega et al., 2006; Chen et al., 2018). Polyamine aminopropyl transferase (spot 13) is involved in the first step of the sub-pathway of polyamine biosynthesis which is mainly function in the growth and function of a normal cell (Wallace et al., 2003; Belda-Palazon et al., 2012).

Two proteins were newly identified as immunogens. Protein spots 9, 14, 16, and 22 were identified as the same protein, hypothetical protein N007_08970. This protein was first identified and reported in our research. The structural property and functional information have not been clearly elucidated in any research up to now. Furthermore, homologs from other organisms eliciting immunoreactions have yet to be reported. Hypothetical protein N007_04435 (spot 11) was also first identified as a novel antigen in our study and predicted to be similar to aldo–keto reductases which are involved in the catalysis of a diverse range of substrates, including aliphatic and aromatic aldehydes, monosaccharides, steroids, prostaglandins, polycyclic aromatic hydrocarbons, and isoflavonoids (Hyndman et al., 2003). As for hypothetical protein N007_04435 (spot 11), homologs from other organisms eliciting similar immunoreactions have yet to be elucidated. However, Aldo–keto reductases have been described as therapeutic drug targets (Jez et al., 1997). Our present work demonstrates that this protein is easily recognizable by antibodies. The results support that these proteins are excellent candidates as biomarkers for the detection of *A. acidoterrestris.* The fact that no interaction partners were identified for either N007_08970 or N007_04435 (Figure 3), illustrates their novelty as antigens from a different perspective.

## Conclusion

This is the first study to report the occurrence of specific antigens in the cell surface of *A. acidoterrestris*, a species that causes fruit juice contamination leading to significant economic losses. Hypothetical protein N007_08970 and Hypothetical protein N007_04435 are two antigens that were first identified as part of this study. The other antigens that were identified as part of this study are homologs of antigens reported in pathogenic species and could also act as biomarkers. We believe that the species-specific antigens identified in this study may be important in the development of methods for the accurate detection of *A. acidoterrestris* in fruit juices.

GADPH, polyamine aminopropyl transferase, succinate-CoA ligase [ADP-forming] subunit alpha, EF-Tu, 6PGD, hypothetical protein N007_04435, SOD and AhpC were selected to express as recombinant in *E. coli* to further evaluate the potential as molecular biomarkers for detection or antibody development. All selected proteins could react with antisera against *A. acidoterrestris*. This demonstrates that these proteins are suitable targets for future development of an immunological detection method for *A. acidoterrestris*.

## Author Contributions

YS conceived and designed the experiments, analyzed the data, and wrote the manuscript. YS and YZ conducted the experiments. YY and TY supervised all work. YS, JW, YY, and TY revised the manuscript.

## Conflict of Interest Statement

The authors declare that the research was conducted in the absence of any commercial or financial relationships that could be construed as a potential conflict of interest.

## Footnotes

1 https://string-db.org/
