# Supplementary material for: Surface Immunoproteomics Reveals Potential Biomarkers in Alicyclobacillus acidoterrestris
Source: Front Microbiol. 2018 Dec 4;9:3032. doi: 10.3389/fmicb.2018.03032 (PMC6288362; doi:10.3389/fmicb.2018.03032)
Supplement: Supplementary file 1 [file Data_Sheet_1.PDF]

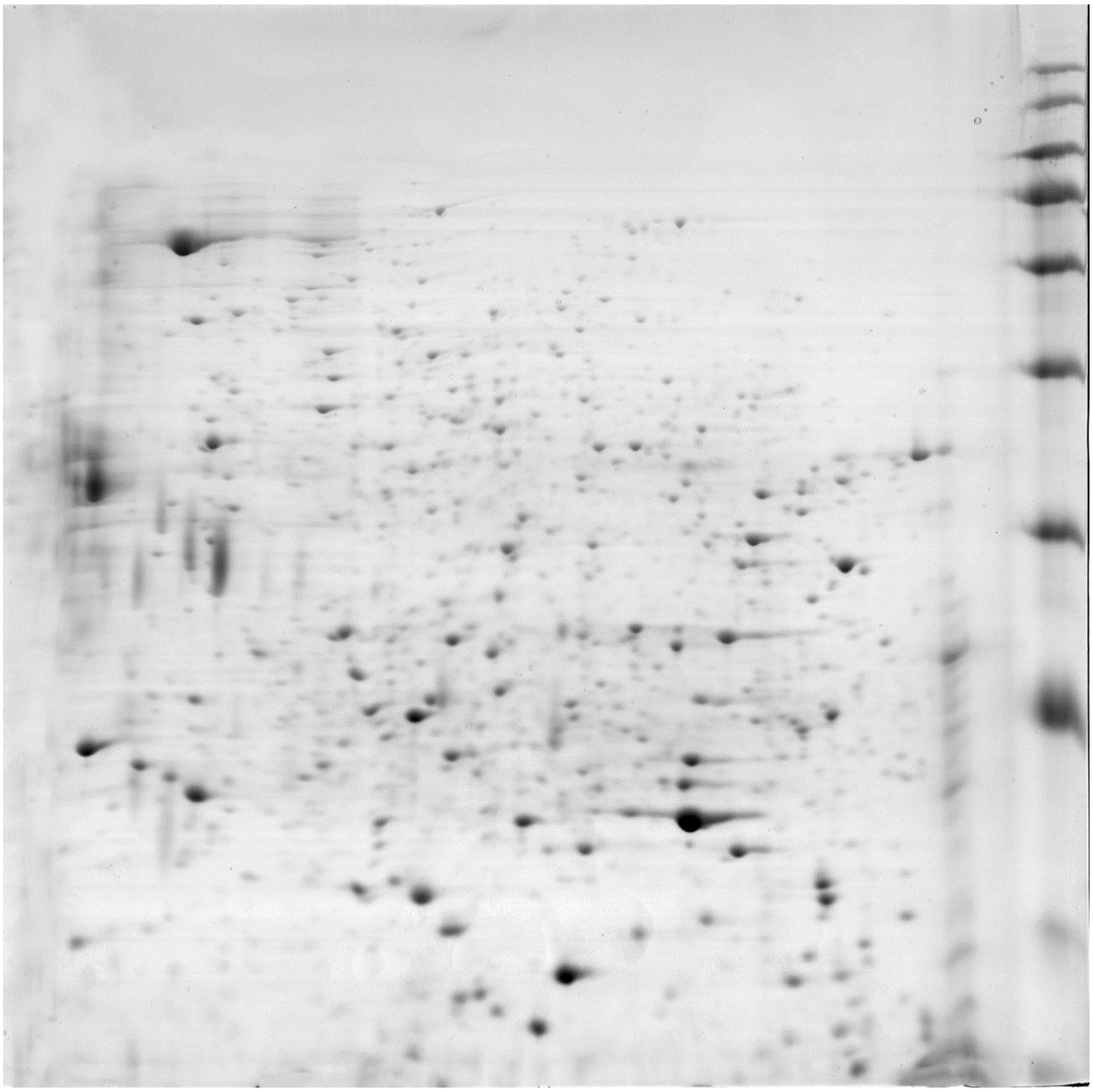

2-D original figure

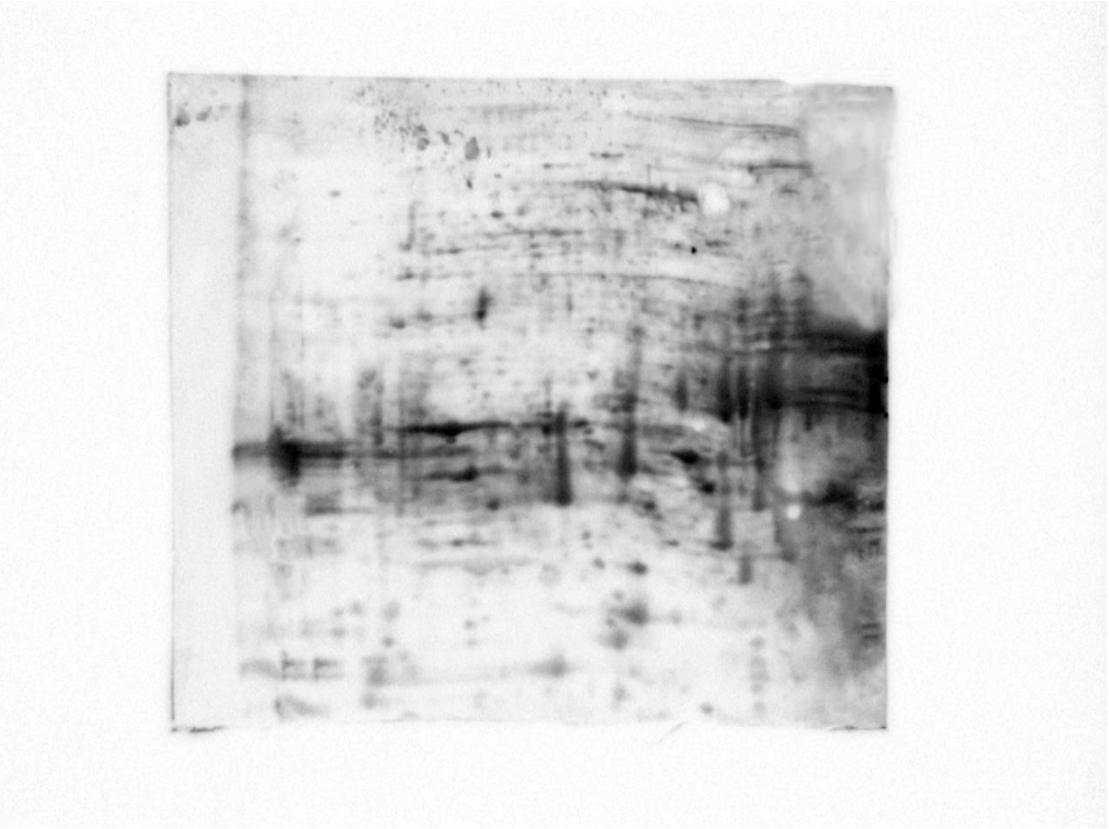
Immunoblot original figure

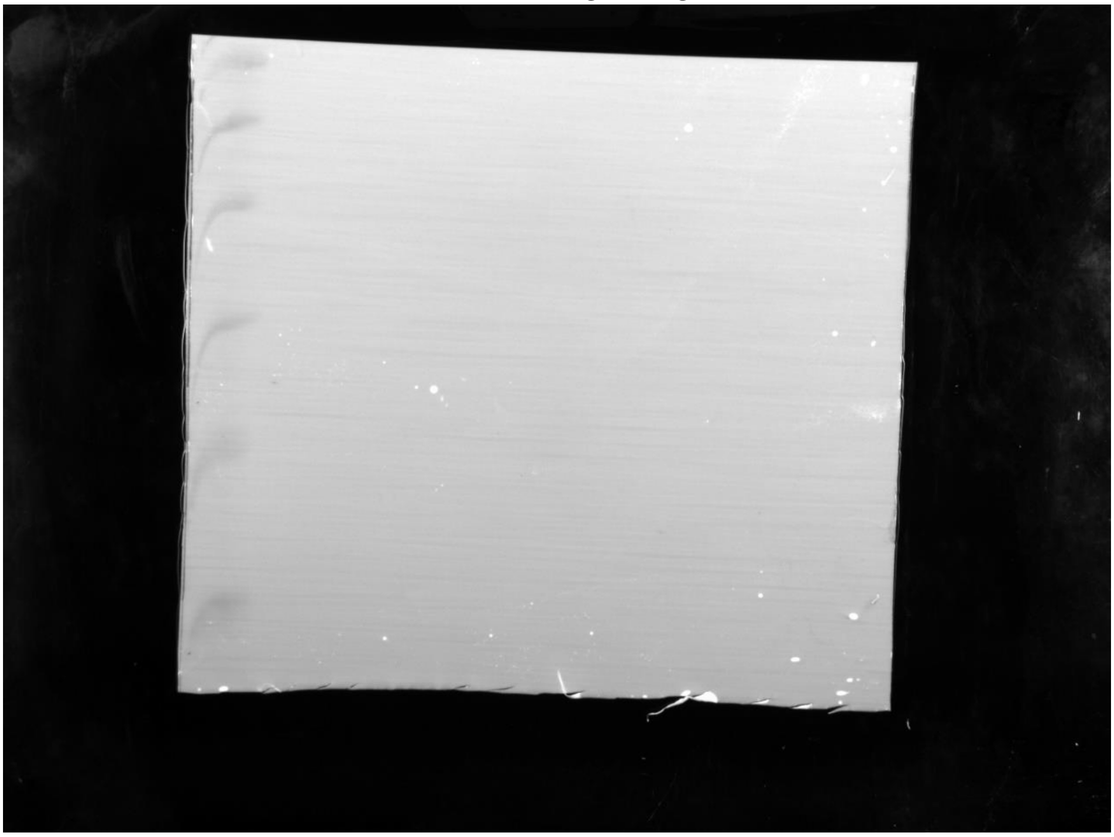
Marker

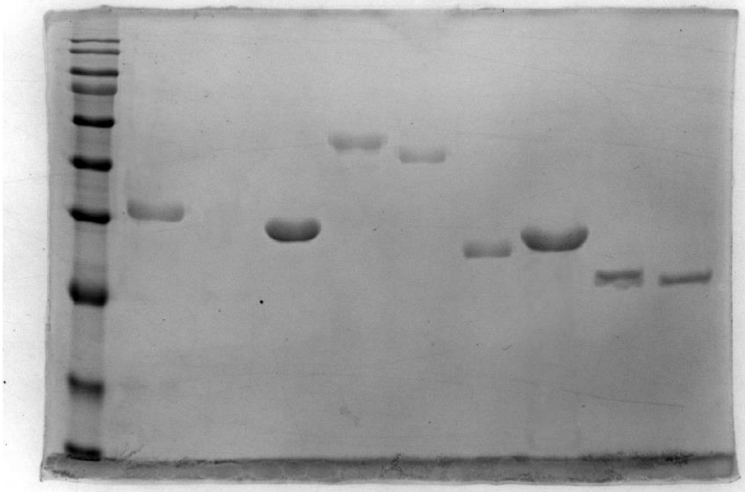

SDS-PAGE of purified recombinant proteins original figure

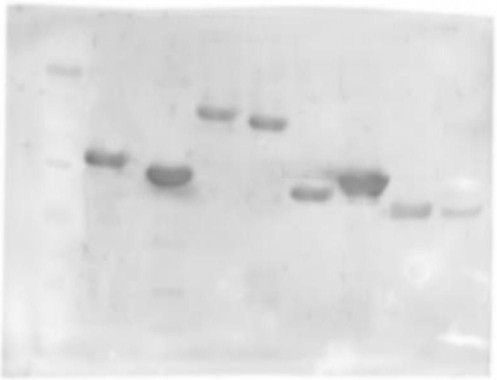

Western blot of purified recombinant proteins original figure
