# Supplementary material for: Surface Immunoproteomics Reveals Potential Biomarkers in Alicyclobacillus acidoterrestris
Source: Front Microbiol. 2018 Dec 4;9:3032. doi: 10.3389/fmicb.2018.03032 (PMC6288362; doi:10.3389/fmicb.2018.03032)
Supplement: Supplementary file 2 [file Data_Sheet_2.PDF]

Analysis Information

Report Type	Protein-Peptide Summary by Spot	Analysis Type	Combined (MS+MS/MS)
Sample Set Name	Sample project20150611	Database	TEST_M
Analysis Name	646	Creation Date	06/23/2017 21:09:32
Reported By	06/23/2017 21:25:59 - admin	Last Modified	06/23/2017 21:12:09

MS Acq. : Proc. Methods	(Unspecified) : (Unspecified)
Interpretation Method	(Unspecified)

**Gel Idx/Pos**	223/I23	**Instr./Gel Origin**	BA2151/full sequence test 20150515	**Process Status**	Analysis Succeeded
**Plate [#] Name**	[3] full sequence test 20150515	**Instrument Sample Name**		**Spectra**	11

Rank	Protein Name	Species	Accession No.	Protein MW	Protein PI	Pep. Count	Protein Score	Protein Score C. I. %	Total Ion Score	Total Ion C. I. %
1	Uncharacterized protein OS=Alicyclobacillus acidoterrestris (strain ATCC 49025 / DSM 3922 / CIP 106132 / NCIMB 13137 / GD3B) GN=N007_00440 PE=4 SV=1		tr|T0C5G8|T0C5G8_ALIAG	63753	4.64	7	209	100	195	100

Peptide Information

Calc. Mass	Obsrv. Mass	± da	± ppm	Start Seq.	End Seq.	Sequence	Ion Score	C. I. %	Modification	Rank	Result Type
852.4825	852.4877	0.0052	6	313	319	YKSDVLK					Mascot
949.4526	949.4538	0.0012	1	249	255	NPNYWQK					Mascot
1364.6693	1364.6747	0.0054	4	256	267	DSDGNKLPYLDK					Mascot
1364.6693	1364.6747	0.0054	4	256	267	DSDGNKLPYLDK					Mascot
1628.7803	1628.7736	-0.0067	-4	350	363	QAIEYATDDSQFIK					Mascot
1628.7803	1628.7736	-0.0067	-4	350	363	QAIEYATDDSQFIK	34	99.807			Mascot
2848.3782	2848.384	0.0058	2	223	248	LDTSEAMGTGPFEVQTNNQNEVVLVR					Mascot
2848.3782	2848.384	0.0058	2	223	248	LDTSEAMGTGPFEVQTNNQNEVVLVR	117	100			Mascot
2864.373	2864.3813	0.0083	3	223	248	LDTSEAMGTGPFEVQTNNQNEVVLVR			Oxidation (M)[7]		Mascot
2864.373	2864.3813	0.0083	3	223	248	LDTSEAMGTGPFEVQTNNQNEVVLVR	118	100	Oxidation (M)[7]		Mascot
2908.2729	2908.3877	0.1148	39	402	426	ELMKEAGYANGFTAEYWNENNPTDK			Oxidation (M)[3]		Mascot
3143.6558	3143.6528	-0.003	-1	320	347	QPQNSIYYIGLNMKPTLDGKPNPLSNLK					Mascot
3143.6558	3143.6528	-0.003	-1	320	347	QPQNSIYYIGLNMKPTLD	42	99.965			Mascot

3159.6506	3159.6377	-0.0129	-4	320	347	GKPNPLSNLK QPQNSIYYIGLNMKPTLD GKPNPLSNLK			Oxidation (M)[13]		Mascot

2	Uncharacterized protein OS=Alicyclobacillus acidoterrestris (strain ATCC 49025 / DSM 3922 / CIP 106132 / NCIMB 13137 / GD3B) GN=N007_08170 PE=4 SV=1	tr|T0D2D0|T0D2D0_ALIAG	6562.5	6.23	4	28	0

Peptide Information

Calc. Mass	Obsrv. Mass	± da	± ppm	Start Seq.	End Seq.	Sequence	Ion Score	C. I. %	Modification	Rank	Result Type
862.4516	862.3826	-0.069	-80	48	55	TVDEVTAK					Mascot
1364.7533	1364.6747	-0.0786	-58	29	40	HLLDAIEALDVR					Mascot
1364.7533	1364.6747	-0.0786	-58	29	40	HLLDAIEALDVR					Mascot
2225.123	2225.1125	-0.0105	-5	29	47	HLLDAIEALDVRISDEQCK			Carbamidomethyl (C)[18]		Mascot
2819.5212	2819.3687	-0.1525	-54	1	26	MPLARFALAWVLHHPAV TCAIIGSTK			Oxidation (M)[1]		Mascot

3	Uncharacterized protein OS=Alicyclobacillus acidoterrestris (strain ATCC 49025 / DSM 3922 / CIP 106132 / NCIMB 13137 / GD3B) GN=N007_00875 PE=4 SV=1	tr|T0DFA8|T0DFA8_ALIAG	21964.2	5.4	5	21	0

Peptide Information

Calc. Mass	Obsrv. Mass	± da	± ppm	Start Seq.	End Seq.	Sequence	Ion Score	C. I. %	Modification	Rank	Result Type
961.4044	961.4347	0.0303	32	1	9	MSEHEGGSK					Mascot
977.3992	977.448	0.0488	50	1	9	MSEHEGGSK			Oxidation (M)[1]		Mascot
977.3992	977.448	0.0488	50	1	9	MSEHEGGSK			Oxidation (M)[1]		Mascot
1059.5615	1059.4734	-0.0881	-83	181	190	AAELVCNALR					Mascot
1105.4943	1105.5713	0.077	70	1	10	MSEHEGGSKK			Oxidation (M)[1]		Mascot
2920.4622	2920.4492	-0.013	-4	81	106	LNQAIEADDGWTGFQAL VSDTMLRIR					Mascot
3181.5933	3181.6082	0.0149	5	150	180	ADVTVADLGLLVIGTSQY LADTSAGTEECLR					Mascot

4	Uncharacterized protein OS=Alicyclobacillus acidoterrestris (strain ATCC 49025 / DSM 3922 / CIP 106132 / NCIMB 13137 / GD3B) GN=N007_21030 PE=4 SV=1	tr|T0DN80|T0DN80_ALIAG	7707	6.1	3	20	0

Peptide Information

Calc. Mass	Obsrv. Mass	± da	± ppm	Start Seq.	End Seq.	Sequence	Ion Score	C. I. %	Modification	Rank	Result Type
981.4999	981.4476	-0.0523	-53	43	50	SVYQLESR					Mascot
1128.6082	1128.5334	-0.0748	-66	33	42	AEAEVPIMLR					Mascot

3126.79	3126.6416	-0.1484	-47	2	30	VVMLYVAGLLFTFLIAYAL GRVVGLSQGR					Mascot
3142.7849	3142.655	-0.1299	-41	2	30	VVMLYVAGLLFTFLIAYAL GRVVGLSQGR			Oxidation (M)[3]		Mascot

5	Uncharacterized protein OS=Alicyclobacillus acidoterrestris (strain ATCC 49025 / DSM 3922 / CIP 106132 / NCIMB 13137 / GD3B) GN=N007_05825 PE=4 SV=1	tr|T0BTQ3|T0BTQ3_ALIAG	31813.2	8.77	6	20	0

Peptide Information

Calc. Mass	Obsrv. Mass	± da	± ppm	Start Seq.	End Seq.	Sequence	Ion Score	C. I. %	Modification	Rank	Result Type
852.4825	852.4877	0.0052	6	224	230	QYLSTLK					Mascot
989.5262	989.4439	-0.0823	-83	243	251	LSVEDISAR					Mascot
1083.4888	1083.4541	-0.0347	-32	203	212	LGYSPSSCNR					Mascot
1133.6096	1133.5133	-0.0963	-85	194	202	DVIRQISMR			Oxidation (M)[8]		Mascot
2861.3359	2861.3843	0.0484	17	2	27	ADLRVHAAEHGFNAPSIC GTFAFCGR			Carbamidomethyl (C)[18,24]		Mascot
2878.3335	2878.387	0.0535	19	1	27	MADLRVHAAEHGFNAPSI CGTFAFCGR					Mascot
2935.355	2935.3711	0.0161	5	1	27	MADLRVHAAEHGFNAPSI CGTFAFCGR			Carbamidomethyl (C)[19]		Mascot

6	Uncharacterized protein OS=Alicyclobacillus acidoterrestris (strain ATCC 49025 / DSM 3922 / CIP 106132 / NCIMB 13137 / GD3B) GN=N007_14305 PE=4 SV=1	tr|T0BPM6|T0BPM6_ALIAG	32028.1	6.2	6	19	0

Peptide Information

Calc. Mass	Obsrv. Mass	± da	± ppm	Start Seq.	End Seq.	Sequence	Ion Score	C. I. %	Modification	Rank	Result Type
949.4407	949.4538	0.0131	14	1	8	MADDQTIR					Mascot
989.5309	989.4439	-0.087	-88	223	230	TQAVCVRR			Carbamidomethyl (C)[5]		Mascot
1105.5419	1105.5713	0.0294	27	1	9	MADDQTIRR					Mascot
1386.6284	1386.6669	0.0385	28	193	203	YEDFVRDEASR					Mascot
2800.3909	2800.4111	0.0202	7	119	141	DVLVSLCHFVVEKPEYH QMNALR			Carbamidomethyl (C)[7], Oxidation (M)[19]		Mascot
2800.3909	2800.4111	0.0202	7	119	141	DVLVSLCHFVVEKPEYH QMNALR			Carbamidomethyl (C)[7], Oxidation (M)[19]		Mascot
3127.5815	3127.6577	0.0762	24	116	141	DLRDVLVSLCHFVVEKPE YHQMNALR			Oxidation (M)[22]		Mascot

7	Tyrosine recombinase XerD OS=Alicyclobacillus acidoterrestris (strain ATCC 49025 / DSM 3922 / CIP 106132 / NCIMB 13137 / GD3B) GN=xerD PE=3 SV=1	tr|T0C7H0|T0C7H0_ALIAG	33776.6	8.94	6	19	0

Peptide Information

Calc. Mass	Obsrv. Mass	± da	± ppm	Start Seq.	End Seq.	Sequence	Ion Score	C. I. %	Modification	Rank	Result Type

977.4509	977.448	-0.0029	-3	1	7	MDEWIQR					Mascot
977.4509	977.448	-0.0029	-3	1	7	MDEWIQR					Mascot
1120.6361	1120.5731	-0.063	-56	227	236	KYAQEAGILK					Mascot
1386.7812	1386.6669	-0.1143	-82	66	78	ANATISRNLASIR					Mascot
1628.8353	1628.7736	-0.0617	-38	133	146	DYAMLELLYATGIR					Mascot
1628.8353	1628.7736	-0.0617	-38	133	146	DYAMLELLYATGIR					Mascot
2877.5735	2877.3862	-0.1873	-65	176	200	IIPIGEYAVQALDNYLQQA RPHLVR					Mascot
2935.4744	2935.3711	-0.1033	-35	40	65	RGSCVIQEVQQHHIAAYL GYLHEAGR					Mascot

8	Uncharacterized protein OS=Alicyclobacillus acidoterrestris (strain ATCC 49025 / DSM 3922 / CIP 106132 / NCIMB 13137 / GD3B) GN=N007_03025 PE=4 SV=1	tr|T0DQ14|T0DQ14_ALIAG	16209.2	7.93	4	19	0

Peptide Information

Calc. Mass	Obsrv. Mass	± da	± ppm	Start Seq.	End Seq.	Sequence	Ion Score	C. I. %	Modification	Rank	Result Type
949.5353	949.4538	-0.0815	-86	84	91	LTIEAFQK					Mascot
960.4495	960.3914	-0.0581	-60	92	99	YYSMTAPK					Mascot
2864.437	2864.3813	-0.0557	-19	58	83	QSTELALQKLSNEIVSSQ LSESESEK					Mascot
2864.437	2864.3813	-0.0557	-19	58	83	QSTELALQKLSNEIVSSQ LSESESEK					Mascot
2908.6296	2908.3877	-0.2419	-83	10	35	ASFKYLFIAYGIASVIAIIVV LMYDK					Mascot
2924.6245	2924.3462	-0.2783	-95	10	35	ASFKYLFIAYGIASVIAIIVV LMYDK			Oxidation (M)[23]		Mascot

9	Uncharacterized protein OS=Alicyclobacillus acidoterrestris (strain ATCC 49025 / DSM 3922 / CIP 106132 / NCIMB 13137 / GD3B) GN=N007_18560 PE=4 SV=1	tr|T0DNY5|T0DNY5_ALIAG	17844.8	5.43	4	19	0

Peptide Information

Calc. Mass	Obsrv. Mass	± da	± ppm	Start Seq.	End Seq.	Sequence	Ion Score	C. I. %	Modification	Rank	Result Type
1021.4619	1021.4702	0.0083	8	97	104	DLDTECIR			Carbamidomethyl (C)[6]		Mascot
1120.5238	1120.5731	0.0493	44	12	21	EIMANAVCGR			Carbamidomethyl (C)[8]		Mascot
2864.457	2864.3813	-0.0757	-26	108	134	EVHVSVIQSPNCLDATLA GNGSEVLVR			Carbamidomethyl (C)[12]		Mascot
2864.457	2864.3813	-0.0757	-26	108	134	EVHVSVIQSPNCLDATLA GNGSEVLVR			Carbamidomethyl (C)[12]		Mascot
3165.5957	3165.6033	0.0076	2	105	134	DSREVHVSVIQSPNCLDA TLAGNGSEVLVR					Mascot

10	Uncharacterized protein OS=Alicyclobacillus acidoterrestris (strain ATCC 49025 / DSM 3922 / CIP 106132 / NCIMB 13137 / GD3B) GN=N007_02240	tr|T0CJE4|T0CJE4_ALIAG	8270.2	9.98	3	19	0

PE=4 SV=1

Peptide Information

Calc. Mass	Obsrv. Mass	± da	± ppm	Start Seq.	End Seq.	Sequence	Ion Score	C. I. %	Modification	Rank	Result Type
1120.531	1120.5731	0.0421	38	68	76	FYTTFSNQL					Mascot
1752.9102	1752.9556	0.0454	26	51	67	VPAVVAQNPGMAEGWV K					Mascot
2870.4182	2870.3818	-0.0364	-13	51	76	VPAVVAQNPGMAEGWV KFYTTFSNQL			Oxidation (M)[11]		Mascot

Gel Idx/Pos	224/I24	Instr./Gel Origin	BA2151/full sequence test 20150515	Process Status	Analysis Succeeded
Plate [#] Name	[3] full sequence test 20150515	Instrument Sample Name		Spectra	11

Rank	Protein Name	Species	Accession No.	Protein MW	Protein PI	Pep. Count	Protein Score	Protein Score C. I. %	Total Ion Score	Total Ion C. I. %
1	Probable thiol peroxidase OS=Alicyclobacillus acidoterrestris (strain ATCC 49025 / DSM 3922 / CIP 106132 / NCIMB 13137 / GD3B) GN=tpx PE=3 SV=1		tr|T0CJI3|T0CJI3_ALIAG	18634.5	5.23	17	317	100	171	100

Peptide Information

Calc. Mass	Obsrv. Mass	± da	± ppm	Start Seq.	End Seq.	Sequence	Ion Score	C. I. %	Modification	Rank	Result Type
958.4775	958.478	0.0005	1	108	115	VMTLSDHR					Mascot
974.4724	974.4821	0.0097	10	108	115	VMTLSDHR			Oxidation (M)[2]		Mascot
977.4687	977.4724	0.0037	4	25	33	VGDTAPDFR					Mascot
977.4687	977.4724	0.0037	4	25	33	VGDTAPDFR					Mascot
980.4796	980.4902	0.0106	11	71	78	RFNEEASK					Mascot
1005.4571	1005.4623	0.0052	5	99	107	WCGAAGVER			Carbamidomethyl (C)[2]		Mascot
1005.4571	1005.4623	0.0052	5	99	107	WCGAAGVER	10	38.116	Carbamidomethyl (C)[2]		Mascot
1066.5416	1066.5737	0.0321	30	136	145	AVFVVDSSDK					Mascot
1161.5582	1161.5696	0.0114	10	98	107	RWCGAAGVER			Carbamidomethyl (C)[3]		Mascot
1397.6519	1397.6637	0.0118	8	116	128	TMGFGDAYGTHIK					Mascot
1397.6519	1397.6637	0.0118	8	116	128	TMGFGDAYGTHIK	18	89.777			Mascot
1413.6467	1413.6542	0.0075	5	116	128	TMGFGDAYGTHIK			Oxidation (M)[2]		Mascot
1413.6467	1413.6542	0.0075	5	116	128	TMGFGDAYGTHIK	37	99.88	Oxidation (M)[2]		Mascot
1560.8115	1560.8187	0.0072	5	34	48	LVANDLSEVSLADSK					Mascot
1861.8538	1861.8926	0.0388	21	116	131	TMGFGDAYGTHIKEYR			Oxidation (M)[2]		Mascot
1930.9904	1930.9919	0.0015	1	53	70	IISVVPSLDTGVCDAQTR			Carbamidomethyl (C)[13]		Mascot
1930.9904	1930.9919	0.0015	1	53	70	IISVVPSLDTGVCDAQTR	101	100	Carbamidomethyl (C)[13]		Mascot
1986.0907	1986.0905	-0.0002	0	79	97	LGDNVVVLTVSADLPFAQK					Mascot
2071.1475	2071.1511	0.0036	2	6	24	EGAFLFPDKPVTLIGPELK					Mascot
2071.1475	2071.1511	0.0036	2	6	24	EGAFLFPDKPVTLIGPELK	22	95.958			Mascot
2087.0913	2087.1211	0.0298	14	53	71	IISVVPSLDTGVCDAQTRR			Carbamidomethyl (C)[13]		Mascot
2142.1919	2142.1938	0.0019	1	79	98	LGDNVVVLTVSADLPFAQKR					Mascot
2142.1919	2142.1938	0.0019	1	79	98	LGDNVVVLTVSADLPFAQKR					Mascot
2541.3711	2541.3914	0.0203	8	2	24	ANEREGAFLFPDKPVTLIGPELK					Mascot

Calc. Mass	Obsrv. Mass	± da	± ppm	Start Seq.	End Seq.	Sequence	Ion Score	C. I. %	Modification	Rank	Result Type
2589.2983	2589.2993	0.001	0	146	169	IVYVQYVPAAGEHPNYEAALDAAK					Mascot
3636.822	3636.9004	0.0784	22	136	169	AVFVVDSSDKIVYVQYVPAAGEHPNYEAALDAAK					Mascot

2	Uncharacterized protein OS=Alicyclobacillus acidoterrestris (strain ATCC 49025 / DSM 3922 / CIP 106132 / NCIMB 13137 / GD3B) GN=N007_07030 PE=4 SV=1	tr|T0C0U9|T0C0U9_ALIAG	17080	4.53	6	29	0

Peptide Information

Calc. Mass	Obsrv. Mass	± da	± ppm	Start Seq.	End Seq.	Sequence	Ion Score	C. I. %	Modification	Rank	Result Type
1033.4546	1033.4572	0.0026	3	66	75	GQSEVGDDAR					Mascot
1066.5021	1066.5737	0.0716	67	2	10	AELQCVTMR			Oxidation (M)[8]		Mascot
1189.5557	1189.5767	0.021	18	66	76	GQSEVGDDARR					Mascot
2067.0176	2067.0957	0.0781	38	11	28	IGDELYGARVEQVMSVER			Oxidation (M)[14]		Mascot
2085.0833	2085.1602	0.0769	37	20	37	VEQVMSVERMLDISPVPR					Mascot
2098.0056	2098.1321	0.1265	60	2	19	AELQCVTMRIGDELYGAR			Carbamidomethyl (C)[5], Oxidation (M)[8]		Mascot

3	Uncharacterized protein OS=Alicyclobacillus acidoterrestris (strain ATCC 49025 / DSM 3922 / CIP 106132 / NCIMB 13137 / GD3B) GN=N007_03545 PE=3 SV=1	tr|T0BXV8|T0BXV8_ALIAG	39070.5	5.19	9	29	0

Peptide Information

Calc. Mass	Obsrv. Mass	± da	± ppm	Start Seq.	End Seq.	Sequence	Ion Score	C. I. %	Modification	Rank	Result Type
875.4655	875.4037	-0.0618	-71	81	88	TVIMGNPK			Oxidation (M)[4]		Mascot
958.5316	958.478	-0.0536	-56	211	219	RAVDELGAK					Mascot
1003.5465	1003.4633	-0.0832	-83	343	350	IAQMRNVR			Oxidation (M)[4]		Mascot
1021.5537	1021.459	-0.0947	-93	348	355	NVRSNYLR					Mascot
1087.5493	1087.4867	-0.0626	-58	1	9	MEIFSYLGK					Mascot
1093.5273	1093.4983	-0.029	-27	164	174	AAFGTDDLAGR					Mascot
1103.5442	1103.5101	-0.0341	-31	1	9	MEIFSYLGK			Oxidation (M)[1]		Mascot
1912.9838	1912.9878	0.004	2	310	326	VDNIYNIISNIFAMSAK					Mascot
1954.9327	1954.9434	0.0107	5	45	60	MWTYAREDDAILDALR			Oxidation (M)[1]		Mascot
2603.322	2603.3376	0.0156	6	27	50	AIIAIHDTTLGPALGGCRMWTYAR			Oxidation (M)[19]		Mascot

4	Uncharacterized protein OS=Alicyclobacillus acidoterrestris (strain ATCC 49025 / DSM 3922 / CIP 106132 / NCIMB 13137 / GD3B) GN=N007_10530 PE=4 SV=1	tr|T0BV11|T0BV11_ALIAG	41296.8	8.04	9	27	0

Peptide Information

Calc. Mass	Obsrv. Mass	± da	± ppm	Start Seq.	End Seq.	Sequence	Ion Score	C. I. %	Modification	Rank	Result Type
946.5039	946.4335	-0.0704	-74	328	334	ARHMIYR					Mascot
1087.5854	1087.4867	-0.0987	-91	2	11	TSATPTRPTR					Mascot
1089.58	1089.495	-0.085	-78	27	34	QIERWTTR					Mascot
1117.5426	1117.5042	-0.0384	-34	160	167	HVEWYVER					Mascot
1186.644	1186.597	-0.047	-40	294	303	QISYGGRIHR					Mascot
1214.6351	1214.6406	0.0055	5	16	25	LFHDVMPLSR					Mascot
1214.6351	1214.6406	0.0055	5	16	25	LFHDVMPLSR					Mascot
1218.626	1218.6376	0.0116	10	1	11	MTSATPTRPTR					Mascot
1230.63	1230.6356	0.0056	5	16	25	LFHDVMPLSR			Oxidation (M)[6]		Mascot
1945.8976	1945.993	0.0954	49	335	350	FGPTACMFYFATVVYR			Carbamidomethyl (C)[6], Oxidation (M)[7]		Mascot
2589.2241	2589.2993	0.0752	29	330	350	HMIYRFGPTACMFYFATV VYR			Oxidation (M)[2]		Mascot

5	Phosphate starvation protein PhoH OS=Alicyclobacillus acidoterrestris (strain ATCC 49025 / DSM 3922 / CIP 106132 / NCIMB 13137 / GD3B) GN=N007_20335 PE=4 SV=1	tr|T0DSZ6|T0DSZ 6_ALIAG	36222.8	6.01	7	26	0	6	0

Peptide Information

Calc. Mass	Obsrv. Mass	± da	± ppm	Start Seq.	End Seq.	Sequence	Ion Score	C. I. %	Modification	Rank	Result Type
977.5275	977.4724	-0.0551	-56	276	284	TSGLVHAHR					Mascot
977.5275	977.4724	-0.0551	-56	276	284	TSGLVHAHR					Mascot
980.4466	980.4902	0.0436	44	1	8	MTDNQTVR			Oxidation (M)[1]		Mascot
1017.4734	1017.455	-0.0184	-18	318	327	TDEGEPSAIV					Mascot
1216.6572	1216.6495	-0.0077	-6	176	186	LGFLPGDLQEK					Mascot
1397.7532	1397.6637	-0.0895	-64	144	156	TYLAVAMAVMALK			Oxidation (M)[7]		Mascot
1397.7532	1397.6637	-0.0895	-64	144	156	TYLAVAMAVMALK	6	0	Oxidation (M)[7]		Mascot
1413.748	1413.6542	-0.0938	-66	144	156	TYLAVAMAVMALK			Oxidation (M)[7,10]		Mascot
1413.748	1413.6542	-0.0938	-66	144	156	TYLAVAMAVMALK			Oxidation (M)[7,10]		Mascot
1560.8315	1560.8187	-0.0128	-8	214	227	GNIEIAPLAYMRGR					Mascot
2085.0435	2085.1602	0.1167	56	72	89	QGIQLGDADYRYVIQMAK			Oxidation (M)[16]		Mascot

6	Uncharacterized protein OS=Alicyclobacillus acidoterrestris (strain ATCC 49025 / DSM 3922 / CIP 106132 / NCIMB 13137 / GD3B) GN=N007_15645 PE=3 SV=1	tr|T0CXJ8|T0CXJ8 _ALIAG	8833.3	5.3	5	25	0

Peptide Information

Calc. Mass	Obsrv. Mass	± da	± ppm	Start Seq.	End Seq.	Sequence	Ion Score	C. I. %	Modification	Rank	Result Type
958.5568	958.478	-0.0788	-82	2	10	KADVIVDAK					Mascot
1060.553	1060.4851	-0.0679	-64	11	20	GLSCPMPIVK			Oxidation (M)[6]		Mascot
1089.5973	1089.495	-0.1023	-94	1	10	MKADVIVDAK					Mascot
1105.5922	1105.4927	-0.0995	-90	1	10	MKADVIVDAK			Oxidation (M)[1]		Mascot
1117.5745	1117.5042	-0.0703	-63	11	20	GLSCPMPIVK			Carbamidomethyl (C)[4], Oxidation (M)[6]		Mascot
1913.0236	1912.9878	-0.0358	-19	3	20	ADVIVDAKGLSCPMPIVK			Carbamidomethyl (C)[12]		Mascot
2066.9622	2067.0957	0.1335	65	24	41	AMEELEVGQIMELQSTDK			Oxidation (M)[2]		Mascot

7	Uncharacterized protein OS=Alicyclobacillus acidoterrestris (strain ATCC 49025 / DSM 3922 / CIP 106132 / NCIMB 13137 / GD3B) GN=N007_17930 PE=4 SV=1	tr|T0DP59|T0DP59_ALIAG	24868.6	9.14	7	23	0		

Peptide Information

Calc. Mass	Obsrv. Mass	± da	± ppm	Start Seq.	End Seq.	Sequence	Ion Score	C. I. %	Modification	Rank	Result Type
1087.5103	1087.4867	-0.0236	-22	15	23	LSACWSHAR			Carbamidomethyl (C)[4]		Mascot
1091.5632	1091.5027	-0.0605	-55	144	152	AWLFSNTPR					Mascot
1186.5898	1186.597	0.0072	6	15	24	LSACWSHARR					Mascot
1435.6812	1435.6284	-0.0528	-37	63	73	EIPSEERYEQR					Mascot
1861.9291	1861.8926	-0.0365	-20	115	130	LVTFLEDGNLEIDNNR					Mascot
1930.9327	1930.9919	0.0592	31	40	56	TATTAREGLDYCNQLFK					Mascot
1930.9327	1930.9919	0.0592	31	40	56	TATTAREGLDYCNQLFK					Mascot
2611.1816	2611.2302	0.0486	19	1	23	MDGYQGYNDVPNVKLSACWSHAR					Mascot

8	30S ribosomal protein S3 OS=Alicyclobacillus acidoterrestris (strain ATCC 49025 / DSM 3922 / CIP 106132 / NCIMB 13137 / GD3B) GN=rpsC PE=3 SV=1	tr|T0BPA8|T0BPA8_ALIAG	25070.5	10.06	6	23	0	0	0

Peptide Information

Calc. Mass	Obsrv. Mass	± da	± ppm	Start Seq.	End Seq.	Sequence	Ion Score	C. I. %	Modification	Rank	Result Type
1005.5258	1005.4623	-0.0635	-63	136	143	QAIQRSMR			Oxidation (M)[7]		Mascot
1005.5258	1005.4623	-0.0635	-63	136	143	QAIQRSMR	0	0	Oxidation (M)[7]		Mascot
1159.5953	1159.5768	-0.0185	-16	49	59	DAAVASIDIER					Mascot
1200.6736	1200.6257	-0.0479	-40	12	21	IGIIRDWEAK					Mascot
1214.6675	1214.6406	-0.0269	-22	1	11	MGQKVNPVGLR			Oxidation (M)[1]		Mascot
1214.6675	1214.6406	-0.0269	-22	1	11	MGQKVNPVGLR			Oxidation (M)[1]		Mascot

Calc. Mass	Obsrv. Mass	± da	± ppm	Start Seq.	End Seq.	Sequence	Ion Score	C. I. %	Modification	Rank	Result Type
2093.1648	2093.1274	-0.0374	-18	60	79	AANRINVTVHTAKPGMVIGK			Oxidation (M)[16]		Mascot
2180.0981	2179.9946	-0.1035	-47	180	199	ADIDYALSEAHTTYGRIGVK					Mascot

9	Uncharacterized protein OS=Alicyclobacillus acidoterrestris (strain ATCC 49025 / DSM 3922 / CIP 106132 / NCIMB 13137 / GD3B) GN=N007_10970 PE=4 SV=1	tr|T0BKH4|T0BKH4_ALIAG	24395.5	9.16	9	22	0

Peptide Information

Calc. Mass	Obsrv. Mass	± da	± ppm	Start Seq.	End Seq.	Sequence	Ion Score	C. I. %	Modification	Rank	Result Type
958.5203	958.478	-0.0423	-44	202	210	ILEGKGLND					Mascot
980.4651	980.4902	0.0251	26	195	201	IEKCMQR			Carbamidomethyl (C)[4], Oxidation (M)[5]		Mascot
1077.5179	1077.4998	-0.0181	-17	21	30	CKLGGTPNCK			Carbamidomethyl (C)[1]		Mascot
1077.5179	1077.4998	-0.0181	-17	21	30	CKLGGTPNCK			Carbamidomethyl (C)[1]		Mascot
1093.5493	1093.4983	-0.051	-47	198	206	CMQRILEGK			Oxidation (M)[2]		Mascot
1101.5179	1101.4633	-0.0546	-50	1	9	MEHKSMNPK					Mascot
1117.5129	1117.5042	-0.0087	-8	1	9	MEHKSMNPK			Oxidation (M)[1]		Mascot
1175.5225	1175.569	0.0465	40	76	84	EFCSLNFMK			Carbamidomethyl (C)[3]		Mascot
1214.6021	1214.6406	0.0385	32	66	75	RNVVMMSAFK			Oxidation (M)[5,6]		Mascot
1214.6021	1214.6406	0.0385	32	66	75	RNVVMMSAFK			Oxidation (M)[5,6]		Mascot
2071.0779	2071.1511	0.0732	35	119	135	MEPILKEYIHEAIEVEK					Mascot
2071.0779	2071.1511	0.0732	35	119	135	MEPILKEYIHEAIEVEK					Mascot
2087.073	2087.1211	0.0481	23	119	135	MEPILKEYIHEAIEVEK			Oxidation (M)[1]		Mascot
2141.989	2142.1938	0.2048	96	67	84	NVVMMSAFKEFCSLNFMK			Oxidation (M)[4]		Mascot
2141.989	2142.1938	0.2048	96	67	84	NVVMMSAFKEFCSLNFMK			Oxidation (M)[4]		Mascot

10	Serine protein kinase OS=Alicyclobacillus acidoterrestris (strain ATCC 49025 / DSM 3922 / CIP 106132 / NCIMB 13137 / GD3B) GN=N007_01390 PE=4 SV=1	tr|T0CZQ9|T0CZQ9_ALIAG	33566.7	5.27	8	22	0

Peptide Information

Calc. Mass	Obsrv. Mass	± da	± ppm	Start Seq.	End Seq.	Sequence	Ion Score	C. I. %	Modification	Rank	Result Type
980.5159	980.4902	-0.0257	-26	118	125	RGLEQYSK					Mascot
1019.5302	1019.4506	-0.0796	-78	191	198	VLMSEERR					Mascot
1091.4972	1091.5027	0.0055	5	163	172	IEGNLCPSCR					Mascot
1200.7021	1200.6257	-0.0764	-64	98	109	ILLLMGPVSGGK			Oxidation (M)[5]		Mascot
1212.6008	1212.6313	0.0305	25	237	246	AYRFDGELNK					Mascot

1218.6365	1218.6376	0.0011	1	199	210	IGIGTFSPSDPK		Mascot
1435.6603	1435.6284	-0.0319	-22	163	174	IEGNLCPSCRMR	Carbamidomethyl (C)[6]	Mascot
2141.0444	2141.1758	0.1314	61	173	190	MRLDEEFGGHIEQVPVE R		Mascot

Gel Idx/Pos	225/J1	Instr./Gel Origin	BA2151/full sequence test 20150515	Process Status	Analysis Succeeded
Plate [#] Name	[3] full sequence test 20150515	Instrument Sample Name		Spectra	11

Rank	Protein Name	Species	Accession No.	Protein MW	Protein PI	Pep. Count	Protein Score	Protein Score C. I. %	Total Ion Score	Total Ion C. I. %
1	Elongation factor Tu OS=Alicyclobacillus acidoterrestris (strain ATCC 49025 / DSM 3922 / CIP 106132 / NCIMB 13137 / GD3B) GN=tuf PE=3 SV=1		tr|T0D867|T0D867_ALIAG	43353	5.09	10	40	55.022	7	0

Peptide Information

Calc. Mass	Obsrv. Mass	± da	± ppm	Start Seq.	End Seq.	Sequence	Ion Score	C. I. %	Modification	Rank	Result Type
829.489	829.4385	-0.0505	-61	232	238	VERGQLK					Mascot
1124.5769	1124.5674	-0.0095	-8	255	264	TVATGIEMFR					Mascot
1140.5718	1140.5729	0.0011	1	255	264	TVATGIEMFR			Oxidation (M)[8]		Mascot
1268.6667	1268.6912	0.0245	19	255	265	TVATGIEMFRK			Oxidation (M)[8]		Mascot
1667.8235	1667.858	0.0345	21	239	253	VGDEVEIVGLHEESR					Mascot
1687.9014	1687.9285	0.0271	16	266	281	LLDFAEAGDNIGALLR	7	0			Mascot
1795.9185	1795.9275	0.009	5	239	254	VGDEVEIVGLHEESRK					Mascot
1815.9963	1816.0203	0.024	13	265	281	KLLDFAEAGDNIGALLR					Mascot
1976.9556	1976.984	0.0284	14	321	335	HTPFFNGYRPQFYFR					Mascot
2153.0947	2153.1362	0.0415	19	206	224	DTSKPFLMPVEDVFTITGR					Mascot
2743.4236	2743.4614	0.0378	14	291	315	GQVVCKPGSINPHTQFAAEVYVLTK			Carbamidomethyl (C)[5]		Mascot

Rank	Protein Name	Species	Accession No.	Protein MW	Protein PI	Pep. Count	Protein Score	Protein Score C. I. %	Total Ion Score	Total Ion C. I. %
2	Uncharacterized protein OS=Alicyclobacillus acidoterrestris (strain ATCC 49025 / DSM 3922 / CIP 106132 / NCIMB 13137 / GD3B) GN=N007_18525 PE=4 SV=1		tr|T0DUD9|T0DUD9_ALIAG	51393.4	10.24	11	33	0		

Peptide Information

Calc. Mass	Obsrv. Mass	± da	± ppm	Start Seq.	End Seq.	Sequence	Ion Score	C. I. %	Modification	Rank	Result Type
935.4866	935.5488	0.0622	66	376	383	ETIAKMDK					Mascot
935.4866	935.5488	0.0622	66	376	383	ETIAKMDK					Mascot
1106.5841	1106.554	-0.0301	-27	253	262	GALIEATFER					Mascot
1124.5616	1124.5674	0.0058	5	3	12	MSVEISSSLR			Oxidation (M)[1]		Mascot
1146.63	1146.5844	-0.0456	-40	381	390	MDKLASQVVR					Mascot
1218.6631	1218.6602	-0.0029	-2	137	146	VFDPVAKTWR					Mascot
1557.925	1557.8602	-0.0648	-42	83	97	LGPVLGLYVDLIASK					Mascot
1557.925	1557.8602	-0.0648	-42	83	97	LGPVLGLYVDLIASK					Mascot

Calc. Mass	Obsrv. Mass	± da	± ppm	Start Seq.	End Seq.	Sequence	Ion Score	C. I. %	Modification	Rank	Result Type
1687.8545	1687.9285	0.074	44	3	17	MSVEISSSLRGNHVR			Oxidation (M)[1]		Mascot
2290.1714	2290.22	0.0486	21	305	324	AVVQDTVELPKLDGQPYDFR					Mascot
2310.2129	2310.1228	-0.0901	-39	438	458	LSLEYLLEYAIRATGYGGAIH					Mascot
2333.2361	2333.2141	-0.022	-9	231	251	NDVYVKPPGGSQGVSIYRLQR					Mascot
2379.1367	2379.2427	0.106	45	210	230	HLPGTTMCTSGAQLYREVMAR			Carbamidomethyl (C)[8]		Mascot

3	GTP cyclohydrolase 1 OS=Alicyclobacillus acidoterrestris (strain ATCC 49025 / DSM 3922 / CIP 106132 / NCIMB 13137 / GD3B) GN=folE PE=3 SV=1	tr|T0D455|T0D455_ALIAG	21118.9	6.16	7	33	0

Peptide Information

Calc. Mass	Obsrv. Mass	± da	± ppm	Start Seq.	End Seq.	Sequence	Ion Score	C. I. %	Modification	Rank	Result Type
1161.6991	1161.6166	-0.0825	-71	107	116	FARLVELVSK					Mascot
1667.9592	1667.858	-0.1012	-61	91	106	AHIAYLPSGRVVGLSK					Mascot
1680.9756	1680.8429	-0.1327	-79	110	123	LVELVSKRPQVQER					Mascot
1819.8928	1819.8904	-0.0024	-1	124	139	MTQQIADAVMNVLEPK			Oxidation (M)[1,10]		Mascot
2318.0588	2318.2314	0.1726	74	73	90	DIDYYTFCEHHLIPFYGK			Carbamidomethyl (C)[8]		Mascot
2347.1677	2347.2166	0.0489	21	170	190	LGAFSEHASLVQEFEQALSRQ					Mascot
2349.2522	2349.2131	-0.0391	-17	17	37	SLLQLIGENPNREGLLDTPNR					Mascot

4	GTPase Obg OS=Alicyclobacillus acidoterrestris (strain ATCC 49025 / DSM 3922 / CIP 106132 / NCIMB 13137 / GD3B) GN=obgE PE=3 SV=1	tr|T0C7Q6|T0C7Q6_ALIAG	46910.3	5.85	10	30	0

Peptide Information

Calc. Mass	Obsrv. Mass	± da	± ppm	Start Seq.	End Seq.	Sequence	Ion Score	C. I. %	Modification	Rank	Result Type
807.4723	807.4531	-0.0192	-24	348	353	VYRLEK					Mascot
935.5196	935.5488	0.0292	31	150	156	YLELELR					Mascot
935.5196	935.5488	0.0292	31	150	156	YLELELR					Mascot
1180.5997	1180.6525	0.0528	45	2	11	FTDHASIYVK					Mascot
1332.61	1332.6672	0.0572	43	284	295	MDLPDAAEGLER			Oxidation (M)[1]		Mascot
1403.7529	1403.7327	-0.0202	-14	41	53	GADIIFVVDEGLR					Mascot
1453.7329	1453.7314	-0.0015	-1	76	88	NRHGANAQDMIVK					Mascot
1619.7847	1619.8069	0.0222	14	284	297	MDLPDAAEGLERFR					Mascot
2349.1721	2349.2131	0.041	17	185	206	VGSYHFTTLTPELGVVETADGR					Mascot
2374.3381	2374.1709	-0.1672	-70	150	171	YLELELRVLADVGLVGFPSVGK					Mascot

Calc. Mass	Obsrv. Mass	± da	± ppm	Start Seq.	End Seq.	Sequence	Ion Score	C. I. %	Modification	Rank	Result Type
2383.2075	2383.158	-0.0495	-21	239	259	IIVHVIDMAAVDGRDPVEDFR			Oxidation (M)[8]		Mascot

5	Uncharacterized protein OS=Alicyclobacillus acidoterrestris (strain ATCC 49025 / DSM 3922 / CIP 106132 / NCIMB 13137 / GD3B) GN=N007_11190 PE=4 SV=1	tr|T0CY60|T0CY60_ALIAG	28081.3	8.15	8	30	0		

Peptide Information

Calc. Mass	Obsrv. Mass	± da	± ppm	Start Seq.	End Seq.	Sequence	Ion Score	C. I. %	Modification	Rank	Result Type
829.4202	829.4385	0.0183	22	22	27	KFTDYR					Mascot
977.5567	977.489	-0.0677	-69	147	154	AFQAVKWK					Mascot
1332.5525	1332.6672	0.1147	86	183	193	FCAEAYEETGR			Carbamidomethyl (C)[2]		Mascot
1403.626	1403.7327	0.1067	76	182	193	KFCAEAYEETGR					Mascot
1795.8871	1795.9275	0.0404	22	6	21	TIFLVGAAHMTFEQCK					Mascot
2180.0991	2180.0432	-0.0559	-26	2	21	GAQKTIFLVGAAHMTFEQCK					Mascot
2343.1294	2343.2156	0.0862	37	1	21	MGAQKTIFLVGAAHMTFEQCK			Oxidation (M)[1,15]		Mascot
2384.156	2384.106	-0.05	-21	1	21	MGAQKTIFLVGAAHMTFEQCK			Carbamidomethyl (C)[20], Oxidation (M)[1]		Mascot
2488.2441	2488.3391	0.095	38	200	220	WILHESAMGNIEQFFKPTAPR			Oxidation (M)[8]		Mascot

6	Uncharacterized protein OS=Alicyclobacillus acidoterrestris (strain ATCC 49025 / DSM 3922 / CIP 106132 / NCIMB 13137 / GD3B) GN=N007_17180 PE=4 SV=1	tr|T0BKR4|T0BKR4_ALIAG	21303.2	9.32	5	29	0	9	32.953

Peptide Information

Calc. Mass	Obsrv. Mass	± da	± ppm	Start Seq.	End Seq.	Sequence	Ion Score	C. I. %	Modification	Rank	Result Type
851.4113	851.4625	0.0512	60	166	173	KCGAMDVK					Mascot
935.6036	935.5488	-0.0548	-59	50	58	GLILHAIAK					Mascot
935.6036	935.5488	-0.0548	-59	50	58	GLILHAIAK	9	32.953			Mascot
1978.0732	1977.9773	-0.0959	-48	174	191	IHGPHYAMFPPVKIVSGK					Mascot
2299.1899	2299.1731	-0.0168	-7	1	20	MLLGCIIVILEAAYMVWGSK			Carbamidomethyl (C)[5], Oxidation (M)[1,15]		Mascot
2357.2129	2357.2104	-0.0025	-1	28	49	DRVVELLGITGDELALDVGCGR			Carbamidomethyl (C)[20]		Mascot

7	Probable DNA-directed RNA polymerase subunit delta OS=Alicyclobacillus acidoterrestris (strain ATCC 49025 / DSM 3922 / CIP 106132 / NCIMB 13137 / GD3B) GN=rpoE PE=3 SV=1	tr|T0BXJ9|T0BXJ9_ALIAG	20430.3	3.81	6	27	0		

Peptide Information

Calc. Mass	Obsrv. Mass	± da	± ppm	Start Seq.	End Seq.	Sequence	Ion Score	C. I. %	Modification	Rank	Result Type

Calc. Mass	Obsrv. Mass	± da	± ppm	Start Seq.	End Seq.	Sequence	Ion Score	C. I. %	Modification	Rank	Result Type
807.4393	807.4531	0.0138	17	1	7	MAVTLTR			Oxidation (M)[1]		Mascot
1274.6774	1274.6624	-0.015	-12	36	45	DIMKEIQELR					Mascot
1519.7838	1519.7772	-0.0066	-4	69	81	FICIGQNVWGLNR					Mascot
2272.1531	2272.2429	0.0898	40	8	26	SDHEIQQMPLVELAYEIL K			Oxidation (M)[8]		Mascot
2310.1489	2310.1228	-0.0261	-11	69	87	FICIGQNVWGLNRWYPT DK					Mascot
2317.1782	2317.2366	0.0584	25	40	58	EIQELRNLTEEQFNDVIA R					Mascot
2317.1782	2317.2366	0.0584	25	40	58	EIQELRNLTEEQFNDVIA R					Mascot

8	Uncharacterized protein OS=Alicyclobacillus acidoterrestris (strain ATCC 49025 / DSM 3922 / CIP 106132 / NCIMB 13137 / GD3B) GN=N007_17530 PE=4 SV=1	tr|T0CNK3|T0CNK3_ALIAG	29048.3	8.79	8	26	0

Peptide Information

Calc. Mass	Obsrv. Mass	± da	± ppm	Start Seq.	End Seq.	Sequence	Ion Score	C. I. %	Modification	Rank	Result Type
807.4723	807.4531	-0.0192	-24	92	98	EAHPLLK					Mascot
935.5421	935.5488	0.0067	7	132	139	FISRVGTR					Mascot
935.5421	935.5488	0.0067	7	132	139	FISRVGTR					Mascot
977.5163	977.489	-0.0273	-28	64	71	DPISRAYR					Mascot
1146.6477	1146.5844	-0.0633	-55	41	50	LELNKSTVSR					Mascot
1161.5027	1161.6166	0.1139	98	140	149	CKAYCTSSGK			Carbamidomethyl (C)[1,5]		Mascot
1180.5593	1180.6525	0.0932	79	192	201	QGYALSQEER					Mascot
1403.8118	1403.7327	-0.0791	-56	92	103	EAHPLLKNLVNR					Mascot
1619.8541	1619.8069	-0.0472	-29	171	183	WTKNTITDPQLFR					Mascot

9	Uncharacterized protein OS=Alicyclobacillus acidoterrestris (strain ATCC 49025 / DSM 3922 / CIP 106132 / NCIMB 13137 / GD3B) GN=N007_19875 PE=4 SV=1	tr|T0CJN3|T0CJN3_ALIAG	32047.2	5.07	7	26	0

Peptide Information

Calc. Mass	Obsrv. Mass	± da	± ppm	Start Seq.	End Seq.	Sequence	Ion Score	C. I. %	Modification	Rank	Result Type
1559.7378	1559.8639	0.1261	81	213	225	FDGQTFIDVQFDK					Mascot
1580.9047	1580.8015	-0.1032	-65	245	258	LPLSVTNKSYLAFK					Mascot
1815.9164	1816.0203	0.1039	57	5	20	LTSYLNDVFAPYDGIK					Mascot
2289.0242	2289.2	0.1758	77	190	209	EATFENCSFHGVDFKSS DLR					Mascot
2318.1375	2318.2314	0.0939	41	1	20	MNEKLTSYLNDVFAPYD GIK					Mascot

Calc. Mass	Obsrv. Mass	± da	± ppm	Start Seq.	End Seq.	Sequence	Ion Score	C. I. %	Modification	Rank	Result Type
2334.1323	2334.2295	0.0972	42	1	20	MNEKLTSYLNDVFAPYD GIK			Oxidation (M)[1]		Mascot
2343.2302	2343.2156	-0.0146	-6	81	102	INLSATNLQESDFAGVTL HKGK					Mascot
2488.2217	2488.3391	0.1174	47	105	129	ASALRGSNFAGADLTGS FFAASDVR					Mascot

10 Uncharacterized protein OS=Alicyclobacillus acidoterrestris (strain ATCC 49025 / DSM 3922 / CIP 106132 / NCIMB 13137 / GD3B) GN=N007_15465 PE=4 SV=1 tr|T0CS71|T0CS71_ALIAG 116169.7 5.52 14 25 0

Peptide Information

Calc. Mass	Obsrv. Mass	± da	± ppm	Start Seq.	End Seq.	Sequence	Ion Score	C. I. %	Modification	Rank	Result Type
1106.6542	1106.554	-0.1002	-91	183	191	LNQVLRAHR					Mascot
1557.7908	1557.8602	0.0694	45	570	583	AKSLLGSYEEAAYR					Mascot
1557.7908	1557.8602	0.0694	45	570	583	AKSLLGSYEEAAYR					Mascot
1673.8817	1673.8256	-0.0561	-34	73	87	QESGELVTLERSLGR					Mascot
1687.8286	1687.9285	0.0999	59	353	368	AWESLPSDLSADAVAR					Mascot
2153.0146	2153.1362	0.1216	56	588	606	DTEWQVATNELQAAASQ YK					Mascot
2308.052	2308.2361	0.1841	80	891	911	MVVEAHQSSPLMANASD YMAR					Mascot
2318.2842	2318.2314	-0.0528	-23	46	68	GGLFGVARLAEGTTPVR PGAHVR					Mascot
2333.1831	2333.2141	0.031	13	809	829	SSLSALQESLIATEDEVE QKR					Mascot
2347.2778	2347.2166	-0.0612	-26	472	493	AMGMRAAILAALGVGLVF EWVR			Oxidation (M)[2]		Mascot
2363.2727	2363.2	-0.0727	-31	472	493	AMGMRAAILAALGVGLVF EWVR			Oxidation (M)[2,4]		Mascot
2396.282	2396.1023	-0.1797	-75	969	989	ERGVILPVILDDPLVNFDD TR					Mascot
2439.2263	2439.2056	-0.0207	-8	686	707	QNLDAPTAAQWAVEALQ ETLNR					Mascot
2502.2161	2502.374	0.1579	63	16	37	DWSVTFHSEGLHVLYGR NEAGK					Mascot
2507.2747	2507.2734	-0.0013	-1	785	808	ALQALVVQCSGETAAFQ VVQMAAR			Oxidation (M)[21]		Mascot
2742.396	2742.5515	0.1555	57	258	281	MAALGPVPEGTYEIWVQ VPTWLER					Mascot

Gel Idx/Pos	226/J2	Instr./Gel Origin	BA2151/full sequence test 20150515	Process Status	Analysis Succeeded
Plate [#] Name	[3] full sequence test 20150515	Instrument Sample Name		Spectra	11

Rank	Protein Name	Species	Accession No.	Protein MW	Protein PI	Pep. Count	Protein Score	Protein Score C. I. %	Total Ion Score	Total Ion C. I. %
1	Elongation factor Tu OS=Alicyclobacillus acidoterrestris (strain ATCC 49025 / DSM 3922 / CIP 106132 / NCIMB 13137 / GD3B) GN=tuf PE=3 SV=1		tr|T0D867|T0D867_ALIAG	43353	5.09	13	409	100	350	100

Peptide Information

Calc. Mass	Obsrv. Mass	± da	± ppm	Start Seq.	End Seq.	Sequence	Ion Score	C. I. %	Modification	Rank	Result Type
1124.5769	1124.6039	0.027	24	255	264	TVATGIEMFR					Mascot
1140.5718	1140.5946	0.0228	20	255	264	TVATGIEMFR			Oxidation (M)[8]		Mascot
1252.6719	1252.7036	0.0317	25	255	265	TVATGIEMFRK					Mascot
1268.6667	1268.6921	0.0254	20	255	265	TVATGIEMFRK			Oxidation (M)[8]		Mascot
1667.8235	1667.8524	0.0289	17	239	253	VGDEVEIVGLHEESR					Mascot
1667.8235	1667.8524	0.0289	17	239	253	VGDEVEIVGLHEESR	56	99.999			Mascot
1687.9014	1687.9305	0.0291	17	266	281	LLDFAEAGDNIGALLR	101	100			Mascot
1795.9185	1795.9493	0.0308	17	239	254	VGDEVEIVGLHEESRK	78	100			Mascot
1795.9563	1795.9493	-0.007	-4	9	25	SKPHVNIGTIGHVDHGK					Mascot
1815.9963	1816.0256	0.0293	16	265	281	KLLDFAEAGDNIGALLR					Mascot
1815.9963	1816.0256	0.0293	16	265	281	KLLDFAEAGDNIGALLR	51	99.996			Mascot
1976.9556	1976.9904	0.0348	18	321	335	HTPFFNGYRPQFYFR					Mascot
1976.9556	1976.9904	0.0348	18	321	335	HTPFFNGYRPQFYFR	11	59.434			Mascot
2094.0825	2094.1047	0.0222	11	235	253	GQLKVGDEVEIVGLHEESR					Mascot
2153.0947	2153.1328	0.0381	18	206	224	DTSKPFLMPVEDVFTITGR					Mascot
2153.0947	2153.1328	0.0381	18	206	224	DTSKPFLMPVEDVFTITGR	13	71.545			Mascot
2166.9604	2167.1184	0.158	73	138	155	CDMVDDEELLELVEMEVR					Mascot
2169.0896	2169.125	0.0354	16	206	224	DTSKPFLMPVEDVFTITGR			Oxidation (M)[8]		Mascot
2169.0896	2169.125	0.0354	16	206	224	DTSKPFLMPVEDVFTITGR			Oxidation (M)[8]		Mascot
2182.9553	2183.0876	0.1323	61	138	155	CDMVDDEELLELVEMEVR			Oxidation (M)[3]		Mascot
2743.4236	2743.4785	0.0549	20	291	315	GQVVCKPGSINPHTQFAAEVYVLTK			Carbamidomethyl (C)[5]		Mascot
2743.4236	2743.4785	0.0549	20	291	315	GQVVCKPGSINPHTQFAAEVYVLTK	30	99.473	Carbamidomethyl (C)[5]		Mascot
3271.6528	3271.7444	0.0916	28	291	320	GQVVCKPGSINPHTQFAAEVYVLTKEEGGR			Carbamidomethyl (C)[5]		Mascot
3271.6528	3271.7444	0.0916	28	291	320	GQVVCKPGSINPHTQFA	10	40.958	Carbamidomethyl (C)[5]		Mascot

AEVYVLTKEEGGR

2	Uncharacterized protein OS=Alicyclobacillus acidoterrestris (strain ATCC 49025 / DSM 3922 / CIP 106132 / NCIMB 13137 / GD3B) GN=N007_15375 PE=4 SV=1	tr|T0BP15|T0BP15_ALIAG	44556.7	10.94	11	36	0

Peptide Information

Calc. Mass	Obsrv. Mass	± da	± ppm	Start Seq.	End Seq.	Sequence	Ion Score	C. I. %	Modification	Rank	Result Type
1139.5917	1139.5712	-0.0205	-18	370	381	KSGGVTGSPGHR					Mascot
1140.6637	1140.5946	-0.0691	-61	237	245	VDFRVVLHR					Mascot
1403.739	1403.7083	-0.0307	-22	181	192	LGPNQYRVSVDR					Mascot
1681.8578	1681.8738	0.016	10	1	17	MAGSTGYIGVATTALPR			Oxidation (M)[1]		Mascot
1795.9272	1795.9493	0.0221	12	202	215	MTISEPQLRHWIER					Mascot
1795.9272	1795.9493	0.0221	12	202	215	MTISEPQLRHWIER					Mascot
1809.9528	1809.9635	0.0107	6	1	18	MAGSTGYIGVATTALPRK			Oxidation (M)[1]		Mascot
1837.8749	1837.9603	0.0854	46	133	147	SNDMLEFTPETERLR					Mascot
1858.9506	1858.9487	-0.0019	-1	264	281	DGVVTNIIAGGERTDVDK					Mascot
1915.9219	1915.9707	0.0488	25	313	330	HPTVGLVGFDMAVEENGK			Oxidation (M)[11]		Mascot
1961.0504	1960.9729	-0.0775	-40	64	80	AWVPVNPAEPRGNWVLR					Mascot
2072.0229	2072.0977	0.0748	36	312	330	RHPTVGLVGFDMAVEENGK			Oxidation (M)[12]		Mascot

3	Uncharacterized protein OS=Alicyclobacillus acidoterrestris (strain ATCC 49025 / DSM 3922 / CIP 106132 / NCIMB 13137 / GD3B) GN=N007_10240 PE=4 SV=1	tr|T0BUW9|T0BUW9_ALIAG	42564	9.68	10	31	0

Peptide Information

Calc. Mass	Obsrv. Mass	± da	± ppm	Start Seq.	End Seq.	Sequence	Ion Score	C. I. %	Modification	Rank	Result Type
1495.6846	1495.7549	0.0703	47	246	259	GGTGEWEMTAITAR			Oxidation (M)[8]		Mascot
1691.8719	1691.8805	0.0086	5	174	187	NMLLIQPLCDGRYR					Mascot
1829.8925	1830.0437	0.1512	83	70	85	IAPMSGNVLYDAMYLR			Oxidation (M)[4]		Mascot
1839.8286	1839.973	0.1444	78	32	48	DVGMEMMITTPANVNGK			Oxidation (M)[4,6]		Mascot
1869.8912	1869.9282	0.037	20	246	262	GGTGEWEMTAITARYAR					Mascot
1959.88	1959.9742	0.0942	48	333	348	QDKAMYVYDCNSRPGR			Carbamidomethyl (C)[10]		Mascot
2000.1111	1999.9662	-0.1449	-72	1	18	MLFVLTGRGPTALQNLPR			Oxidation (M)[1]		Mascot
2175.9951	2176.1123	0.1172	54	50	69	GVCHGWTMGESAQTISWTPK					Mascot
2186.0984	2186.1204	0.022	10	70	88	IAPMSGNVLYDAMYLRDLK			Oxidation (M)[4]		Mascot

3270.5989	3270.655	0.0561	17	20	48	ILTFQLLQQACRDVGME MMITTPANVNGK			Oxidation (M)[16,18,19]		Mascot

4	Uncharacterized protein OS=Alicyclobacillus acidoterrestris (strain ATCC 49025 / DSM 3922 / CIP 106132 / NCIMB 13137 / GD3B) GN=N007_05115 PE=4 SV=1	tr|T0C3R8|T0C3R 8_ALIAG	9102.8	9.05	4	28	0	

Peptide Information

Calc. Mass	Obsrv. Mass	± da	± ppm	Start Seq.	End Seq.	Sequence	Ion Score	C. I. %	Modification	Rank	Result Type
1731.8953	1731.8701	-0.0252	-15	28	42	HFLVDLAEDAEVFVK					Mascot
1931.0273	1931.0344	0.0071	4	26	42	AKHFLVDLAEDAEVFVK					Mascot
1998.9979	1998.9525	-0.0454	-23	57	75	QFTGLDSTSTQSAVETAK K					Mascot
3271.6328	3271.7444	0.1116	34	43	74	QGFALVSEGEAAIKQFTG LDSTSTQSAVETAK					Mascot
3271.6328	3271.7444	0.1116	34	43	74	QGFALVSEGEAAIKQFTG LDSTSTQSAVETAK					Mascot

5	Uncharacterized protein OS=Alicyclobacillus acidoterrestris (strain ATCC 49025 / DSM 3922 / CIP 106132 / NCIMB 13137 / GD3B) GN=N007_18315 PE=4 SV=1	tr|T0CJX8|T0CJX8 _ALIAG	15049.7	8.93	6	28	0	

Peptide Information

Calc. Mass	Obsrv. Mass	± da	± ppm	Start Seq.	End Seq.	Sequence	Ion Score	C. I. %	Modification	Rank	Result Type
1268.6522	1268.6921	0.0399	31	97	107	LGYIEQGVFDK					Mascot
1674.819	1674.8149	-0.0041	-2	22	35	RSMTLCENIYSLTK			Oxidation (M)[3]		Mascot
1731.8405	1731.8701	0.0296	17	22	35	RSMTLCENIYSLTK			Carbamidomethyl (C)[6], Oxidation (M)[3]		Mascot
1815.8694	1816.0256	0.1562	86	73	88	QMAFYSNALGSLQETR					Mascot
1815.8694	1816.0256	0.1562	86	73	88	QMAFYSNALGSLQETR					Mascot
1858.9559	1858.9487	-0.0072	-4	36	51	QFPKHELYALGSQGQR					Mascot
1916.0344	1915.9707	-0.0637	-33	108	124	LDAEAQELVRMLVGMIK					Mascot
1959.9594	1959.9742	0.0148	8	72	88	KQMAFYSNALGSLQETR			Oxidation (M)[3]		Mascot

6	Succinate dehydrogenase flavoprotein subunit OS=Alicyclobacillus acidoterrestris (strain ATCC 49025 / DSM 3922 / CIP 106132 / NCIMB 13137 / GD3B) GN=sdhA PE=4 SV=1	tr|T0BYA7|T0BYA 7_ALIAG	65656.8	5.81	12	28	0	

Peptide Information

Calc. Mass	Obsrv. Mass	± da	± ppm	Start Seq.	End Seq.	Sequence	Ion Score	C. I. %	Modification	Rank	Result Type
854.4011	854.3743	-0.0268	-31	99	105	MGVMFNR					Mascot
880.4047	880.3344	-0.0703	-80	541	547	DDENFLK					Mascot

1701.8218	1701.9532	0.1314	77	416	430	SVDSLPESLFESYTK					Mascot
1709.835	1709.8894	0.0544	32	189	205	ADAVVMCTGGNGLIFGK			Carbamidomethyl (C)[7]		Mascot
1725.83	1725.8828	0.0528	31	189	205	ADAVVMCTGGNGLIFGK			Carbamidomethyl (C)[7], Oxidation (M)[6]		Mascot
1809.9939	1809.9635	-0.0304	-17	510	525	NMLHMARVITLGALNR					Mascot
1829.9167	1830.0437	0.127	69	416	431	SVDSLPESLFESYTKK					Mascot
1934.9575	1934.9849	0.0274	14	173	188	GIVAQDLRSMEIMHFR			Oxidation (M)[10,13]		Mascot
1999.9762	1999.9662	-0.01	-5	81	98	QVLGMCEAAPAIIHLMDR			Oxidation (M)[5,16]		Mascot
2071.9753	2072.0977	0.1224	59	227	245	YANGEMIQVHPTAIPGDD K			Oxidation (M)[6]		Mascot
2185.9858	2186.1204	0.1346	62	155	172	YEGWDFLGAVIDDEQICR			Carbamidomethyl (C)[17]		Mascot
2222.0229	2222.2058	0.1829	82	489	506	IHMADTSRWENQMAQFT R					Mascot
2743.2305	2743.4785	0.248	90	432	453	YQDDFESILKMEGDENP YQLHR			Oxidation (M)[11]		Mascot
2743.2305	2743.4785	0.248	90	432	453	YQDDFESILKMEGDENP YQLHR			Oxidation (M)[11]		Mascot

7 Uncharacterized protein OS=Alicyclobacillus acidoterrestris (strain ATCC 49025 / DSM 3922 / CIP 106132 / NCIMB 13137 / GD3B) GN=N007_06050 PE=4 SV=1 — tr|T0DCY7|T0DCY7_ALIAG — 23656.1 — 5.83 — 6 — 25 — 0 — 2 — 0

Peptide Information

Calc. Mass	Obsrv. Mass	± da	± ppm	Start Seq.	End Seq.	Sequence	Ion Score	C. I. %	Modification	Rank	Result Type
1389.7406	1389.6915	-0.0491	-35	119	130	VLTISCDQEILR					Mascot
1389.7406	1389.6915	-0.0491	-35	119	130	VLTISCDQEILR	2	0			Mascot
1403.6948	1403.7083	0.0135	10	192	205	LLQMPAGSSESGAR					Mascot
1731.9137	1731.8701	-0.0436	-25	14	29	HIHLSAQGAEIELSAR					Mascot
1740.7932	1740.8219	0.0287	16	53	68	MGDGAFVVEAVPETMR			Oxidation (M)[1,15]		Mascot
1896.8943	1896.9596	0.0653	34	53	69	MGDGAFVVEAVPETMRR			Oxidation (M)[1,15]		Mascot
2065.0271	2065.0156	-0.0115	-6	174	191	VGDEVNLECDIIAKYVER					Mascot

8 Uncharacterized protein OS=Alicyclobacillus acidoterrestris (strain ATCC 49025 / DSM 3922 / CIP 106132 / NCIMB 13137 / GD3B) GN=N007_15300 PE=4 SV=1 — tr|T0BNR2|T0BNR2_ALIAG — 57489.2 — 5.25 — 10 — 25 — 0

Peptide Information

Calc. Mass	Obsrv. Mass	± da	± ppm	Start Seq.	End Seq.	Sequence	Ion Score	C. I. %	Modification	Rank	Result Type
854.3825	854.3743	-0.0082	-10	255	261	YCANVER					Mascot
1138.5422	1138.5809	0.0387	34	255	263	YCANVERAR			Carbamidomethyl (C)[2]		Mascot
1140.6848	1140.5946	-0.0902	-79	507	517	AGAILDVLRGR					Mascot

Calc. Mass	Obsrv. Mass	± da	± ppm	Start Seq.	End Seq.	Sequence	Ion Score	C. I. %	Modification	Rank	Result Type
1701.9204	1701.9532	0.0328	19	436	449	IRAMEIIDELETLR					Mascot
1815.9495	1816.0256	0.0761	42	325	342	ALNGSAEMKPIAGTSRG R	1	0			Mascot
1815.9501	1816.0256	0.0755	42	282	297	IEGALHPYAAYDALRR					Mascot
1930.9514	1931.0344	0.083	43	380	396	VCLSGSVHVPDFMVVER			Carbamidomethyl (C)[2]		Mascot
1958.0707	1957.9497	-0.121	-62	480	498	GTQYVQVGAGIVLDSVP VR					Mascot
1960.9103	1960.9729	0.0626	32	358	374	ADEKESAEHVMLVDLCR			Oxidation (M)[11]		Mascot
2001.9369	2001.9456	0.0087	4	358	374	ADEKESAEHVMLVDLCR			Carbamidomethyl (C)[16]		Mascot
2168.1321	2168.1436	0.0115	5	104	120	YFRPVDPMAFLEELRIR			Oxidation (M)[8]		Mascot

9	Uncharacterized protein OS=Alicyclobacillus acidoterrestris (strain ATCC 49025 / DSM 3922 / CIP 106132 / NCIMB 13137 / GD3B) GN=N007_20080 PE=4 SV=1	tr|T0C8Y8|T0C8Y8_ALIAG	12226.3	9.37	4	23	0

Peptide Information

Calc. Mass	Obsrv. Mass	± da	± ppm	Start Seq.	End Seq.	Sequence	Ion Score	C. I. %	Modification	Rank	Result Type
1139.6168	1139.5712	-0.0456	-40	54	63	DPTLDIPRGR					Mascot
1477.7039	1477.7517	0.0478	32	76	88	VWNQVASMCVNAR					Mascot
1935.0852	1934.9849	-0.1003	-52	89	105	LFHTLFRIGTSPGLFTK					Mascot
3271.5471	3271.7444	0.1973	60	1	30	MAVDEGNSCEQAAEQVA PPIGPLSSRTVWR			Carbamidomethyl (C)[9], Oxidation (M)[1]		Mascot
3271.5471	3271.7444	0.1973	60	1	30	MAVDEGNSCEQAAEQVA PPIGPLSSRTVWR			Carbamidomethyl (C)[9], Oxidation (M)[1]		Mascot

10	Thymidine kinase OS=Alicyclobacillus acidoterrestris (strain ATCC 49025 / DSM 3922 / CIP 106132 / NCIMB 13137 / GD3B) GN=tdk PE=3 SV=1	tr|T0CAI2|T0CAI2_ALIAG	22183.3	6.08	6	23	0

Peptide Information

Calc. Mass	Obsrv. Mass	± da	± ppm	Start Seq.	End Seq.	Sequence	Ion Score	C. I. %	Modification	Rank	Result Type
1233.6838	1233.655	-0.0288	-23	33	43	VTLFTPAIDTR					Mascot
1477.7257	1477.7517	0.026	18	4	15	LYYRFGQMNASK					Mascot
1819.9874	1819.9552	-0.0322	-18	99	114	VVDELNTPVIMYGLLK			Oxidation (M)[11]		Mascot
1829.9392	1830.0437	0.1045	57	16	31	SIQLLTVAHNYEEQGK					Mascot
1958.0342	1957.9497	-0.0845	-43	16	32	SIQLLTVAHNYEEQGKK					Mascot
2167.0303	2167.1184	0.0881	41	158	177	DGVPVYSGEQIEVGGND LYR					Mascot

Gel Idx/Pos	227/J3	Instr./Gel Origin	BA2151/full sequence test 20150515	Process Status	Analysis Succeeded
Plate [#] Name	[3] full sequence test 20150515	Instrument Sample Name		Spectra	11

Rank	Protein Name	Species	Accession No.	Protein MW	Protein PI	Pep. Count	Protein Score	Protein Score C. I. %	Total Ion Score	Total Ion C. I. %
1	Alkyl hydroperoxide reductase subunit C OS=Alicyclobacillus acidoterrestris (strain ATCC 49025 / DSM 3922 / CIP 106132 / NCIMB 13137 / GD3B) GN=N007_20450 PE=4 SV=1		tr|T0CIG8|T0CIG8_ALIAG	20796.5	4.93	11	367	100	297	100

Peptide Information

Calc. Mass	Obsrv. Mass	± da	± ppm	Start Seq.	End Seq.	Sequence	Ion Score	C. I. %	Modification	Rank	Result Type
860.4724	860.4766	0.0042	5	144	151	DASILVDK					Mascot
1101.6514	1101.67	0.0186	17	144	153	DASILVDKIK					Mascot
1108.5205	1108.5576	0.0371	33	160	169	NHPGEVCPAK			Carbamidomethyl (C)[7]		Mascot
1201.5597	1201.5834	0.0237	20	81	90	AWHDTSDTIR					Mascot
1218.7092	1218.7352	0.026	21	2	12	SLIGTEVKPFK					Mascot
1605.7755	1605.8	0.0245	15	107	120	NFDVLIEEEGVADR					Mascot
1605.7755	1605.8	0.0245	15	107	120	NFDVLIEEEGVADR	79	100			Mascot
1703.8785	1703.9009	0.0224	13	92	106	ITYVMIGDPTHTISR					Mascot
1719.8735	1719.8988	0.0253	15	92	106	ITYVMIGDPTHTISR			Oxidation (M)[5]		Mascot
1719.8735	1719.8988	0.0253	15	92	106	ITYVMIGDPTHTISR			Oxidation (M)[5]		Mascot
1831.9735	1831.9968	0.0233	13	91	106	KITYVMIGDPTHTISR					Mascot
1847.9685	1847.9895	0.021	11	91	106	KITYVMIGDPTHTISR			Oxidation (M)[6]		Mascot
1847.9685	1847.9895	0.021	11	91	106	KITYVMIGDPTHTISR			Oxidation (M)[6]		Mascot
1992.9662	1992.9946	0.0284	14	63	80	SLGVEVYSVSTDSHFTHK					Mascot
1992.9662	1992.9946	0.0284	14	63	80	SLGVEVYSVSTDSHFTHK	113	100			Mascot
2067.9771	2068.011	0.0339	16	13	30	AQAYHNGEFIEVTEANFK					Mascot
2067.9771	2068.011	0.0339	16	13	30	AQAYHNGEFIEVTEANFK	80	100			Mascot
2326.2402	2326.2791	0.0389	17	121	143	GTFIIDPDGVIQAIEINAGG IGR					Mascot
2326.2402	2326.2791	0.0389	17	121	143	GTFIIDPDGVIQAIEINAGG IGR	25	98.304			Mascot

Rank	Protein Name	Species	Accession No.	Protein MW	Protein PI	Pep. Count	Protein Score	Protein Score C. I. %	Total Ion Score	Total Ion C. I. %
2	Heat shock protein Hsp20 OS=Alicyclobacillus acidoterrestris (strain ATCC 49025 / DSM 3922 / CIP 106132 / NCIMB 13137 / GD3B) GN=N007_11060 PE=3 SV=1		tr|T0D430|T0D430_ALIAG	17223.5	6.06	8	43	79.441		

Peptide Information

Calc. Mass	Obsrv. Mass	± da	± ppm	Start Seq.	End Seq.	Sequence	Ion Score	C. I. %	Modification	Rank	Result Type

Calc. Mass	Obsrv. Mass	± da	± ppm	Start Seq.	End Seq.	Sequence	Ion Score	C. I. %	Modification	Rank	Result Type
1185.543	1185.5808	0.0378	32	90	98	DENVHRMER					Mascot
1201.5378	1201.5834	0.0456	38	90	98	DENVHRMER			Oxidation (M)[7]		Mascot
1345.6318	1345.6636	0.0318	24	137	147	QMGRQIDVDFH					Mascot
1361.6267	1361.6664	0.0397	29	137	147	QMGRQIDVDFH			Oxidation (M)[2]		Mascot
1449.7195	1449.7798	0.0603	42	2	13	PLVPYDPFNMTR					Mascot
1580.76	1580.8026	0.0426	27	1	13	MPLVPYDPFNMTR					Mascot
1605.8207	1605.8	-0.0207	-13	2	14	PLVPYDPFNMTRR					Mascot
1605.8207	1605.8	-0.0207	-13	2	14	PLVPYDPFNMTRR					Mascot
1832.9753	1833.0048	0.0295	16	106	123	TVPLPTAVNDTGAKASYK					Mascot
1998.0753	1998.0568	-0.0185	-9	47	64	ETQNEVIVTAEIPGLEKK					Mascot
2326.1899	2326.2791	0.0892	38	65	84	EDVNITVHDNHLHLSGKIER					Mascot
2326.1899	2326.2791	0.0892	38	65	84	EDVNITVHDNHLHLSGKIER					Mascot

3	Transcriptional regulator OS=Alicyclobacillus acidoterrestris (strain ATCC 49025 / DSM 3922 / CIP 106132 / NCIMB 13137 / GD3B) GN=N007_20770 PE=4 SV=1	tr|T0CGM5|T0CGM5_ALIAG	12822.8	8.09	6	32	0	

Peptide Information

Calc. Mass	Obsrv. Mass	± da	± ppm	Start Seq.	End Seq.	Sequence	Ion Score	C. I. %	Modification	Rank	Result Type
1070.5088	1070.5183	0.0095	9	93	100	FWSTNMLR			Oxidation (M)[6]		Mascot
1205.6161	1205.58	-0.0361	-30	10	20	DVYVAVADPTR					Mascot
1245.5845	1245.5858	0.0013	1	101	110	LNQLIEEEEE					Mascot
1361.7173	1361.6664	-0.0509	-37	10	21	DVYVAVADPTRR					Mascot
1587.933	1587.798	-0.135	-85	52	65	HLTILKEAGLVTHR					Mascot
2111.0186	2110.9868	-0.0318	-15	2	20	MTATNQSRDVYVAVADPTR			Oxidation (M)[1]		Mascot

4	Dephospho-CoA kinase OS=Alicyclobacillus acidoterrestris (strain ATCC 49025 / DSM 3922 / CIP 106132 / NCIMB 13137 / GD3B) GN=coaE PE=3 SV=1	tr|T0C4I7|T0C4I7_ALIAG	22742.8	5.83	7	31	0	

Peptide Information

Calc. Mass	Obsrv. Mass	± da	± ppm	Start Seq.	End Seq.	Sequence	Ion Score	C. I. %	Modification	Rank	Result Type
1070.6106	1070.5183	-0.0923	-86	71	79	IVFTNAHLR					Mascot
1217.5869	1217.5829	-0.004	-3	147	157	DGLNESEARAR					Mascot
1233.6873	1233.5724	-0.1149	-93	1	13	MIVGLTGGIATGK			Oxidation (M)[1]		Mascot
1357.7257	1357.675	-0.0507	-37	156	167	ARISAQMPIDQK					Mascot

Calc. Mass	Obsrv. Mass	± da	± ppm	Start Seq.	End Seq.	Sequence	Ion Score	C. I. %	Modification	Rank	Result Type
1357.7257	1357.675	-0.0507	-37	156	167	ARISAQMPIDQK					Mascot
1719.8813	1719.8988	0.0175	10	22	36	ELGAYVVDADVWARR					Mascot
1719.8813	1719.8988	0.0175	10	22	36	ELGAYVVDADVWARR					Mascot
2050.0564	2050.0527	-0.0037	-2	168	185	RALADYVIVNDSTLENTR					Mascot
2107.125	2106.9712	-0.1538	-73	1	21	MIVGLTGGIATGKSTVSD MLR					Mascot
2123.1199	2122.9788	-0.1411	-66	1	21	MIVGLTGGIATGKSTVSD MLR			Oxidation (M)[1]		Mascot

5	Uncharacterized protein OS=Alicyclobacillus acidoterrestris (strain ATCC 49025 / DSM 3922 / CIP 106132 / NCIMB 13137 / GD3B) GN=N007_07145 PE=4 SV=1	tr|T0BQG7|T0BQG 7_ALIAG	28516.4	4.84	7	26	0

Peptide Information

Calc. Mass	Obsrv. Mass	± da	± ppm	Start Seq.	End Seq.	Sequence	Ion Score	C. I. %	Modification	Rank	Result Type
1108.611	1108.5576	-0.0534	-48	239	247	LEELHQALR					Mascot
1629.7836	1629.8022	0.0186	11	239	251	LEELHQALRDCMR			Oxidation (M)[12]		Mascot
1703.8361	1703.9009	0.0648	38	184	198	VHPDDYAAARQAHPR					Mascot
1833.0303	1833.0048	-0.0255	-14	133	149	LHALQPIVEELAMAAVK					Mascot
1977.1201	1977.0111	-0.109	-55	133	150	LHALQPIVEELAMAAVKK			Oxidation (M)[13]		Mascot
1984.0458	1984.1396	0.0938	47	112	129	QKEQAALTQLAEQIEAGR					Mascot
2068.0669	2068.011	-0.0559	-27	65	84	AEVEAQAAIAAAQVEAQE IR					Mascot
2068.0669	2068.011	-0.0559	-27	65	84	AEVEAQAAIAAAQVEAQE IR					Mascot

6	Uncharacterized protein OS=Alicyclobacillus acidoterrestris (strain ATCC 49025 / DSM 3922 / CIP 106132 / NCIMB 13137 / GD3B) GN=N007_16045 PE=4 SV=1	tr|T0BNP8|T0BNP 8_ALIAG	28683.1	6.11	7	24	0

Peptide Information

Calc. Mass	Obsrv. Mass	± da	± ppm	Start Seq.	End Seq.	Sequence	Ion Score	C. I. %	Modification	Rank	Result Type
860.4373	860.4766	0.0393	46	17	23	HQTFTAR					Mascot
1357.7838	1357.675	-0.1088	-80	58	69	LLQPGDVILSFR					Mascot
1357.7838	1357.675	-0.1088	-80	58	69	LLQPGDVILSFR					Mascot
1679.8721	1679.9325	0.0604	36	82	97	VLGAMMVPSGYATRAR					Mascot
1831.9048	1831.9968	0.092	50	138	152	MVDGPFVYIDKNYVR			Oxidation (M)[1]		Mascot
1997.9606	1998.0568	0.0962	48	1	16	MQTMMDLRDPLFQSLR			Oxidation (M)[1]		Mascot
2090.1692	2089.9968	-0.1724	-82	117	137	IPQAFKPGLHAALAAAGI GMR					Mascot
2106.1641	2105.9661	-0.198	-94	117	137	IPQAFKPGLHAALAAAGI GMR			Oxidation (M)[20]		Mascot

Calc. Mass	Obsrv. Mass	± da	± ppm	Start Seq.	End Seq.	Sequence	Ion Score	C. I. %	Modification	Rank	Result Type
2341.1777	2341.2922	0.1145	49	242	263	ELPVDLVDVGAAVLDAP MVDMR			Oxidation (M)[18]		Mascot

7	Heat shock protein Hsp20 OS=Alicyclobacillus acidoterrestris (strain ATCC 49025 / DSM 3922 / CIP 106132 / NCIMB 13137 / GD3B) GN=N007_14950 PE=3 SV=1	tr|T0BEM1|T0BEM 1_ALIAG	17185.6	5.85	5	23	0

Peptide Information

Calc. Mass	Obsrv. Mass	± da	± ppm	Start Seq.	End Seq.	Sequence	Ion Score	C. I. %	Modification	Rank	Result Type
1185.543	1185.5808	0.0378	32	90	98	DENVHRMER					Mascot
1201.5378	1201.5834	0.0456	38	90	98	DENVHRMER			Oxidation (M)[7]		Mascot
1205.5521	1205.58	0.0279	23	31	40	FEGHFANMPR					Mascot
1861.9655	1861.9282	-0.0373	-20	106	123	TVPLPTAVDDTGAKASYR					Mascot
1974.8981	1974.9742	0.0761	39	15	30	DFNSFPSFPRLFDDDR					Mascot
2326.1899	2326.2791	0.0892	38	65	84	EDVNITVHDNHLHLSGKI ER					Mascot
2326.1899	2326.2791	0.0892	38	65	84	EDVNITVHDNHLHLSGKI ER					Mascot

8	Uncharacterized protein OS=Alicyclobacillus acidoterrestris (strain ATCC 49025 / DSM 3922 / CIP 106132 / NCIMB 13137 / GD3B) GN=N007_21125 PE=4 SV=1	tr|T0CDS7|T0CDS 7_ALIAG	17761.9	4.99	5	23	0

Peptide Information

Calc. Mass	Obsrv. Mass	± da	± ppm	Start Seq.	End Seq.	Sequence	Ion Score	C. I. %	Modification	Rank	Result Type
870.5771	870.5514	-0.0257	-30	93	99	QKLLQLK					Mascot
1651.7778	1651.8684	0.0906	55	12	25	NESEVICRCLDSVK			Carbamidomethyl (C)[7]		Mascot
1703.77	1703.9009	0.1309	77	53	66	VFDFEWVDDFASAR					Mascot
2137.0813	2136.8914	-0.1899	-89	2	19	INISLCMIVKNESEVICR			Carbamidomethyl (C)[6], Oxidation (M)[7]		Mascot
2341.1382	2341.2922	0.154	66	1	19	MINISLCMIVKNESEVICR			Carbamidomethyl (C)[7,18], Oxidation (M)[1,8]		Mascot

9	Uncharacterized protein OS=Alicyclobacillus acidoterrestris (strain ATCC 49025 / DSM 3922 / CIP 106132 / NCIMB 13137 / GD3B) GN=N007_07030 PE=4 SV=1	tr|T0C0U9|T0C0U 9_ALIAG	17080	4.53	5	23	0

Peptide Information

Calc. Mass	Obsrv. Mass	± da	± ppm	Start Seq.	End Seq.	Sequence	Ion Score	C. I. %	Modification	Rank	Result Type
1070.6205	1070.5183	-0.1022	-95	50	59	GTVTPVIDLR					Mascot
1123.5234	1123.621	0.0976	87	2	10	AELQCVTMR			Carbamidomethyl (C)[5], Oxidation (M)[8]		Mascot
2051.0227	2051.0212	-0.0015	-1	11	28	IGDELYGARVEQVMSVE					Mascot

Calc. Mass	Obsrv. Mass	± da	± ppm	Start Seq.	End Seq.	Sequence	Ion Score	C. I. %	Modification	Rank	Result Type
						R					
2082.0107	2082.0295	0.0188	9	2	19	AELQCVTMRIGDELYGA R			Carbamidomethyl (C)[5]		Mascot
2092.1396	2091.9807	-0.1589	-76	139	156	ILSDVEVAQLHEVEKQVR					Mascot

10	Uncharacterized protein OS=Alicyclobacillus acidoterrestris (strain ATCC 49025 / DSM 3922 / CIP 106132 / NCIMB 13137 / GD3B) GN=N007_15745 PE=4 SV=1	tr|T0BNT9|T0BNT9_ALIAG	3630.7	5.8	3	22	0

Peptide Information

Calc. Mass	Obsrv. Mass	± da	± ppm	Start Seq.	End Seq.	Sequence	Ion Score	C. I. %	Modification	Rank	Result Type
1340.6191	1340.6818	0.0627	47	24	35	VYAYMYKSAGTA			Oxidation (M)[5]		Mascot
1830.8917	1830.9949	0.1032	56	17	30	TICTYVRVYAYMYK			Carbamidomethyl (C)[3]		Mascot
2326.1167	2326.2791	0.1624	70	1	23	MDVDDAGQAGGAAALKT ICTYVR					Mascot
2326.1167	2326.2791	0.1624	70	1	23	MDVDDAGQAGGAAALKT ICTYVR					Mascot

Gel Idx/Pos	228/J4	Instr./Gel Origin	BA2151/full sequence test 20150515	Process Status	Analysis Succeeded
Plate [#] Name	[3] full sequence test 20150515	Instrument Sample Name		Spectra	11

Rank	Protein Name	Species	Accession No.	Protein MW	Protein PI	Pep. Count	Protein Score	Protein Score C. I. %	Total Ion Score	Total Ion C. I. %
1	Elongation factor Tu OS=Alicyclobacillus acidoterrestris (strain ATCC 49025 / DSM 3922 / CIP 106132 / NCIMB 13137 / GD3B) GN=tuf PE=3 SV=1		tr|T0D867|T0D867_ALIAG	43353	5.09	15	596	100	523	100

Peptide Information

Calc. Mass	Obsrv. Mass	± da	± ppm	Start Seq.	End Seq.	Sequence	Ion Score	C. I. %	Modification	Rank	Result Type
1124.5769	1124.5863	0.0094	8	255	264	TVATGIEMFR					Mascot
1130.678	1130.6949	0.0169	15	384	395	TVGAGVVSSIIK					Mascot
1140.5718	1140.5774	0.0056	5	255	264	TVATGIEMFR			Oxidation (M)[8]		Mascot
1252.6719	1252.6846	0.0127	10	255	265	TVATGIEMFRK					Mascot
1268.6667	1268.6788	0.0121	10	255	265	TVATGIEMFRK			Oxidation (M)[8]		Mascot
1364.6328	1364.6578	0.025	18	47	57	YDEIDKAPEER					Mascot
1667.8235	1667.8345	0.011	7	239	253	VGDEVEIVGLHEESR					Mascot
1667.8235	1667.8345	0.011	7	239	253	VGDEVEIVGLHEESR	86	100			Mascot
1687.9014	1687.9138	0.0124	7	266	281	LLDFAEAGDNIGALLR	116	100			Mascot
1711.7646	1711.8608	0.0962	56	76	90	HYAHVDCPGHADYVK					Mascot
1795.9185	1795.9319	0.0134	7	239	254	VGDEVEIVGLHEESRK					Mascot
1795.9185	1795.9319	0.0134	7	239	254	VGDEVEIVGLHEESRK	59	100			Mascot
1815.9963	1816.0088	0.0125	7	265	281	KLLDFAEAGDNIGALLR					Mascot
1815.9963	1816.0088	0.0125	7	265	281	KLLDFAEAGDNIGALLR	88	100			Mascot
1976.9556	1976.9718	0.0162	8	321	335	HTPFFNGYRPQFYFR					Mascot
1976.9556	1976.9718	0.0162	8	321	335	HTPFFNGYRPQFYFR	42	99.959			Mascot
1989.9877	1989.9689	-0.0188	-9	58	74	ERGITINTAHVEYETEK					Mascot
2153.0947	2153.1152	0.0205	10	206	224	DTSKPFLMPVEDVFTITGR					Mascot
2153.0947	2153.1152	0.0205	10	206	224	DTSKPFLMPVEDVFTITGR	91	100			Mascot
2169.0896	2169.1016	0.012	6	206	224	DTSKPFLMPVEDVFTITGR			Oxidation (M)[8]		Mascot
2169.0896	2169.1016	0.012	6	206	224	DTSKPFLMPVEDVFTITGR	44	99.975	Oxidation (M)[8]		Mascot
2182.9553	2183.1104	0.1551	71	138	155	CDMVDDEELLELVEMEVR			Oxidation (M)[3]		Mascot
2743.4236	2743.4526	0.029	11	291	315	GQVVCKPGSINPHTQFAAEVYVLTK			Carbamidomethyl (C)[5]		Mascot
2743.4236	2743.4526	0.029	11	291	315	GQVVCKPGSINPHTQFAAEVYVLTK	44	99.976	Carbamidomethyl (C)[5]		Mascot

Calc. Mass	Obsrv. Mass	± da	± ppm	Start Seq.	End Seq.	Sequence	Ion Score	C. I. %	Modification	Rank	Result Type
3271.6528	3271.7056	0.0528	16	291	320	GQVVCKPGSINPHTQFA AEVYVLTKEEGGR			Carbamidomethyl (C)[5]		Mascot

2	Uncharacterized protein OS=Alicyclobacillus acidoterrestris (strain ATCC 49025 / DSM 3922 / CIP 106132 / NCIMB 13137 / GD3B) GN=N007_18315 PE=4 SV=1	tr|T0CJX8|T0CJX8 _ALIAG	15049.7	8.93	7	37	14.297

Peptide Information

Calc. Mass	Obsrv. Mass	± da	± ppm	Start Seq.	End Seq.	Sequence	Ion Score	C. I. %	Modification	Rank	Result Type
1268.6522	1268.6788	0.0266	21	97	107	LGYIEQGVFDK					Mascot
1674.819	1674.7942	-0.0248	-15	22	35	RSMTLCENIYSLTK			Oxidation (M)[3]		Mascot
1815.8694	1816.0088	0.1394	77	73	88	QMAFYSNALGSLQETR					Mascot
1815.8694	1816.0088	0.1394	77	73	88	QMAFYSNALGSLQETR					Mascot
1932.0293	1931.9546	-0.0747	-39	108	124	LDAEAQELVRMLVGMIK			Oxidation (M)[11]		Mascot
1943.9644	1943.9139	-0.0505	-26	72	88	KQMAFYSNALGSLQETR					Mascot
1959.9594	1959.9496	-0.0098	-5	72	88	KQMAFYSNALGSLQETR			Oxidation (M)[3]		Mascot
1959.9594	1959.9496	-0.0098	-5	72	88	KQMAFYSNALGSLQETR			Oxidation (M)[3]		Mascot
2002.9977	2002.9298	-0.0679	-34	23	39	SMTLCENIYSLTKQFPK					Mascot
2166.0825	2166.1846	0.1021	47	52	71	SSSSVPLNIAEGNSFQLY PR					Mascot

3	Succinate dehydrogenase flavoprotein subunit OS=Alicyclobacillus acidoterrestris (strain ATCC 49025 / DSM 3922 / CIP 106132 / NCIMB 13137 / GD3B) GN=sdhA PE=4 SV=1	tr|T0BYA7|T0BYA 7_ALIAG	65656.8	5.81	12	32	0

Peptide Information

Calc. Mass	Obsrv. Mass	± da	± ppm	Start Seq.	End Seq.	Sequence	Ion Score	C. I. %	Modification	Rank	Result Type
1701.8218	1701.9248	0.103	61	416	430	SVDSLPESLFESYTK					Mascot
1709.835	1709.8881	0.0531	31	189	205	ADAVVMCTGGNGLIFGK			Carbamidomethyl (C)[7]		Mascot
1725.83	1725.8669	0.0369	21	189	205	ADAVVMCTGGNGLIFGK			Carbamidomethyl (C)[7], Oxidation (M)[6]		Mascot
1809.9939	1809.9502	-0.0437	-24	510	525	NMLHMARVITLGALNR					Mascot
1829.9167	1830.0232	0.1065	58	416	431	SVDSLPESLFESYTKK					Mascot
1934.9575	1934.9624	0.0049	3	173	188	GIVAQDLRSMEIMHFR			Oxidation (M)[10,13]		Mascot
1972.1147	1971.9434	-0.1713	-87	2	21	AEQTIIVVGGGLAGLMTTI K					Mascot
2071.9753	2072.0737	0.0984	47	227	245	YANGEMIQVHPTAIPGDD K			Oxidation (M)[6]		Mascot
2175.0613	2175.1018	0.0405	19	454	471	ELGQWMNDNVTVVRVN ER			Oxidation (M)[6]		Mascot
2185.9858	2186.1194	0.1336	61	155	172	YEGWDFLGAVIDDEQICR			Carbamidomethyl (C)[17]		Mascot
2726.1755	2726.4377	0.2622	96	57	80	GEGDSPWEHFDDTIYGG DFLANQR					Mascot

2741.303	2741.4143	0.1113	41	181	205	SMEIMHFRADAVVMCTG GNGLIFGK			Carbamidomethyl (C)[15]		Mascot
2743.2305	2743.4526	0.2221	81	432	453	YQDDFESILKMEGDENP YQLHR			Oxidation (M)[11]		Mascot
2743.2305	2743.4526	0.2221	81	432	453	YQDDFESILKMEGDENP YQLHR			Oxidation (M)[11]		Mascot

4 Dephospho-CoA kinase OS=Alicyclobacillus acidoterrestris (strain ATCC 49025 / DSM 3922 / CIP 106132 / NCIMB 13137 / GD3B) GN=coaE PE=3 SV=1 | tr|T0C4I7|T0C4I7_ALIAG | 22742.8 | 5.83 | 7 | 31 | 0

Peptide Information

Calc. Mass	Obsrv. Mass	± da	± ppm	Start Seq.	End Seq.	Sequence	Ion Score	C. I. %	Modification	Rank	Result Type
1130.5874	1130.6949	0.1075	95	158	167	ISAQMPIDQK					Mascot
1233.6873	1233.6403	-0.047	-38	1	13	MIVGLTGGIATGK			Oxidation (M)[1]		Mascot
1461.7114	1461.7216	0.0102	7	143	155	LMARDGLNESEAR					Mascot
1477.7064	1477.7289	0.0225	15	143	155	LMARDGLNESEAR			Oxidation (M)[2]		Mascot
1673.8857	1673.8022	-0.0835	-50	51	66	VFGDAVLAPDGTLDRK					Mascot
1701.892	1701.9248	0.0328	19	50	65	RVFGDAVLAPDGTLDR					Mascot
1976.0846	1975.931	-0.1536	-78	2	21	IVGLTGGIATGKSTVSDM LR					Mascot
2123.1199	2123.0679	-0.052	-24	1	21	MIVGLTGGIATGKSTVSD MLR			Oxidation (M)[1]		Mascot

5 Uncharacterized protein OS=Alicyclobacillus acidoterrestris (strain ATCC 49025 / DSM 3922 / CIP 106132 / NCIMB 13137 / GD3B) GN=N007_10460 PE=4 SV=1 | tr|T0D4I8|T0D4I8_ALIAG | 6408.1 | 4.3 | 4 | 30 | 0

Peptide Information

Calc. Mass	Obsrv. Mass	± da	± ppm	Start Seq.	End Seq.	Sequence	Ion Score	C. I. %	Modification	Rank	Result Type
1124.5219	1124.5863	0.0644	57	10	19	IESDGGDIYR					Mascot
1461.7697	1461.7216	-0.0481	-33	33	45	QLVVFAEQLSDGR					Mascot
2105.0259	2105.0979	0.072	34	33	51	QLVVFAEQLSDGRADDE GR					Mascot
2109.0723	2109.1047	0.0324	15	2	19	TDRPIFVRIESDGGDIYR					Mascot

6 Glycosyl transferase OS=Alicyclobacillus acidoterrestris (strain ATCC 49025 / DSM 3922 / CIP 106132 / NCIMB 13137 / GD3B) GN=N007_03170 PE=4 SV=1 | tr|T0CFI7|T0CFI7_ALIAG | 43243.4 | 7.19 | 9 | 27 | 0

Peptide Information

Calc. Mass	Obsrv. Mass	± da	± ppm	Start Seq.	End Seq.	Sequence	Ion Score	C. I. %	Modification	Rank	Result Type
989.4898	989.4909	0.0011	1	330	337	QLLTDDER					Mascot
1233.6475	1233.6403	-0.0072	-6	173	183	GVDVELFQQAK					Mascot

Calc. Mass	Obsrv. Mass	± da	± ppm	Start Seq.	End Seq.	Sequence	Ion Score	C. I. %	Modification	Rank	Result Type
1389.6831	1389.6777	-0.0054	-4	360	370	QLLEHYEAVCK			Carbamidomethyl (C)[10]		Mascot
1389.7485	1389.6777	-0.0708	-51	173	184	GVDVELFQQAKR					Mascot
1725.8741	1725.8669	-0.0072	-4	360	373	QLLEHYEAVCKAHK			Carbamidomethyl (C)[10]		Mascot
1934.8623	1934.9624	0.1001	52	312	329	AGFIFDSSSDAAMIECVR			Oxidation (M)[13]		Mascot
1975.8889	1975.931	0.0421	21	312	329	AGFIFDSSSDAAMIECVR			Carbamidomethyl (C)[16]		Mascot
1999.0205	1998.9441	-0.0764	-38	3	20	IAMFTETFLPSTDGIVTR					Mascot
2015.0155	2014.9178	-0.0977	-48	3	20	IAMFTETFLPSTDGIVTR			Oxidation (M)[3]		Mascot
2181.1665	2181.0693	-0.0972	-45	47	66	YASATIVGIPAMPFILYPEK					Mascot
2183.1226	2183.1104	-0.0122	-6	124	139	HYKLNFLEPALWWYFR					Mascot

7	Glutamate racemase OS=Alicyclobacillus acidoterrestris (strain ATCC 49025 / DSM 3922 / CIP 106132 / NCIMB 13137 / GD3B) GN=murI PE=3 SV=1	tr|T0C7V4|T0C7V4_ALIAG	30306	6.06	7	26	0

Peptide Information

Calc. Mass	Obsrv. Mass	± da	± ppm	Start Seq.	End Seq.	Sequence	Ion Score	C. I. %	Modification	Rank	Result Type
1701.9283	1701.9248	-0.0035	-2	114	129	IGVIGTSVTIQSHAYR					Mascot
1830.0232	1830.0232	0	0	114	130	IGVIGTSVTIQSHAYRK					Mascot
1990.0063	1989.9689	-0.0374	-19	3	22	ACEQPIGIFDSGVGGLTVAR					Mascot
2003.0664	2002.9298	-0.1366	-68	70	89	CLVIACNTATAVALEALQAK					Mascot
2049.0044	2049.0034	-0.001	0	139	155	MHVVEHACPEFVPLVER			Carbamidomethyl (C)[8]		Mascot
2064.9995	2064.9961	-0.0034	-2	139	155	MHVVEHACPEFVPLVER			Carbamidomethyl (C)[8], Oxidation (M)[1]		Mascot
2175.1228	2175.1018	-0.021	-10	2	22	KACEQPIGIFDSGVGGLTVAR			Carbamidomethyl (C)[3]		Mascot
2181.239	2181.0693	-0.1697	-78	257	278	TPVASDAVVPVGLPLPSHAVVR					Mascot

8	Elongation factor G OS=Alicyclobacillus acidoterrestris (strain ATCC 49025 / DSM 3922 / CIP 106132 / NCIMB 13137 / GD3B) GN=fusA PE=3 SV=1	tr|T0BZT0|T0BZT0_ALIAG	76373.8	5.06	12	26	0	3	0

Peptide Information

Calc. Mass	Obsrv. Mass	± da	± ppm	Start Seq.	End Seq.	Sequence	Ion Score	C. I. %	Modification	Rank	Result Type
1233.7103	1233.6403	-0.07	-57	30	39	ILFYTGRVHK					Mascot
1403.7125	1403.6892	-0.0233	-17	482	493	ETITQRVDQEGK					Mascot
1461.7855	1461.7216	-0.0639	-44	350	361	IGRILQMHANHR			Oxidation (M)[7]		Mascot
1628.8643	1628.8235	-0.0408	-25	159	173	AVAIQLPIGAEDEFR					Mascot
1815.8326	1816.0088	0.1762	97	137	152	MDIIGADFLSCVEQMK			Oxidation (M)[1]		Mascot
1815.8326	1816.0088	0.1762	97	137	152	MDIIGADFLSCVEQMK	3	0	Oxidation (M)[1]		Mascot

Calc. Mass	Obsrv. Mass	± da	± ppm	Start Seq.	End Seq.	Sequence	Ion Score	C. I. %	Modification	Rank	Result Type
1839.9423	1839.9426	0.0003	0	247	263	GTVNVQLFPVLCGSSYR					Mascot
1849.9542	1849.8673	-0.0869	-47	362	379	EEISQVYSGDIAAAVGLK					Mascot
1928.8994	1928.9451	0.0457	24	466	481	EFKVDCNVGMPQVAYR			Carbamidomethyl (C)[6], Oxidation (M)[10]		Mascot
2002.0492	2001.9351	-0.1141	-57	328	346	VYSGILESGSYVLNSTKG K					Mascot
2048.9441	2049.0034	0.0593	29	193	210	QSEDTEIPADMRALAEEK			Oxidation (M)[11]		Mascot
2106.0457	2106.1157	0.07	33	211	228	RTELIEAVAEVDEELMMK					Mascot
2151.1367	2151.1047	-0.032	-15	174	192	GIIDLVEMKAIIYTDDLGR			Oxidation (M)[8]		Mascot

9	Ribulokinase OS=Alicyclobacillus acidoterrestris (strain ATCC 49025 / DSM 3922 / CIP 106132 / NCIMB 13137 / GD3B) GN=araB PE=3 SV=1	tr|T0BMB2|T0BMB 2_ALIAG	60526.9	6.06	10	25	0

Peptide Information

Calc. Mass	Obsrv. Mass	± da	± ppm	Start Seq.	End Seq.	Sequence	Ion Score	C. I. %	Modification	Rank	Result Type
1176.6426	1176.5918	-0.0508	-43	122	130	LWKHHAAWK					Mascot
1510.7874	1510.7692	-0.0182	-12	31	43	HRTVYAHGVLSDR					Mascot
1628.8102	1628.8235	0.0133	8	449	462	NAFLMQLYADITGR			Oxidation (M)[5]		Mascot
1672.829	1672.8846	0.0556	33	2	16	SRYTLGLDFGTESAR					Mascot
1705.8909	1705.8093	-0.0816	-48	524	537	LYQVYLTLHDDLGR					Mascot
1819.8644	1819.9572	0.0928	51	1	16	MSRYTLGLDFGTESAR			Oxidation (M)[1]		Mascot
1977.0189	1976.9718	-0.0471	-24	524	539	LYQVYLTLHDDLGRDR					Mascot
1977.0189	1976.9718	-0.0471	-24	524	539	LYQVYLTLHDDLGRDR					Mascot
2016.9695	2016.9182	-0.0513	-25	163	179	MLQVLEEDEQIWAAADR					Mascot
2765.3247	2765.4434	0.1187	43	316	341	DGIVPGYFGYEAGQAAV GDIFAWYAK					Mascot
3271.4497	3271.7056	0.2559	78	488	518	EHGGYDTFAEAVESMTS GHPGAVYSPNPAHR					Mascot

10	Uncharacterized protein OS=Alicyclobacillus acidoterrestris (strain ATCC 49025 / DSM 3922 / CIP 106132 / NCIMB 13137 / GD3B) GN=N007_09530 PE=4 SV=1	tr|T0BW78|T0BW7 8_ALIAG	15404.9	6.82	5	24	0

Peptide Information

Calc. Mass	Obsrv. Mass	± da	± ppm	Start Seq.	End Seq.	Sequence	Ion Score	C. I. %	Modification	Rank	Result Type
857.4111	857.4451	0.034	40	55	63	GHAGSDVSK					Mascot
1140.5792	1140.5774	-0.0018	-2	129	138	MELSMALKFA					Mascot
1795.9225	1795.9319	0.0094	5	110	126	AYGIIPISAFSEVSDAR					Mascot
1795.9225	1795.9319	0.0094	5	110	126	AYGIIPISAFSEVSDAR					Mascot
1839.9423	1839.9426	0.0003	0	87	104	AVVAGYSMGGQIALEFA R					Mascot

2065.1077	2064.9961	-0.1116	-54	110	128	AYGIIPISAFSEVSDARLR	Mascot

Gel Idx/Pos	229/J5	Instr./Gel Origin	BA2151/full sequence test 20150515	Process Status	Analysis Succeeded
Plate [#] Name	[3] full sequence test 20150515	Instrument Sample Name		Spectra	11

Rank	Protein Name	Species	Accession No.	Protein MW	Protein PI	Pep. Count	Protein Score	Protein Score C. I. %	Total Ion Score	Total Ion C. I. %
1	Anti-sigma F factor antagonist OS=Alicyclobacillus acidoterrestris (strain ATCC 49025 / DSM 3922 / CIP 106132 / NCIMB 13137 / GD3B) GN=N007_03480 PE=3 SV=1		tr|T0C8T4|T0C8T4_ALIAG	12526.5	5.55	5	27	0		

Peptide Information

Calc. Mass	Obsrv. Mass	± da	± ppm	Start Seq.	End Seq.	Sequence	Ion Score	C. I. %	Modification	Rank	Result Type
1332.6254	1332.6517	0.0263	20	39	49	TDYQGLVMSFR			Oxidation (M)[8]		Mascot
1459.8043	1459.7852	-0.0191	-13	97	109	VLPLYEAEDLAVK					Mascot
2302.1431	2302.2478	0.1047	45	66	86	FRSISQHGGQMALCEVGPSLK			Carbamidomethyl (C)[14]		Mascot
2316.1177	2316.1733	0.0556	24	30	49	DDIEAQLAKTDYQGLVMSFR			Oxidation (M)[17]		Mascot
2318.1379	2318.1875	0.0496	21	66	86	FRSISQHGGQMALCEVGPSLK			Carbamidomethyl (C)[14], Oxidation (M)[11]		Mascot
2350.2039	2350.1938	-0.0101	-4	7	27	YENGVLVISLQGELDHHAVEK					Mascot

Rank	Protein Name	Species	Accession No.	Protein MW	Protein PI	Pep. Count	Protein Score	Protein Score C. I. %	Total Ion Score	Total Ion C. I. %
2	Uncharacterized protein OS=Alicyclobacillus acidoterrestris (strain ATCC 49025 / DSM 3922 / CIP 106132 / NCIMB 13137 / GD3B) GN=N007_17800 PE=4 SV=1		tr|T0CMX7|T0CMX7_ALIAG	36841	8.52	8	27	0		

Peptide Information

Calc. Mass	Obsrv. Mass	± da	± ppm	Start Seq.	End Seq.	Sequence	Ion Score	C. I. %	Modification	Rank	Result Type
1043.6096	1043.5845	-0.0251	-24	27	36	VEKGLSVSPK					Mascot
1070.5953	1070.5896	-0.0057	-5	147	156	QAQALGIDVR					Mascot
1218.6511	1218.6396	-0.0115	-9	30	40	GLSVSPKDMIR			Oxidation (M)[9]		Mascot
1218.6511	1218.6396	-0.0115	-9	30	40	GLSVSPKDMIR			Oxidation (M)[9]		Mascot
1732.8303	1732.8734	0.0431	25	44	58	GAYVAAWRTHGSWDR					Mascot
2289.1147	2289.1882	0.0735	32	172	192	HFGSLDAALAEIEYVPDATNR					Mascot
2316.1731	2316.1733	0.0002	0	52	72	THGSWDRALIAAGVSLDVYER					Mascot
2322.1169	2322.1729	0.056	24	296	315	ALETVKELECSVEDIWSGEK			Carbamidomethyl (C)[10]		Mascot
2350.0986	2350.1938	0.0952	41	86	105	EAYEAGADISDTTIKHYFYR					Mascot

Rank	Protein Name	Species	Accession No.	Protein MW	Protein PI	Pep. Count	Protein Score	Protein Score C. I. %	Total Ion Score	Total Ion C. I. %
3	Uncharacterized protein OS=Alicyclobacillus acidoterrestris (strain ATCC 49025 / DSM 3922 / CIP		tr|T0BRW8|T0BRW8_ALIAG	15466.9	6.38	5	24	0		

106132 / NCIMB 13137 / GD3B) GN=N007_13665 PE=4 SV=1

Peptide Information

Calc. Mass	Obsrv. Mass	± da	± ppm	Start Seq.	End Seq.	Sequence	Ion Score	C. I. %	Modification	Rank	Result Type
957.4676	957.5094	0.0418	44	102	108	FYEETLR					Mascot
2302.1899	2302.2478	0.0579	25	14	33	NIDETLPFYLHVLGGVLMDR					Mascot
2304.0596	2304.1409	0.0813	35	116	135	LIYMHMASTSGTLIELCEYP			Oxidation (M)[4,6]		Mascot
2318.1851	2318.1875	0.0024	1	14	33	NIDETLPFYLHVLGGVLMDR			Oxidation (M)[18]		Mascot
2445.2634	2445.2209	-0.0425	-17	75	96	GQGVHHVAYRVPNLEQAIEEAK					Mascot
2501.1873	2501.3416	0.1543	62	115	135	RLIYMHMASTSGTLIELCEYP			Carbamidomethyl (C)[18], Oxidation (M)[5]		Mascot

4	Uncharacterized protein OS=Alicyclobacillus acidoterrestris (strain ATCC 49025 / DSM 3922 / CIP 106132 / NCIMB 13137 / GD3B) GN=N007_10785 PE=4 SV=1	tr|T0D4B5|T0D4B5_ALIAG	23005.4	6.65	6	23	0

Peptide Information

Calc. Mass	Obsrv. Mass	± da	± ppm	Start Seq.	End Seq.	Sequence	Ion Score	C. I. %	Modification	Rank	Result Type
947.3532	947.3544	0.0012	1	181	187	CCFSAMR			Carbamidomethyl (C)[1,2], Oxidation (M)[6]		Mascot
1578.8024	1578.8054	0.003	2	38	50	EPNYFALANREVR					Mascot
1937.7584	1937.8969	0.1385	71	172	187	TSMCFPEHRCCFSAMR			Oxidation (M)[3,15]		Mascot
2211.9692	2212.1116	0.1424	64	161	180	GVVSTSNSCNRTSMCFPEHR					Mascot
2395.1296	2395.1841	0.0545	23	152	171	HWAPMWHLRGVVSTSNSCNR			Carbamidomethyl (C)[18]		Mascot
2526.1826	2526.2419	0.0593	23	181	203	CCFSAMRISIGIQSQSVDAYSIF					Mascot

5	Glycosyl transferase OS=Alicyclobacillus acidoterrestris (strain ATCC 49025 / DSM 3922 / CIP 106132 / NCIMB 13137 / GD3B) GN=N007_03170 PE=4 SV=1	tr|T0CFI7|T0CFI7_ALIAG	43243.4	7.19	8	22	0

Peptide Information

Calc. Mass	Obsrv. Mass	± da	± ppm	Start Seq.	End Seq.	Sequence	Ion Score	C. I. %	Modification	Rank	Result Type
829.493	829.4339	-0.0591	-71	68	74	YALPLPR					Mascot
935.4502	935.5356	0.0854	91	154	161	ATMNELEK					Mascot
935.4502	935.5356	0.0854	91	154	161	ATMNELEK					Mascot
1332.6616	1332.6517	-0.0099	-7	360	370	QLLEHYEAVCK					Mascot
1581.8456	1581.8143	-0.0313	-20	140	153	TLHNRADINLATSR					Mascot

Calc. Mass	Obsrv. Mass	± da	± ppm	Start Seq.	End Seq.	Sequence	Ion Score	C. I. %	Modification	Rank	Result Type
1934.8623	1934.9601	0.0978	51	312	329	AGFIFDSSSDAAMIECVR			Oxidation (M)[13]		Mascot
2181.1665	2181.0161	-0.1504	-69	47	66	YASATIVGIPAMPFILYPEK					Mascot
2302.157	2302.2478	0.0908	39	1	20	MRIAMFTETFLPSTDGIVTR			Oxidation (M)[1]		Mascot
2318.1519	2318.1875	0.0356	15	1	20	MRIAMFTETFLPSTDGIVTR			Oxidation (M)[1,5]		Mascot
2376.24	2376.1726	-0.0674	-28	127	144	LNFLEPALWWYFRTLHNR					Mascot

6 Uncharacterized protein OS=Alicyclobacillus acidoterrestris (strain ATCC 49025 / DSM 3922 / CIP 106132 / NCIMB 13137 / GD3B) GN=N007_06495 PE=4 SV=1 — tr|T0D797|T0D797_ALIAG 60539.8 5.45 11 22 0

Peptide Information

Calc. Mass	Obsrv. Mass	± da	± ppm	Start Seq.	End Seq.	Sequence	Ion Score	C. I. %	Modification	Rank	Result Type
829.5002	829.4339	-0.0663	-80	330	336	QRLGISR					Mascot
889.4009	889.3238	-0.0771	-87	417	424	EGEQAEAR					Mascot
935.5196	935.5356	0.016	17	322	329	YAIAIQEK					Mascot
935.5196	935.5356	0.016	17	322	329	YAIAIQEK					Mascot
1043.4865	1043.5845	0.098	94	102	111	NLTSSHDGGR					Mascot
1087.5929	1087.5099	-0.083	-76	425	433	QVLLAQMER					Mascot
1595.8329	1595.7207	-0.1122	-70	338	349	RELDYLGQWFLR					Mascot
2021.8732	2021.9963	0.1231	61	379	395	WSDSPDLSMWDEAPMVR					Mascot
2068.9856	2068.9653	-0.0203	-10	1	18	MTVDVGYNDEIDSVVLQR			Oxidation (M)[1]		Mascot
2212.1445	2212.1116	-0.0329	-15	359	378	LGAAAYGLMKPRHFQGEPPR			Oxidation (M)[9]		Mascot
2225.0657	2225.1248	0.0591	27	249	267	TDSAQWRTFLAAVVEDEMR					Mascot
2291.1125	2291.1914	0.0789	34	497	516	AELTFSDGQLDMPNFHISIR					Mascot
2291.1125	2291.1914	0.0789	34	497	516	AELTFSDGQLDMPNFHISIR					Mascot

7 Uncharacterized protein OS=Alicyclobacillus acidoterrestris (strain ATCC 49025 / DSM 3922 / CIP 106132 / NCIMB 13137 / GD3B) GN=N007_08345 PE=4 SV=1 — tr|T0BNP2|T0BNP2_ALIAG 4326.4 12.18 3 22 0

Peptide Information

Calc. Mass	Obsrv. Mass	± da	± ppm	Start Seq.	End Seq.	Sequence	Ion Score	C. I. %	Modification	Rank	Result Type
832.3869	832.3337	-0.0532	-64	2	8	PETMNPK			Oxidation (M)[4]		Mascot
947.4324	947.3544	-0.078	-82	1	8	MPETMNPK					Mascot
2305.3716	2305.2012	-0.1704	-74	9	30	LLSQVQNAPTVLIVGAGLVTRR					Mascot

8	Uncharacterized protein OS=Alicyclobacillus acidoterrestris (strain ATCC 49025 / DSM 3922 / CIP 106132 / NCIMB 13137 / GD3B) GN=N007_09400 PE=4 SV=1	tr|T0BMH2|T0BMH2_ALIAG	26395.8	5.73	6	22	0

Peptide Information

Calc. Mass	Obsrv. Mass	± da	± ppm	Start Seq.	End Seq.	Sequence	Ion Score	C. I. %	Modification	Rank	Result Type
1047.5251	1047.5759	0.0508	48	129	136	QILNQMER			Oxidation (M)[6]		Mascot
1387.754	1387.6847	-0.0693	-50	39	52	SLSETLGVGQAAVR					Mascot
1574.7081	1574.792	0.0839	53	137	150	DLGNAELGEEADWR					Mascot
1732.9381	1732.8734	-0.0647	-37	195	208	RLVEQHYAIYEAIK					Mascot
2309.1052	2309.1655	0.0603	26	209	228	DQDQTRAVSAMQQHLNHVSK			Oxidation (M)[11]		Mascot
2318.167	2318.1875	0.0205	9	215	236	AVSAMQQHLNHVSKQLQGGDGL					Mascot
2334.1619	2334.1868	0.0249	11	215	236	AVSAMQQHLNHVSKQLQGGDGL			Oxidation (M)[5]		Mascot

9	Fe-S cluster assembly protein SufB OS=Alicyclobacillus acidoterrestris (strain ATCC 49025 / DSM 3922 / CIP 106132 / NCIMB 13137 / GD3B) GN=N007_10370 PE=4 SV=1	tr|T0BUY6|T0BUY6_ALIAG	52438.8	4.98	9	22	0

Peptide Information

Calc. Mass	Obsrv. Mass	± da	± ppm	Start Seq.	End Seq.	Sequence	Ion Score	C. I. %	Modification	Rank	Result Type
869.3709	869.3348	-0.0361	-42	458	465	FEMEGSIG					Mascot
1218.6477	1218.6396	-0.0081	-7	358	370	GLASFAPNAVGSK					Mascot
1218.6477	1218.6396	-0.0081	-7	358	370	GLASFAPNAVGSK					Mascot
1934.8589	1934.9601	0.1012	52	4	18	QLPDMEEYQYGFRDK			Oxidation (M)[5]		Mascot
1952.0382	1951.9282	-0.11	-56	311	330	AMVLSIAVAGKGQHQDAGAK					Mascot
2021.9095	2021.9963	0.0868	43	1	16	MAKQLPDMEEYQYGFR			Oxidation (M)[1]		Mascot
2317.0298	2317.2009	0.1711	74	33	51	TVEEISMMKNEPGWMTDFR			Oxidation (M)[7]		Mascot
2317.0298	2317.2009	0.1711	74	33	51	TVEEISMMKNEPGWMTDFR			Oxidation (M)[7]		Mascot
2322.1257	2322.1729	0.0472	20	139	158	MGVIFCDTDTALREHPEIFK					Mascot
2333.0247	2333.1885	0.1638	70	33	51	TVEEISMMKNEPGWMTDFR			Oxidation (M)[7,8]		Mascot
2349.0195	2349.1758	0.1563	67	33	51	TVEEISMMKNEPGWMTDFR			Oxidation (M)[7,8,15]		Mascot
2386.2146	2386.1348	-0.0798	-33	299	321	YPSVYMQGEGAKAMVLSIAVAGK			Oxidation (M)[6]		Mascot
2395.1421	2395.1841	0.042	18	139	158	MGVIFCDTDTALREHPEIFK			Carbamidomethyl (C)[6], Oxidation (M)[1]		Mascot
2402.2095	2402.1313	-0.0782	-33	299	321	YPSVYMQGEGAKAMVLS			Oxidation (M)[6,14]		Mascot

2531.1807	2531.3774	0.1967	78	194	214	IAVAGK CEVPLQAYFRINSENMG QFER					Mascot

10	Uncharacterized protein OS=Alicyclobacillus acidoterrestris (strain ATCC 49025 / DSM 3922 / CIP 106132 / NCIMB 13137 / GD3B) GN=N007_05575 PE=4 SV=1	tr|T0C3Y7|T0C3Y7_ALIAG	33222.2	6.36	8	22	0

Peptide Information

Calc. Mass	Obsrv. Mass	± da	± ppm	Start Seq.	End Seq.	Sequence	Ion Score	C. I. %	Modification	Rank	Result Type
935.4515	935.5356	0.0841	90	206	213	SMGWGRNK					Mascot
935.4515	935.5356	0.0841	90	206	213	SMGWGRNK					Mascot
1047.5139	1047.5759	0.062	59	176	184	NTISEMQPK					Mascot
1161.5933	1161.5956	0.0023	2	1	10	MNELALVESR					Mascot
1581.802	1581.8143	0.0123	8	82	94	YKTEWQNATLTAR					Mascot
1934.9376	1934.9601	0.0225	12	64	80	EELDSDGMVVLRGEELK			Oxidation (M)[8]		Mascot
2314.1958	2314.144	-0.0518	-22	165	184	QELLLENGELKNTISEMQ PK					Mascot
2330.1909	2330.1296	-0.0613	-26	165	184	QELLLENGELKNTISEMQ PK			Oxidation (M)[17]		Mascot
2347.1375	2347.2161	0.0786	33	185	205	VDVYEDLVSAENYMTIAT VAK			Oxidation (M)[14]		Mascot
2395.2017	2395.1841	-0.0176	-7	265	283	GHEFIYNIVCKHNVFYLN K			Carbamidomethyl (C)[10]		Mascot

Gel Idx/Pos	230/J6	Instr./Gel Origin	BA2151/full sequence test 20150515	Process Status	Analysis Succeeded
Plate [#] Name	[3] full sequence test 20150515	Instrument Sample Name		Spectra	11

Rank	Protein Name	Species	Accession No.	Protein MW	Protein PI	Pep. Count	Protein Score	Protein Score C. I. %	Total Ion Score	Total Ion C. I. %
1	Uncharacterized protein OS=Alicyclobacillus acidoterrestris (strain ATCC 49025 / DSM 3922 / CIP 106132 / NCIMB 13137 / GD3B) GN=N007_08970 PE=4 SV=1		tr|T0BYX4|T0BYX4_ALIAG	114313.8	4.01	6	30	0	30	99.883

Peptide Information

Calc. Mass	Obsrv. Mass	± da	± ppm	Start Seq.	End Seq.	Sequence	Ion Score	C. I. %	Modification	Rank	Result Type
807.4359	807.4467	0.0108	13	121	127	FNTQVAK					Mascot
935.5309	935.5504	0.0195	21	120	127	KFNTQVAK					Mascot
1117.5848	1117.6023	0.0175	16	243	253	DANGNVLSVTK					Mascot
1238.6085	1238.6306	0.0221	18	46	56	VLSNPYEMTGK					Mascot
1254.6035	1254.6232	0.0197	16	46	56	VLSNPYEMTGK			Oxidation (M)[8]		Mascot
2254.0815	2254.0942	0.0127	6	57	76	DSGNTTGFFPIYYFNQALAK					Mascot
2254.0815	2254.0942	0.0127	6	57	76	DSGNTTGFFPIYYFNQALAK	30	99.883			Mascot
2361.2483	2361.155	-0.0933	-40	35	56	ASQLPIVVNGKVLSNPYEMTGK			Oxidation (M)[19]		Mascot

Rank	Protein Name	Species	Accession No.	Protein MW	Protein PI	Pep. Count	Protein Score	Protein Score C. I. %	Total Ion Score	Total Ion C. I. %
2	Uncharacterized protein OS=Alicyclobacillus acidoterrestris (strain ATCC 49025 / DSM 3922 / CIP 106132 / NCIMB 13137 / GD3B) GN=N007_00940 PE=4 SV=1		tr|T0D3D6|T0D3D6_ALIAG	34559.7	5.17	6	18	0		

Peptide Information

Calc. Mass	Obsrv. Mass	± da	± ppm	Start Seq.	End Seq.	Sequence	Ion Score	C. I. %	Modification	Rank	Result Type
935.4614	935.5504	0.089	95	121	128	EDMIRGAK			Oxidation (M)[3]		Mascot
961.4737	961.4018	-0.0719	-75	171	178	LWSAEEAR					Mascot
1439.7754	1439.725	-0.0504	-35	47	59	LGHESAWISKVGR					Mascot
2210.1274	2210.0654	-0.062	-28	228	248	LGPSGCIYATSENRITVPGFK					Mascot
2239.2268	2239.1501	-0.0767	-34	2	22	AELITLGETMVLLKPFHGGGR					Mascot
2383.175	2383.1401	-0.0349	-15	179	199	ATLLPMAREADFFFPGLDEAR			Oxidation (M)[6]		Mascot

Rank	Protein Name	Species	Accession No.	Protein MW	Protein PI	Pep. Count	Protein Score	Protein Score C. I. %	Total Ion Score	Total Ion C. I. %
3	AbrB family transcriptional regulator OS=Alicyclobacillus acidoterrestris (strain ATCC 49025 / DSM 3922 / CIP 106132 / NCIMB 13137 / GD3B) GN=N007_00240 PE=4 SV=1		tr|T0CAR1|T0CAR1_ALIAG	8789.6	6.56	3	17	0		

Peptide Information

Calc. Mass	Obsrv. Mass	± da	± ppm	Start Seq.	End Seq.	Sequence	Ion Score	C. I. %	Modification	Rank	Result Type
1254.5164	1254.6232	0.1068	85	68	79	NICPSCIAEMSS					Mascot
1439.6327	1439.725	0.0923	64	66	79	GKNICPSCIAEMSS					Mascot
2383.1211	2383.1401	0.019	8	48	67	YEPACIFCGQADEIIHFKG K			Carbamidomethyl (C)[5,8]		Mascot

4	Uncharacterized protein OS=Alicyclobacillus acidoterrestris (strain ATCC 49025 / DSM 3922 / CIP 106132 / NCIMB 13137 / GD3B) GN=N007_17070 PE=4 SV=1	tr|T0BBL1|T0BBL1 _ALIAG	16321.6	8.09	4	17	0

Peptide Information

Calc. Mass	Obsrv. Mass	± da	± ppm	Start Seq.	End Seq.	Sequence	Ion Score	C. I. %	Modification	Rank	Result Type
828.4938	828.5497	0.0559	67	71	78	QVAALQAK					Mascot
1015.5101	1015.5251	0.015	15	108	115	TMEIRHGR			Oxidation (M)[2]		Mascot
1238.6641	1238.6306	-0.0335	-27	131	140	FGEFLSRLNR					Mascot
2408.2126	2408.22	0.0074	3	1	20	MDTERDNIDVIQYEVALL VR			Oxidation (M)[1]		Mascot

5	Uncharacterized protein OS=Alicyclobacillus acidoterrestris (strain ATCC 49025 / DSM 3922 / CIP 106132 / NCIMB 13137 / GD3B) GN=N007_17790 PE=4 SV=1	tr|T0CTC5|T0CTC 5_ALIAG	3623.9	4.59	2	16	0

Peptide Information

Calc. Mass	Obsrv. Mass	± da	± ppm	Start Seq.	End Seq.	Sequence	Ion Score	C. I. %	Modification	Rank	Result Type
1254.7164	1254.6232	-0.0932	-74	22	33	AGIRNLVEQAIA					Mascot
2405.238	2405.2861	0.0481	20	1	21	MGYLVIEEDGEINVSPLIR NK			Oxidation (M)[1]		Mascot

6	30S ribosomal protein S2 OS=Alicyclobacillus acidoterrestris (strain ATCC 49025 / DSM 3922 / CIP 106132 / NCIMB 13137 / GD3B) GN=rpsB PE=3 SV=1	tr|T0D3P8|T0D3P8 _ALIAG	26898	6.88	5	16	0

Peptide Information

Calc. Mass	Obsrv. Mass	± da	± ppm	Start Seq.	End Seq.	Sequence	Ion Score	C. I. %	Modification	Rank	Result Type
1117.5095	1117.6023	0.0928	83	87	95	SGMFYVNQR			Oxidation (M)[3]		Mascot
1186.6038	1186.5656	-0.0382	-32	27	35	MARYIFTER					Mascot
1254.6477	1254.6232	-0.0245	-20	49	58	KVEEAYNFVR					Mascot
2236.2019	2236.0842	-0.1177	-53	2	21	AIISMKQLLEAGVHFGHQ TR					Mascot

Calc. Mass	Obsrv. Mass	± da	± ppm	Start Seq.	End Seq.	Sequence	Ion Score	C. I. %	Modification	Rank	Result Type
2383.2373	2383.1401	-0.0972	-41	1	21	MAIISMKQLLEAGVHFGHQTR			Oxidation (M)[1]		Mascot

7	Aspartyl/glutamyl-tRNA(Asn/Gln) amidotransferase subunit B OS=Alicyclobacillus acidoterrestris (strain ATCC 49025 / DSM 3922 / CIP 106132 / NCIMB 13137 / GD3B) GN=gatB PE=3 SV=1	tr|T0D023|T0D023_ALIAG	52757.5	5.02	7	16	0

Peptide Information

Calc. Mass	Obsrv. Mass	± da	± ppm	Start Seq.	End Seq.	Sequence	Ion Score	C. I. %	Modification	Rank	Result Type
906.5043	906.4414	-0.0629	-69	207	214	FGNKAELK					Mascot
919.3904	919.3199	-0.0705	-77	264	270	EEAHDYR					Mascot
1186.5596	1186.5656	0.006	5	17	26	TETKIFCGCK			Carbamidomethyl (C)[7]		Mascot
2209.9233	2210.0654	0.1421	64	174	192	SIMQYCDVSDCKMEEGSLR			Oxidation (M)[3]		Mascot
2279.0757	2279.0984	0.0227	10	167	185	LYLEALKSIMQYCDVSDCK			Carbamidomethyl (C)[13]		Mascot
2291.1118	2291.2139	0.1021	45	186	206	MEEGSLRCDANVSLRPVGETK					Mascot
2383.1809	2383.1401	-0.0408	-17	360	382	SPAECPISPENLAGLIQEVSSGK			Carbamidomethyl (C)[5]		Mascot

8	Uncharacterized protein OS=Alicyclobacillus acidoterrestris (strain ATCC 49025 / DSM 3922 / CIP 106132 / NCIMB 13137 / GD3B) GN=N007_02040 PE=4 SV=1	tr|T0CJ90|T0CJ90_ALIAG	11621.7	5.34	3	15	0

Peptide Information

Calc. Mass	Obsrv. Mass	± da	± ppm	Start Seq.	End Seq.	Sequence	Ion Score	C. I. %	Modification	Rank	Result Type
1186.6176	1186.5656	-0.052	-44	41	50	ENRDPTLSVR					Mascot
2180.1836	2180.0203	-0.1633	-75	78	96	AHEGAEVHVRPIPGRQWIK					Mascot
2253.0527	2253.0071	-0.0456	-20	1	20	MPEITMYIVQGSPDSGQVER			Oxidation (M)[1]		Mascot

9	Uncharacterized protein OS=Alicyclobacillus acidoterrestris (strain ATCC 49025 / DSM 3922 / CIP 106132 / NCIMB 13137 / GD3B) GN=N007_11370 PE=4 SV=1	tr|T0BUL1|T0BUL1_ALIAG	7308.9	8.93	3	15	0

Peptide Information

Calc. Mass	Obsrv. Mass	± da	± ppm	Start Seq.	End Seq.	Sequence	Ion Score	C. I. %	Modification	Rank	Result Type
935.5533	935.5504	-0.0029	-3	47	53	RPLRHEK					Mascot
2210.0039	2210.0654	0.0615	28	51	69	HEKCLNCLESAQFAVSDMK			Carbamidomethyl (C)[4]		Mascot
2408.3696	2408.22	-0.1496	-62	2	28	AGAGGIVVGVVGVVGVVGVVGVVGEMR			Oxidation (M)[26]		Mascot

10	10 kDa chaperonin OS=Alicyclobacillus acidoterrestris	tr|T0BUU2|T0BUU	10468.7	4.94	3	15	0

(strain ATCC 49025 / DSM 3922 / CIP 106132 / NCIMB 13137 / GD3B) GN=groS PE=3 SV=1 | 2_ALIAG

Peptide Information

Calc. Mass	Obsrv. Mass	± da	± ppm	Start Seq.	End Seq.	Sequence	Ion Score	C. I. %	Modification	Rank	Result Type
812.4988	812.5456	0.0468	58	2	8	LKPLADR					Mascot
906.5043	906.4414	-0.0629	-69	52	58	RFELDVK					Mascot
2297.2598	2297.3062	0.0464	20	75	94	VDNEEVLILRESDILAIVE K					Mascot

Gel Idx/Pos	231/J7	Instr./Gel Origin	BA2151/full sequence test 20150515	Process Status	Analysis Succeeded
Plate [#] Name	[3] full sequence test 20150515	Instrument Sample Name		Spectra	11

Rank	Protein Name	Species	Accession No.	Protein MW	Protein PI	Pep. Count	Protein Score	Protein Score C. I. %	Total Ion Score	Total Ion C. I. %
1	Uncharacterized protein OS=Alicyclobacillus acidoterrestris (strain ATCC 49025 / DSM 3922 / CIP 106132 / NCIMB 13137 / GD3B) GN=N007_08970 PE=4 SV=1		tr|T0BYX4|T0BYX4_ALIAG	114313.8	4.01	8	188	100	180	100

Peptide Information

Calc. Mass	Obsrv. Mass	± da	± ppm	Start Seq.	End Seq.	Sequence	Ion Score	C. I. %	Modification	Rank	Result Type
807.4359	807.4377	0.0018	2	121	127	FNTQVAK					Mascot
935.5309	935.5335	0.0026	3	120	127	KFNTQVAK					Mascot
1117.5848	1117.5968	0.012	11	243	253	DANGNVLSVTK					Mascot
1125.6627	1125.6663	0.0036	3	35	45	ASQLPIVVNGK					Mascot
1238.6085	1238.6162	0.0077	6	46	56	VLSNPYEMTGK					Mascot
1238.6085	1238.6162	0.0077	6	46	56	VLSNPYEMTGK	46	99.985			Mascot
1254.6035	1254.6185	0.015	12	46	56	VLSNPYEMTGK			Oxidation (M)[8]		Mascot
1254.6035	1254.6185	0.015	12	46	56	VLSNPYEMTGK	15	81.432	Oxidation (M)[8]		Mascot
2254.0815	2254.0815	0	0	57	76	DSGNTTGFFPIYYFNQALAK					Mascot
2254.0815	2254.0815	0	0	57	76	DSGNTTGFFPIYYFNQALAK	134	100			Mascot
2352.2302	2352.1895	-0.0407	-17	4	28	SLTGIAAAAVVLGAMSPMAFAATSK			Oxidation (M)[15]		Mascot
2524.3262	2524.2119	-0.1143	-45	3	28	RSLTGIAAAAVVLGAMSPMAFAATSK			Oxidation (M)[16,19]		Mascot

Rank	Protein Name	Species	Accession No.	Protein MW	Protein PI	Pep. Count	Protein Score	Protein Score C. I. %	Total Ion Score	Total Ion C. I. %
2	Enolase OS=Alicyclobacillus acidoterrestris (strain ATCC 49025 / DSM 3922 / CIP 106132 / NCIMB 13137 / GD3B) GN=eno PE=3 SV=1		tr|T0D603|T0D603_ALIAG	45854.4	4.81	9	59	99.472	33	99.715

Peptide Information

Calc. Mass	Obsrv. Mass	± da	± ppm	Start Seq.	End Seq.	Sequence	Ion Score	C. I. %	Modification	Rank	Result Type
1073.5375	1073.5437	0.0062	6	2	10	SEIFDVHAR					Mascot
1194.5538	1194.5747	0.0209	17	254	263	YHFEGEGVTR					Mascot
1204.578	1204.6271	0.0491	41	1	10	MSEIFDVHAR					Mascot
1288.6896	1288.6945	0.0049	4	121	132	AAAEEVGLPLYR					Mascot
1764.924	1764.9248	0.0008	0	35	52	AIVPSGASTGAHEAVELR					Mascot
1889.924	1889.9305	0.0065	3	17	34	GNPTVEVEVELESGAFGR					Mascot

Calc. Mass	Obsrv. Mass	± da	± ppm	Start Seq.	End Seq.	Sequence	Ion Score	C. I. %	Modification	Rank	Result Type
1889.924	1889.9305	0.0065	3	17	34	GNPTVEVEVELESGAFGR	9	17.44			Mascot
1903.9331	1903.9427	0.0096	5	1	16	MSEIFDVHAREVLDSR					Mascot
2180.0942	2180.01	-0.0842	-39	35	56	AIVPSGASTGAHEAVELRDGDK					Mascot
2485.1929	2485.2129	0.02	8	155	176	HADNTVDIQEFMVVPHGATTFR					Mascot
2485.1929	2485.2129	0.02	8	155	176	HADNTVDIQEFMVVPHGATTFR	25	97.868			Mascot
2501.188	2501.2295	0.0415	17	155	176	HADNTVDIQEFMVVPHGATTFR			Oxidation (M)[12]		Mascot
2501.188	2501.2295	0.0415	17	155	176	HADNTVDIQEFMVVPHGATTFR			Oxidation (M)[12]		Mascot

3 Uncharacterized protein OS=Alicyclobacillus acidoterrestris (strain ATCC 49025 / DSM 3922 / CIP 106132 / NCIMB 13137 / GD3B) GN=N007_12105 PE=4 SV=1 — tr|T0BSL1|T0BSL1_ALIAG 40660.2 5.65 10 36 8.168

Peptide Information

Calc. Mass	Obsrv. Mass	± da	± ppm	Start Seq.	End Seq.	Sequence	Ion Score	C. I. %	Modification	Rank	Result Type
807.3995	807.4377	0.0382	47	142	147	LDFEQR					Mascot
1125.5898	1125.6663	0.0765	68	280	288	HDITEIIER					Mascot
1252.7261	1252.6268	-0.0993	-79	2	14	LPVGLDLGNGAVK					Mascot
1306.6572	1306.7267	0.0695	53	253	264	NAFPGGRMELAK			Oxidation (M)[8]		Mascot
2289.1367	2289.1431	0.0064	3	175	195	VVSEGHMAFLALCQQSPEKSK					Mascot
2305.1316	2305.1392	0.0076	3	175	195	VVSEGHMAFLALCQQSPEKSK			Oxidation (M)[7]		Mascot
2314.1763	2314.0752	-0.1011	-44	148	168	LSGEHTVRFMSPSPWGGITVR					Mascot
2318.2	2318.1609	-0.0391	-17	280	299	HDITEIIERHLSAAASHVYR					Mascot
2335.2114	2335.1931	-0.0183	-8	350	371	WLNADGMFLTAVRLAADTVTAV					Mascot
2335.2114	2335.1931	-0.0183	-8	350	371	WLNADGMFLTAVRLAADTVTAV					Mascot
2374.1997	2374.1641	-0.0356	-15	41	62	QEPLNALDVEVVSSAFTDGARR					Mascot
2392.1965	2392.2051	0.0086	4	63	84	VFVGKLAADQGPQATYMQPNEK					Mascot

4 ATP-dependent Lon protease OS=Alicyclobacillus acidoterrestris (strain ATCC 49025 / DSM 3922 / CIP 106132 / NCIMB 13137 / GD3B) GN=N007_20830 PE=4 SV=1 — tr|T0DJZ2|T0DJZ2_ALIAG 76559.1 5.57 14 35 0

Peptide Information

Calc. Mass	Obsrv. Mass	± da	± ppm	Start Seq.	End Seq.	Sequence	Ion Score	C. I. %	Modification	Rank	Result Type
807.428	807.4377	0.0097	12	27	33	DLTSMIK					Mascot

Calc. Mass	Obsrv. Mass	± da	± ppm	Start Seq.	End Seq.	Sequence	Ion Score	C. I. %	Modification	Rank	Result Type
935.523	935.5335	0.0105	11	26	33	KDLTSMIK					Mascot
1047.5503	1047.582	0.0317	30	291	299	DKDGIQIMK					Mascot
1073.599	1073.5437	-0.0553	-52	121	130	GVPISPNYVK					Mascot
1117.575	1117.5968	0.0218	20	533	542	FEKSGVASHR					Mascot
1172.631	1172.587	-0.044	-38	188	196	EEWIDVLIR					Mascot
1204.6581	1204.6271	-0.031	-26	135	145	LLAGGIWCMLK					Mascot
1630.8435	1630.8875	0.044	27	69	82	ILAENFVRPDEAEK					Mascot
1889.8884	1889.9305	0.0421	22	293	309	DGIQIMKDYMASGSFAR					Mascot
1889.8884	1889.9305	0.0421	22	293	309	DGIQIMKDYMASGSFAR					Mascot
2019.0791	2019.016	-0.0631	-31	605	624	SVQESLVVLGNMTVGGTI AK			Oxidation (M)[12]		Mascot
2209.1533	2209.0693	-0.084	-38	315	335	TASASMVFIGNINQSLDS LIK					Mascot
2225.1482	2225.1191	-0.0291	-13	315	335	TASASMVFIGNINQSLDS LIK			Oxidation (M)[6]		Mascot
2286.1912	2286.1155	-0.0757	-33	217	237	LVPLVENNYNLCELGPR GTGK					Mascot
2524.1709	2524.2119	0.041	16	135	155	LLAGGIWCMLKMDYFFD EEAR			Oxidation (M)[9]		Mascot
2831.3855	2831.2275	-0.158	-56	495	521	LIPEGMGKPGHVYTVGH GDSGMIGVYK			Oxidation (M)[6,22]		Mascot

5	Uncharacterized protein OS=Alicyclobacillus acidoterrestris (strain ATCC 49025 / DSM 3922 / CIP 106132 / NCIMB 13137 / GD3B) GN=N007_17145 PE=4 SV=1	tr|T0BBJ0|T0BBJ0_ALIAG	10294.4	6.58	6	35	0

Peptide Information

Calc. Mass	Obsrv. Mass	± da	± ppm	Start Seq.	End Seq.	Sequence	Ion Score	C. I. %	Modification	Rank	Result Type
1125.6085	1125.6663	0.0578	51	7	16	MPVQLDAPVR					Mascot
1172.6633	1172.587	-0.0763	-65	31	40	LINSTINELR					Mascot
1186.5409	1186.5995	0.0586	49	62	71	VFESESVMSR			Oxidation (M)[8]		Mascot
1252.6168	1252.6268	0.01	8	19	29	NIYAQNTSDVK					Mascot
2209.1282	2209.0693	-0.0589	-27	72	89	LEEMDEQLLLLQQQIGHR			Oxidation (M)[4]		Mascot
2296.1602	2296.074	-0.0862	-38	72	90	LEEMDEQLLLLQQQIGHR S			Oxidation (M)[4]		Mascot

6	Uncharacterized protein OS=Alicyclobacillus acidoterrestris (strain ATCC 49025 / DSM 3922 / CIP 106132 / NCIMB 13137 / GD3B) GN=N007_09405 PE=4 SV=1	tr|T0BWK5|T0BWK5_ALIAG	27204.3	5.82	9	32	0

Peptide Information

Calc. Mass	Obsrv. Mass	± da	± ppm	Start Seq.	End Seq.	Sequence	Ion Score	C. I. %	Modification	Rank	Result Type
1204.5126	1204.6271	0.1145	95	170	181	DCCGFGGTFAVK					Mascot

Calc. Mass	Obsrv. Mass	± da	± ppm	Start Seq.	End Seq.	Sequence	Ion Score	C. I. %	Modification	Rank	Result Type
1252.5548	1252.6268	0.072	57	182	193	MGDISGAMVDEK					Mascot
1268.5498	1268.6254	0.0756	60	182	193	MGDISGAMVDEK			Oxidation (M)[1]		Mascot
1695.7809	1695.8765	0.0956	56	128	142	FPHAVTYHPSCHGSR					Mascot
2192.0952	2192.0579	-0.0373	-17	223	241	TGQSTRVMHLAELLYEGMR					Mascot
2263.1553	2263.1003	-0.055	-24	128	147	FPHAVTYHPSCHGSRLLGVK			Carbamidomethyl (C)[11]		Mascot
2318.1487	2318.1609	0.0122	5	108	127	TYEFSQFMVNVLKVDDVGAR					Mascot
2425.262	2425.1575	-0.1045	-43	3	24	VSLFITCLVDNIFPNVGVAMTR			Oxidation (M)[20]		Mascot
2584.0823	2584.2241	0.1418	55	170	193	DCCGFGGTFAVKMGDISGAMVDEK			Carbamidomethyl (C)[2,3], Oxidation (M)[13,20]		Mascot
2691.2537	2691.3008	0.0471	18	194	219	AQHVTETGAEVLVGTDMGCLMNIGGR			Oxidation (M)[17,21]		Mascot

7	Uncharacterized protein OS=Alicyclobacillus acidoterrestris (strain ATCC 49025 / DSM 3922 / CIP 106132 / NCIMB 13137 / GD3B) GN=N007_01550 PE=4 SV=1	tr|T0BGG4|T0BGG4_ALIAG	23626.3	5.58	7	31	0

Peptide Information

Calc. Mass	Obsrv. Mass	± da	± ppm	Start Seq.	End Seq.	Sequence	Ion Score	C. I. %	Modification	Rank	Result Type
1073.6201	1073.5437	-0.0764	-71	152	161	GELVDKTLAK					Mascot
2150.0547	2150.0798	0.0251	12	43	62	CETAVGNAEDIQFLREAGVK					Mascot
2151.1743	2151.0952	-0.0791	-37	2	21	TFCIIGVGRFGTGAAIELLR			Carbamidomethyl (C)[3]		Mascot
2225.1934	2225.1191	-0.0743	-33	1	21	MTFCIIGVGRFGTGAAIELLR					Mascot
2241.1882	2241.1074	-0.0808	-36	1	21	MTFCIIGVGRFGTGAAIELLR			Oxidation (M)[1]		Mascot
2282.2148	2282.0859	-0.1289	-56	1	21	MTFCIIGVGRFGTGAAIELLR			Carbamidomethyl (C)[4]		Mascot
2298.2097	2298.0891	-0.1206	-52	1	21	MTFCIIGVGRFGTGAAIELLR			Carbamidomethyl (C)[4], Oxidation (M)[1]		Mascot
2317.2776	2317.124	-0.1536	-66	11	31	FGTGAAIELLRHGHEVLLIDR					Mascot
2317.2776	2317.124	-0.1536	-66	11	31	FGTGAAIELLRHGHEVLLIDR					Mascot
2392.1826	2392.2051	0.0225	9	22	42	HGHEVLLIDRNPSCLEAFADR					Mascot
2524.1907	2524.2119	0.0212	8	131	151	SAILDMIELYEEVFMMEITAK			Oxidation (M)[6,15,16]		Mascot

8	ATP-dependent protease subunit HslV OS=Alicyclobacillus acidoterrestris (strain ATCC 49025 / DSM 3922 / CIP 106132 / NCIMB 13137 / GD3B) GN=hslV PE=3 SV=1	tr|T0C0X6|T0C0X6_ALIAG	18867.7	5.58	6	29	0

Peptide Information

Calc. Mass	Obsrv. Mass	± da	± ppm	Start Seq.	End Seq.	Sequence	Ion Score	C. I. %	Modification	Rank	Result Type

Calc. Mass	Obsrv. Mass	± da	± ppm	Start Seq.	End Seq.	Sequence	Ion Score	C. I. %	Modification	Rank	Result Type
1172.6058	1172.587	-0.0188	-16	78	87	AAVELAQEWR					Mascot
1327.7216	1327.7297	0.0081	6	143	154	NTDLPAEQIAKK					Mascot
2128.9673	2129.1318	0.1645	77	12	32	DGKGAMAGDGQVTLGN SMIMK			Oxidation (M)[6,18,20]		Mascot
2209.0522	2209.0693	0.0171	8	15	36	GAMAGDGQVTLGNSMIM KQGAR			Oxidation (M)[3]		Mascot
2225.0471	2225.1191	0.072	32	15	36	GAMAGDGQVTLGNSMIM KQGAR			Oxidation (M)[3,15]		Mascot
2241.042	2241.1074	0.0654	29	15	36	GAMAGDGQVTLGNSMIM KQGAR			Oxidation (M)[3,15,17]		Mascot
2373.2007	2373.1367	-0.064	-27	155	176	ALEIASEICVFTNDHIIVET VG					Mascot
2501.2957	2501.2295	-0.0662	-26	154	176	KALEIASEICVFTNDHIIVE TVG					Mascot
2501.2957	2501.2295	-0.0662	-26	154	176	KALEIASEICVFTNDHIIVE TVG					Mascot

9 Uncharacterized protein OS=Alicyclobacillus acidoterrestris (strain ATCC 49025 / DSM 3922 / CIP 106132 / NCIMB 13137 / GD3B) GN=N007_18060 PE=4 SV=1 — tr|T0CK27|T0CK27_ALIAG 32733 5.47 8 28 0

Peptide Information

Calc. Mass	Obsrv. Mass	± da	± ppm	Start Seq.	End Seq.	Sequence	Ion Score	C. I. %	Modification	Rank	Result Type
1323.6692	1323.6749	0.0057	4	122	133	YLGNVEGGYPVR					Mascot
1851.9487	1851.9294	-0.0193	-10	100	116	DYVSIINAPTSDKPFGK					Mascot
2139.1484	2139.0461	-0.1023	-48	81	99	DLGLTPQEFFAKYAGIQL K					Mascot
2209.136	2209.0693	-0.0667	-30	23	42	QQASEYRSQYLAGAPVT ALR					Mascot
2254.0525	2254.0815	0.029	13	55	72	LLCYFLGTPPDTFDFEYR			Carbamidomethyl (C)[3]		Mascot
2254.0525	2254.0815	0.029	13	55	72	LLCYFLGTPPDTFDFEYR			Carbamidomethyl (C)[3]		Mascot
2359.1863	2359.1702	-0.0161	-7	144	164	RLALAQIQDGEPVWFGC DVGK			Carbamidomethyl (C)[17]		Mascot
2439.1797	2439.1687	-0.011	-5	196	218	LAYGESLMTHAMVFTGA NVVDGR					Mascot
2691.2205	2691.3008	0.0803	30	169	192	ETGIMDTAFYDYEGILGT AFTMTK			Oxidation (M)[5]		Mascot

10 Uncharacterized protein OS=Alicyclobacillus acidoterrestris (strain ATCC 49025 / DSM 3922 / CIP 106132 / NCIMB 13137 / GD3B) GN=N007_11080 PE=4 SV=1 — tr|T0BV50|T0BV50_ALIAG 3175.5 7.88 3 28 0

Peptide Information

Calc. Mass	Obsrv. Mass	± da	± ppm	Start Seq.	End Seq.	Sequence	Ion Score	C. I. %	Modification	Rank	Result Type
1047.5112	1047.582	0.0708	68	2	12	ARCGGTQAGAR					Mascot
1194.5466	1194.5747	0.0281	24	1	12	MARCGGTQAGAR			Oxidation (M)[1]		Mascot

2932.3901	2932.5317	0.1416	48	4	36	CGGTQAGARVAGIGQGG AGHAPPAVDLGACGAA	Carbamidomethyl (C)[1,30]	Mascot

Gel Idx/Pos	232/J8	Instr./Gel Origin	BA2151/full sequence test 20150515	Process Status	Analysis Succeeded
Plate [#] Name	[3] full sequence test 20150515	Instrument Sample Name		Spectra	11

Rank	Protein Name	Species	Accession No.	Protein MW	Protein PI	Pep. Count	Protein Score	Protein Score C. I. %	Total Ion Score	Total Ion C. I. %
1	Malate dehydrogenase OS=Alicyclobacillus acidoterrestris (strain ATCC 49025 / DSM 3922 / CIP 106132 / NCIMB 13137 / GD3B) GN=mdh PE=3 SV=1		tr|T0BTR2|T0BTR2_ALIAG	33360.7	5.2	17	381	100	271	100

Peptide Information

Calc. Mass	Obsrv. Mass	± da	± ppm	Start Seq.	End Seq.	Sequence	Ion Score	C. I. %	Modification	Rank	Result Type
886.4992	886.5095	0.0103	12	206	212	IEEIVQR					Mascot
900.5513	900.5367	-0.0146	-16	305	312	KVIQVVQS					Mascot
1171.6429	1171.6477	0.0048	4	204	212	ERIEEIVQR					Mascot
1171.6429	1171.6477	0.0048	4	204	212	ERIEEIVQR	29	99.213			Mascot
1176.626	1176.6312	0.0052	4	189	199	YSNVAGVPLEK					Mascot
1199.6743	1199.6742	-0.0001	0	144	155	VIGQAGVLDTAR					Mascot
1258.6638	1258.6686	0.0048	4	93	104	DDLVNTNAGIVK					Mascot
1258.6638	1258.6686	0.0048	4	93	104	DDLVNTNAGIVK	40	99.936			Mascot
1311.7406	1311.7426	0.002	2	283	293	IVELELLPEEK					Mascot
1469.8184	1469.8146	-0.0038	-3	142	155	NRVIGQAGVLDTAR					Mascot
1716.866	1716.8673	0.0013	1	47	62	ALDMLEAMPVIGSDVR	23	97.013			Mascot
1732.8608	1732.8611	0.0003	0	47	62	ALDMLEAMPVIGSDVR			Oxidation (M)[4]		Mascot
1732.8608	1732.8611	0.0003	0	47	62	ALDMLEAMPVIGSDVR	34	99.772	Oxidation (M)[4]		Mascot
1748.8558	1748.8473	-0.0085	-5	47	62	ALDMLEAMPVIGSDVR			Oxidation (M)[4,8]		Mascot
1748.8558	1748.8473	-0.0085	-5	47	62	ALDMLEAMPVIGSDVR	40	99.942	Oxidation (M)[4,8]		Mascot
1915.0066	1915.0042	-0.0024	-1	87	104	KPGMSRDDLVNTNAGIVK					Mascot
1931.0016	1931.0002	-0.0014	-1	87	104	KPGMSRDDLVNTNAGIVK			Oxidation (M)[4]		Mascot
1931.0016	1931.0002	-0.0014	-1	87	104	KPGMSRDDLVNTNAGIVK	13	71.277	Oxidation (M)[4]		Mascot
2011.0416	2011.0219	-0.0197	-10	27	44	ELGDVVLLDIPQMENPTK					Mascot
2019.0291	2019.012	-0.0171	-8	7	26	ISVIGAGFTGATTAFMLAMK			Oxidation (M)[16,19]		Mascot
2027.0366	2027.037	0.0004	0	27	44	ELGDVVLLDIPQMENPTK			Oxidation (M)[13]		Mascot
2147.124	2147.1089	-0.0151	-7	6	26	KISVIGAGFTGATTAFMLAMK			Oxidation (M)[17,20]		Mascot
2478.2722	2478.2722	0	0	63	86	IVGTANYEDTADSDLVIITAGIAR					Mascot
2478.2722	2478.2722	0	0	63	86	IVGTANYEDTADSDLVIITAGIAR	118	100			Mascot

Calc. Mass	Obsrv. Mass	± da	± ppm	Start Seq.	End Seq.	Sequence	Ion Score	C. I. %	Modification	Rank	Result Type
2546.3323	2546.3342	0.0019	1	113	135	YSPNTILIVLSNPVDAMTY VAHK					Mascot
2562.3271	2562.323	-0.0041	-2	113	135	YSPNTILIVLSNPVDAMTY VAHK			Oxidation (M)[17]		Mascot
2562.3271	2562.323	-0.0041	-2	113	135	YSPNTILIVLSNPVDAMTY VAHK	31	99.512	Oxidation (M)[17]		Mascot
3139.5979	3139.5854	-0.0125	-4	215	246	TGGGEIVSLLGNGSAYYA PAASLAQMAESILK					Mascot
3155.593	3155.6218	0.0288	9	215	246	TGGGEIVSLLGNGSAYYA PAASLAQMAESILK			Oxidation (M)[26]		Mascot
3599.7839	3599.866	0.0821	23	156	188	FNTFVAEELGVSVEDVH GFVLGVHGDDMVPLVR			Oxidation (M)[28]		Mascot

2	Transketolase OS=Alicyclobacillus acidoterrestris (strain ATCC 49025 / DSM 3922 / CIP 106132 / NCIMB 13137 / GD3B) GN=N007_12855 PE=3 SV=1	tr|T0BI13|T0BI13_ ALIAG	72451.8	5.57	12	78	99.993	51	99.995

Peptide Information

Calc. Mass	Obsrv. Mass	± da	± ppm	Start Seq.	End Seq.	Sequence	Ion Score	C. I. %	Modification	Rank	Result Type
822.4105	822.3947	-0.0158	-19	402	408	NVDYGVR					Mascot
957.4611	957.4585	-0.0026	-3	135	142	FMAATFNR					Mascot
973.456	973.4635	0.0075	8	135	142	FMAATFNR			Oxidation (M)[2]		Mascot
994.5104	994.5233	0.0129	13	210	217	SYGWNVLR					Mascot
1026.5942	1026.5087	-0.0855	-83	509	518	DAPVALALTR					Mascot
1223.642	1223.6437	0.0017	1	385	394	TFIKSSEHFK					Mascot
1373.6597	1373.665	0.0053	4	50	60	FNPENPSWINR					Mascot
1387.653	1387.6595	0.0065	5	284	294	YESFFVPDEVR					Mascot
1387.653	1387.6595	0.0065	5	284	294	YESFFVPDEVR	49	99.993			Mascot
1644.7877	1644.8099	0.0222	13	50	62	FNPENPSWINRDR					Mascot
1862.9746	1862.9463	-0.0283	-15	181	197	LIVLYDSNDISLDGPTK					Mascot
1985.9774	1985.9702	-0.0072	-4	218	235	VEDGNNLDEIERAIAEAK					Mascot
2299.1111	2299.1289	0.0178	8	25	46	ANSGHPGLPMGAAPMAY VLWSR			Oxidation (M)[10]		Mascot
2315.106	2315.106	0	0	25	46	ANSGHPGLPMGAAPMAY VLWSR			Oxidation (M)[10,15]		Mascot
2335.2544	2335.1821	-0.0723	-31	236	257	TFTDGPTLIEVKTIIGYGA PNK					Mascot
2335.2544	2335.1821	-0.0723	-31	236	257	TFTDGPTLIEVKTIIGYGA PNK	2	0			Mascot

3	Glyoxalase OS=Alicyclobacillus acidoterrestris (strain ATCC 49025 / DSM 3922 / CIP 106132 / NCIMB 13137 / GD3B) GN=N007_11010 PE=4 SV=1	tr|T0D416|T0D416 _ALIAG	14286.1	5.52	7	43	80.367

Peptide Information

Calc. Mass	Obsrv. Mass	± da	± ppm	Start Seq.	End Seq.	Sequence	Ion Score	C. I. %	Modification	Rank	Result Type

Calc. Mass	Obsrv. Mass	± da	± ppm	Start Seq.	End Seq.	Sequence	Ion Score	C. I. %	Modification	Rank	Result Type
957.5403	957.4585	-0.0818	-85	42	49	WIEIAPTK					Mascot
1325.6995	1325.7251	0.0256	19	95	107	GVAVGEIVHMGTR					Mascot
1469.7893	1469.8146	0.0253	17	95	108	GVAVGEIVHMGTRK			Oxidation (M)[10]		Mascot
1772.7578	1772.8115	0.0537	30	26	41	MGFSVISEEDNGQGMR			Oxidation (M)[1]		Mascot
2010.8903	2011.0219	0.1316	65	109	125	VFNFADNEGNYFAVMEK			Oxidation (M)[15]		Mascot
2298.9973	2299.1289	0.1316	57	109	128	VFNFADNEGNYFAVMEK SNS			Oxidation (M)[15]		Mascot
2388.1541	2388.1521	-0.002	-1	5	25	VGQVMLYVSNQAEAVSF WTDK			Oxidation (M)[5]		Mascot

4 Uncharacterized protein OS=Alicyclobacillus acidoterrestris (strain ATCC 49025 / DSM 3922 / CIP 106132 / NCIMB 13137 / GD3B) GN=N007_14080 PE=4 SV=1 | tr|T0BFZ5|T0BFZ5_ALIAG | 14440.4 | 6.84 | 6 | 33 | 0

Peptide Information

Calc. Mass	Obsrv. Mass	± da	± ppm	Start Seq.	End Seq.	Sequence	Ion Score	C. I. %	Modification	Rank	Result Type
1155.6005	1155.6506	0.0501	43	6	16	SLEIDGVTHGK					Mascot
1253.7114	1253.6111	-0.1003	-80	51	60	QVQLVFQHVR					Mascot
1311.7015	1311.7426	0.0411	31	5	16	RSLEIDGVTHGK					Mascot
1746.9473	1746.8811	-0.0662	-38	51	64	QVQLVFQHVRSFMK					Mascot
1762.9421	1762.8713	-0.0708	-40	51	64	QVQLVFQHVRSFMK			Oxidation (M)[13]		Mascot
1915.0132	1915.0042	-0.009	-5	115	132	NGALVQVEITAVIDSTRE					Mascot
2501.2017	2501.2783	0.0766	31	76	95	MTVYLADEQYRDLVNEE WVK					Mascot

5 Phenylalanine--tRNA ligase alpha subunit OS=Alicyclobacillus acidoterrestris (strain ATCC 49025 / DSM 3922 / CIP 106132 / NCIMB 13137 / GD3B) GN=pheS PE=3 SV=1 | tr|T0CRM3|T0CRM3_ALIAG | 39583.1 | 5.93 | 11 | 33 | 0

Peptide Information

Calc. Mass	Obsrv. Mass	± da	± ppm	Start Seq.	End Seq.	Sequence	Ion Score	C. I. %	Modification	Rank	Result Type
935.4979	935.4993	0.0014	1	226	233	GIRMSDLK			Oxidation (M)[4]		Mascot
957.4901	957.4585	-0.0316	-33	242	249	ALFGEHQR					Mascot
1010.5629	1010.5131	-0.0498	-49	8	16	QELATLHAK					Mascot
1343.7291	1343.6736	-0.0555	-41	106	117	HRDVGAVHPISR					Mascot
1373.6768	1373.665	-0.0118	-9	81	92	RESIEAAEQNAR					Mascot
1408.7505	1408.8534	0.1029	73	229	241	MSDLKGVLEAFAK					Mascot
1431.8066	1431.6913	-0.1153	-81	334	344	QLYQNDLRLLR					Mascot
1772.9442	1772.8115	-0.1327	-75	234	249	GVLEAFAKALFGEHQR					Mascot
2041.0834	2041.0226	-0.0608	-30	183	200	TMEQMHPHAPIKVIVPGR					Mascot

Calc. Mass	Obsrv. Mass	± da	± ppm	Start Seq.	End Seq.	Sequence	Ion Score	C. I. %	Modification	Rank	Result Type
2315.1926	2315.106	-0.0866	-37	50	69	QMGQLDAQTRPLFGQLLNER					Mascot
2568.3264	2568.3193	-0.0071	-3	82	105	ESIEAAEQNARLAAETIDITLPGR					Mascot

6	Uncharacterized protein OS=Alicyclobacillus acidoterrestris (strain ATCC 49025 / DSM 3922 / CIP 106132 / NCIMB 13137 / GD3B) GN=N007_14025 PE=4 SV=1	tr|T0CU30|T0CU30_ALIAG	30109.7	7.14	9	32	0

Peptide Information

Calc. Mass	Obsrv. Mass	± da	± ppm	Start Seq.	End Seq.	Sequence	Ion Score	C. I. %	Modification	Rank	Result Type
800.3937	800.3841	-0.0096	-12	256	261	LGYYER					Mascot
1010.5451	1010.5131	-0.032	-32	125	132	LECKHPVK			Carbamidomethyl (C)[3]		Mascot
1171.5889	1171.6477	0.0588	50	140	150	KNPLHCTSSGK					Mascot
1171.5889	1171.6477	0.0588	50	140	150	KNPLHCTSSGK					Mascot
1399.6964	1399.7067	0.0103	7	251	261	EISNRLGYYER					Mascot
1429.707	1429.7073	0.0003	0	93	104	EGQHLLEDFVSR					Mascot
1684.8766	1684.8844	0.0078	5	204	218	EGVHSVAVPVRDYTR					Mascot
2066.0586	2066.0969	0.0383	19	151	167	TILAFQSDEMIEEVIRR			Oxidation (M)[10]		Mascot
2315.1443	2315.106	-0.0383	-17	105	124	FDETVHLAVLEDYSTVYVSK					Mascot
2335.1851	2335.1821	-0.003	-1	25	46	CFTTDEPELGITQIAQALGLSK					Mascot
2335.1851	2335.1821	-0.003	-1	25	46	CFTTDEPELGITQIAQALGLSK					Mascot

7	Uncharacterized protein OS=Alicyclobacillus acidoterrestris (strain ATCC 49025 / DSM 3922 / CIP 106132 / NCIMB 13137 / GD3B) GN=N007_17900 PE=4 SV=1	tr|T0BIX1|T0BIX1_ALIAG	6893.5	9.52	4	29	0

Peptide Information

Calc. Mass	Obsrv. Mass	± da	± ppm	Start Seq.	End Seq.	Sequence	Ion Score	C. I. %	Modification	Rank	Result Type
1128.583	1128.6848	0.1018	90	2	11	GITISNMHQK					Mascot
1770.8326	1770.8286	-0.004	-2	45	60	SRASEDLYSMTTPNAK					Mascot
1786.8276	1786.8265	-0.0011	-1	45	60	SRASEDLYSMTTPNAK			Oxidation (M)[10]		Mascot
1955.061	1954.9791	-0.0819	-42	12	29	YQFGAAPPRSGLTHALLR					Mascot
2247.1162	2247.0691	-0.0471	-21	1	20	MGITISNMHQKYQFGAAPPR					Mascot

8	Uncharacterized protein OS=Alicyclobacillus acidoterrestris (strain ATCC 49025 / DSM 3922 / CIP 106132 / NCIMB 13137 / GD3B) GN=N007_05685 PE=4 SV=1	tr|T0DDK8|T0DDK8_ALIAG	13901.2	9.9	6	28	0

Peptide Information

Calc. Mass	Obsrv. Mass	± da	± ppm	Start Seq.	End Seq.	Sequence	Ion Score	C. I. %	Modification	Rank	Result Type
1343.7253	1343.6736	-0.0517	-38	53	63	LMQWNNVLVAR					Mascot
1373.613	1373.665	0.052	38	64	74	KSGYVWCGWCK			Carbamidomethyl (C)[7]		Mascot
1487.8152	1487.7252	-0.09	-60	53	64	LMQWNNVLVARK			Oxidation (M)[2]		Mascot
1705.7455	1705.8536	0.1081	63	84	99	GIVTCECAATGHAEMR			Carbamidomethyl (C)[5]		Mascot
1762.767	1762.8713	0.1043	59	84	99	GIVTCECAATGHAEMR			Carbamidomethyl (C)[5,7]		Mascot
2292.0681	2292.0791	0.011	5	80	99	YLPRGIVTCECAATGHAE MR			Carbamidomethyl (C)[9,11]		Mascot
2501.2783	2501.2783	0	0	11	31	LDYETERSIPVGLHNVLD FQR					Mascot

9	GTP cyclohydrolase 1 OS=Alicyclobacillus acidoterrestris (strain ATCC 49025 / DSM 3922 / CIP 106132 / NCIMB 13137 / GD3B) GN=folE PE=3 SV=1	tr|T0D455|T0D455 _ALIAG	21118.9	6.16	6	28	0

Peptide Information

Calc. Mass	Obsrv. Mass	± da	± ppm	Start Seq.	End Seq.	Sequence	Ion Score	C. I. %	Modification	Rank	Result Type
1716.8595	1716.8673	0.0078	5	140	155	GVIVTLDGIHLCMCAR			Oxidation (M)[13]		Mascot
1954.9838	1954.9791	-0.0047	-2	1	16	MSITMDKHQLITDHIR			Oxidation (M)[1]		Mascot
2318.0588	2318.1313	0.0725	31	73	90	DIDYYTFCEHHLIPFYGK			Carbamidomethyl (C)[8]		Mascot
2347.1677	2347.1079	-0.0598	-25	170	190	LGAFSEHASLVQEFEQAL SRQ					Mascot
3383.6306	3383.7563	0.1257	37	73	100	DIDYYTFCEHHLIPFYGKA HIAYLPSGR			Carbamidomethyl (C)[8]		Mascot
3599.7876	3599.866	0.0784	22	124	155	MTQQIADAVMNVLEPKG VIVTLDGIHLCMCAR			Carbamidomethyl (C)[28,30], Oxidation (M)[1]		Mascot

10	Uncharacterized protein OS=Alicyclobacillus acidoterrestris (strain ATCC 49025 / DSM 3922 / CIP 106132 / NCIMB 13137 / GD3B) GN=N007_04425 PE=3 SV=1	tr|T0BVJ7|T0BVJ7 _ALIAG	47727.8	5.81	9	27	0

Peptide Information

Calc. Mass	Obsrv. Mass	± da	± ppm	Start Seq.	End Seq.	Sequence	Ion Score	C. I. %	Modification	Rank	Result Type
973.4771	973.4635	-0.0136	-14	100	107	ALCDEPLR			Carbamidomethyl (C)[3]		Mascot
1214.7178	1214.6281	-0.0897	-74	322	333	EAVVVVLGLGMK					Mascot
1314.7124	1314.645	-0.0674	-51	305	316	IARDVAQSLADR					Mascot
1469.8435	1469.8146	-0.0289	-20	209	221	IAQLSKVIENAQR					Mascot
1644.8625	1644.8099	-0.0526	-32	222	236	DVNLAMVQELQAVAK			Oxidation (M)[6]		Mascot
2147.1179	2147.1089	-0.009	-4	237	254	AHQVNLFELIQMVNTHPR					Mascot
2492.2703	2492.2822	0.0119	5	163	185	VAEGRAMEEFQTLDIVVG					Mascot

						GLTEK		
2540.3396	2540.2815	-0.0581	-23	76	99	TAVAAWEATTYLVTVGV PVHPETK		Mascot
3494.7988	3494.7297	-0.0691	-20	76	107	TAVAAWEATTYLVTVGV PVHPETKALCDEPLR	Carbamidomethyl (C)[27]	Mascot

Gel Idx/Pos	233/J9	Instr./Gel Origin	BA2151/full sequence test 20150515	Process Status	Analysis Succeeded
Plate [#] Name	[3] full sequence test 20150515	Instrument Sample Name		Spectra	11

Rank	Protein Name	Species	Accession No.	Protein MW	Protein PI	Pep. Count	Protein Score	Protein Score C. I. %	Total Ion Score	Total Ion C. I. %
1	Uncharacterized protein OS=Alicyclobacillus acidoterrestris (strain ATCC 49025 / DSM 3922 / CIP 106132 / NCIMB 13137 / GD3B) GN=N007_04435 PE=4 SV=1		tr|T0C6H1|T0C6H1_ALIAG	32737.6	5.18	14	499	100	422	100

Peptide Information

Calc. Mass	Obsrv. Mass	± da	± ppm	Start Seq.	End Seq.	Sequence	Ion Score	C. I. %	Modification	Rank	Result Type
839.5097	839.5212	0.0115	14	179	186	ILPAAQAR					Mascot
1013.5051	1013.5065	0.0014	1	221	228	ALQYDFTR					Mascot
1013.5051	1013.5065	0.0014	1	221	228	ALQYDFTR	32	99.605			Mascot
1033.4989	1033.5043	0.0054	5	69	76	NDFYLFSK					Mascot
1189.6001	1189.6073	0.0072	6	68	76	RNDFYLFSK					Mascot
1189.6001	1189.6073	0.0072	6	68	76	RNDFYLFSK	45	99.98			Mascot
1254.6841	1254.6875	0.0034	3	219	228	LKALQYDFTR					Mascot
1299.7379	1299.7426	0.0047	4	229	240	RPTTEAVSVALR					Mascot
1299.7379	1299.7426	0.0047	4	229	240	RPTTEAVSVALR	31	99.528			Mascot
1325.6808	1325.693	0.0122	9	56	67	SEELIGQAVSHR					Mascot
1325.6808	1325.693	0.0122	9	56	67	SEELIGQAVSHR					Mascot
1481.782	1481.7914	0.0094	6	56	68	SEELIGQAVSHRR					Mascot
1774.9083	1774.9087	0.0004	0	13	30	VSALGFGGAEIGQQVDAR					Mascot
1774.9083	1774.9087	0.0004	0	13	30	VSALGFGGAEIGQQVDAR	140	100			Mascot
1988.0812	1988.0806	-0.0006	0	241	260	FTLSVPGVTTAIVGTTNPGR					Mascot
1988.0812	1988.0806	-0.0006	0	241	260	FTLSVPGVTTAIVGTTNPGR	145	100			Mascot
2009.9498	2009.9496	-0.0002	0	77	94	CGHASGLPTPDWDVQTLR			Carbamidomethyl (C)[1]		Mascot
2009.9498	2009.9496	-0.0002	0	77	94	CGHASGLPTPDWDVQTLR	5	0	Carbamidomethyl (C)[1]		Mascot
2547.2109	2547.2146	0.0037	1	261	282	WQQNADLVNQGPLDTTEYDAIR					Mascot
2577.2979	2577.3071	0.0092	4	31	55	QVDALLGSALDAGLNLIDTAACYGR			Carbamidomethyl (C)[22]		Mascot
2577.2979	2577.3071	0.0092	4	31	55	QVDALLGSALDAGLNLIDTAACYGR	24	97.652	Carbamidomethyl (C)[22]		Mascot
3061.4592	3061.4819	0.0227	7	194	218	RPIANVAWQYADTPDNAYYVTYWQR					Mascot

Rank	Protein Name	Species	Accession No.	Protein MW	Protein PI	Pep. Count	Protein Score	Protein Score C. I. %	Total Ion Score	Total Ion C. I. %
2	Uncharacterized protein OS=Alicyclobacillus		tr|T0BAG0|T0BAG	32141.6	6.06	8	31	0		

	acidoterrestris (strain ATCC 49025 / DSM 3922 / CIP 106132 / NCIMB 13137 / GD3B) GN=N007_17435 PE=4 SV=1	0_ALIAG					

Peptide Information

Calc. Mass	Obsrv. Mass	± da	± ppm	Start Seq.	End Seq.	Sequence	Ion Score	C. I. %	Modification	Rank	Result Type
1254.6324	1254.6875	0.0551	44	234	244	ADLHVQSEIDK					Mascot
1278.6953	1278.6329	-0.0624	-49	39	48	VFEHIHLEVR					Mascot
1338.7489	1338.6941	-0.0548	-41	50	63	GDRIGIVGPNGVGK					Mascot
1788.9524	1788.9254	-0.027	-15	131	146	KEDVLKPISSLSMGER					Mascot
1788.9524	1788.9254	-0.027	-15	131	146	KEDVLKPISSLSMGER					Mascot
1804.9474	1804.8958	-0.0516	-29	131	146	KEDVLKPISSLSMGER			Oxidation (M)[13]		Mascot
1973.8844	1974.0284	0.144	73	217	233	SFDGTMEAFVSQMHSRK			Oxidation (M)[6]		Mascot
1992.9841	1992.9391	-0.045	-23	132	148	EDVLKPISSLSMGERCR			Carbamidomethyl (C)[16], Oxidation (M)[12]		Mascot
2098.1001	2097.9458	-0.1543	-74	179	197	LEEALLAYPGALMIVSHD R					Mascot
2577.2053	2577.3071	0.1018	40	1	22	MERPHHEPNIGVSFQHN AFDAK			Oxidation (M)[1]		Mascot
2577.2053	2577.3071	0.1018	40	1	22	MERPHHEPNIGVSFQHN AFDAK			Oxidation (M)[1]		Mascot

3	Uncharacterized protein OS=Alicyclobacillus acidoterrestris (strain ATCC 49025 / DSM 3922 / CIP 106132 / NCIMB 13137 / GD3B) GN=N007_07030 PE=4 SV=1	tr|T0C0U9|T0C0U 9_ALIAG	17080	4.53	6	30	0

Peptide Information

Calc. Mass	Obsrv. Mass	± da	± ppm	Start Seq.	End Seq.	Sequence	Ion Score	C. I. %	Modification	Rank	Result Type
1033.4546	1033.5043	0.0497	48	66	75	GQSEVGDDAR					Mascot
1189.5557	1189.6073	0.0516	43	66	76	GQSEVGDDARR					Mascot
1189.5557	1189.6073	0.0516	43	66	76	GQSEVGDDARR					Mascot
1213.5374	1213.5745	0.0371	31	1	10	MAELQCVTMR			Oxidation (M)[1,9]		Mascot
1254.564	1254.6875	0.1235	98	1	10	MAELQCVTMR			Carbamidomethyl (C)[6], Oxidation (M)[1]		Mascot
1748.9615	1748.8766	-0.0849	-49	29	43	MLDISPVPRTLSYIK			Oxidation (M)[1]		Mascot
2024.9893	2024.9106	-0.0787	-39	2	19	AELQCVTMRIGDELYGA R					Mascot
2040.9841	2040.9177	-0.0664	-33	2	19	AELQCVTMRIGDELYGA R			Oxidation (M)[8]		Mascot
2051.0227	2050.9973	-0.0254	-12	11	28	IGDELYGARVEQVMSVE R					Mascot
2082.0107	2081.9675	-0.0432	-21	2	19	AELQCVTMRIGDELYGA R			Carbamidomethyl (C)[5]		Mascot
2098.0056	2097.9458	-0.0598	-29	2	19	AELQCVTMRIGDELYGA R			Carbamidomethyl (C)[5], Oxidation (M)[8]		Mascot

4	Uncharacterized protein OS=Alicyclobacillus acidoterrestris (strain ATCC 49025 / DSM 3922 / CIP 106132 / NCIMB 13137 / GD3B) GN=N007_06755 PE=4 SV=1	tr|T0D9G5|T0D9G5_ALIAG	27568.4	5.31	7	28	0

Peptide Information

Calc. Mass	Obsrv. Mass	± da	± ppm	Start Seq.	End Seq.	Sequence	Ion Score	C. I. %	Modification	Rank	Result Type
828.5302	828.5398	0.0096	12	118	124	ELLARVK					Mascot
1641.7426	1641.856	0.1134	69	218	231	LESDASQPEYVMTR			Oxidation (M)[12]		Mascot
1781.8488	1781.9154	0.0666	37	218	232	LESDASQPEYVMTRR					Mascot
2013.1379	2012.9592	-0.1787	-89	144	160	LVIRDLVVDLTHYEVTK					Mascot
2041.8807	2041.9386	0.0579	28	189	205	EQLVSEVWGMDYEGDER					Mascot
2081.0913	2080.9392	-0.1521	-73	99	117	VLGLELGADDYVTKPFSTR					Mascot
2577.3672	2577.3071	-0.0601	-23	148	170	DLVVDLTHYEVTKAGEPIALTHR					Mascot
2577.3672	2577.3071	-0.0601	-23	148	170	DLVVDLTHYEVTKAGEPIALTHR					Mascot

5	DNA-directed RNA polymerase subunit omega OS=Alicyclobacillus acidoterrestris (strain ATCC 49025 / DSM 3922 / CIP 106132 / NCIMB 13137 / GD3B) GN=rpoZ PE=3 SV=1	tr|T0CFH0|T0CFH0_ALIAG	7700	8.04	4	28	0

Peptide Information

Calc. Mass	Obsrv. Mass	± da	± ppm	Start Seq.	End Seq.	Sequence	Ion Score	C. I. %	Modification	Rank	Result Type
1780.8495	1780.9412	0.0917	51	29	44	QLQTETMNQVGSSTTR					Mascot
1867.9722	1867.9218	-0.0504	-27	1	16	MLYPSIDKLIDLTDSK			Oxidation (M)[1]		Mascot
2007.9877	2007.9333	-0.0544	-27	27	44	ARQLQTETMNQVGSSTTR					Mascot
2023.9825	2023.9426	-0.0399	-20	27	44	ARQLQTETMNQVGSSTTR			Oxidation (M)[9]		Mascot
2037.04	2036.9354	-0.1046	-51	45	62	NVSRALWEIYNGDVATTK					Mascot

6	Uncharacterized protein OS=Alicyclobacillus acidoterrestris (strain ATCC 49025 / DSM 3922 / CIP 106132 / NCIMB 13137 / GD3B) GN=N007_06480 PE=4 SV=1	tr|T0C2W1|T0C2W1_ALIAG	19495.9	7.77	6	27	0

Peptide Information

Calc. Mass	Obsrv. Mass	± da	± ppm	Start Seq.	End Seq.	Sequence	Ion Score	C. I. %	Modification	Rank	Result Type
1812.8625	1812.8721	0.0096	5	7	21	ADFYYGSMLSYLINR					Mascot
1944.9406	1945.0647	0.1241	64	154	169	VSRDALTEYMLMENIK			Oxidation (M)[10,12]		Mascot

Calc. Mass	Obsrv. Mass	± da	± ppm	Start Seq.	End Seq.	Sequence	Ion Score	C. I. %	Modification	Rank	Result Type
1968.9636	1969.0088	0.0452	23	6	21	RADFYYGSMLSYLINR					Mascot
1984.0975	1984.0813	-0.0162	-8	135	153	GLHGLKVYGTGISDINAIR					Mascot
2013.0189	2012.9592	-0.0597	-30	35	51	RIYSLSTNHGDFQIYAK					Mascot
2039.0233	2038.938	-0.0853	-42	60	77	QGSGVKIWNFSFTPDEVK					Mascot

7	4-hydroxy-3-methylbut-2-en-1-yl diphosphate synthase (flavodoxin) OS=Alicyclobacillus acidoterrestris (strain ATCC 49025 / DSM 3922 / CIP 106132 / NCIMB 13137 / GD3B) GN=ispG PE=3 SV=1	tr|T0BS43|T0BS43_ALIAG	39371.7	6.12	9	26	0

Peptide Information

Calc. Mass	Obsrv. Mass	± da	± ppm	Start Seq.	End Seq.	Sequence	Ion Score	C. I. %	Modification	Rank	Result Type
812.4625	812.532	0.0695	86	102	109	INPGNIGK					Mascot
828.5302	828.5398	0.0096	12	245	251	VARELLK					Mascot
1013.585	1013.5065	-0.0785	-77	4	11	REQTKPVR					Mascot
1013.585	1013.5065	-0.0785	-77	4	11	REQTKPVR					Mascot
1781.8884	1781.9154	0.027	15	215	232	SSAGLGTLLSMGIGNTMR			Oxidation (M)[11]		Mascot
1788.9274	1788.9254	-0.002	-1	46	61	LEDAGCQVVRVTVNTK			Carbamidomethyl (C)[6]		Mascot
1788.9274	1788.9254	-0.002	-1	46	61	LEDAGCQVVRVTVNTK			Carbamidomethyl (C)[6]		Mascot
1836.9756	1836.8185	-0.1571	-86	73	87	QIHIPLVADIHFDYR					Mascot
1993.0767	1992.9391	-0.1376	-69	72	87	RQIHIPLVADIHFDYR					Mascot
2010.0186	2009.9496	-0.069	-34	38	55	GTVEQIHRLEDAGCQVVR					Mascot
2010.0186	2009.9496	-0.069	-34	38	55	GTVEQIHRLEDAGCQVVR					Mascot
2034.0907	2033.8915	-0.1992	-98	196	214	YPLHLGITESGTLFSGTIK					Mascot

8	Uncharacterized protein OS=Alicyclobacillus acidoterrestris (strain ATCC 49025 / DSM 3922 / CIP 106132 / NCIMB 13137 / GD3B) GN=N007_02645 PE=4 SV=1	tr|T0CIS0|T0CIS0_ALIAG	13958.3	9.02	6	26	0

Peptide Information

Calc. Mass	Obsrv. Mass	± da	± ppm	Start Seq.	End Seq.	Sequence	Ion Score	C. I. %	Modification	Rank	Result Type
1033.4296	1033.5043	0.0747	72	3	10	TYEFNCEK					Mascot
1323.7015	1323.7091	0.0076	6	58	68	ELENDGIIHRK					Mascot
1349.5864	1349.6512	0.0648	48	1	10	MKTYEFNCEK			Carbamidomethyl (C)[8]		Mascot
1827.8694	1827.8661	-0.0033	-2	101	116	SYARDMNIDISGWTAK					Mascot
1993.0841	1992.9391	-0.145	-73	21	36	WKMIILWHIGLDSPQR					Mascot
2009.079	2008.9252	-0.1538	-77	21	36	WKMIILWHIGLDSPQR			Oxidation (M)[3]		Mascot

Calc. Mass	Obsrv. Mass	± da	± ppm	Start Seq.	End Seq.	Sequence	Ion Score	C. I. %	Modification	Rank	Result Type
2025.0546	2024.9106	-0.144	-71	51	67	MLTSQLRELENDGIIHR					Mascot
2041.0496	2040.9177	-0.1319	-65	51	67	MLTSQLRELENDGIIHR			Oxidation (M)[1]		Mascot

9	Succinate--CoA ligase [ADP-forming] subunit alpha OS=Alicyclobacillus acidoterrestris (strain ATCC 49025 / DSM 3922 / CIP 106132 / NCIMB 13137 / GD3B) GN=sucD PE=3 SV=1	tr|T0C0Z3|T0C0Z3_ALIAG	31636.3	5.37	7	25	0

Peptide Information

Calc. Mass	Obsrv. Mass	± da	± ppm	Start Seq.	End Seq.	Sequence	Ion Score	C. I. %	Modification	Rank	Result Type
1037.4867	1037.4719	-0.0148	-14	265	272	KMQECGIR			Carbamidomethyl (C)[5], Oxidation (M)[2]		Mascot
1756.876	1756.9019	0.0259	15	244	262	RMGHAGAIVSGGAGTAESK					Mascot
1988.0812	1988.0806	-0.0006	0	8	26	DTKVITQGITGATGLFHTK					Mascot
1988.0812	1988.0806	-0.0006	0	8	26	DTKVITQGITGATGLFHTK					Mascot
2034.0212	2033.8915	-0.1297	-64	273	291	VAPTPSEMGATLYAVIEER					Mascot
2041.09	2040.9177	-0.1723	-84	224	243	ENMTKPVVGFIAGVTAPPGR					Mascot
2197.1912	2197.2644	0.0732	33	224	244	ENMTKPVVGFIAGVTAPPGRR					Mascot
2560.3328	2560.2542	-0.0786	-31	273	296	VAPTPSEMGATLYAVIEERGLLDK					Mascot

10	Uncharacterized protein OS=Alicyclobacillus acidoterrestris (strain ATCC 49025 / DSM 3922 / CIP 106132 / NCIMB 13137 / GD3B) GN=N007_04190 PE=4 SV=1	tr|T0DHP6|T0DHP6_ALIAG	6929.4	6.81	4	25	0

Peptide Information

Calc. Mass	Obsrv. Mass	± da	± ppm	Start Seq.	End Seq.	Sequence	Ion Score	C. I. %	Modification	Rank	Result Type
1178.5735	1178.5759	0.0024	2	2	12	ASSQHAIHCPK					Mascot
1325.6089	1325.693	0.0841	63	1	12	MASSQHAIHCPK			Oxidation (M)[1]		Mascot
1325.6089	1325.693	0.0841	63	1	12	MASSQHAIHCPK			Oxidation (M)[1]		Mascot
1973.9069	1974.0284	0.1215	62	2	19	ASSQHAIHCPKCNSTSFR					Mascot
2065.1765	2064.9846	-0.1919	-93	23	41	IVQLDSSVVIRDGLPVQAR					Mascot

Gel Idx/Pos	234/J10	Instr./Gel Origin	BA2151/full sequence test 20150515	Process Status	Analysis Succeeded
Plate [#] Name	[3] full sequence test 20150515	Instrument Sample Name		Spectra	11

Rank	Protein Name	Species	Accession No.	Protein MW	Protein PI	Pep. Count	Protein Score	Protein Score C. I. %	Total Ion Score	Total Ion C. I. %
1	Succinate--CoA ligase [ADP-forming] subunit alpha OS=Alicyclobacillus acidoterrestris (strain ATCC 49025 / DSM 3922 / CIP 106132 / NCIMB 13137 / GD3B) GN=sucD PE=3 SV=1		tr|T0C0Z3|T0C0Z3_ALIAG	31636.3	5.37	15	469	100	381	100

Peptide Information

Calc. Mass	Obsrv. Mass	± da	± ppm	Start Seq.	End Seq.	Sequence	Ion Score	C. I. %	Modification	Rank	Result Type
909.3917	909.3997	0.008	9	266	272	MQECGIR			Carbamidomethyl (C)[4], Oxidation (M)[1]		Mascot
1021.5565	1021.5464	-0.0101	-10	185	193	GTEFIDVLK					Mascot
1037.4867	1037.4923	0.0056	5	265	272	KMQECGIR			Carbamidomethyl (C)[5], Oxidation (M)[2]		Mascot
1410.7926	1410.7875	-0.0051	-4	135	147	IGIMPGYIHKPGK					Mascot
1426.7876	1426.7802	-0.0074	-5	135	147	IGIMPGYIHKPGK			Oxidation (M)[4]		Mascot
1426.7876	1426.7802	-0.0074	-5	135	147	IGIMPGYIHKPGK	28	99.019	Oxidation (M)[4]		Mascot
1483.8115	1483.8107	-0.0008	-1	169	184	GIGQSTVVGIGGDPVK					Mascot
1616.7698	1616.7733	0.0035	2	245	262	MGHAGAIVSGGAGTAES K			Oxidation (M)[1]		Mascot
1646.8021	1646.7939	-0.0082	-5	154	168	SGTLTYEAAYQLSSR					Mascot
1646.8021	1646.7939	-0.0082	-5	154	168	SGTLTYEAAYQLSSR	129	100			Mascot
1711.8507	1711.8431	-0.0076	-4	119	134	LIGPNCPGVITPGECK			Carbamidomethyl (C)[6,15]		Mascot
1711.8507	1711.8431	-0.0076	-4	119	134	LIGPNCPGVITPGECK	30	99.433	Carbamidomethyl (C)[6,15]		Mascot
1988.0812	1988.0613	-0.0199	-10	8	26	DTKVITQGITGATGLFHTK					Mascot
1992.0154	1992.0836	0.0682	34	116	134	HTRLIGPNCPGVITPGEC K					Mascot
2034.0212	2034.0177	-0.0035	-2	273	291	VAPTPSEMGATLYAVIEE R					Mascot
2034.0212	2034.0177	-0.0035	-2	273	291	VAPTPSEMGATLYAVIEE R	53	99.997			Mascot
2038.1631	2038.0325	-0.1306	-64	135	153	IGIMPGYIHKPGKVGVISR			Oxidation (M)[4]		Mascot
2041.09	2041.0759	-0.0141	-7	224	243	ENMTKPVVGFIAGVTAPP GR					Mascot
2050.0161	2050.0044	-0.0117	-6	273	291	VAPTPSEMGATLYAVIEE R			Oxidation (M)[8]		Mascot
2050.0161	2050.0044	-0.0117	-6	273	291	VAPTPSEMGATLYAVIEE R	67	100	Oxidation (M)[8]		Mascot
2057.085	2057.0735	-0.0115	-6	224	243	ENMTKPVVGFIAGVTAPP GR			Oxidation (M)[3]		Mascot
2213.186	2213.1653	-0.0207	-9	224	244	ENMTKPVVGFIAGVTAPP GRR			Oxidation (M)[3]		Mascot
2213.186	2213.1653	-0.0207	-9	224	244	ENMTKPVVGFIAGVTAPP GRR	8	6.721	Oxidation (M)[3]		Mascot

Calc. Mass	Obsrv. Mass	± da	± ppm	Start Seq.	End Seq.	Sequence	Ion Score	C. I. %	Modification	Rank	Result Type
2486.3501	2486.3438	-0.0063	-3	169	193	GIGQSTVVGIGGDPVKGTEFIDVLK					Mascot
2486.3501	2486.3438	-0.0063	-3	169	193	GIGQSTVVGIGGDPVKGTEFIDVLK	121	100			Mascot

2	Uncharacterized protein OS=Alicyclobacillus acidoterrestris (strain ATCC 49025 / DSM 3922 / CIP 106132 / NCIMB 13137 / GD3B) GN=N007_05765 PE=4 SV=1	tr|T0C5D6|T0C5D6_ALIAG	20521.5	5.65	9	45	86.417

Peptide Information

Calc. Mass	Obsrv. Mass	± da	± ppm	Start Seq.	End Seq.	Sequence	Ion Score	C. I. %	Modification	Rank	Result Type
1253.5765	1253.5851	0.0086	7	60	69	VYQHIGMSMR			Oxidation (M)[7,9]		Mascot
1253.5765	1253.5851	0.0086	7	60	69	VYQHIGMSMR			Oxidation (M)[7,9]		Mascot
1381.6715	1381.6692	-0.0023	-2	59	69	KVYQHIGMSMR			Oxidation (M)[8,10]		Mascot
1424.7931	1424.791	-0.0021	-1	1	15	MIPNIAIVGPAGSGK					Mascot
1595.7952	1595.8481	0.0529	33	70	82	EIDADVWIKYTDK					Mascot
1646.865	1646.7939	-0.0711	-43	111	124	GWYTVRIVAPDDVR					Mascot
1646.865	1646.7939	-0.0711	-43	111	124	GWYTVRIVAPDDVR					Mascot
1774.9083	1774.8983	-0.01	-6	21	35	YIQQVLQSHSVTSER					Mascot
1939.1045	1939.052	-0.0525	-27	1	20	MIPNIAIVGPAGSGKTTLAK					Mascot
2066.9788	2067.0198	0.041	20	83	99	RISDYPCTVHFVLDDMR					Mascot
2180.0627	2179.9963	-0.0664	-30	84	101	ISDYPCTVHFVLDDMRLR					Mascot

3	Adenylate kinase OS=Alicyclobacillus acidoterrestris (strain ATCC 49025 / DSM 3922 / CIP 106132 / NCIMB 13137 / GD3B) GN=adk PE=3 SV=1	tr|T0D2X0|T0D2X0_ALIAG	24183.4	5.6	8	37	25.356

Peptide Information

Calc. Mass	Obsrv. Mass	± da	± ppm	Start Seq.	End Seq.	Sequence	Ion Score	C. I. %	Modification	Rank	Result Type
925.4196	925.3933	-0.0263	-28	153	160	CGGELYQR					Mascot
947.468	947.4241	-0.0439	-46	161	169	ADDTVEAVK					Mascot
1021.5465	1021.5464	-0.0001	0	80	88	GFLLDGFPR					Mascot
1749.8555	1749.8257	-0.0298	-17	74	88	EEDAQRGFLLDGFPR					Mascot
2006.0277	2006.0078	-0.0199	-10	20	37	ITASFGIPHISTGDMFRR					Mascot
2039.0404	2039.0637	0.0233	11	38	57	AVASGTELGNTLKSYLDSGR					Mascot
2180.0884	2179.9963	-0.0921	-42	172	188	LEQYQHTAPLIDYYRNR					Mascot
2508.23	2508.3269	0.0969	39	14	36	GTQAERITASFGIPHISTGDMFR			Oxidation (M)[21]		Mascot

4	Uncharacterized protein OS=Alicyclobacillus acidoterrestris (strain ATCC 49025 / DSM 3922 / CIP	tr|T0CSF3|T0CSF3_ALIAG	8314.7	12.6	5	35	0

106132 / NCIMB 13137 / GD3B) GN=N007_15165
PE=4 SV=1

Peptide Information

Calc. Mass	Obsrv. Mass	± da	± ppm	Start Seq.	End Seq.	Sequence	Ion Score	C. I. %	Modification	Rank	Result Type
1387.7727	1387.6764	-0.0963	-69	1	13	MVTAVQLLAGQTR					Mascot
1426.7438	1426.7802	0.0364	26	40	52	GFNNTVFVKSGTR					Mascot
1426.7438	1426.7802	0.0364	26	40	52	GFNNTVFVKSGTR					Mascot
1667.0327	1666.9443	-0.0884	-53	57	71	TRIINLAIGQTVIVR					Mascot
1718.8755	1718.8264	-0.0491	-29	14	28	NFTRGHATMNTITVR					Mascot
1774.9923	1774.8983	-0.094	-53	2	17	VTAVQLLAGQTRNFTR					Mascot

5 Uncharacterized protein OS=Alicyclobacillus acidoterrestris (strain ATCC 49025 / DSM 3922 / CIP 106132 / NCIMB 13137 / GD3B) GN=N007_11175 PE=4 SV=1   tr|T0BTY1|T0BTY1_ALIAG   62277.7   6.76   12   33   0

Peptide Information

Calc. Mass	Obsrv. Mass	± da	± ppm	Start Seq.	End Seq.	Sequence	Ion Score	C. I. %	Modification	Rank	Result Type
1037.5262	1037.4923	-0.0339	-33	237	245	ILSDFSNNK					Mascot
1278.7052	1278.7052	0	0	237	247	ILSDFSNNKLK					Mascot
1602.8381	1602.7997	-0.0384	-24	530	542	MVLSIREQAQQQR			Oxidation (M)[1]		Mascot
1616.8656	1616.7733	-0.0923	-57	373	385	FIEHVNLFANRTR					Mascot
1684.8918	1684.7623	-0.1295	-77	469	481	QEHLLELYHIPHR					Mascot
1691.9229	1691.7949	-0.128	-76	85	99	AEGAFRILQDLGVFR					Mascot
1812.8256	1812.9305	0.1049	58	348	362	QTMMTGESVTEWLQR			Oxidation (M)[3]		Mascot
1938.8828	1939.052	0.1692	87	115	131	SDGSQIGDVFEHVTYER					Mascot
2022.9702	2023.0566	0.0864	43	68	84	SIPEPGLIIYDECHHSR			Carbamidomethyl (C)[13]		Mascot
2026.9611	2027.0701	0.109	54	217	236	LAGYAAASVDGSMSAEQ RAR			Oxidation (M)[13]		Mascot
2032.0685	2032.0051	-0.0634	-31	5	21	FYQEVCSEKPILVLAHR					Mascot
2524.3843	2524.3086	-0.0757	-30	44	67	VQGARNEQLAEVILASTQ TLVAGR					Mascot

6 Uncharacterized protein OS=Alicyclobacillus acidoterrestris (strain ATCC 49025 / DSM 3922 / CIP 106132 / NCIMB 13137 / GD3B) GN=N007_15300 PE=4 SV=1   tr|T0BNR2|T0BNR2_ALIAG   57489.2   5.25   11   31   0

Peptide Information

Calc. Mass	Obsrv. Mass	± da	± ppm	Start Seq.	End Seq.	Sequence	Ion Score	C. I. %	Modification	Rank	Result Type

Calc. Mass	Obsrv. Mass	± da	± ppm	Start Seq.	End Seq.	Sequence	Ion Score	C. I. %	Modification	Rank	Result Type
1140.6848	1140.5984	-0.0864	-76	507	517	AGAILDVLRGR					Mascot
1266.6477	1266.7299	0.0822	65	314	324	LYGASPEVQFR					Mascot
1448.7301	1448.7562	0.0261	18	438	449	AMEIIDELETLR			Oxidation (M)[2]		Mascot
1450.7723	1450.759	-0.0133	-9	208	220	IDEFASMLARVAK					Mascot
1502.7672	1502.7372	-0.03	-20	1	13	MKVQSYTYANVAK					Mascot
1602.8268	1602.7997	-0.0271	-17	325	340	ALNGSAEMKPIAGTSR					Mascot
2005.9913	2006.0078	0.0165	8	499	515	EWEECHHKAGAILDVLR					Mascot
2056.9902	2057.0735	0.0833	40	362	379	ESAEHVMLVDLCRNDLG R					Mascot
2213.1172	2213.1653	0.0481	22	101	118	SSRYFRPVDPMAFLEEL R					Mascot
2213.1172	2213.1653	0.0481	22	101	118	SSRYFRPVDPMAFLEEL R					Mascot
2486.228	2486.3438	0.1158	47	375	396	NDLGRVCLSGSVHVPDF MVVER			Carbamidomethyl (C)[7]		Mascot
2486.228	2486.3438	0.1158	47	375	396	NDLGRVCLSGSVHVPDF MVVER			Carbamidomethyl (C)[7]		Mascot
2508.1785	2508.3269	0.1484	59	195	217	DQEDGAGNSLVNRIDEF ASMLAR					Mascot
2524.1733	2524.3086	0.1353	54	195	217	DQEDGAGNSLVNRIDEF ASMLAR			Oxidation (M)[20]		Mascot

7	Uncharacterized protein OS=Alicyclobacillus acidoterrestris (strain ATCC 49025 / DSM 3922 / CIP 106132 / NCIMB 13137 / GD3B) GN=N007_17535 PE=4 SV=1	tr|T0BAK4|T0BAK 4_ALIAG	11290.7	5.28	5	30	0

Peptide Information

Calc. Mass	Obsrv. Mass	± da	± ppm	Start Seq.	End Seq.	Sequence	Ion Score	C. I. %	Modification	Rank	Result Type
1993.0432	1993.0544	0.0112	6	1	19	MLVGITIALVGCSMAPFN R					Mascot
2009.0381	2009.0232	-0.0149	-7	1	19	MLVGITIALVGCSMAPFN R			Oxidation (M)[1]		Mascot
2034.0988	2034.0177	-0.0811	-40	2	20	LVGITIALVGCSMAPFNR R			Oxidation (M)[13]		Mascot
2034.0988	2034.0177	-0.0811	-40	2	20	LVGITIALVGCSMAPFNR R			Oxidation (M)[13]		Mascot
2050.0647	2050.0044	-0.0603	-29	1	19	MLVGITIALVGCSMAPFN R			Carbamidomethyl (C)[12]		Mascot
2050.0647	2050.0044	-0.0603	-29	1	19	MLVGITIALVGCSMAPFN R			Carbamidomethyl (C)[12]		Mascot
2079.1194	2079.0469	-0.0725	-35	83	100	LIPMEALFAESTLERFIV					Mascot
2149.1443	2149.1311	-0.0132	-6	1	20	MLVGITIALVGCSMAPFN RR					Mascot
2213.1196	2213.1653	0.0457	21	63	82	NQATLTAAVQYSYEQGLI SR					Mascot
2213.1196	2213.1653	0.0457	21	63	82	NQATLTAAVQYSYEQGLI SR					Mascot

8	Uncharacterized protein OS=Alicyclobacillus acidoterrestris (strain ATCC 49025 / DSM 3922 / CIP 106132 / NCIMB 13137 / GD3B) GN=N007_16405	tr|T0BMU7|T0BMU 7_ALIAG	33316.4	9.22	8	30	0

PE=4 SV=1

Peptide Information

Calc. Mass	Obsrv. Mass	± da	± ppm	Start Seq.	End Seq.	Sequence	Ion Score	C. I. %	Modification	Rank	Result Type
1228.5627	1228.6437	0.081	66	1	10	MSQTDYLTNR					Mascot
1464.8058	1464.7351	-0.0707	-48	223	235	QVTGLTINQYVTK					Mascot
1668.8275	1668.7793	-0.0482	-29	270	283	MFRQQVGVSPNAYR			Oxidation (M)[1]		Mascot
1733.9908	1733.829	-0.1618	-93	223	237	QVTGLTINQYVTKLR					Mascot
1992.1389	1992.0836	-0.0553	-28	70	86	GEIVHLVKPSERVPYLR					Mascot
2079.054	2079.0469	-0.0071	-3	87	105	SILNFREDACGSLDALVQK					Mascot
2486.3623	2486.3438	-0.0185	-7	144	164	EVGYQDMMHALLTVLVLRIIR			Oxidation (M)[7]		Mascot
2486.3623	2486.3438	-0.0185	-7	144	164	EVGYQDMMHALLTVLVLRIIR			Oxidation (M)[7]		Mascot
2578.2783	2578.2412	-0.0371	-14	2	22	SQTDYLTNRILTEDTVYVQYR					Mascot

9	Uncharacterized protein OS=Alicyclobacillus acidoterrestris (strain ATCC 49025 / DSM 3922 / CIP 106132 / NCIMB 13137 / GD3B) GN=N007_06270 PE=4 SV=1	tr|T0BTL1|T0BTL1_ALIAG	5673	9.39	4	28	0

Peptide Information

Calc. Mass	Obsrv. Mass	± da	± ppm	Start Seq.	End Seq.	Sequence	Ion Score	C. I. %	Modification	Rank	Result Type
1381.7549	1381.6692	-0.0857	-62	8	19	WSIATVIFGMLK			Oxidation (M)[10]		Mascot
1668.8275	1668.7793	-0.0482	-29	28	41	QPFAIRDAPMHDVR			Oxidation (M)[10]		Mascot
1939.0721	1939.052	-0.0201	-10	2	19	TISAGKWSIATVIFGMLK			Oxidation (M)[16]		Mascot
2044.9944	2045.0763	0.0819	40	34	50	DAPMHDVRMDVKPFINK			Oxidation (M)[4,9]		Mascot

10	Uncharacterized protein OS=Alicyclobacillus acidoterrestris (strain ATCC 49025 / DSM 3922 / CIP 106132 / NCIMB 13137 / GD3B) GN=N007_14240 PE=4 SV=1	tr|T0CYZ2|T0CYZ2_ALIAG	18341.3	5.32	6	28	0

Peptide Information

Calc. Mass	Obsrv. Mass	± da	± ppm	Start Seq.	End Seq.	Sequence	Ion Score	C. I. %	Modification	Rank	Result Type
1013.5989	1013.501	-0.0979	-97	80	88	ALIEELVAR					Mascot
1278.5995	1278.7052	0.1057	83	33	43	NEVSVMDDTIR					Mascot
1668.8448	1668.7793	-0.0655	-39	45	59	MVDLGVGLMSFSREK					Mascot
1684.8397	1684.7623	-0.0774	-46	45	59	MVDLGVGLMSFSREK			Oxidation (M)[1]		Mascot
1992.0985	1992.0836	-0.0149	-7	111	128	LGLREQTAGLEHQLASIR					Mascot

2027.0516	2027.0701	0.0185	9	94	110	TELQNNIQDQVQKALER		Mascot
2081.0234	2081.0369	0.0135	6	15	32	QLHVSAMEVVHYGEVNP R	Oxidation (M)[7]	Mascot

Gel Idx/Pos	235/J11	Instr./Gel Origin	BA2151/full sequence test 20150515	Process Status	Analysis Succeeded
Plate [#] Name	[3] full sequence test 20150515	Instrument Sample Name		Spectra	11

Rank	Protein Name	Species	Accession No.	Protein MW	Protein PI	Pep. Count	Protein Score	Protein Score C. I. %	Total Ion Score	Total Ion C. I. %
1	Polyamine aminopropyltransferase OS=Alicyclobacillus acidoterrestris (strain ATCC 49025 / DSM 3922 / CIP 106132 / NCIMB 13137 / GD3B) GN=speE PE=3 SV=1		tr|T0DMQ2|T0DMQ2_ALIAG	31094.8	5.52	16	308	100	205	100

Peptide Information

Calc. Mass	Obsrv. Mass	± da	± ppm	Start Seq.	End Seq.	Sequence	Ion Score	C. I. %	Modification	Rank	Result Type
947.4622	947.4686	0.0064	7	174	180	FYESVFR					Mascot
947.4622	947.4686	0.0064	7	174	180	FYESVFR	14	78.942			Mascot
1075.5571	1075.5626	0.0055	5	173	180	KFYESVFR					Mascot
1075.5571	1075.5626	0.0055	5	173	180	KFYESVFR	34	99.781			Mascot
1085.6201	1085.6252	0.0051	5	134	143	VDVQIVDGIK					Mascot
1193.6637	1193.6648	0.0011	1	95	104	EVIKHPSVER					Mascot
1325.7537	1325.7596	0.0059	4	81	94	NVLVVGGGDGGIIR					Mascot
1325.7537	1325.7596	0.0059	4	81	94	NVLVVGGGDGGIIR	31	99.542			Mascot
1395.691	1395.6786	-0.0124	-9	239	249	VHRPQDVQTCR			Carbamidomethyl (C)[10]		Mascot
1434.7377	1434.7361	-0.0016	-1	121	133	YFPQIAAGLSDPR	28	99.124			Mascot
1562.8325	1562.8314	-0.0011	-1	120	133	KYFPQIAAGLSDPR					Mascot
1599.8953	1599.8925	-0.0028	-2	105	120	AVLAEIDGAVVEASKK					Mascot
2086.0869	2086.0791	-0.0078	-4	255	272	YYNAQVHHASLALPQFVK					Mascot
2086.0869	2086.0791	-0.0078	-4	255	272	YYNAQVHHASLALPQFVK	97	100			Mascot
2124.0496	2124.0371	-0.0125	-6	25	42	TLHSEVTPYQTLDVYETK					Mascot
2359.2041	2359.1965	-0.0076	-3	2	21	PQSNLWFTELQNENLSIGLR					Mascot
2359.2041	2359.1965	-0.0076	-3	2	21	PQSNLWFTELQNENLSIGLR					Mascot
2501.3398	2501.3281	-0.0117	-5	121	143	YFPQIAAGLSDPRVDVQIVDGIK					Mascot
2664.2756	2664.2739	-0.0017	-1	184	207	SDGVMAAQTESPFVNQELIQNVMR					Mascot
2680.2705	2680.2791	0.0086	3	184	207	SDGVMAAQTESPFVNQELIQNVMR			Oxidation (M)[5]		Mascot
2976.4917	2976.5059	0.0142	5	181	207	ALKSDGVMAAQTESPFVNQELIQNVMR					Mascot
2992.4866	2992.4902	0.0036	1	181	207	ALKSDGVMAAQTESPFVNQELIQNVMR			Oxidation (M)[8]		Mascot
3117.5212	3117.5225	0.0013	0	211	238	GTFPISQLYLAHVPTYPTGMWSFTMGSK					Mascot
3133.5164	3133.522	0.0056	2	211	238	GTFPISQLYLAHVPTYPT			Oxidation (M)[20]		Mascot

GMWSFTMGSK

2	Uncharacterized protein OS=Alicyclobacillus acidoterrestris (strain ATCC 49025 / DSM 3922 / CIP 106132 / NCIMB 13137 / GD3B) GN=N007_05370 PE=4 SV=1	tr|T0C561|T0C561_ALIAG	9137.4	6.06	6	33	0

Peptide Information

Calc. Mass	Obsrv. Mass	± da	± ppm	Start Seq.	End Seq.	Sequence	Ion Score	C. I. %	Modification	Rank	Result Type
971.4477	971.4258	-0.0219	-23	34	40	YMAWICK			Carbamidomethyl (C)[6]		Mascot
1349.6155	1349.7207	0.1052	78	2	12	STVPPFEECQR			Carbamidomethyl (C)[9]		Mascot
1419.6218	1419.6639	0.0421	30	34	43	YMAWICKMER			Carbamidomethyl (C)[6], Oxidation (M)[2,8]		Mascot
1448.6951	1448.743	0.0479	33	2	13	STVPPFEECQRR					Mascot
2364.1846	2364.2085	0.0239	10	14	33	FVLYCIAHGLQPGDEWKPYK					Mascot
2391.1252	2391.2012	0.076	32	55	73	WTPIDDQDDFTLYIEQHVR					Mascot

3	Uncharacterized protein (Fragment) OS=Alicyclobacillus acidoterrestris (strain ATCC 49025 / DSM 3922 / CIP 106132 / NCIMB 13137 / GD3B) GN=N007_15525 PE=4 SV=1	tr|T0BE66|T0BE66_ALIAG	42678.4	5.95	8	25	0

Peptide Information

Calc. Mass	Obsrv. Mass	± da	± ppm	Start Seq.	End Seq.	Sequence	Ion Score	C. I. %	Modification	Rank	Result Type
1416.6464	1416.7019	0.0555	39	202	212	YFEIMNAELDR			Oxidation (M)[5]		Mascot
1554.7581	1554.7366	-0.0215	-14	350	363	IITAHHGDMEISSK			Oxidation (M)[9]		Mascot
2101.1296	2101.0906	-0.039	-19	215	232	MILSEMLVLAKPQSTHFR					Mascot
2372.3408	2372.2068	-0.134	-56	163	185	LNAVGQLAAGVAHEIRNPLTSLK					Mascot
2374.2986	2374.2168	-0.0818	-34	213	232	IKMILSEMLVLAKPQSTHFR			Oxidation (M)[3,8]		Mascot
2388.3796	2388.1924	-0.1872	-78	234	254	SHIKLILHEVITLLSALANMR			Oxidation (M)[20]		Mascot
2630.3938	2630.2825	-0.1113	-42	122	145	NGEVFHAELLGTYIGDNAIKISLR					Mascot
2697.2383	2697.2439	0.0056	2	255	278	SVDIQTAFDDNEVAMVLCDENQLK					Mascot

4	Uncharacterized protein OS=Alicyclobacillus acidoterrestris (strain ATCC 49025 / DSM 3922 / CIP 106132 / NCIMB 13137 / GD3B) GN=N007_17135 PE=4 SV=1	tr|T0CV51|T0CV51_ALIAG	5851	6.71	4	24	0

Peptide Information

Calc. Mass	Obsrv. Mass	± da	± ppm	Start Seq.	End Seq.	Sequence	Ion Score	C. I. %	Modification	Rank	Result Type

Calc. Mass	Obsrv. Mass	± da	± ppm	Start Seq.	End Seq.	Sequence	Ion Score	C. I. %	Modification	Rank	Result Type
947.4429	947.4686	0.0257	27	33	40	VDTQDNQK					Mascot
947.4429	947.4686	0.0257	27	33	40	VDTQDNQK					Mascot
1193.6559	1193.6648	0.0089	7	5	14	LMSSLETLRK			Oxidation (M)[2]		Mascot
1395.7148	1395.6786	-0.0362	-26	2	13	SDKLMSSLETLR			Oxidation (M)[5]		Mascot
1458.7911	1458.7008	-0.0903	-62	15	27	SSDLDLVIRVDAR					Mascot
1458.7911	1458.7008	-0.0903	-62	15	27	SSDLDLVIRVDAR					Mascot

5	Uncharacterized protein OS=Alicyclobacillus acidoterrestris (strain ATCC 49025 / DSM 3922 / CIP 106132 / NCIMB 13137 / GD3B) GN=N007_02385 PE=4 SV=1	tr|T0CIZ5|T0CIZ5_ALIAG	47574.2	9.72	8	23	0

Peptide Information

Calc. Mass	Obsrv. Mass	± da	± ppm	Start Seq.	End Seq.	Sequence	Ion Score	C. I. %	Modification	Rank	Result Type
1075.5994	1075.5626	-0.0368	-34	149	158	QLTVLASESK					Mascot
1075.5994	1075.5626	-0.0368	-34	149	158	QLTVLASESK					Mascot
2108.9785	2109.125	0.1465	69	72	88	SNTWYNTRFVTSQEGYR					Mascot
2124.0735	2124.0371	-0.0364	-17	159	175	AQIWSQHQFRLPDDNLR					Mascot
2135.1062	2135.0195	-0.0867	-41	6	25	VLPILLSLNCAWMTTGCGVK			Oxidation (M)[13]		Mascot
2357.2361	2357.1631	-0.073	-31	149	168	QLTVLASESKAQIWSQHQFR					Mascot
2359.1711	2359.1965	0.0254	11	176	198	IHSVSMLSNGDGWVVLSNATSGK					Mascot
2359.1711	2359.1965	0.0254	11	176	198	IHSVSMLSNGDGWVVLSNATSGK					Mascot
2391.1882	2391.2012	0.013	5	302	323	YVGFQSGHAVVHTAVFWTEAGK					Mascot
2525.3555	2525.2993	-0.0562	-22	3	25	LHRVLPILLSLNCAWMTTGCGVK					Mascot

6	Enolase OS=Alicyclobacillus acidoterrestris (strain ATCC 49025 / DSM 3922 / CIP 106132 / NCIMB 13137 / GD3B) GN=eno PE=3 SV=1	tr|T0D603|T0D603_ALIAG	45854.4	4.81	8	22	0

Peptide Information

Calc. Mass	Obsrv. Mass	± da	± ppm	Start Seq.	End Seq.	Sequence	Ion Score	C. I. %	Modification	Rank	Result Type
1175.5878	1175.6488	0.061	52	181	190	MGAEIFHNLK			Oxidation (M)[1]		Mascot
1349.593	1349.7207	0.1277	95	264	274	TADEMIAYYEK			Oxidation (M)[5]		Mascot
1458.8348	1458.7008	-0.134	-92	106	120	LGANAILTVSMAVAK					Mascot
1458.8348	1458.7008	-0.134	-92	106	120	LGANAILTVSMAVAK					Mascot
1643.9513	1643.9275	-0.0238	-14	104	120	GKLGANAILTVSMAVAK					Mascot
2295.1868	2295.2268	0.04	17	229	250	AGYKPGEQASIALDVASTELFK					Mascot

2411.1887	2411.1265	-0.0622	-26	133	154	YLGGFYAHTLPTPMMNIL NGGK			Oxidation (M)[14]		Mascot
2501.188	2501.3281	0.1401	56	155	176	HADNTVDIQEFMVVPHG ATTFR			Oxidation (M)[12]		Mascot
2525.1289	2525.2993	0.1704	67	254	274	YHFEGEGVTRTADEMIAY YEK			Oxidation (M)[15]		Mascot

7	Uncharacterized protein OS=Alicyclobacillus acidoterrestris (strain ATCC 49025 / DSM 3922 / CIP 106132 / NCIMB 13137 / GD3B) GN=N007_14080 PE=4 SV=1	tr|T0BFZ5|T0BFZ5_ALIAG	14440.4	6.84	4	21	0

Peptide Information

Calc. Mass	Obsrv. Mass	± da	± ppm	Start Seq.	End Seq.	Sequence	Ion Score	C. I. %	Modification	Rank	Result Type
2357.1707	2357.1631	-0.0076	-3	87	106	DLVNEEWVKMFPDPASR PAR					Mascot
2416.1086	2416.1863	0.0777	32	65	86	NAGGSPDDIGKMTVYLA DEQYR			Oxidation (M)[12]		Mascot
2501.2017	2501.3281	0.1264	51	76	95	MTVYLADEQYRDLVNEE WVK					Mascot
2694.4534	2694.2927	-0.1607	-60	107	131	HSTVSQLRNGALVQVEIT AVIDSTR					Mascot

8	Uncharacterized protein OS=Alicyclobacillus acidoterrestris (strain ATCC 49025 / DSM 3922 / CIP 106132 / NCIMB 13137 / GD3B) GN=N007_03250 PE=3 SV=1	tr|T0CGH4|T0CGH4_ALIAG	13481.7	4.88	4	21	0

Peptide Information

Calc. Mass	Obsrv. Mass	± da	± ppm	Start Seq.	End Seq.	Sequence	Ion Score	C. I. %	Modification	Rank	Result Type
947.4581	947.4686	0.0105	11	70	77	AQQEAWSK					Mascot
947.4581	947.4686	0.0105	11	70	77	AQQEAWSK					Mascot
1175.5944	1175.6488	0.0544	46	107	118	TLPGFIGGEVDA					Mascot
2501.0854	2501.3281	0.2427	97	85	106	VRHELSAASQMEMDAAF ASMDR			Oxidation (M)[11,13,20]		Mascot
3133.5623	3133.522	-0.0403	-13	16	41	QRQIIEMYHVEDWSLSEI AESLSITR					Mascot

9	Uncharacterized protein (Fragment) OS=Alicyclobacillus acidoterrestris (strain ATCC 49025 / DSM 3922 / CIP 106132 / NCIMB 13137 / GD3B) GN=N007_19610 PE=4 SV=1	tr|T0CIX3|T0CIX3_ALIAG	32792.5	8.84	6	21	0

Peptide Information

Calc. Mass	Obsrv. Mass	± da	± ppm	Start Seq.	End Seq.	Sequence	Ion Score	C. I. %	Modification	Rank	Result Type
1419.6283	1419.6639	0.0356	25	124	135	YVMMDAGYDQVK					Mascot
1599.7432	1599.8925	0.1493	93	155	170	GEKEPPAGMASDGTPR					Mascot
1987.8922	1988.0796	0.1874	94	198	217	VGCPLGTAACSDSNYGM			Oxidation (M)[17]		Mascot

						VVK					
2085.9402	2086.0791	0.1389	67	198	217	VGCPLGTAACSDSNYGM VVK			Carbamidomethyl (C)[3,10]		Mascot
2085.9402	2086.0791	0.1389	67	198	217	VGCPLGTAACSDSNYGM VVK			Carbamidomethyl (C)[3,10]		Mascot
2123.9524	2124.0371	0.0847	40	124	141	YVMMDAGYDQVKNYEA AR					Mascot
2664.2402	2664.2739	0.0337	13	191	217	CPHAVGKVGCPLGTAAC SDSNYGMVVK					Mascot
2680.2351	2680.2791	0.044	16	191	217	CPHAVGKVGCPLGTAAC SDSNYGMVVK			Oxidation (M)[24]		Mascot
3181.2754	3181.4548	0.1794	56	158	186	EPPAGMASDGTPRCSM GYDMVYWGADGDR			Carbamidomethyl (C)[14], Oxidation (M)[6,16]		Mascot

10	Probable dual-specificity RNA methyltransferase RlmN OS=Alicyclobacillus acidoterrestris (strain ATCC 49025 / DSM 3922 / CIP 106132 / NCIMB 13137 / GD3B) GN=rlmN PE=3 SV=1	tr|T0CFH5|T0CFH 5_ALIAG	38926.4	7.15	9	21	0	

Peptide Information

Calc. Mass	Obsrv. Mass	± da	± ppm	Start Seq.	End Seq.	Sequence	Ion Score	C. I. %	Modification	Rank	Result Type
1395.6825	1395.6786	-0.0039	-3	39	50	VTTFDEMTNLPK					Mascot
1434.8679	1434.7361	-0.1318	-92	190	203	HITISTVGLVPGIK					Mascot
1458.614	1458.7008	0.0868	60	241	251	LMDACHYYNQK			Carbamidomethyl (C)[5], Oxidation (M)[2]		Mascot
1458.614	1458.7008	0.0868	60	241	251	LMDACHYYNQK			Carbamidomethyl (C)[5], Oxidation (M)[2]		Mascot
1643.774	1643.9275	0.1535	93	326	340	REMGHDIAAACGQLR			Oxidation (M)[3]		Mascot
2085.9885	2086.0791	0.0906	43	235	251	AYPIAKLMDACHYYNQK			Carbamidomethyl (C)[11]		Mascot
2085.9885	2086.0791	0.0906	43	235	251	AYPIAKLMDACHYYNQK			Carbamidomethyl (C)[11]		Mascot
2092.0745	2092.0386	-0.0359	-17	132	149	AGEMVEQLLFSQRLLDD K					Mascot
2101.0271	2101.0906	0.0635	30	256	274	ISFEYALVGGKNDSLECA K			Carbamidomethyl (C)[17]		Mascot
2108.9167	2109.125	0.2083	99	114	131	MGCTFCASTLGGMIRQM R			Carbamidomethyl (C)[3,6], Oxidation (M)[1,13]		Mascot
2371.2307	2371.2	-0.0307	-13	307	326	QIYAFWNALQSYGVNATI RR					Mascot

Gel Idx/Pos	236/J12	Instr./Gel Origin	BA2151/full sequence test 20150515	Process Status	Analysis Succeeded
Plate [#] Name	[3] full sequence test 20150515	Instrument Sample Name		Spectra	11

Rank	Protein Name	Species	Accession No.	Protein MW	Protein PI	Pep. Count	Protein Score	Protein Score C. I. %	Total Ion Score	Total Ion C. I. %
1	Uncharacterized protein OS=Alicyclobacillus acidoterrestris (strain ATCC 49025 / DSM 3922 / CIP 106132 / NCIMB 13137 / GD3B) GN=N007_08970 PE=4 SV=1		tr|T0BYX4|T0BYX4_ALIAG	114313.8	4.01	6	183	100	183	100

Peptide Information

Calc. Mass	Obsrv. Mass	± da	± ppm	Start Seq.	End Seq.	Sequence	Ion Score	C. I. %	Modification	Rank	Result Type
807.4359	807.4256	-0.0103	-13	121	127	FNTQVAK					Mascot
935.5309	935.5226	-0.0083	-9	120	127	KFNTQVAK					Mascot
1117.5848	1117.5908	0.006	5	243	253	DANGNVLSVTK					Mascot
1125.6627	1125.6521	-0.0106	-9	35	45	ASQLPIVVNGK					Mascot
1238.6085	1238.6074	-0.0011	-1	46	56	VLSNPYEMTGK					Mascot
1238.6085	1238.6074	-0.0011	-1	46	56	VLSNPYEMTGK	45	99.983			Mascot
1254.6035	1254.5989	-0.0046	-4	46	56	VLSNPYEMTGK			Oxidation (M)[8]		Mascot
1254.6035	1254.5989	-0.0046	-4	46	56	VLSNPYEMTGK	45	99.982	Oxidation (M)[8]		Mascot
2254.0815	2254.0627	-0.0188	-8	57	76	DSGNTTGFFPIYYFNQALAK					Mascot
2254.0815	2254.0627	-0.0188	-8	57	76	DSGNTTGFFPIYYFNQALAK	137	100			Mascot

Rank	Protein Name	Species	Accession No.	Protein MW	Protein PI	Pep. Count	Protein Score	Protein Score C. I. %	Total Ion Score	Total Ion C. I. %
2	NADP-dependent aryl-alcohol dehydrogenase OS=Alicyclobacillus acidoterrestris (strain ATCC 49025 / DSM 3922 / CIP 106132 / NCIMB 13137 / GD3B) GN=N007_14115 PE=4 SV=1		tr|T0BQW0|T0BQW0_ALIAG	36505.5	5.6	13	62	99.735		

Peptide Information

Calc. Mass	Obsrv. Mass	± da	± ppm	Start Seq.	End Seq.	Sequence	Ion Score	C. I. %	Modification	Rank	Result Type
1250.6296	1250.6241	-0.0055	-4	106	116	AIMTEIDNSLK			Oxidation (M)[3]		Mascot
1323.738	1323.6573	-0.0807	-61	81	92	DEVVIATKVHGR					Mascot
1340.7216	1340.6666	-0.055	-41	93	105	MRPGPNGAGLSRK					Mascot
1378.7246	1378.6886	-0.036	-26	105	116	KAIMTEIDNSLK			Oxidation (M)[4]		Mascot
1557.6859	1557.832	0.1461	94	16	28	ICLGCMSYGVPER			Carbamidomethyl (C)[2,5], Oxidation (M)[6]		Mascot
1557.6859	1557.832	0.1461	94	16	28	ICLGCMSYGVPER			Carbamidomethyl (C)[2,5], Oxidation (M)[6]		Mascot
1592.8068	1592.7966	-0.0102	-6	118	130	LGTDYVDLYQIHR					Mascot
1652.8795	1652.8751	-0.0044	-3	313	326	LEEPYVPHPVLGFR					Mascot

1660.8516	1660.8528	0.0012	1	181	193	FVSMQNYLNLLYR					Mascot
2281.1719	2281.071	-0.1009	-44	198	218	EMLPLCEAEGIGVIPWSPLAR					Mascot
2291.0537	2291.1716	0.1179	51	131	149	WDYDTPIEETMEALHDVVK					Mascot
2362.3857	2362.1738	-0.2119	-90	268	290	AQIALAWVLQKSPVTAPIVGATK					Mascot
2407.2173	2407.2026	-0.0147	-6	291	312	MHHLDDAVASLSITLTPEEIAK			Oxidation (M)[1]		Mascot
2407.2173	2407.2026	-0.0147	-6	291	312	MHHLDDAVASLSITLTPEEIAK			Oxidation (M)[1]		Mascot
2441.1985	2441.0872	-0.1113	-46	6	28	LGTTGLEVSRICLGCMSYGVPER					Mascot

3	Uncharacterized protein OS=Alicyclobacillus acidoterrestris (strain ATCC 49025 / DSM 3922 / CIP 106132 / NCIMB 13137 / GD3B) GN=N007_18905 PE=4 SV=1	tr|T0DU28|T0DU28_ALIAG	50399.8	5.48	14	50	95.802

Peptide Information

Calc. Mass	Obsrv. Mass	± da	± ppm	Start Seq.	End Seq.	Sequence	Ion Score	C. I. %	Modification	Rank	Result Type
935.4363	935.5226	0.0863	92	282	289	MESGRPSR			Oxidation (M)[1]		Mascot
1064.5194	1064.5374	0.018	17	120	128	AVAYPCVER			Carbamidomethyl (C)[6]		Mascot
1106.5874	1106.5221	-0.0653	-59	368	377	LASSDVMVIR			Oxidation (M)[7]		Mascot
1252.6144	1252.6207	0.0063	5	129	139	GGLVWTYMGPR			Oxidation (M)[8]		Mascot
1378.6494	1378.6886	0.0392	28	299	309	YPDMQVPMLPR			Oxidation (M)[4,8]		Mascot
1621.6873	1621.7872	0.0999	62	102	115	CIDMPNEPPESDFK					Mascot
1652.7275	1652.8751	0.1476	89	89	101	CVYHGWQFDVEGR			Carbamidomethyl (C)[1]		Mascot
1678.7087	1678.8219	0.1132	67	102	115	CIDMPNEPPESDFK			Carbamidomethyl (C)[1]		Mascot
2210.0522	2210.0559	0.0037	2	299	316	YPDMQVPMLPRTTDWFGR					Mascot
2225.0994	2225.0867	-0.0127	-6	120	139	AVAYPCVERGGLVWTYMGPR					Mascot
2236.0486	2236.1104	0.0618	28	5	24	EDNEFISRVGPGTPMGNLMR			Oxidation (M)[15]		Mascot
2305.1685	2305.1108	-0.0577	-25	317	335	FRPAVNADNDYLIDRELQR					Mascot
2313.1731	2313.1138	-0.0593	-26	290	309	LPIQGNSEKYPDMQVPMLPR					Mascot
2374.1206	2374.0979	-0.0227	-10	25	45	QYWIPAMLSSELPGPDCDPVR					Mascot
2390.1155	2390.1084	-0.0071	-3	25	45	QYWIPAMLSSELPGPDCDPVR			Oxidation (M)[7]		Mascot
3312.4185	3312.3247	-0.0938	-28	89	115	CVYHGWQFDVEGRCIDMPNEPPESDFK			Carbamidomethyl (C)[1,14]		Mascot

4	Uncharacterized protein OS=Alicyclobacillus acidoterrestris (strain ATCC 49025 / DSM 3922 / CIP 106132 / NCIMB 13137 / GD3B) GN=N007_18525 PE=4 SV=1	tr|T0DUD9|T0DUD9_ALIAG	51393.4	10.24	12	38	40.708

Peptide Information

Calc. Mass	Obsrv. Mass	± da	± ppm	Start Seq.	End Seq.	Sequence	Ion Score	C. I. %	Modification	Rank	Result Type
935.4866	935.5226	0.036	38	376	383	ETIAKMDK					Mascot
1106.5841	1106.5221	-0.062	-56	253	262	GALIEATFER					Mascot
1218.6631	1218.6298	-0.0333	-27	137	146	VFDPVAKTWR					Mascot
1250.6344	1250.6241	-0.0103	-8	427	436	MLRMLSQDTR					Mascot
1557.925	1557.832	-0.093	-60	83	97	LGPVLGLYVDLIASK					Mascot
1557.925	1557.832	-0.093	-60	83	97	LGPVLGLYVDLIASK					Mascot
1592.781	1592.7966	0.0156	10	36	49	ATTVQCTRNQGQTK			Carbamidomethyl (C)[6]		Mascot
1913.024	1912.8885	-0.1355	-71	325	341	WLVQSSDEPTVVARVAR					Mascot
2236.1067	2236.1104	0.0037	2	280	298	TTIEHSVIRSVDECVSFW K					Mascot
2281.2227	2281.071	-0.1517	-66	391	411	TLATRYGPFAELGIDFALT PK					Mascot
2290.1714	2290.1338	-0.0376	-16	305	324	AVVQDTVELPKLDGQPY DFR					Mascot
2293.1282	2293.0671	-0.0611	-27	280	298	TTIEHSVIRSVDECVSFW K			Carbamidomethyl (C)[14]		Mascot
2313.2061	2313.1138	-0.0923	-40	184	202	LYTLPSQCSNKWTLYQV LR					Mascot
2333.2361	2333.1355	-0.1006	-43	231	251	NDVYVKPPGGSQGVSIY RLQR					Mascot

5	Uncharacterized protein OS=Alicyclobacillus acidoterrestris (strain ATCC 49025 / DSM 3922 / CIP 106132 / NCIMB 13137 / GD3B) GN=N007_12105 PE=4 SV=1	tr|T0BSL1|T0BSL1 _ALIAG	40660.2	5.65	9	32	0

Peptide Information

Calc. Mass	Obsrv. Mass	± da	± ppm	Start Seq.	End Seq.	Sequence	Ion Score	C. I. %	Modification	Rank	Result Type
807.3995	807.4256	0.0261	32	142	147	LDFEQR					Mascot
1125.5898	1125.6521	0.0623	55	280	288	HDITEIIER					Mascot
1252.7261	1252.6207	-0.1054	-84	2	14	LPVGLDLGNGAVK					Mascot
2289.1367	2289.1228	-0.0139	-6	175	195	VVSEGHMAFLALCQQSP EKSK					Mascot
2305.1316	2305.1108	-0.0208	-9	175	195	VVSEGHMAFLALCQQSP EKSK			Oxidation (M)[7]		Mascot
2318.2	2318.0828	-0.1172	-51	280	299	HDITEIIERHLSAAASHVY R					Mascot
2335.2114	2335.1716	-0.0398	-17	350	371	WLNADGMFLTAVRLAAD TVTAV					Mascot
2335.2114	2335.1716	-0.0398	-17	350	371	WLNADGMFLTAVRLAAD TVTAV					Mascot
2362.1531	2362.1738	0.0207	9	175	195	VVSEGHMAFLALCQQSP EKSK			Carbamidomethyl (C)[13], Oxidation (M)[7]		Mascot
2374.1997	2374.0979	-0.1018	-43	41	62	QEPLNALDVEVVSSAFTD GARR					Mascot

Calc. Mass	Obsrv. Mass	± da	± ppm	Start Seq.	End Seq.	Sequence	Ion Score	C. I. %	Modification	Rank	Result Type
2380.2905	2380.1257	-0.1648	-69	86	107	TSSEQAILMYLTALASAVVKQR					Mascot
2408.1914	2408.1951	0.0037	2	63	84	VFVGKLAADQGPQATYMQPNEK			Oxidation (M)[17]		Mascot

6	Glycosyl transferase OS=Alicyclobacillus acidoterrestris (strain ATCC 49025 / DSM 3922 / CIP 106132 / NCIMB 13137 / GD3B) GN=N007_03170 PE=4 SV=1	tr|T0CFI7|T0CFI7_ALIAG	43243.4	7.19	9	30	0

Peptide Information

Calc. Mass	Obsrv. Mass	± da	± ppm	Start Seq.	End Seq.	Sequence	Ion Score	C. I. %	Modification	Rank	Result Type
935.4502	935.5226	0.0724	77	154	161	ATMNELEK					Mascot
1581.8456	1581.7715	-0.0741	-47	140	153	TLHNRADINLATSR					Mascot
2015.0155	2014.9445	-0.071	-35	3	20	IAMFTETFLPSTDGIVTR			Oxidation (M)[3]		Mascot
2086.0869	2086.0608	-0.0261	-13	106	123	YHLPLIASYHTNVPAYAR					Mascot
2284.2007	2284.1431	-0.0576	-25	3	22	IAMFTETFLPSTDGIVTRLR			Oxidation (M)[3]		Mascot
2286.1621	2286.1118	-0.0503	-22	1	20	MRIAMFTETFLPSTDGIVTR					Mascot
2318.1519	2318.0828	-0.0691	-30	1	20	MRIAMFTETFLPSTDGIVTR			Oxidation (M)[1,5]		Mascot
2353.2625	2353.1731	-0.0894	-38	47	67	YASATIVGIPAMPFILYPEKR			Oxidation (M)[12]		Mascot
2374.1497	2374.0979	-0.0518	-22	351	370	TLDWEGPSRQLLEHYEAVCK					Mascot
2718.4192	2718.3274	-0.0918	-34	82	105	FQPDLIHAVNPAFLGLGGIYYAWR					Mascot

7	Anti-sigma F factor antagonist OS=Alicyclobacillus acidoterrestris (strain ATCC 49025 / DSM 3922 / CIP 106132 / NCIMB 13137 / GD3B) GN=N007_03480 PE=3 SV=1	tr|T0C8T4|T0C8T4_ALIAG	12526.5	5.55	5	29	0

Peptide Information

Calc. Mass	Obsrv. Mass	± da	± ppm	Start Seq.	End Seq.	Sequence	Ion Score	C. I. %	Modification	Rank	Result Type
2014.9685	2014.9445	-0.024	-12	68	86	SISQHGGQMALCEVGPSLK			Carbamidomethyl (C)[12], Oxidation (M)[9]		Mascot
2086.042	2086.0608	0.0188	9	68	87	SISQHGGQMALCEVGPSLKK			Oxidation (M)[9]		Mascot
2316.1177	2315.9915	-0.1262	-54	30	49	DDIEAQLAKTDYQGLVMSFR			Oxidation (M)[17]		Mascot
2316.1177	2315.9915	-0.1262	-54	30	49	DDIEAQLAKTDYQGLVMSFR			Oxidation (M)[17]		Mascot
2318.1379	2318.0828	-0.0551	-24	66	86	FRSISQHGGQMALCEVGPSLK			Carbamidomethyl (C)[14], Oxidation (M)[11]		Mascot
2350.2039	2350.146	-0.0579	-25	7	27	YENGVLVISLQGELDHHAVEK					Mascot

8	Uncharacterized protein OS=Alicyclobacillus acidoterrestris (strain ATCC 49025 / DSM 3922 / CIP 106132 / NCIMB 13137 / GD3B) GN=N007_12680 PE=4 SV=1	tr|T0BHY5|T0BHY5_ALIAG	51353.1	5.59	10	29	0

Peptide Information

Calc. Mass	Obsrv. Mass	± da	± ppm	Start Seq.	End Seq.	Sequence	Ion Score	C. I. %	Modification	Rank	Result Type
1117.5782	1117.5908	0.0126	11	105	114	AMADAEVVRR					Mascot
1292.6633	1292.5574	-0.1059	-82	460	469	LIHLAYDYER					Mascot
1550.6688	1550.7284	0.0596	38	318	330	MNAGMDVMVYEFK			Oxidation (M)[1]		Mascot
2139.1082	2139.0146	-0.0936	-44	46	65	AVIEVNPDALWIAAALDS DR					Mascot
2180.0957	2179.9834	-0.1123	-52	405	423	ALKDHGLDALLFPSYFGC R			Carbamidomethyl (C)[18]		Mascot
2251.1328	2251.0459	-0.0869	-39	408	427	DHGLDALLFPSYFGCRIA AK			Carbamidomethyl (C)[15]		Mascot
2326.2654	2326.0723	-0.1931	-83	296	317	AVEDLTRLGAVVVDLTDF PPAK					Mascot
2360.0242	2360.1365	0.1123	48	83	104	DNLDTADTMHTTAGSVA MAEHR			Oxidation (M)[9]		Mascot
2372.1375	2372.094	-0.0435	-18	16	36	QQRGEMSAYEIAVACFE QIAK					Mascot
2441.1304	2441.0872	-0.0432	-18	126	146	ANLTEWANFISDHMKNG YSSR					Mascot

9	Aldehyde dehydrogenase OS=Alicyclobacillus acidoterrestris (strain ATCC 49025 / DSM 3922 / CIP 106132 / NCIMB 13137 / GD3B) GN=gabD PE=3 SV=1	tr|T0DTL9|T0DTL9 _ALIAG	52934.1	5.74	10	28	0

Peptide Information

Calc. Mass	Obsrv. Mass	± da	± ppm	Start Seq.	End Seq.	Sequence	Ion Score	C. I. %	Modification	Rank	Result Type
1117.5637	1117.5908	0.0271	24	213	221	EFLENPNVR					Mascot
1204.5555	1204.6331	0.0776	64	465	474	YAMDAFLETK			Oxidation (M)[3]		Mascot
1278.6213	1278.7128	0.0915	72	120	131	VYGETVPSSDPK					Mascot
1838.9033	1838.9152	0.0119	6	465	480	YAMDAFLETKFVSFGI					Mascot
2086.1213	2086.0608	-0.0605	-29	82	100	DELATIIATEMGKPLREAK					Mascot
2266.2166	2266.1138	-0.1028	-45	139	159	QPVGVVAAITPWNFPVN MVAR					Mascot
2284.0298	2284.1431	0.1133	50	404	423	MANDSTYGLAAYVYTRD NSR			Oxidation (M)[1]		Mascot
2286.0647	2286.1118	0.0471	21	101	119	WEVLGAADNFMWYAEE AKR					Mascot
2360.1729	2360.1365	-0.0364	-15	24	46	VISPANGEEVGVVTFGDE RDAAK					Mascot
2463.4004	2463.1929	-0.2075	-84	161	185	IAPALAAGCTVILKPAEST PLSAIR					Mascot

10	Hypoxanthine phosphoribosyltransferase OS=Alicyclobacillus acidoterrestris (strain ATCC 49025 / DSM 3922 / CIP 106132 / NCIMB 13137 / GD3B) GN=N007_00050 PE=4 SV=1	tr|T0DP70|T0DP70 _ALIAG	20557.6	5.25	6	26	0

Peptide Information

Calc. Mass	Obsrv. Mass	± da	± ppm	Start Seq.	End Seq.	Sequence	Ion Score	C. I. %	Modification	Rank	Result Type
1117.6438	1117.5908	-0.053	-47	34	42	NPLFICILK			Carbamidomethyl (C)[6]		Mascot
1218.6412	1218.6298	-0.0114	-9	109	117	YLRETLMHR					Mascot
2224.9812	2225.0867	0.1055	47	55	74	VEVPMEMDFMAISSYGT SSK			Oxidation (M)[5]		Mascot
2293.2764	2293.0671	-0.2093	-91	88	108	SIEGRHVVIVEDIVDTGLT LK					Mascot
2387.1912	2387.1694	-0.0218	-9	1	20	MHPDLESILFDEETIQSKV R					Mascot
2397.0771	2397.0894	0.0123	5	54	74	RVEVPMEMDFMAISSYG TSSK			Oxidation (M)[6,8]		Mascot

Gel Idx/Pos	237/J13	Instr./Gel Origin	BA2151/full sequence test 20150515	Process Status	Analysis Succeeded
Plate [#] Name	[3] full sequence test 20150515	Instrument Sample Name		Spectra	11

Rank	Protein Name	Species	Accession No.	Protein MW	Protein PI	Pep. Count	Protein Score	Protein Score C. I. %	Total Ion Score	Total Ion C. I. %
1	Glyoxalase OS=Alicyclobacillus acidoterrestris (strain ATCC 49025 / DSM 3922 / CIP 106132 / NCIMB 13137 / GD3B) GN=N007_19125 PE=4 SV=1		tr|T0DNC0|T0DNC0_ALIAG	16114.8	5.63	4	19	0		

Peptide Information

Calc. Mass	Obsrv. Mass	± da	± ppm	Start Seq.	End Seq.	Sequence	Ion Score	C. I. %	Modification	Rank	Result Type
1134.5803	1134.4817	-0.0986	-87	67	75	HFGVTFRDR					Mascot
1426.705	1426.6583	-0.0467	-33	63	73	MYPRHFGVTFR			Oxidation (M)[1]		Mascot
1893.8939	1893.9143	0.0204	11	16	31	DLDEAYTFYVEKLGCK					Mascot
2754.2717	2754.1047	-0.167	-61	40	62	ITLDFFGDQVVCHLSQDYDQEPK			Carbamidomethyl (C)[12]		Mascot

Rank	Protein Name	Species	Accession No.	Protein MW	Protein PI	Pep. Count	Protein Score	Protein Score C. I. %	Total Ion Score	Total Ion C. I. %
2	Uncharacterized protein OS=Alicyclobacillus acidoterrestris (strain ATCC 49025 / DSM 3922 / CIP 106132 / NCIMB 13137 / GD3B) GN=N007_13735 PE=4 SV=1		tr|T0BRU1|T0BRU1_ALIAG	12979.9	6.25	3	19	0		

Peptide Information

Calc. Mass	Obsrv. Mass	± da	± ppm	Start Seq.	End Seq.	Sequence	Ion Score	C. I. %	Modification	Rank	Result Type
2595.229	2595.3508	0.1218	47	34	54	YQQMMQQELQNAQTLEQLAQR			Oxidation (M)[4]		Mascot
2698.2495	2698.1753	-0.0742	-27	55	77	EHQAAQMIGQVLQGHQTVMEQMR			Oxidation (M)[7,19,22]		Mascot
3923.7131	3923.9421	0.229	58	1	33	MYGNQMQSMSAQLNQCRQIAQQMMQQTQQSSQR			Oxidation (M)[1,6]		Mascot

Rank	Protein Name	Species	Accession No.	Protein MW	Protein PI	Pep. Count	Protein Score	Protein Score C. I. %	Total Ion Score	Total Ion C. I. %
3	Uncharacterized protein (Fragment) OS=Alicyclobacillus acidoterrestris (strain ATCC 49025 / DSM 3922 / CIP 106132 / NCIMB 13137 / GD3B) GN=N007_21290 PE=4 SV=1		tr|T0C8C1|T0C8C1_ALIAG	22026.5	9.54	4	16	0		

Peptide Information

Calc. Mass	Obsrv. Mass	± da	± ppm	Start Seq.	End Seq.	Sequence	Ion Score	C. I. %	Modification	Rank	Result Type
1287.6804	1287.6008	-0.0796	-62	145	155	HQVSINPEHVK					Mascot
1439.7489	1439.6757	-0.0732	-51	169	181	VAGLQVEPDVEQR					Mascot
2698.3447	2698.1753	-0.1694	-63	93	115	VLNDCRISWNASLYSVPWQYVGK					Mascot
3267.7122	3267.5715	-0.1407	-43	127	155	LQIEYGGQVIAEHSLVDK					Mascot

Calc. Mass	Obsrv. Mass	± da	± ppm	Start Seq.	End Seq.	Sequence	Ion Score	C. I. %	Modification	Rank	Result Type
						HQVSINPEHVK					

4	Uncharacterized protein OS=Alicyclobacillus acidoterrestris (strain ATCC 49025 / DSM 3922 / CIP 106132 / NCIMB 13137 / GD3B) GN=N007_04340 PE=4 SV=1	tr|T0DHR4|T0DHR4_ALIAG	5455.6	6.25	3	16	0

Peptide Information

Calc. Mass	Obsrv. Mass	± da	± ppm	Start Seq.	End Seq.	Sequence	Ion Score	C. I. %	Modification	Rank	Result Type
827.4006	827.4486	0.048	58	39	45	HEEGLSR					Mascot
1110.5175	1110.4357	-0.0818	-74	39	48	HEEGLSRGEP					Mascot
1488.7037	1488.6123	-0.0914	-61	33	45	SETSTRHEEGLSR					Mascot

5	Uncharacterized protein OS=Alicyclobacillus acidoterrestris (strain ATCC 49025 / DSM 3922 / CIP 106132 / NCIMB 13137 / GD3B) GN=N007_02255 PE=4 SV=1	tr|T0DNJ2|T0DNJ2_ALIAG	20846.6	9.12	4	15	0

Peptide Information

Calc. Mass	Obsrv. Mass	± da	± ppm	Start Seq.	End Seq.	Sequence	Ion Score	C. I. %	Modification	Rank	Result Type
947.4291	947.3426	-0.0865	-91	1	7	MAEYVYR			Oxidation (M)[1]		Mascot
1765.9194	1765.9296	0.0102	6	136	151	FADVGYPCDLVVGLIK			Carbamidomethyl (C)[8]		Mascot
1894.0143	1893.9143	-0.1	-53	135	151	KFADVGYPCDLVVGLIK			Carbamidomethyl (C)[9]		Mascot
2852.2344	2851.9651	-0.2693	-94	152	173	WFLIEHDMNDWNTFGRDCAFYR			Oxidation (M)[8]		Mascot

6	6-phosphogluconate dehydrogenase OS=Alicyclobacillus acidoterrestris (strain ATCC 49025 / DSM 3922 / CIP 106132 / NCIMB 13137 / GD3B) GN=N007_01705 PE=4 SV=1	tr|T0CYB3|T0CYB3_ALIAG	32612.2	5.57	5	15	0

Peptide Information

Calc. Mass	Obsrv. Mass	± da	± ppm	Start Seq.	End Seq.	Sequence	Ion Score	C. I. %	Modification	Rank	Result Type
875.4985	875.479	-0.0195	-22	144	150	TIEPLFR					Mascot
1412.6805	1412.6648	-0.0157	-11	151	163	DTAVPNGYVYTGR					Mascot
1439.6987	1439.6757	-0.023	-16	216	227	GWLMELTESAFR					Mascot
1870.8719	1870.8535	-0.0184	-10	111	128	HGIYFFDVGTSGGTEGAR					Mascot
3435.4934	3435.3682	-0.1252	-36	164	194	AGSGHYCKMVHNGIEYGMMAAIGEGFEVMEK			Carbamidomethyl (C)[7], Oxidation (M)[9,18]		Mascot

7	Uncharacterized protein OS=Alicyclobacillus acidoterrestris (strain ATCC 49025 / DSM 3922 / CIP 106132 / NCIMB 13137 / GD3B) GN=N007_00785 PE=4 SV=1	tr|T0BW05|T0BW05_ALIAG	23153.5	5.64	4	14	0

Peptide Information

Calc. Mass	Obsrv. Mass	± da	± ppm	Start Seq.	End Seq.	Sequence	Ion Score	C. I. %	Modification	Rank	Result Type
875.4668	875.479	0.0122	14	95	100	MNWRLR					Mascot
1110.5249	1110.4357	-0.0892	-80	177	185	DDCLYVQVR					Mascot
1181.595	1181.5446	-0.0504	-43	128	137	FAVDFLEANR					Mascot
3566.6978	3566.9739	0.2761	77	156	185	CGYHLEAIMLNNFVDPQ GQIRDDCLYVQVR			Carbamidomethyl (C)[1]		Mascot

8	Uncharacterized protein OS=Alicyclobacillus acidoterrestris (strain ATCC 49025 / DSM 3922 / CIP 106132 / NCIMB 13137 / GD3B) GN=N007_09405 PE=4 SV=1	tr|T0BWK5|T0BWK5_ALIAG	27204.3	5.82	3	14	0

Peptide Information

Calc. Mass	Obsrv. Mass	± da	± ppm	Start Seq.	End Seq.	Sequence	Ion Score	C. I. %	Modification	Rank	Result Type
2593.2678	2593.4502	0.1824	70	99	120	EMAIDVANKTYEFSQFMV NVLK			Oxidation (M)[2]		Mascot
3517.8118	3517.4744	-0.3374	-96	3	34	VSLFITCLVDNIFPNVGVA MTRILQAHGCDVR			Oxidation (M)[20]		Mascot
3956.7803	3957.0488	0.2685	68	182	219	MGDISGAMVDEKAQHVT ETGAEVLVGTDMGCLMN IGGR			Oxidation (M)[1,8,29,33]		Mascot

9	Uncharacterized protein OS=Alicyclobacillus acidoterrestris (strain ATCC 49025 / DSM 3922 / CIP 106132 / NCIMB 13137 / GD3B) GN=N007_11770 PE=4 SV=1	tr|T0BUT4|T0BUT4_ALIAG	34235.4	9.68	4	14	0

Peptide Information

Calc. Mass	Obsrv. Mass	± da	± ppm	Start Seq.	End Seq.	Sequence	Ion Score	C. I. %	Modification	Rank	Result Type
1571.0295	1571.0823	0.0528	34	133	146	YALLTLVLLIGILR					Mascot
2598.3579	2598.5994	0.2415	93	60	80	QFGLRLTYVFLTSMFFNA WLK			Oxidation (M)[14]		Mascot
3339.7957	3339.6245	-0.1712	-51	241	271	ICAGVIGVGGMVAIQWGL QSTESVLPWLLLR			Carbamidomethyl (C)[2], Oxidation (M)[11]		Mascot
3799.9248	3800.1902	0.2654	70	1	32	MATHTFPLPAGFGWQYH FILWIQSFHTPILDK					Mascot

10	PhoP family transcriptional regulator OS=Alicyclobacillus acidoterrestris (strain ATCC 49025 / DSM 3922 / CIP 106132 / NCIMB 13137 / GD3B) GN=N007_13200 PE=4 SV=1	tr|T0BRG0|T0BRG0_ALIAG	27473.2	5.11	4	14	0

Peptide Information

Calc. Mass	Obsrv. Mass	± da	± ppm	Start Seq.	End Seq.	Sequence	Ion Score	C. I. %	Modification	Rank	Result Type

827.3781	827.4486	0.0705	85	235	242	FTEAATTS		Mascot
2452.2107	2452.0076	-0.2031	-83	48	68	FDLVVLDLMLPGMDGMEI CRK	Carbamidomethyl (C)[19]	Mascot
2698.2708	2698.1753	-0.0955	-35	45	67	ADRFDLVVLDLMLPGMD GMEICR	Carbamidomethyl (C)[22], Oxidation (M)[12,16]	Mascot
2777.3879	2777.2996	-0.0883	-32	85	109	DEEVDLVLGLELGADDYV TKPFSPR		Mascot

Gel Idx/Pos	238/J14	Instr./Gel Origin	BA2151/full sequence test 20150515	Process Status	Analysis Succeeded
Plate [#] Name	[3] full sequence test 20150515	Instrument Sample Name		Spectra	11

Rank	Protein Name	Species	Accession No.	Protein MW	Protein PI	Pep. Count	Protein Score	Protein Score C. I. %	Total Ion Score	Total Ion C. I. %
1	Uncharacterized protein OS=Alicyclobacillus acidoterrestris (strain ATCC 49025 / DSM 3922 / CIP 106132 / NCIMB 13137 / GD3B) GN=N007_08970 PE=4 SV=1		tr|T0BYX4|T0BYX 4_ALIAG	114313.8	4.01	6	129	100	129	100

Peptide Information

Calc. Mass	Obsrv. Mass	± da	± ppm	Start Seq.	End Seq.	Sequence	Ion Score	C. I. %	Modification	Rank	Result Type
807.4359	807.4375	0.0016	2	121	127	FNTQVAK					Mascot
935.5309	935.5319	0.001	1	120	127	KFNTQVAK					Mascot
1254.6035	1254.6089	0.0054	4	46	56	VLSNPYEMTGK			Oxidation (M)[8]		Mascot
1254.6035	1254.6089	0.0054	4	46	56	VLSNPYEMTGK	21	96.061	Oxidation (M)[8]		Mascot
1403.7278	1403.6882	-0.0396	-28	121	134	FNTQVAKDPAGGAK					Mascot
2254.0815	2254.0669	-0.0146	-6	57	76	DSGNTTGFFPIYYFNQAL AK					Mascot
2254.0815	2254.0669	-0.0146	-6	57	76	DSGNTTGFFPIYYFNQAL AK	108	100			Mascot
2361.2483	2361.1375	-0.1108	-47	35	56	ASQLPIVVNGKVLSNPYE MTGK			Oxidation (M)[19]		Mascot

Rank	Protein Name	Species	Accession No.	Protein MW	Protein PI	Pep. Count	Protein Score	Protein Score C. I. %	Total Ion Score	Total Ion C. I. %
2	6-phosphogluconate dehydrogenase OS=Alicyclobacillus acidoterrestris (strain ATCC 49025 / DSM 3922 / CIP 106132 / NCIMB 13137 / GD3B) GN=N007_01705 PE=4 SV=1		tr|T0CYB3|T0CYB 3_ALIAG	32612.2	5.57	10	42	74.707		

Peptide Information

Calc. Mass	Obsrv. Mass	± da	± ppm	Start Seq.	End Seq.	Sequence	Ion Score	C. I. %	Modification	Rank	Result Type
875.4985	875.5075	0.009	10	144	150	TIEPLFR					Mascot
1086.5327	1086.5082	-0.0245	-23	288	297	NQFGGHAVEK					Mascot
1284.6543	1284.6001	-0.0542	-42	100	110	ESIRHAEDLSK					Mascot
1412.6805	1412.6833	0.0028	2	151	163	DTAVPNGYVYTGR					Mascot
1433.6544	1433.665	0.0106	7	270	281	YRSEETDTFTGK					Mascot
1439.6987	1439.7089	0.0102	7	216	227	GWLMELTESAFR					Mascot
1455.6937	1455.718	0.0243	17	216	227	GWLMELTESAFR			Oxidation (M)[4]		Mascot
1654.7352	1654.7344	-0.0008	0	129	143	HGACYMIGGDAEVFK			Carbamidomethyl (C)[4]		Mascot
1670.7302	1670.8114	0.0812	49	129	143	HGACYMIGGDAEVFK			Carbamidomethyl (C)[4], Oxidation (M)[6]		Mascot
1688.8701	1688.8802	0.0101	6	39	56	LAGEGATGASSIADLVEK					Mascot

Calc. Mass	Obsrv. Mass	± da	± ppm	Start Seq.	End Seq.	Sequence	Ion Score	C. I. %	Modification	Rank	Result Type
1870.8719	1870.8649	-0.007	-4	111	128	HGIYFFDVGTSGGTEGAR					Mascot
1870.8719	1870.8649	-0.007	-4	111	128	HGIYFFDVGTSGGTEGAR					Mascot
2254.2039	2254.0669	-0.137	-61	39	61	LAGEGATGASSIADLVEKLQAPR					Mascot
2254.2039	2254.0669	-0.137	-61	39	61	LAGEGATGASSIADLVEKLQAPR					Mascot

3	Uncharacterized protein OS=Alicyclobacillus acidoterrestris (strain ATCC 49025 / DSM 3922 / CIP 106132 / NCIMB 13137 / GD3B) GN=N007_08075 PE=4 SV=1	tr|T0BNW5|T0BNW5_ALIAG	10562.2	5.64	5	32	0

Peptide Information

Calc. Mass	Obsrv. Mass	± da	± ppm	Start Seq.	End Seq.	Sequence	Ion Score	C. I. %	Modification	Rank	Result Type
1159.6681	1159.6129	-0.0552	-48	2	12	EAAQKIALTSK					Mascot
1306.7035	1306.5764	-0.1271	-97	1	12	MEAAQKIALTSK			Oxidation (M)[1]		Mascot
1502.709	1502.7416	0.0326	22	78	89	SLLEMIRQCHES			Carbamidomethyl (C)[9]		Mascot
1968.9927	1968.9344	-0.0583	-30	29	45	QWPIDQLALYPSSTGHR					Mascot
2254.1365	2254.0669	-0.0696	-31	27	45	ERQWPIDQLALYPSSTGHR					Mascot
2254.1365	2254.0669	-0.0696	-31	27	45	ERQWPIDQLALYPSSTGHR					Mascot

4	Kynurenine formamidase OS=Alicyclobacillus acidoterrestris (strain ATCC 49025 / DSM 3922 / CIP 106132 / NCIMB 13137 / GD3B) GN=kynB PE=3 SV=1	tr|T0BPC6|T0BPC6_ALIAG	24952.8	5.48	7	28	0

Peptide Information

Calc. Mass	Obsrv. Mass	± da	± ppm	Start Seq.	End Seq.	Sequence	Ion Score	C. I. %	Modification	Rank	Result Type
935.4404	935.5319	0.0915	98	4	10	WMDISQR					Mascot
1086.6167	1086.5082	-0.1085	-100	160	169	ALPAHHALTR					Mascot
1327.6134	1327.7081	0.0947	71	1	10	MTKWMDISQR			Oxidation (M)[1,5]		Mascot
1557.8635	1557.8386	-0.0249	-16	66	79	VLELDVNLYVGPAR					Mascot
1581.8734	1581.7703	-0.1031	-65	145	159	LLGLDLPSVDPLDSK					Mascot
2209.1904	2209.0479	-0.1425	-65	122	140	VFPEKIPEIHPDLAEYLAK					Mascot
2359.0952	2359.1589	0.0637	27	207	228	AILRPMGPCNGAIEACGDERGV			Carbamidomethyl (C)[9,16], Oxidation (M)[6]		Mascot

5	Uncharacterized protein OS=Alicyclobacillus acidoterrestris (strain ATCC 49025 / DSM 3922 / CIP 106132 / NCIMB 13137 / GD3B) GN=N007_21200 PE=4 SV=1	tr|T0BYP8|T0BYP8_ALIAG	20032.1	6.11	6	27	0

Peptide Information

Calc. Mass	Obsrv. Mass	± da	± ppm	Start	End	Sequence	Ion	C. I. %	Modification	Rank	Result Type

				Seq.	Seq.		Score				
1278.6688	1278.578	-0.0908	-71	152	162	YDAAELQTIVR					Mascot
1499.6724	1499.7028	0.0304	20	163	176	FLSELAEFGMSPGD					Mascot
1502.8802	1502.7416	-0.1386	-92	17	29	ASLHSEIHKLIVR					Mascot
1688.933	1688.8802	-0.0528	-31	75	91	LGPVNGATVASYLGITR					Mascot
1774.7549	1774.8737	0.1188	67	2	16	FSSEENNMSNETTIR			Oxidation (M)[8]		Mascot
2378.1306	2378.1877	0.0571	24	30	48	VHHSFEMHDQQELKQLAER			Oxidation (M)[7]		Mascot

6	Uncharacterized protein OS=Alicyclobacillus acidoterrestris (strain ATCC 49025 / DSM 3922 / CIP 106132 / NCIMB 13137 / GD3B) GN=N007_20185 PE=3 SV=1	tr|T0DT25|T0DT25_ALIAG	15340.4	5.14	5	24	0

Peptide Information

Calc. Mass	Obsrv. Mass	± da	± ppm	Start Seq.	End Seq.	Sequence	Ion Score	C. I. %	Modification	Rank	Result Type
1254.5573	1254.6089	0.0516	41	1	10	MDEYGQGIWR					Mascot
1254.5573	1254.6089	0.0516	41	1	10	MDEYGQGIWR					Mascot
1412.5482	1412.6833	0.1351	96	76	87	MDDVSDDSVEER			Oxidation (M)[1]		Mascot
1433.6995	1433.665	-0.0345	-24	58	68	CIDYFRSAFVR			Carbamidomethyl (C)[1]		Mascot
1654.851	1654.7344	-0.1166	-70	42	55	EGSSMKTWLFVIEK					Mascot
1670.8458	1670.8114	-0.0344	-21	42	55	EGSSMKTWLFVIEK			Oxidation (M)[5]		Mascot
2179.9773	2179.9819	0.0046	2	76	94	MDDVSDDSVEERVIASEQR					Mascot

7	Uncharacterized protein OS=Alicyclobacillus acidoterrestris (strain ATCC 49025 / DSM 3922 / CIP 106132 / NCIMB 13137 / GD3B) GN=N007_18850 PE=4 SV=1	tr|T0CKG1|T0CKG1_ALIAG	11622	11.75	4	22	0

Peptide Information

Calc. Mass	Obsrv. Mass	± da	± ppm	Start Seq.	End Seq.	Sequence	Ion Score	C. I. %	Modification	Rank	Result Type
1403.704	1403.6882	-0.0158	-11	57	67	HNQRIADWHAR					Mascot
1416.7317	1416.647	-0.0847	-60	1	10	MLNYIQRWHR					Mascot
1416.7317	1416.647	-0.0847	-60	1	10	MLNYIQRWHR					Mascot
1487.8077	1487.6875	-0.1202	-81	68	81	HLAEVLAGTNGLHR					Mascot
1757.9293	1757.8575	-0.0718	-41	16	31	IAATLGLPEDLSHHQR					Mascot

8	Uncharacterized protein OS=Alicyclobacillus acidoterrestris (strain ATCC 49025 / DSM 3922 / CIP 106132 / NCIMB 13137 / GD3B) GN=N007_09240 PE=4 SV=1	tr|T0D608|T0D608_ALIAG	46367.3	5.75	8	21	0

Peptide Information

Calc. Mass	Obsrv. Mass	± da	± ppm	Start Seq.	End Seq.	Sequence	Ion Score	C. I. %	Modification	Rank	Result Type
875.4846	875.5075	0.0229	26	387	393	HIIEAHR					Mascot
1276.5596	1276.5902	0.0306	24	174	185	MCSLDVMHAGGR					Mascot
1413.7076	1413.6759	-0.0317	-22	2	14	SLAMNKSSMTISK			Oxidation (M)[4]		Mascot
1429.7025	1429.7405	0.038	27	2	14	SLAMNKSSMTISK			Oxidation (M)[4,9]		Mascot
1429.7025	1429.7405	0.038	27	2	14	SLAMNKSSMTISK			Oxidation (M)[4,9]		Mascot
1467.7472	1467.7205	-0.0267	-18	8	20	SSMTISKSTIEQR					Mascot
1694.9297	1694.934	0.0043	3	204	219	NRSLASLSAGIAHEIR					Mascot
1863.7969	1863.9359	0.139	75	174	190	MCSLDVMHAGGRCAPDK			Carbamidomethyl (C)[2], Oxidation (M)[1]		Mascot
2293.0918	2293.0581	-0.0337	-15	154	173	TYDEAHLHSMLGTAVYAIER			Oxidation (M)[10]		Mascot
2361.0864	2361.1375	0.0511	22	355	374	DNGCGISDMHHLFRPFQTTK			Carbamidomethyl (C)[4]		Mascot

9	Uncharacterized protein OS=Alicyclobacillus acidoterrestris (strain ATCC 49025 / DSM 3922 / CIP 106132 / NCIMB 13137 / GD3B) GN=N007_17860 PE=4 SV=1	tr|T0BJB1|T0BJB1_ALIAG	6227.2	6.54	3	20	0

Peptide Information

Calc. Mass	Obsrv. Mass	± da	± ppm	Start Seq.	End Seq.	Sequence	Ion Score	C. I. %	Modification	Rank	Result Type
1525.8009	1525.6621	-0.1388	-91	20	32	YYTDKGLVPSVQR					Mascot
1581.8456	1581.7703	-0.0753	-48	25	38	GLVPSVQRDINNNR					Mascot
1884.916	1884.9349	0.0189	10	10	24	MLDLTEHTVRYYTDK					Mascot

10	Uncharacterized protein OS=Alicyclobacillus acidoterrestris (strain ATCC 49025 / DSM 3922 / CIP 106132 / NCIMB 13137 / GD3B) GN=N007_09960 PE=4 SV=1	tr|T0BXM2|T0BXM2_ALIAG	13028.6	5.63	4	20	0

Peptide Information

Calc. Mass	Obsrv. Mass	± da	± ppm	Start Seq.	End Seq.	Sequence	Ion Score	C. I. %	Modification	Rank	Result Type
820.4346	820.4651	0.0305	37	40	46	RMVVGDK			Oxidation (M)[2]		Mascot
829.3322	829.3961	0.0639	77	111	117	AQHSEEE					Mascot
1884.8796	1884.9349	0.0553	29	12	28	ADGVPTIEMAQSYPYSR					Mascot
2359.2041	2359.1589	-0.0452	-19	74	93	LEDRLHSFELVAEAFGLQER					Mascot

Gel Idx/Pos	239/J15	Instr./Gel Origin	BA2151/full sequence test 20150515	Process Status	Analysis Succeeded
Plate [#] Name	[3] full sequence test 20150515	Instrument Sample Name		Spectra	11

Rank	Protein Name	Species	Accession No.	Protein MW	Protein PI	Pep. Count	Protein Score	Protein Score C. I. %	Total Ion Score	Total Ion C. I. %
1	Glyceraldehyde-3-phosphate dehydrogenase OS=Alicyclobacillus acidoterrestris (strain ATCC 49025 / DSM 3922 / CIP 106132 / NCIMB 13137 / GD3B) GN=gapA PE=3 SV=1		tr|T0BM94|T0BM94_ALIAG	35675.5	5.78	11	103	100	59	100

Peptide Information

Calc. Mass	Obsrv. Mass	± da	± ppm	Start Seq.	End Seq.	Sequence	Ion Score	C. I. %	Modification	Rank	Result Type
1046.6721	1046.673	0.0009	1	216	225	AVGLVLPHLK					Mascot
1046.6721	1046.673	0.0009	1	216	225	AVGLVLPHLK					Mascot
1134.6041	1134.6122	0.0081	7	164	173	VIDDVFGIEK					Mascot
1176.6624	1176.635	-0.0274	-23	323	333	VVDLANFIASK					Mascot
1212.6041	1212.6064	0.0023	2	188	197	ILDLPHSDMR			Oxidation (M)[9]		Mascot
1348.6954	1348.6803	-0.0151	-11	251	263	STATVESVNEALK					Mascot
1368.7053	1368.708	0.0027	2	188	198	ILDLPHSDMRR			Oxidation (M)[9]		Mascot
1419.7478	1419.7465	-0.0013	-1	272	284	GILAYSDEPLVSR					Mascot
1419.7478	1419.7465	-0.0013	-1	272	284	GILAYSDEPLVSR	20	96.498			Mascot
1622.7592	1622.7515	-0.0077	-5	174	187	GLMTTVHSYTNDQR					Mascot
1638.7542	1638.7463	-0.0079	-5	174	187	GLMTTVHSYTNDQR			Oxidation (M)[3]		Mascot
1680.9531	1680.9496	-0.0035	-2	235	250	VPTPNVSLVDLTAQVK					Mascot
1680.9531	1680.9496	-0.0035	-2	235	250	VPTPNVSLVDLTAQVK	39	99.949			Mascot
1757.8916	1757.7501	-0.1415	-80	24	39	NENIEIVAVNDLTDAK					Mascot
1758.7871	1758.7732	-0.0139	-8	309	322	VVAWYDNEWGYSNR					Mascot

Rank	Protein Name	Species	Accession No.	Protein MW	Protein PI	Pep. Count	Protein Score	Protein Score C. I. %	Total Ion Score	Total Ion C. I. %
2	Thioredoxin OS=Alicyclobacillus acidoterrestris (strain ATCC 49025 / DSM 3922 / CIP 106132 / NCIMB 13137 / GD3B) GN=N007_12660 PE=3 SV=1		tr|T0BS27|T0BS27_ALIAG	12495.1	4.6	5	28	0		

Peptide Information

Calc. Mass	Obsrv. Mass	± da	± ppm	Start Seq.	End Seq.	Sequence	Ion Score	C. I. %	Modification	Rank	Result Type
1368.7019	1368.708	0.0061	4	45	56	YAGDFSIVRANR					Mascot
1798.8202	1798.7194	-0.1008	-56	3	18	QITSNEEYAQSTQSGR					Mascot
1818.7649	1818.7791	0.0142	8	19	32	VMVEFYANWCPDCK			Carbamidomethyl (C)[10,13]		Mascot
1876.818	1876.8118	-0.0062	-3	19	33	VMVEFYANWCPDCKR			Oxidation (M)[2]		Mascot

Calc. Mass	Obsrv. Mass	± da	± ppm	Start Seq.	End Seq.	Sequence	Ion Score	C. I. %	Modification	Rank	Result Type
1926.9152	1926.9475	0.0323	17	2	18	KQITSNEEYAQSTQSGR					Mascot

3	Uncharacterized protein OS=Alicyclobacillus acidoterrestris (strain ATCC 49025 / DSM 3922 / CIP 106132 / NCIMB 13137 / GD3B) GN=N007_04605 PE=4 SV=1	tr|T0C596|T0C596_ALIAG	32862.4	8.42	8	24	0

Peptide Information

Calc. Mass	Obsrv. Mass	± da	± ppm	Start Seq.	End Seq.	Sequence	Ion Score	C. I. %	Modification	Rank	Result Type
1419.7413	1419.7465	0.0052	4	177	188	GRVYLPLEEMGR					Mascot
1419.7413	1419.7465	0.0052	4	177	188	GRVYLPLEEMGR					Mascot
1433.6915	1433.7687	0.0772	54	1	12	MNLTQAYKYCAK					Mascot
1433.6915	1433.7687	0.0772	54	1	12	MNLTQAYKYCAK					Mascot
1457.8145	1457.7167	-0.0978	-67	156	168	DMGIALQLTNILR					Mascot
1798.8905	1798.7194	-0.1711	-95	201	215	TTPAFYALMNEQTRR					Mascot
1814.8854	1814.759	-0.1264	-70	201	215	TTPAFYALMNEQTRR			Oxidation (M)[9]		Mascot
1818.9531	1818.7791	-0.174	-96	225	239	NLFPLVESDSLRCLR			Carbamidomethyl (C)[13]		Mascot
1827.9575	1827.7888	-0.1687	-92	95	110	FHIPKEPFAGLLDGMR					Mascot
1843.9524	1843.8613	-0.0911	-49	95	110	FHIPKEPFAGLLDGMR			Oxidation (M)[15]		Mascot
2383.0669	2383.1455	0.0786	33	127	146	YCEYVAGTIGQMCVHIFGYR			Carbamidomethyl (C)[2], Oxidation (M)[12]		Mascot
2500.313	2500.2075	-0.1055	-42	147	168	DPAAFQWARDMGIALQLTNILR					Mascot

4	Uncharacterized protein OS=Alicyclobacillus acidoterrestris (strain ATCC 49025 / DSM 3922 / CIP 106132 / NCIMB 13137 / GD3B) GN=N007_01020 PE=4 SV=1	tr|T0BPY7|T0BPY7_ALIAG	17213.1	6.62	6	23	0

Peptide Information

Calc. Mass	Obsrv. Mass	± da	± ppm	Start Seq.	End Seq.	Sequence	Ion Score	C. I. %	Modification	Rank	Result Type
1046.5775	1046.673	0.0955	91	76	84	RMETAGLLR					Mascot
1046.6205	1046.673	0.0525	50	94	102	TVRISLTEK					Mascot
1467.7764	1467.7123	-0.0641	-44	63	75	LYLDSGTLTPMLK			Oxidation (M)[11]		Mascot
1623.8774	1623.7616	-0.1158	-71	63	76	LYLDSGTLTPMLKR			Oxidation (M)[11]		Mascot
1628.8102	1628.8269	0.0167	10	9	22	LDNQLCFSVYALSR					Mascot
2060.0845	2059.9551	-0.1294	-63	58	75	HLGERLYLDSGTLTPMLK			Oxidation (M)[16]		Mascot

5	Glutamate--tRNA ligase OS=Alicyclobacillus acidoterrestris (strain ATCC 49025 / DSM 3922 / CIP 106132 / NCIMB 13137 / GD3B) GN=gltX PE=3 SV=1	tr|T0BZR7|T0BZR7_ALIAG	54513.7	5.19	9	22	0

Peptide Information

Calc. Mass	Obsrv. Mass	± da	± ppm	Start Seq.	End Seq.	Sequence	Ion Score	C. I. %	Modification	Rank	Result Type
1046.5928	1046.673	0.0802	77	439	447	GRALFMPVR					Mascot
1046.5928	1046.673	0.0802	77	439	447	GRALFMPVR					Mascot
1070.5854	1070.64	0.0546	51	32	40	RHGGEFVLR					Mascot
1148.5807	1148.5939	0.0132	11	123	132	EGRVPQYSGR					Mascot
1198.5708	1198.6332	0.0624	52	83	91	CMERLDLYR					Mascot
1416.7264	1416.6427	-0.0837	-59	328	340	ALAPDEATAMVRR			Oxidation (M)[10]		Mascot
1575.8391	1575.7435	-0.0956	-61	7	21	FAPSPTGHLHIGSVR					Mascot
1638.8698	1638.7463	-0.1235	-75	157	170	VPENQEIVVDDLIR					Mascot
1858.9043	1858.8013	-0.103	-55	33	48	HGGEFVLRIEDTDTNR					Mascot
2379.1826	2379.124	-0.0586	-25	404	425	EVVAAYLALAKADDEWTAEASR					Mascot

6 Uncharacterized protein (Fragment) OS=Alicyclobacillus acidoterrestris (strain ATCC 49025 / DSM 3922 / CIP 106132 / NCIMB 13137 / GD3B) GN=N007_18230 PE=4 SV=1 — tr|T0CKS1|T0CKS1_ALIAG — 9411.8 — 6.71 — 4 — 21 — 0

Peptide Information

Calc. Mass	Obsrv. Mass	± da	± ppm	Start Seq.	End Seq.	Sequence	Ion Score	C. I. %	Modification	Rank	Result Type
1622.806	1622.7515	-0.0545	-34	39	52	LFDDESLNWLTAAK					Mascot
1858.9116	1858.8013	-0.1103	-59	10	24	MLDLTEHTVRYHTDK					Mascot
1864.9586	1864.8998	-0.0588	-32	66	81	RFVDLCLEGVSTIQER					Mascot
1874.9066	1874.7992	-0.1074	-57	10	24	MLDLTEHTVRYHTDK			Oxidation (M)[1]		Mascot
2349.147	2349.1394	-0.0076	-3	33	52	DINNNRLFDDESLNWLTAAK					Mascot

7 Uncharacterized protein OS=Alicyclobacillus acidoterrestris (strain ATCC 49025 / DSM 3922 / CIP 106132 / NCIMB 13137 / GD3B) GN=N007_10385 PE=4 SV=1 — tr|T0CZC1|T0CZC1_ALIAG — 46981.9 — 5.64 — 8 — 20 — 0

Peptide Information

Calc. Mass	Obsrv. Mass	± da	± ppm	Start Seq.	End Seq.	Sequence	Ion Score	C. I. %	Modification	Rank	Result Type
1070.6317	1070.64	0.0083	8	413	421	DLVVARIER					Mascot
1457.6625	1457.7167	0.0542	37	284	295	QHMDLTASMVHR			Oxidation (M)[3,9]		Mascot
1467.7009	1467.7123	0.0114	8	298	310	HTESDITMHGALR					Mascot
1575.8854	1575.7435	-0.1419	-90	114	127	HESLVKEHLASVVK					Mascot
1638.7952	1638.7463	-0.0489	-30	284	297	QHMDLTASMVHRGR					Mascot
1680.8235	1680.9496	0.1261	75	296	310	GRHTESDITMHGALR					Mascot

Calc. Mass	Obsrv. Mass	± da	± ppm	Start Seq.	End Seq.	Sequence	Ion Score	C. I. %	Modification	Rank	Result Type
1680.8235	1680.9496	0.1261	75	296	310	GRHTESDITMHGALR					Mascot
1814.8953	1814.759	-0.1363	-75	347	362	ADAIPMLLIDENDVNR			Oxidation (M)[6]		Mascot
2366.1379	2366.1794	0.0415	18	363	383	CGHAASVGKIDPNQVYYLMSR			Carbamidomethyl (C)[1]		Mascot

8	Uncharacterized protein OS=Alicyclobacillus acidoterrestris (strain ATCC 49025 / DSM 3922 / CIP 106132 / NCIMB 13137 / GD3B) GN=N007_21470 PE=4 SV=1	tr|T0DGG4|T0DGG4_ALIAG	16906.9	10.57	5	20	0

Peptide Information

Calc. Mass	Obsrv. Mass	± da	± ppm	Start Seq.	End Seq.	Sequence	Ion Score	C. I. %	Modification	Rank	Result Type
1176.5367	1176.635	0.0983	84	1	10	MTYHGAFGHR					Mascot
1467.7737	1467.7123	-0.0614	-42	139	152	AVASLTLHGPCVSR			Carbamidomethyl (C)[11]		Mascot
1547.8098	1547.7346	-0.0752	-49	32	46	AVTSVAAMQLVEQGK			Oxidation (M)[8]		Mascot
1573.762	1573.7992	0.0372	24	2	15	TYHGAFGHRAVDSR					Mascot
1575.8126	1575.7435	-0.0691	-44	65	78	VLEGFSESGEPRLR					Mascot

9	Uncharacterized protein OS=Alicyclobacillus acidoterrestris (strain ATCC 49025 / DSM 3922 / CIP 106132 / NCIMB 13137 / GD3B) GN=N007_10645 PE=3 SV=1	tr|T0CZG2|T0CZG2_ALIAG	29268.3	5.87	6	20	0

Peptide Information

Calc. Mass	Obsrv. Mass	± da	± ppm	Start Seq.	End Seq.	Sequence	Ion Score	C. I. %	Modification	Rank	Result Type
1134.6001	1134.6122	0.0121	11	51	62	LVSGLESASSGK					Mascot
1623.787	1623.7616	-0.0254	-16	164	175	LEMQRLIEDMWK			Oxidation (M)[3,10]		Mascot
1628.8322	1628.8269	-0.0053	-3	198	211	VILIEHGMIAMDMR					Mascot
1778.9113	1778.7335	-0.1778	-100	235	249	FFAPTVEQHSLDFIK					Mascot
1804.8752	1804.7705	-0.1047	-58	221	234	DTQFLYYENTILER					Mascot
2379.3567	2379.124	-0.2327	-98	141	163	ALVAAPKMLLLDEPLGALDALTK			Oxidation (M)[8]		Mascot

10	Uncharacterized protein OS=Alicyclobacillus acidoterrestris (strain ATCC 49025 / DSM 3922 / CIP 106132 / NCIMB 13137 / GD3B) GN=N007_01415 PE=4 SV=1	tr|T0CZR5|T0CZR5_ALIAG	42030.7	6.45	7	20	0

Peptide Information

Calc. Mass	Obsrv. Mass	± da	± ppm	Start Seq.	End Seq.	Sequence	Ion Score	C. I. %	Modification	Rank	Result Type
1348.7117	1348.6803	-0.0314	-23	47	57	MIVMPVPYNLR			Oxidation (M)[1]		Mascot
1573.8115	1573.7992	-0.0123	-8	157	169	RDTQCINALDVLR			Carbamidomethyl (C)[5]		Mascot

1798.8314	1798.7194	-0.112	-62	173	188	DGLDQHASISAEDRER		Mascot
1825.9039	1825.8134	-0.0905	-50	170	186	AIKDGLDQHASISAEDR		Mascot
1862.9171	1862.7882	-0.1289	-69	206	221	TEVQKAFVYSFEESAK		Mascot
2366.1194	2366.1794	0.06	25	13	32	THHLTDVEMIVAHTNEAE YR		Mascot
2500.2178	2500.2075	-0.0103	-4	222	241	TLLDNYLDNVEAYCNWQ KLK	Carbamidomethyl (C)[14]	Mascot

Gel Idx/Pos	240/J16	Instr./Gel Origin	BA2151/full sequence test 20150515	Process Status	Analysis Succeeded
Plate [#] Name	[3] full sequence test 20150515	Instrument Sample Name		Spectra	11

Rank	Protein Name	Species	Accession No.	Protein MW	Protein PI	Pep. Count	Protein Score	Protein Score C. I. %	Total Ion Score	Total Ion C. I. %
1	Uncharacterized protein OS=Alicyclobacillus acidoterrestris (strain ATCC 49025 / DSM 3922 / CIP 106132 / NCIMB 13137 / GD3B) GN=N007_00440 PE=4 SV=1		tr|T0C5G8|T0C5G8_ALIAG	63753	4.64	9	174	100	155	100

Peptide Information

Calc. Mass	Obsrv. Mass	± da	± ppm	Start Seq.	End Seq.	Sequence	Ion Score	C. I. %	Modification	Rank	Result Type
1008.4996	1008.5047	0.0051	5	386	394	NLDPSATYK					Mascot
1140.6049	1140.605	0.0001	0	443	451	LDIHEVTWK					Mascot
1140.6049	1140.605	0.0001	0	443	451	LDIHEVTWK	47	99.989			Mascot
1680.8262	1680.8179	-0.0083	-5	428	442	DDQAFQSMLSQIGIK					Mascot
1696.8212	1696.8085	-0.0127	-7	428	442	DDQAFQSMLSQIGIK			Oxidation (M)[8]		Mascot
1744.9269	1744.9165	-0.0104	-6	443	456	LDIHEVTWKDFLTK					Mascot
1744.9269	1744.9165	-0.0104	-6	443	456	LDIHEVTWKDFLTK	87	100			Mascot
1776.0128	1775.8817	-0.1311	-74	201	216	ILAMPFLSAVDPKFVK					Mascot
1808.9211	1808.9124	-0.0087	-5	427	442	KDDQAFQSMLSQIGIK					Mascot
1808.9211	1808.9124	-0.0087	-5	427	442	KDDQAFQSMLSQIGIK	22	96.2			Mascot
1824.916	1824.9042	-0.0118	-6	427	442	KDDQAFQSMLSQIGIK			Oxidation (M)[9]		Mascot
2074.9539	2074.9282	-0.0257	-12	125	142	YSNGDPMVASDFVFELQR					Mascot
2391.0161	2391.0056	-0.0105	-4	406	426	EAGYANGFTAEYWNENNPTDK					Mascot
2548.166	2548.1438	-0.0222	-9	121	142	DDVKYSNGDPMVASDFVFELQR			Oxidation (M)[11]		Mascot

Rank	Protein Name	Species	Accession No.	Protein MW	Protein PI	Pep. Count	Protein Score	Protein Score C. I. %	Total Ion Score	Total Ion C. I. %
2	Uncharacterized protein OS=Alicyclobacillus acidoterrestris (strain ATCC 49025 / DSM 3922 / CIP 106132 / NCIMB 13137 / GD3B) GN=N007_10530 PE=4 SV=1		tr|T0BV11|T0BV11_ALIAG	41296.8	8.04	10	36	0		

Peptide Information

Calc. Mass	Obsrv. Mass	± da	± ppm	Start Seq.	End Seq.	Sequence	Ion Score	C. I. %	Modification	Rank	Result Type
1140.6736	1140.605	-0.0686	-60	35	44	AKTIPNEVLR					Mascot
1140.6736	1140.605	-0.0686	-60	35	44	AKTIPNEVLR					Mascot
1186.644	1186.62	-0.024	-20	294	303	QISYGGRIHR					Mascot
1717.9166	1717.8477	-0.0689	-40	107	122	LHDAMRDAVVPGAPLR					Mascot

Calc. Mass	Obsrv. Mass	± da	± ppm	Start Seq.	End Seq.	Sequence	Ion Score	C. I. %	Modification	Rank	Result Type
1765.9385	1765.8724	-0.0661	-37	113	128	DAVVPGAPLRAYYAFR					Mascot
1797.9429	1797.9122	-0.0307	-17	1	15	MTSATPTRPTRFLYR					Mascot
1813.9379	1813.895	-0.0429	-24	1	15	MTSATPTRPTRFLYR			Oxidation (M)[1]		Mascot
1824.8698	1824.9042	0.0344	19	168	181	YCELQEHKHIQPDK			Carbamidomethyl (C)[2]		Mascot
1954.0468	1954.0848	0.038	19	304	320	FIVQGLLGMYLSDDKVR					Mascot
2044.9773	2044.9769	-0.0004	0	335	351	FGPTACMFYFATVVYRR			Oxidation (M)[7]		Mascot
2078.9026	2078.9033	0.0007	0	262	279	EGDFNFVACYPDAHTAHK			Carbamidomethyl (C)[9]		Mascot
2573.229	2573.0789	-0.1501	-58	330	350	HMIYRFGPTACMFYFATVVYR					Mascot

3	Uncharacterized protein OS=Alicyclobacillus acidoterrestris (strain ATCC 49025 / DSM 3922 / CIP 106132 / NCIMB 13137 / GD3B) GN=N007_00890 PE=4 SV=1	tr|T0C5P9|T0C5P9_ALIAG	11703.7	4.9	6	30	0

Peptide Information

Calc. Mass	Obsrv. Mass	± da	± ppm	Start Seq.	End Seq.	Sequence	Ion Score	C. I. %	Modification	Rank	Result Type
1024.4988	1024.4897	-0.0091	-9	2	9	MMMLLTER					Mascot
1171.5342	1171.5702	0.036	31	1	9	MMMMLLTER			Oxidation (M)[1]		Mascot
1954.9039	1955.0212	0.1173	60	1	16	MMMMLLTERCSHQLSK			Oxidation (M)[1]		Mascot
2002.8888	2002.9757	0.0869	43	1	16	MMMMLLTERCSHQLSK			Oxidation (M)[1,2,3,4]		Mascot
2002.8888	2002.9757	0.0869	43	1	16	MMMMLLTERCSHQLSK			Oxidation (M)[1,2,3,4]		Mascot
2014.9097	2014.9312	0.0215	11	50	67	ISEDECSVANGISFLMDK			Carbamidomethyl (C)[6]		Mascot
2018.8617	2018.9678	0.1061	53	27	45	LSAEYEGGGCGCSVAVQMK			Carbamidomethyl (C)[10,12], Oxidation (M)[18]		Mascot
2018.8617	2018.9678	0.1061	53	27	45	LSAEYEGGGCGCSVAVQMK			Carbamidomethyl (C)[10,12], Oxidation (M)[18]		Mascot
2059.9102	2059.9624	0.0522	25	1	16	MMMMLLTERCSHQLSK			Carbamidomethyl (C)[10], Oxidation (M)[1,2,3,4]		Mascot
2502.1057	2502.1021	-0.0036	-1	27	49	LSAEYEGGGCGCSVAVQMKVDER			Carbamidomethyl (C)[10,12]		Mascot
2518.1008	2518.095	-0.0058	-2	27	49	LSAEYEGGGCGCSVAVQMKVDER			Carbamidomethyl (C)[10,12], Oxidation (M)[18]		Mascot

4	Elongation factor G OS=Alicyclobacillus acidoterrestris (strain ATCC 49025 / DSM 3922 / CIP 106132 / NCIMB 13137 / GD3B) GN=fusA PE=3 SV=1	tr|T0BZT0|T0BZT0_ALIAG	76373.8	5.06	12	29	0

Peptide Information

Calc. Mass	Obsrv. Mass	± da	± ppm	Start Seq.	End Seq.	Sequence	Ion Score	C. I. %	Modification	Rank	Result Type
1743.837	1743.8937	0.0567	33	534	548	EYIPAVEEGIHEAMR					Mascot
1765.9041	1765.8724	-0.0317	-18	421	436	MGIALSKLAEEDPTFK			Oxidation (M)[1]		Mascot
1804.8939	1804.8743	-0.0196	-11	642	657	GFVPLSEMFGYTTSLR					Mascot

Calc. Mass	Obsrv. Mass	± da	± ppm	Start Seq.	End Seq.	Sequence	Ion Score	C. I. %	Modification	Rank	Result Type
1820.8888	1820.8567	-0.0321	-18	642	657	GFVPLSEMFGYTTSLR			Oxidation (M)[8]		Mascot
2002.0492	2001.9592	-0.09	-45	328	346	VYSGILESGSYVLNSTKGK					Mascot
2009.0491	2008.9982	-0.0509	-25	509	525	VIFEPLERGQGFVFENK					Mascot
2025.0587	2024.9662	-0.0925	-46	246	263	KGTVNVQLFPVLCGSSYR			Carbamidomethyl (C)[13]		Mascot
2056.9866	2056.9246	-0.062	-30	137	154	MDIIGADFLSCVEQMKTR					Mascot
2180.0588	2179.9968	-0.062	-28	469	487	VDCNVGMPQVAYRETITQR					Mascot
2392.1814	2391.9978	-0.1836	-77	605	625	VEVTVPEEYMGDILGDINSRR					Mascot
2408.1763	2408.0034	-0.1729	-72	605	625	VEVTVPEEYMGDILGDINSRR			Oxidation (M)[10]		Mascot
2520.2183	2520.0879	-0.1304	-52	131	152	IAYVNKMDIIGADFLSCVEQMK			Oxidation (M)[7,21]		Mascot
2520.2183	2520.0879	-0.1304	-52	131	152	IAYVNKMDIIGADFLSCVEQMK			Oxidation (M)[7,21]		Mascot
2525.1824	2525.1165	-0.0659	-26	183	204	AIIYTDDLGRQSEDTEIPADMR			Oxidation (M)[21]		Mascot
2534.2344	2534.113	-0.1214	-48	98	121	VLDGACAVFDAKGGVEPQSETVWR					Mascot

5 Uncharacterized protein OS=Alicyclobacillus acidoterrestris (strain ATCC 49025 / DSM 3922 / CIP 106132 / NCIMB 13137 / GD3B) GN=N007_04920 PE=4 SV=1 — tr|T0C5T9|T0C5T9_ALIAG — 48682 — 6.45 — 11 — 29 — 0

Peptide Information

Calc. Mass	Obsrv. Mass	± da	± ppm	Start Seq.	End Seq.	Sequence	Ion Score	C. I. %	Modification	Rank	Result Type
981.5186	981.4789	-0.0397	-40	16	23	MNQLKGYK					Mascot
1144.6208	1144.6001	-0.0207	-18	335	343	EIEIEEVRK					Mascot
1217.5757	1217.6757	0.1	82	108	118	EADQVAATQER					Mascot
1726.8971	1726.9038	0.0067	4	304	320	SIQSATHPLIGGTTTDK					Mascot
1765.8248	1765.8724	0.0476	27	54	68	ELQEWGLSVCMVAER			Oxidation (M)[11]		Mascot
1776.0126	1775.8817	-0.1309	-74	364	380	NQHKAIVLEKPAAASAK					Mascot
1806.8368	1806.8403	0.0035	2	163	179	TGVLYNFAAYAAEEGCK					Mascot
1820.8984	1820.8567	-0.0417	-23	108	124	EADQVAATQERTVSTSK					Mascot
1953.9198	1954.0848	0.165	84	232	247	YGFYMITAASREEMIR			Oxidation (M)[5]		Mascot
2074.9653	2074.9282	-0.0371	-18	287	302	LYMFGLYDQMQYVEFK					Mascot
2405.1191	2405.0122	-0.1069	-44	284	302	ADKLYMFGLYDQMQYVEFK			Oxidation (M)[6]		Mascot

6 Uncharacterized protein OS=Alicyclobacillus acidoterrestris (strain ATCC 49025 / DSM 3922 / CIP 106132 / NCIMB 13137 / GD3B) GN=N007_12160 PE=4 SV=1 — tr|T0BTK3|T0BTK3_ALIAG — 34323.8 — 8.22 — 9 — 28 — 0

Peptide Information

Calc. Mass	Obsrv. Mass	± da	± ppm	Start Seq.	End Seq.	Sequence	Ion Score	C. I. %	Modification	Rank	Result Type
832.504	832.5152	0.0112	13	207	214	FGILVGAR					Mascot
1680.9142	1680.8179	-0.0963	-57	251	264	SIYWIEMGIVARVK			Oxidation (M)[7]		Mascot
1717.9021	1717.8477	-0.0544	-32	35	48	VELYTRHGNIWTTK					Mascot
1775.868	1775.8817	0.0137	8	193	206	TMDTCILAWRQQPR			Carbamidomethyl (C)[5]		Mascot
1788.7786	1788.8661	0.0875	49	7	20	EEIFDSPEYCFQPK			Carbamidomethyl (C)[10]		Mascot
1797.8881	1797.9122	0.0241	13	282	295	FIFDKKPEECIWVS			Carbamidomethyl (C)[10]		Mascot
2023.8636	2023.9601	0.0965	48	160	176	EMDWEGTCAKHVDSPY R					Mascot
2039.8586	2039.9702	0.1116	55	160	176	EMDWEGTCAKHVDSPY R			Oxidation (M)[2]		Mascot
2225.1238	2225.1013	-0.0225	-10	215	234	FQSGLKALAWVENGFSD VEK					Mascot
2534.1765	2534.113	-0.0635	-25	1	20	MLLTMREEIFDSPEYCFQ PK			Carbamidomethyl (C)[16]		Mascot

7	1-(5-phosphoribosyl)-5-[(5-phosphoribosylamino)methylideneamino] imidazole-4-carboxamide isomerase OS=Alicyclobacillus acidoterrestris (strain ATCC 49025 / DSM 3922 / CIP 106132 / NCIMB 13137 / GD3B) GN=hisA PE=3 SV=1	tr|T0BSZ4|T0BSZ4_ALIAG	25524.1	4.97	7	27	0	

Peptide Information

Calc. Mass	Obsrv. Mass	± da	± ppm	Start Seq.	End Seq.	Sequence	Ion Score	C. I. %	Modification	Rank	Result Type
1024.5535	1024.4897	-0.0638	-62	167	175	HALVTDVNR					Mascot
1154.5913	1154.5983	0.007	6	59	69	DGHSVNASVIR					Mascot
1696.9017	1696.8085	-0.0932	-55	225	239	SLYDGKFNLTQALAR					Mascot
1782.8116	1782.881	0.0694	39	113	128	DVAQMASWIDTFGADR					Mascot
1798.8065	1798.8773	0.0708	39	113	128	DVAQMASWIDTFGADR			Oxidation (M)[5]		Mascot
1831.0259	1830.8818	-0.1441	-79	2	18	TFTLLPAIDVLGGRCVR					Mascot
1979.9491	1979.9418	-0.0073	-4	231	247	FNLTQALARLQEEESAC			Carbamidomethyl (C)[17]		Mascot
2019.0879	2018.9678	-0.1201	-59	1	18	MTFTLLPAIDVLGGRCVR			Carbamidomethyl (C)[16]		Mascot
2019.0879	2018.9678	-0.1201	-59	1	18	MTFTLLPAIDVLGGRCVR			Carbamidomethyl (C)[16]		Mascot

8	Chromosome partition protein Smc OS=Alicyclobacillus acidoterrestris (strain ATCC 49025 / DSM 3922 / CIP 106132 / NCIMB 13137 / GD3B) GN=smc PE=3 SV=1	tr|T0C5V6|T0C5V6_ALIAG	133747	5.12	16	27	0	

Peptide Information

Calc. Mass	Obsrv. Mass	± da	± ppm	Start Seq.	End Seq.	Sequence	Ion Score	C. I. %	Modification	Rank	Result Type
1172.6633	1172.594	-0.0693	-59	181	190	LEETKANLVR					Mascot

1172.6633	1172.594	-0.0693	-59	181	190	LEETKANLVR						Mascot
1419.8392	1419.7349	-0.1043	-73	1098	1110	ALTAMALLFAILR			Oxidation (M)[5]			Mascot
1742.7915	1742.9266	0.1351	78	107	120	SGESEYFINRQPCR			Carbamidomethyl (C)[13]			Mascot
1744.9052	1744.9165	0.0113	6	121	135	LKDIHELFMDTGLGR						Mascot
1744.9052	1744.9165	0.0113	6	121	135	LKDIHELFMDTGLGR						Mascot
1760.9	1760.9084	0.0084	5	121	135	LKDIHELFMDTGLGR			Oxidation (M)[9]			Mascot
1766.8749	1766.9031	0.0282	16	1036	1049	FLETFHQIQEEFAK						Mascot
1790.8477	1790.9056	0.0579	32	1021	1035	LDELIEEIDTEMANR						Mascot
1806.8425	1806.8403	-0.0022	-1	1021	1035	LDELIEEIDTEMANR			Oxidation (M)[12]			Mascot
1825.043	1824.9042	-0.1388	-76	225	240	AEIALLVVEIDKLNER						Mascot
1979.9491	1979.9418	-0.0073	-4	799	814	EALAEFEKQQQVMQER			Oxidation (M)[13]			Mascot
2001.991	2001.9592	-0.0318	-16	73	89	KPTNLCEVSLVLDNEDR			Carbamidomethyl (C)[6]			Mascot
2024.0084	2023.9601	-0.0483	-24	598	617	GQAGFVGIASDLVETDAA FR						Mascot
2057.041	2056.9246	-0.1164	-57	256	273	DLAQVSVEAAEAKWQQQ R						Mascot
2057.9268	2057.9177	-0.0091	-4	1154	1171	RGTMEEADALYGVTMPE R			Oxidation (M)[4,15]			Mascot
2075.0801	2074.9282	-0.1519	-73	779	798	SLAETDALLATIAQAMTA QR						Mascot
2520.2888	2520.0879	-0.2009	-80	647	670	VRIVTYQGDVVAPGGVM SGGHHQR						Mascot
2520.2888	2520.0879	-0.2009	-80	647	670	VRIVTYQGDVVAPGGVM SGGHHQR						Mascot
2552.2451	2552.0903	-0.1548	-61	123	145	DIHELFMDTGLGRDAYSII GQGK			Oxidation (M)[7]			Mascot
3337.604	3337.8093	0.2053	62	61	89	MEDVIFAGSETRKPTNLC EVSLVLDNEDR			Carbamidomethyl (C)[18]			Mascot

9	Nucleotide-binding protein N007_01185 OS=Alicyclobacillus acidoterrestris (strain ATCC 49025 / DSM 3922 / CIP 106132 / NCIMB 13137 / GD3B) GN=N007_01185 PE=3 SV=1	tr|T0CZK6|T0CZK 6_ALIAG	32120.4	5.83	7	25	0

Peptide Information

Calc. Mass	Obsrv. Mass	± da	± ppm	Start Seq.	End Seq.	Sequence	Ion Score	C. I. %	Modification	Rank	Result Type
1744.948	1744.9165	-0.0315	-18	66	81	GGALFEPLLTTVEELR					Mascot
1744.948	1744.9165	-0.0315	-18	66	81	GGALFEPLLTTVEELR					Mascot
1748.9575	1748.916	-0.0415	-24	2	18	TVRLETVILTGMSGAGK			Oxidation (M)[12]		Mascot
1769.8414	1769.9102	0.0688	39	228	241	AEEMLDFLLPEYQR			Oxidation (M)[4]		Mascot
1820.931	1820.8567	-0.0743	-41	135	152	AAADIVIDTSDMATSVLK					Mascot
2024.0005	2023.9601	-0.0404	-20	85	102	DMDTTLVFLDANEATLVR					Mascot
2039.9955	2039.9702	-0.0253	-12	85	102	DMDTTLVFLDANEATLVR			Oxidation (M)[2]		Mascot
2180.1016	2179.9968	-0.1048	-48	85	103	DMDTTLVFLDANEATLVR R					Mascot

2391.1794	2391.0056	-0.1738	-73	44	65	LLDMAEHASGSLSKVAFA CDLR			Carbamidomethyl (C)[19]		Mascot

10	2-methylisocitrate lyase OS=Alicyclobacillus acidoterrestris (strain ATCC 49025 / DSM 3922 / CIP 106132 / NCIMB 13137 / GD3B) GN=prpB PE=3 SV=1	tr|T0D3H9|T0D3H9_ALIAG	33350.3	5.34	7	25	0

Peptide Information

Calc. Mass	Obsrv. Mass	± da	± ppm	Start Seq.	End Seq.	Sequence	Ion Score	C. I. %	Modification	Rank	Result Type
1742.9833	1742.9266	-0.0567	-33	137	151	LISADEMVQKIQVIR					Mascot
1758.9783	1758.8915	-0.0868	-49	137	151	LISADEMVQKIQVIR			Oxidation (M)[7]		Mascot
1773.9495	1773.8916	-0.0579	-33	62	77	GIPDLGLVYSEEVARR					Mascot
1986.0114	1985.9766	-0.0348	-18	1	17	MTWLVEPDIQQNTLAAR					Mascot
2002.0062	2001.9592	-0.047	-23	1	17	MTWLVEPDIQQNTLAAR			Oxidation (M)[1]		Mascot
2075.0955	2074.9282	-0.1673	-81	24	43	QPEVLQIPGAHDGMSALL AK					Mascot
2089.978	2089.866	-0.112	-54	109	127	EMVEAGVAAVQIEDQEM PK			Oxidation (M)[2]		Mascot
2374.1628	2374.0073	-0.1555	-65	106	127	TAKEMVEAGVAAVQIED QEMPK					Mascot
2406.1528	2405.9973	-0.1555	-65	106	127	TAKEMVEAGVAAVQIED QEMPK			Oxidation (M)[5,20]		Mascot
2520.2517	2520.0879	-0.1638	-65	275	295	AELYETIHYHAYEALDNRI AK					Mascot
2520.2517	2520.0879	-0.1638	-65	275	295	AELYETIHYHAYEALDNRI AK					Mascot

Gel Idx/Pos	241/J17	Instr./Gel Origin	BA2151/full sequence test 20150515	Process Status	Analysis Succeeded
Plate [#] Name	[3] full sequence test 20150515	Instrument Sample Name		Spectra	11

Rank	Protein Name	Species	Accession No.	Protein MW	Protein PI	Pep. Count	Protein Score	Protein Score C. I. %	Total Ion Score	Total Ion C. I. %
1	Uncharacterized protein OS=Alicyclobacillus acidoterrestris (strain ATCC 49025 / DSM 3922 / CIP 106132 / NCIMB 13137 / GD3B) GN=N007_00440 PE=4 SV=1		tr|T0C5G8|T0C5G8_ALIAG	63753	4.64	9	142	100	121	100

Peptide Information

Calc. Mass	Obsrv. Mass	± da	± ppm	Start Seq.	End Seq.	Sequence	Ion Score	C. I. %	Modification	Rank	Result Type
852.4825	852.4664	-0.0161	-19	313	319	YKSDVLK					Mascot
949.4526	949.4396	-0.013	-14	249	255	NPNYWQK					Mascot
1140.6049	1140.6174	0.0125	11	443	451	LDIHEVTWK					Mascot
1628.7803	1628.7806	0.0003	0	350	363	QAIEYATDDSQFIK					Mascot
1744.9269	1744.9272	0.0003	0	443	456	LDIHEVTWKDFLTK					Mascot
1744.9269	1744.9272	0.0003	0	443	456	LDIHEVTWKDFLTK	40	99.959			Mascot
1808.9211	1808.9204	-0.0007	0	427	442	KDDQAFQSMLSQIGIK					Mascot
1824.916	1824.9103	-0.0057	-3	427	442	KDDQAFQSMLSQIGIK			Oxidation (M)[9]		Mascot
2344.1602	2344.1626	0.0024	1	364	385	INNGAVEGLNQPLPNTMDGYVK					Mascot
2360.155	2360.1565	0.0015	1	364	385	INNGAVEGLNQPLPNTMDGYVK			Oxidation (M)[17]		Mascot
2848.3782	2848.3857	0.0075	3	223	248	LDTSEAMGTGPFEVQTNNQNEVVLVR					Mascot
2848.3782	2848.3857	0.0075	3	223	248	LDTSEAMGTGPFEVQTNNQNEVVLVR	81	100			Mascot
3159.6506	3159.5964	-0.0542	-17	320	347	QPQNSIYYIGLNMKPTLDGKPNPLSNLK			Oxidation (M)[13]		Mascot

Rank	Protein Name	Species	Accession No.	Protein MW	Protein PI	Pep. Count	Protein Score	Protein Score C. I. %	Total Ion Score	Total Ion C. I. %
2	Uncharacterized protein OS=Alicyclobacillus acidoterrestris (strain ATCC 49025 / DSM 3922 / CIP 106132 / NCIMB 13137 / GD3B) GN=N007_10130 PE=4 SV=1		tr|T0D4R7|T0D4R7_ALIAG	29139.8	5.68	7	29	0		

Peptide Information

Calc. Mass	Obsrv. Mass	± da	± ppm	Start Seq.	End Seq.	Sequence	Ion Score	C. I. %	Modification	Rank	Result Type
999.4274	999.4279	0.0005	1	51	58	NFMSPMEK			Oxidation (M)[3]		Mascot
1015.4223	1015.3972	-0.0251	-25	51	58	NFMSPMEK			Oxidation (M)[3,6]		Mascot
1142.5763	1142.5798	0.0035	3	86	95	TYVEMVTGVK			Oxidation (M)[5]		Mascot
1628.8862	1628.7806	-0.1056	-65	228	242	SVIVMMLNQKPSPGK					Mascot

Calc. Mass	Obsrv. Mass	± da	± ppm	Start Seq.	End Seq.	Sequence	Ion Score	C. I. %	Modification	Rank	Result Type
2003.0413	2002.9805	-0.0608	-30	228	245	SVIVMMLNQKPSPGKGER			Oxidation (M)[5,6]		Mascot
2384.1855	2384.0994	-0.0861	-36	2	23	NSPFGARSGQHPNQQIANYIGK					Mascot
2848.2837	2848.3857	0.102	36	51	73	NFMSPMEKVLMENDQEETVYELR			Oxidation (M)[3]		Mascot
2848.2837	2848.3857	0.102	36	51	73	NFMSPMEKVLMENDQEETVYELR			Oxidation (M)[3]		Mascot
3127.6643	3127.6453	-0.019	-6	143	168	ISQEIQKPPEEIYSCMLNPRTLLIVR			Carbamidomethyl (C)[15]		Mascot

3	Uncharacterized protein OS=Alicyclobacillus acidoterrestris (strain ATCC 49025 / DSM 3922 / CIP 106132 / NCIMB 13137 / GD3B) GN=N007_00355 PE=4 SV=1	tr|T0CL47|T0CL47_ALIAG	24603.2	4.76	6	24	0

Peptide Information

Calc. Mass	Obsrv. Mass	± da	± ppm	Start Seq.	End Seq.	Sequence	Ion Score	C. I. %	Modification	Rank	Result Type
950.4797	950.4587	-0.021	-22	17	24	DMIATIMR					Mascot
1053.5364	1053.4962	-0.0402	-38	61	69	FEEIAAFAR					Mascot
1367.6512	1367.6737	0.0225	16	5	16	VICDFDGTIAEK			Carbamidomethyl (C)[3]		Mascot
2166.1191	2166.1775	0.0584	27	154	173	QLADGQTRFIVVGDGVTDFK					Mascot
2328.9902	2329.1816	0.1914	82	132	153	WGVLCDDACTGDCGLCKPSVMR					Mascot
2344.9851	2345.1416	0.1565	67	132	153	WGVLCDDACTGDCGLCKPSVMR			Oxidation (M)[21]		Mascot
2402.0066	2402.0935	0.0869	36	132	153	WGVLCDDACTGDCGLCKPSVMR			Carbamidomethyl (C)[5], Oxidation (M)[21]		Mascot
2800.2344	2800.3882	0.1538	55	130	153	VKWGVLCDDACTGDCGLCKPSVMR			Carbamidomethyl (C)[7,11,15,18], Oxidation (M)[23]		Mascot

4	Uncharacterized protein OS=Alicyclobacillus acidoterrestris (strain ATCC 49025 / DSM 3922 / CIP 106132 / NCIMB 13137 / GD3B) GN=N007_05585 PE=4 SV=1	tr|T0DDI8|T0DDI8_ALIAG	51865.9	9.71	10	24	0

Peptide Information

Calc. Mass	Obsrv. Mass	± da	± ppm	Start Seq.	End Seq.	Sequence	Ion Score	C. I. %	Modification	Rank	Result Type
819.3843	819.3959	0.0116	14	165	172	TGGDDNLK					Mascot
981.5226	981.4521	-0.0705	-72	224	231	WMLAVAYK					Mascot
981.5226	981.4521	-0.0705	-72	224	231	WMLAVAYK					Mascot
1049.5739	1049.4856	-0.0883	-84	314	322	ISDKVANFR					Mascot
1168.6433	1168.6171	-0.0262	-22	405	414	NRPKVPDQSK					Mascot
1666.8833	1666.7424	-0.1409	-85	83	97	YSAMGNVTLINQLVK			Oxidation (M)[4]		Mascot
1846.9221	1846.8926	-0.0295	-16	35	49	IKNSAIQYFYEDDIK					Mascot
1848.764	1848.885	0.121	65	417	432	CLSCGYETNADFNAAR			Carbamidomethyl (C)[1,4]		Mascot

Calc. Mass	Obsrv. Mass	± da	± ppm	Start Seq.	End Seq.	Sequence	Ion Score	C. I. %	Modification	Rank	Result Type
2166.0503	2166.1775	0.1272	59	64	82	GEFYGSSVSVYNYVRPDVK					Mascot
2520.2261	2520.0925	-0.1336	-53	242	263	NRVLGIDMGVVYPAYMAVNYDK			Oxidation (M)[8,16]		Mascot
3127.5852	3127.6453	0.0601	19	1	26	MIVKTMPYEIIKPLSCDWAVFGEMLR			Carbamidomethyl (C)[16]		Mascot
3159.575	3159.5964	0.0214	7	1	26	MIVKTMPYEIIKPLSCDWAVFGEMLR			Carbamidomethyl (C)[16], Oxidation (M)[1,6]		Mascot

5	Uncharacterized protein OS=Alicyclobacillus acidoterrestris (strain ATCC 49025 / DSM 3922 / CIP 106132 / NCIMB 13137 / GD3B) GN=N007_11845 PE=4 SV=1	tr|T0BJ34|T0BJ34_ALIAG	27231	6.08	6	22	0	

Peptide Information

Calc. Mass	Obsrv. Mass	± da	± ppm	Start Seq.	End Seq.	Sequence	Ion Score	C. I. %	Modification	Rank	Result Type
1156.5303	1156.571	0.0407	35	158	169	GAAMEAAYSASK					Mascot
1172.5253	1172.601	0.0757	65	158	169	GAAMEAAYSASK			Oxidation (M)[4]		Mascot
1172.5253	1172.601	0.0757	65	158	169	GAAMEAAYSASK			Oxidation (M)[4]		Mascot
1611.8411	1611.755	-0.0861	-53	120	133	VLQTNLTSTFLCSK			Carbamidomethyl (C)[12]		Mascot
1846.8752	1846.8926	0.0174	9	1	17	MEAESREWGQSAPLAGK					Mascot
2150.0876	2150.2017	0.1141	53	235	255	FLASPDSSFITGQVISPNGGR					Mascot
2344.1792	2344.1626	-0.0166	-7	39	60	AGAHVAVHYHEAQVEALDTVAR					Mascot
2360.2246	2360.1565	-0.0681	-29	235	257	FLASPDSSFITGQVISPNGGRLP					Mascot

6	Uncharacterized protein OS=Alicyclobacillus acidoterrestris (strain ATCC 49025 / DSM 3922 / CIP 106132 / NCIMB 13137 / GD3B) GN=N007_17610 PE=4 SV=1	tr|T0BAM2|T0BAM2_ALIAG	21274.7	5.88	6	22	0	

Peptide Information

Calc. Mass	Obsrv. Mass	± da	± ppm	Start Seq.	End Seq.	Sequence	Ion Score	C. I. %	Modification	Rank	Result Type
1053.4735	1053.4962	0.0227	22	177	185	EWITSESTT					Mascot
1156.6433	1156.571	-0.0723	-63	129	138	VEIRAAVENR					Mascot
1363.6641	1363.705	0.0409	30	92	103	SASIGYWLSHDK					Mascot
1649.8646	1649.7467	-0.1179	-71	115	128	AVTDTLFKEYGLHR					Mascot
1666.8291	1666.7424	-0.0867	-52	1	13	MFCLRVDDEIQLK			Carbamidomethyl (C)[3]		Mascot
1744.9164	1744.9272	0.0108	6	142	156	AIPERIGFVQEGICR			Carbamidomethyl (C)[14]		Mascot
1744.9164	1744.9272	0.0108	6	142	156	AIPERIGFVQEGICR			Carbamidomethyl (C)[14]		Mascot

7	Uncharacterized protein OS=Alicyclobacillus acidoterrestris (strain ATCC 49025 / DSM 3922 / CIP 106132 / NCIMB 13137 / GD3B) GN=N007_08250 PE=4 SV=1	tr|T0BZ10|T0BZ10_ALIAG	12241.2	5.3	4	22	0	

Peptide Information

Calc. Mass	Obsrv. Mass	± da	± ppm	Start Seq.	End Seq.	Sequence	Ion Score	C. I. %	Modification	Rank	Result Type
1049.5547	1049.4856	-0.0691	-66	46	54	DETAIMILK			Oxidation (M)[6]		Mascot
1935.9741	1935.901	-0.0731	-38	38	54	LPFCGPCKDETAIMILK			Carbamidomethyl (C)[4]		Mascot
2831.4575	2831.3311	-0.1264	-45	80	103	RIVEVEEATPENNFLFDD VLVNLR					Mascot
2889.3538	2889.3396	-0.0142	-5	55	79	ADSLEEAEELLAGDLFTR ANYYQDR					Mascot

8	Elongation factor Ts OS=Alicyclobacillus acidoterrestris (strain ATCC 49025 / DSM 3922 / CIP 106132 / NCIMB 13137 / GD3B) GN=tsf PE=3 SV=1	tr|T0D959|T0D959 _ALIAG	24135.5	5.18	7	22	0

Peptide Information

Calc. Mass	Obsrv. Mass	± da	± ppm	Start Seq.	End Seq.	Sequence	Ion Score	C. I. %	Modification	Rank	Result Type
949.5023	949.4396	-0.0627	-66	2	10	AEITAAMVK			Oxidation (M)[7]		Mascot
1367.7318	1367.6737	-0.0581	-42	191	202	FARYVVGEGIEK					Mascot
1628.8214	1628.7806	-0.0408	-25	97	111	DVAMHIAAASPQYVR					Mascot
1678.8759	1678.8547	-0.0212	-13	129	143	AQTLNEGKPEHIVDK					Mascot
1758.9136	1758.894	-0.0196	-11	152	165	FFKDICLLEQEFVK					Mascot
1800.9174	1800.9209	0.0035	2	97	112	DVAMHIAAASPQYVRR			Oxidation (M)[4]		Mascot
1848.9048	1848.885	-0.0198	-11	155	169	DICLLEQEFVKDPDK			Carbamidomethyl (C)[3]		Mascot

9	Uncharacterized protein OS=Alicyclobacillus acidoterrestris (strain ATCC 49025 / DSM 3922 / CIP 106132 / NCIMB 13137 / GD3B) GN=N007_08205 PE=4 SV=1	tr|T0BY66|T0BY66 _ALIAG	16909.8	9.83	5	21	0

Peptide Information

Calc. Mass	Obsrv. Mass	± da	± ppm	Start Seq.	End Seq.	Sequence	Ion Score	C. I. %	Modification	Rank	Result Type
977.4291	977.4493	0.0202	21	103	110	SCMHALSR			Carbamidomethyl (C)[2], Oxidation (M)[3]		Mascot
1649.8237	1649.7467	-0.077	-47	75	88	TQMTPVQQLMEISK			Oxidation (M)[3]		Mascot
1789.9299	1789.8553	-0.0746	-42	75	89	TQMTPVQQLMEISKR					Mascot
2344.2371	2344.1626	-0.0745	-32	41	61	FISIAASNLDEFFMVRVA GLK			Oxidation (M)[14]		Mascot
2360.1206	2360.1565	0.0359	15	127	145	DFLARYFSEHIPYLNSNH H					Mascot

10	Uncharacterized protein OS=Alicyclobacillus acidoterrestris (strain ATCC 49025 / DSM 3922 / CIP 106132 / NCIMB 13137 / GD3B) GN=N007_18355 PE=4 SV=1	tr|T0CJU0|T0CJU0 _ALIAG	56046.8	5.66	8	21	0

Peptide Information

Calc. Mass	Obsrv. Mass	± da	± ppm	Start Seq.	End Seq.	Sequence	Ion Score	C. I. %	Modification	Rank	Result Type
1349.6011	1349.6591	0.058	43	453	465	ESGTGLGMMVCHK					Mascot
1766.8207	1766.8953	0.0746	42	303	316	WSLNVFTMDHPEYK					Mascot
1831.0245	1830.9216	-0.1029	-56	352	368	LEEILGVVITLMSSQAK					Mascot
1847.0194	1846.8926	-0.1268	-69	352	368	LEEILGVVITLMSSQAK			Oxidation (M)[12]		Mascot
2003.1206	2002.9805	-0.1401	-70	352	369	LEEILGVVITLMSSQAKR			Oxidation (M)[12]		Mascot
2329.0847	2329.1816	0.0969	42	453	473	ESGTGLGMMVCHKIIQDH AGR			Carbamidomethyl (C)[11], Oxidation (M)[8,9]		Mascot
2402.1189	2402.0935	-0.0254	-11	444	465	LGEPFFTTKESGTGLGM MVCHK			Oxidation (M)[17,18]		Mascot
2848.4761	2848.3857	-0.0904	-32	220	244	QGKPIQLETDILMADDSV RTFELTK					Mascot
2848.4761	2848.3857	-0.0904	-32	220	244	QGKPIQLETDILMADDSV RTFELTK					Mascot
3124.5198	3124.7239	0.2041	65	303	328	WSLNVFTMDHPEYKDQF QAILGELSR					Mascot

Gel Idx/Pos	242/J18	Instr./Gel Origin	BA2151/full sequence test 20150515	Process Status	Analysis Succeeded
Plate [#] Name	[3] full sequence test 20150515	Instrument Sample Name		Spectra	11

Rank	Protein Name	Species	Accession No.	Protein MW	Protein PI	Pep. Count	Protein Score	Protein Score C. I. %	Total Ion Score	Total Ion C. I. %
1	Superoxide dismutase OS=Alicyclobacillus acidoterrestris (strain ATCC 49025 / DSM 3922 / CIP 106132 / NCIMB 13137 / GD3B) GN=N007_10430 PE=3 SV=1		tr|T0D4I3|T0D4I3_ALIAG	22299.1	5.66	11	280	100	214	100

Peptide Information

Calc. Mass	Obsrv. Mass	± da	± ppm	Start Seq.	End Seq.	Sequence	Ion Score	C. I. %	Modification	Rank	Result Type
1064.512	1064.5223	0.0103	10	117	126	EQFNAAATGR					Mascot
1064.512	1064.5223	0.0103	10	117	126	EQFNAAATGR	22	96.714			Mascot
1129.5848	1129.5935	0.0087	8	42	52	ALEGQADLANK					Mascot
1283.6492	1283.6575	0.0083	6	31	41	HHGTYVTNLNK					Mascot
1339.6754	1339.684	0.0086	6	115	126	FKEQFNAAATGR					Mascot
1499.7377	1499.7396	0.0019	1	101	114	LAEAINSTFGSFDK					Mascot
1538.7247	1538.7131	-0.0116	-8	74	87	NNGGGHANHSLFWK					Mascot
1774.9011	1774.9062	0.0051	3	101	116	LAEAINSTFGSFDKFK					Mascot
1774.9011	1774.9062	0.0051	3	101	116	LAEAINSTFGSFDKFK	18	90.78			Mascot
1814.9316	1814.9298	-0.0018	-1	141	157	LAIISTANQDNPLMEGK					Mascot
1830.9266	1830.928	0.0014	1	141	157	LAIISTANQDNPLMEGK			Oxidation (M)[14]		Mascot
1883.9709	1883.9741	0.0032	2	53	69	SVEDLISDLNAVPENIR					Mascot
1883.9709	1883.9741	0.0032	2	53	69	SVEDLISDLNAVPENIR	107	100			Mascot
1931.0426	1931.0461	0.0035	2	158	173	KPVLGLDVWEHAYYLK					Mascot
1931.0426	1931.0461	0.0035	2	158	173	KPVLGLDVWEHAYYLK	66	100			Mascot
3322.6313	3322.6729	0.0416	13	2	30	AHELPALPYAFDALEPHIDALTMEIHHDR					Mascot

Rank	Protein Name	Species	Accession No.	Protein MW	Protein PI	Pep. Count	Protein Score	Protein Score C. I. %	Total Ion Score	Total Ion C. I. %
2	Integrase (Fragment) OS=Alicyclobacillus acidoterrestris (strain ATCC 49025 / DSM 3922 / CIP 106132 / NCIMB 13137 / GD3B) GN=N007_14940 PE=4 SV=1		tr|T0BP12|T0BP12_ALIAG	46576	9.27	10	34	0		

Peptide Information

Calc. Mass	Obsrv. Mass	± da	± ppm	Start Seq.	End Seq.	Sequence	Ion Score	C. I. %	Modification	Rank	Result Type
1362.764	1362.6573	-0.1067	-78	294	304	HRYLLAYSALR					Mascot
1510.8199	1510.7375	-0.0824	-55	96	108	EFMHPLRPVVSAK					Mascot

Calc. Mass	Obsrv. Mass	± da	± ppm	Start Seq.	End Seq.	Sequence	Ion Score	C. I. %	Modification	Rank	Result Type
1526.8148	1526.7291	-0.0857	-56	96	108	EFMHPLRPVVSAK			Oxidation (M)[3]		Mascot
1591.809	1591.668	-0.141	-89	214	226	YYGFVPKACKPYR					Mascot
1592.76	1592.6829	-0.0771	-48	139	151	TLWCFAMVLSYSR			Oxidation (M)[7]		Mascot
1654.8218	1654.7374	-0.0844	-51	12	25	MYQEGVSISELSRR					Mascot
1670.8167	1670.7419	-0.0748	-45	12	25	MYQEGVSISELSRR			Oxidation (M)[1]		Mascot
1870.9254	1870.8822	-0.0432	-23	36	52	VVQEQEDGNNPLSGTKR					Mascot
1938.0742	1937.9807	-0.0935	-48	96	112	EFMHPLRPVVSAKATVR					Mascot
1999.0496	1998.9944	-0.0552	-28	340	357	LQIEYGGQVIAEHSLVDK					Mascot
2801.4768	2801.3962	-0.0806	-29	84	108	EQGYTGGITVLREFMHPL RPVVSAK			Oxidation (M)[15]		Mascot
2837.3489	2837.4319	0.083	29	113	138	FETGPGEQAQIDLGAFPY VDAEGNRR					Mascot

3 Methionine--tRNA ligase OS=Alicyclobacillus acidoterrestris (strain ATCC 49025 / DSM 3922 / CIP 106132 / NCIMB 13137 / GD3B) GN=metG PE=3 SV=1 | tr|T0DPA1|T0DPA1_ALIAG | 74286 | 5.64 | 12 | 27 | 0

Peptide Information

Calc. Mass	Obsrv. Mass	± da	± ppm	Start Seq.	End Seq.	Sequence	Ion Score	C. I. %	Modification	Rank	Result Type
1046.5114	1046.5117	0.0003	0	527	536	ALQNTEDAGK					Mascot
1338.7377	1338.6869	-0.0508	-38	307	318	GNVIDPLQLVDR					Mascot
1338.7377	1338.6869	-0.0508	-38	307	318	GNVIDPLQLVDR					Mascot
1339.5988	1339.684	0.0852	64	157	166	EESYFFRMSK			Oxidation (M)[8]		Mascot
1494.7046	1494.734	0.0294	20	423	434	ANKYIDECQPWK					Mascot
1536.7047	1536.7098	0.0051	3	145	156	CPECGREVQFVR			Carbamidomethyl (C)[1,4]		Mascot
1551.7261	1551.7106	-0.0155	-10	423	434	ANKYIDECQPWK			Carbamidomethyl (C)[8]		Mascot
1559.7676	1559.7064	-0.0612	-39	426	437	YIDECQPWKLHK					Mascot
1565.8323	1565.7003	-0.132	-84	579	591	LLQFQVDLGFETR					Mascot
1616.7889	1616.6942	-0.0947	-59	426	437	YIDECQPWKLHK			Carbamidomethyl (C)[5]		Mascot
1774.8871	1774.9062	0.0191	11	349	363	LNYDLANDFGNLIHR					Mascot
1774.8871	1774.9062	0.0191	11	349	363	LNYDLANDFGNLIHR					Mascot
1870.9294	1870.8822	-0.0472	-25	86	100	LNISYDDFIRTTEQR					Mascot
2017.0861	2016.9929	-0.0932	-46	457	475	LATIMVQPFMTDAPVAIA K					Mascot
2209.0991	2209.0842	-0.0149	-7	189	207	TEMLKNFIEPGLQDLCVS R			Oxidation (M)[3]		Mascot
2858.229	2858.3782	0.1492	52	116	138	GDIYLSEYEGWYCTPDE SFWLER					Mascot

4 Uncharacterized protein OS=Alicyclobacillus acidoterrestris (strain ATCC 49025 / DSM 3922 / CIP 106132 / NCIMB 13137 / GD3B) GN=N007_10130 PE=4 SV=1 | tr|T0D4R7|T0D4R7_ALIAG | 29139.8 | 5.68 | 7 | 27 | 0

Peptide Information

Calc. Mass	Obsrv. Mass	± da	± ppm	Start Seq.	End Seq.	Sequence	Ion Score	C. I. %	Modification	Rank	Result Type
1561.6628	1561.7086	0.0458	29	214	225	VMDSFVDWDFER			Oxidation (M)[2]		Mascot
1654.8296	1654.7374	-0.0922	-56	9	23	SGQHPNQQIANYIGK					Mascot
1788.7898	1788.9186	0.1288	72	214	227	VMDSFVDWDFERDK					Mascot
1883.8691	1883.9741	0.105	56	59	73	VLMENDQEETVYELR			Oxidation (M)[3]		Mascot
1883.8691	1883.9741	0.105	56	59	73	VLMENDQEETVYELR			Oxidation (M)[3]		Mascot
1969.0464	1969.0054	-0.041	-21	79	95	SLFPEIRTYVEMVTGVK					Mascot
1985.0413	1985.0127	-0.0286	-14	79	95	SLFPEIRTYVEMVTGVK			Oxidation (M)[12]		Mascot
2003.0413	2003.0332	-0.0081	-4	228	245	SVIVMMLNQKPSPGKGE R			Oxidation (M)[5,6]		Mascot
2832.2888	2832.4102	0.1214	43	51	73	NFMSPMEKVLMENDQEE TVYELR					Mascot

5	Uncharacterized protein OS=Alicyclobacillus acidoterrestris (strain ATCC 49025 / DSM 3922 / CIP 106132 / NCIMB 13137 / GD3B) GN=N007_13360 PE=4 SV=1	tr|T0BRZ1|T0BRZ1_ALIAG	15271.5	4.85	5	26	0

Peptide Information

Calc. Mass	Obsrv. Mass	± da	± ppm	Start Seq.	End Seq.	Sequence	Ion Score	C. I. %	Modification	Rank	Result Type
1360.6532	1360.6697	0.0165	12	4	14	LTPYFYSENAR					Mascot
1559.7853	1559.7064	-0.0789	-51	2	14	AKLTPYFYSENAR					Mascot
1830.8029	1830.928	0.1251	68	74	89	ASFDLTLEFDSDDEAR					Mascot
1935.0256	1935.0405	0.0149	8	90	107	EAFTKLSAGGQVIMPLEK			Oxidation (M)[14]		Mascot
3351.6279	3351.6819	0.054	16	4	32	LTPYFYSENAREQASFYI AALGGEIQQER					Mascot

6	Ribose-phosphate pyrophosphokinase OS=Alicyclobacillus acidoterrestris (strain ATCC 49025 / DSM 3922 / CIP 106132 / NCIMB 13137 / GD3B) GN=prs PE=3 SV=1	tr|T0DUM5|T0DUM5_ALIAG	34109.1	6.1	7	26	0

Peptide Information

Calc. Mass	Obsrv. Mass	± da	± ppm	Start Seq.	End Seq.	Sequence	Ion Score	C. I. %	Modification	Rank	Result Type
1549.8585	1549.7046	-0.1539	-99	269	282	EVVVTNTIALPEHK					Mascot
1814.8954	1814.9298	0.0344	19	162	178	QLEDPVVVSPDMGGVTR			Oxidation (M)[12]		Mascot
1884.0127	1883.9741	-0.0386	-20	83	99	ASARAINVVIPYYGYAR					Mascot
1884.0127	1883.9741	-0.0386	-20	83	99	ASARAINVVIPYYGYAR					Mascot
1897.9919	1897.9917	-0.0002	0	87	102	AINVVIPYYGYARQDR					Mascot
1897.9919	1897.9917	-0.0002	0	87	102	AINVVIPYYGYARQDR					Mascot

1930.9263	1931.0461	0.1198	62	244	261	GVYACCIHPVLSGDGVQR			Carbamidomethyl (C)[5]		Mascot
1930.9263	1931.0461	0.1198	62	244	261	GVYACCIHPVLSGDGVQR			Carbamidomethyl (C)[5]		Mascot
3029.5361	3029.5969	0.0608	20	9	37	IVTGNSNPALAQEIADYIGVALAECQVNR					Mascot
3351.7803	3351.6819	-0.0984	-29	53	82	GSNVFIIQPTSAPVNEHLMELLIMVDALKR			Oxidation (M)[19]		Mascot

7	Uncharacterized protein OS=Alicyclobacillus acidoterrestris (strain ATCC 49025 / DSM 3922 / CIP 106132 / NCIMB 13137 / GD3B) GN=N007_11000 PE=4 SV=1	tr|T0BUK2|T0BUK2_ALIAG	6169.8	4.45	4	25	0

Peptide Information

Calc. Mass	Obsrv. Mass	± da	± ppm	Start Seq.	End Seq.	Sequence	Ion Score	C. I. %	Modification	Rank	Result Type
1360.6743	1360.6697	-0.0046	-3	12	23	EVEWKDGAELGK					Mascot
1410.6075	1410.7057	0.0982	70	1	11	MFVIHCNSCSR			Carbamidomethyl (C)[6,9]		Mascot
1836.8521	1836.9187	0.0666	36	2	16	FVIHCNSCSREVEWK					Mascot
2820.2532	2820.4119	0.1587	56	24	49	LQIEMCGSTVICACGHGVGDDNGTLR			Carbamidomethyl (C)[6,12,14]		Mascot
2820.2532	2820.4119	0.1587	56	24	49	LQIEMCGSTVICACGHGVGDDNGTLR			Carbamidomethyl (C)[6,12,14]		Mascot

8	Uncharacterized protein OS=Alicyclobacillus acidoterrestris (strain ATCC 49025 / DSM 3922 / CIP 106132 / NCIMB 13137 / GD3B) GN=N007_04235 PE=4 SV=1	tr|T0C9D1|T0C9D1_ALIAG	10637.5	6.28	4	24	0

Peptide Information

Calc. Mass	Obsrv. Mass	± da	± ppm	Start Seq.	End Seq.	Sequence	Ion Score	C. I. %	Modification	Rank	Result Type
1360.6678	1360.6697	0.0019	1	1	11	MPQTDFHLLSR			Oxidation (M)[1]		Mascot
1870.9592	1870.8822	-0.077	-41	1	15	MPQTDFHLLSREQLR					Mascot
2209.0476	2209.0842	0.0366	17	45	63	QMNLLEGQMTSEELAEVVR			Oxidation (M)[2,9]		Mascot
2845.4434	2845.3838	-0.0596	-21	45	69	QMNLLEGQMTSEELAEVVRLAEQVK					Mascot

9	Uncharacterized protein OS=Alicyclobacillus acidoterrestris (strain ATCC 49025 / DSM 3922 / CIP 106132 / NCIMB 13137 / GD3B) GN=N007_02350 PE=4 SV=1	tr|T0C9C3|T0C9C3_ALIAG	88290.9	5.49	12	24	0

Peptide Information

Calc. Mass	Obsrv. Mass	± da	± ppm	Start Seq.	End Seq.	Sequence	Ion Score	C. I. %	Modification	Rank	Result Type
1376.6918	1376.6427	-0.0491	-36	614	624	QRHSYLDQTTK					Mascot

Calc. Mass	Obsrv. Mass	± da	± ppm	Start Seq.	End Seq.	Sequence	Ion Score	C. I. %	Modification	Rank	Result Type
1508.7203	1508.7595	0.0392	26	272	283	QFMQPDFPDLVR			Oxidation (M)[3]		Mascot
1516.8119	1516.7279	-0.084	-55	736	749	AKLLGDPSQAFDVR					Mascot
1539.7328	1539.7194	-0.0134	-9	724	735	QLMEQLSLMQMR			Oxidation (M)[3,9]		Mascot
1555.7278	1555.6997	-0.0281	-18	724	735	QLMEQLSLMQMR			Oxidation (M)[3,9,11]		Mascot
1774.9303	1774.9062	-0.0241	-14	198	213	IQAKSMQVIGAEALCR			Carbamidomethyl (C)[15]		Mascot
1774.9303	1774.9062	-0.0241	-14	198	213	IQAKSMQVIGAEALCR			Carbamidomethyl (C)[15]		Mascot
1852.9188	1852.9098	-0.009	-5	48	63	QVNENFTYGVGDQILR					Mascot
1896.9814	1896.9263	-0.0551	-29	600	615	LGDTIYAAYLDELRQR					Mascot
1911.9594	1912.0059	0.0465	24	304	320	TAMQDVDNAVHVLTELR					Mascot
1930.979	1931.0461	0.0671	35	288	303	ETKMDPNLLELEITER					Mascot
1930.979	1931.0461	0.0671	35	288	303	ETKMDPNLLELEITER					Mascot
1946.974	1947.0371	0.0631	32	288	303	ETKMDPNLLELEITER			Oxidation (M)[4]		Mascot
2826.4377	2826.4263	-0.0114	-4	158	182	NQVCVHSTTETDLPLTPL IVETMLR			Oxidation (M)[23]		Mascot
2834.4797	2834.4314	-0.0483	-17	326	350	IAIDDFGIGYSSLSYLMRF PVQTIK					Mascot
2845.2847	2845.3838	0.0991	35	568	591	GQEVYGMFTLQSSHPNL YTDDDRR			Oxidation (M)[7]		Mascot

10 Serine recombinase OS=Alicyclobacillus acidoterrestris (strain ATCC 49025 / DSM 3922 / CIP 106132 / NCIMB 13137 / GD3B) GN=N007_10480 PE=4 SV=1 — tr|T0CZ98|T0CZ98_ALIAG — 59684.9 — 9.2 — 8 — 24 — 0 — 5 — 0

Peptide Information

Calc. Mass	Obsrv. Mass	± da	± ppm	Start Seq.	End Seq.	Sequence	Ion Score	C. I. %	Modification	Rank	Result Type
875.4767	875.5129	0.0362	41	322	328	CGEKLIR			Carbamidomethyl (C)[1]		Mascot
1226.6852	1226.6439	-0.0413	-34	267	276	RPEEEVIRAK					Mascot
1339.573	1339.684	0.111	83	104	114	GDMEDQGRIMR			Oxidation (M)[3,10]		Mascot
1774.9116	1774.9062	-0.0054	-3	88	103	GETQAVLTMDIDRLGR					Mascot
1774.9116	1774.9062	-0.0054	-3	88	103	GETQAVLTMDIDRLGR	5	0			Mascot
1906.0255	1905.9508	-0.0747	-39	463	478	TIPHVIHVLDLYPNMK			Oxidation (M)[15]		Mascot
1930.9725	1931.0461	0.0736	38	308	325	QIANPLAGLIECSKCGEK			Carbamidomethyl (C)[12]		Mascot
1930.9725	1931.0461	0.0736	38	308	325	QIANPLAGLIECSKCGEK			Carbamidomethyl (C)[12]		Mascot
2826.5083	2826.4263	-0.082	-29	200	225	LIFDLHVNRGMGGSHIAS YLNTLGIK					Mascot
2842.5032	2842.3889	-0.1143	-40	200	225	LIFDLHVNRGMGGSHIAS YLNTLGIK			Oxidation (M)[11]		Mascot
3322.4199	3322.6729	0.253	76	365	392	EWLCEYTISCDEVTAATN APTEAHHNMR			Carbamidomethyl (C)[4,10], Oxidation (M)[27]		Mascot

Gel Idx/Pos	243/J19	Instr./Gel Origin	BA2151/full sequence test 20150515	Process Status	Analysis Succeeded
Plate [#] Name	[3] full sequence test 20150515	Instrument Sample Name		Spectra	11

Rank	Protein Name	Species	Accession No.	Protein MW	Protein PI	Pep. Count	Protein Score	Protein Score C. I. %	Total Ion Score	Total Ion C. I. %
1	6-phosphogluconate dehydrogenase OS=Alicyclobacillus acidoterrestris (strain ATCC 49025 / DSM 3922 / CIP 106132 / NCIMB 13137 / GD3B) GN=N007_01705 PE=4 SV=1		tr|T0CYB3|T0CYB3_ALIAG	32612.2	5.57	12	384	100	325	100

Peptide Information

Calc. Mass	Obsrv. Mass	± da	± ppm	Start Seq.	End Seq.	Sequence	Ion Score	C. I. %	Modification	Rank	Result Type
875.4985	875.5094	0.0109	12	144	150	TIEPLFR					Mascot
879.3777	879.3948	0.0171	19	164	171	AGSGHYCK			Carbamidomethyl (C)[7]		Mascot
1029.6125	1029.6151	0.0026	3	1	10	MQIGIIGLGK					Mascot
1300.6896	1300.6982	0.0086	7	24	34	HEVVAFDLDKK					Mascot
1402.6962	1402.7052	0.009	6	87	99	GDIIIEGGNSHYK					Mascot
1402.6962	1402.7052	0.009	6	87	99	GDIIIEGGNSHYK	67	100			Mascot
1412.6805	1412.6919	0.0114	8	151	163	DTAVPNGYVYTGR					Mascot
1537.696	1537.7108	0.0148	10	11	23	MGYNLALNMMDHK					Mascot
1553.6909	1553.697	0.0061	4	11	23	MGYNLALNMMDHK			Oxidation (M)[1]		Mascot
1569.6859	1569.6876	0.0017	1	11	23	MGYNLALNMMDHK			Oxidation (M)[1,9]		Mascot
1585.6808	1585.71	0.0292	18	11	23	MGYNLALNMMDHK			Oxidation (M)[1,9,10]		Mascot
1654.7352	1654.7446	0.0094	6	129	143	HGACYMIGGDAEVFK			Carbamidomethyl (C)[4]		Mascot
1654.7352	1654.7446	0.0094	6	129	143	HGACYMIGGDAEVFK	106	100	Carbamidomethyl (C)[4]		Mascot
1670.7302	1670.7395	0.0093	6	129	143	HGACYMIGGDAEVFK			Carbamidomethyl (C)[4], Oxidation (M)[6]		Mascot
1670.7302	1670.7395	0.0093	6	129	143	HGACYMIGGDAEVFK	59	100	Carbamidomethyl (C)[4], Oxidation (M)[6]		Mascot
1688.8701	1688.8762	0.0061	4	39	56	LAGEGATGASSIADLVEK					Mascot
1688.8701	1688.8762	0.0061	4	39	56	LAGEGATGASSIADLVEK					Mascot
1870.8719	1870.875	0.0031	2	111	128	HGIYFFDVGTSGGTEGAR					Mascot
1870.8719	1870.875	0.0031	2	111	128	HGIYFFDVGTSGGTEGAR	152	100			Mascot
1887.9559	1887.9424	-0.0135	-7	87	103	GDIIIEGGNSHYKESIR					Mascot
2254.2039	2254.2178	0.0139	6	39	61	LAGEGATGASSIADLVEKLQAPR					Mascot

Rank	Protein Name	Species	Accession No.	Protein MW	Protein PI	Pep. Count	Protein Score	Protein Score C. I. %	Total Ion Score	Total Ion C. I. %
2	Uncharacterized protein OS=Alicyclobacillus acidoterrestris (strain ATCC 49025 / DSM 3922 / CIP 106132 / NCIMB 13137 / GD3B) GN=N007_13145 PE=4 SV=1		tr|T0BH84|T0BH84_ALIAG	13496.9	5.31	6	32	0		

Peptide Information

Calc. Mass	Obsrv. Mass	± da	± ppm	Start Seq.	End Seq.	Sequence	Ion Score	C. I. %	Modification	Rank	Result Type
1666.7598	1666.7452	-0.0146	-9	1	14	MGQQELMSQIENMK					Mascot
1674.8732	1674.7424	-0.1308	-78	105	119	SSLPTVQGRMLDIEL			Oxidation (M)[10]		Mascot
1714.7445	1714.6853	-0.0592	-35	1	14	MGQQELMSQIENMK			Oxidation (M)[1,7,13]		Mascot
1883.0458	1882.8837	-0.1621	-86	71	86	ILQQNEQISLGISQRR					Mascot
1883.0458	1882.8837	-0.1621	-86	70	85	RILQQNEQISLGISQR					Mascot
1906.9757	1906.8436	-0.1321	-69	20	36	ILAQLEDASVSFESIER					Mascot
2225.0359	2225.1399	0.104	47	1	19	MGQQELMSQIENMKSIG ER			Oxidation (M)[1]		Mascot

3	Uncharacterized protein OS=Alicyclobacillus acidoterrestris (strain ATCC 49025 / DSM 3922 / CIP 106132 / NCIMB 13137 / GD3B) GN=N007_08170 PE=4 SV=1	tr|T0D2D0|T0D2D 0_ALIAG	6562.5	6.23	4	29	0

Peptide Information

Calc. Mass	Obsrv. Mass	± da	± ppm	Start Seq.	End Seq.	Sequence	Ion Score	C. I. %	Modification	Rank	Result Type
879.3876	879.3948	0.0072	8	41	47	ISDEQCK			Carbamidomethyl (C)[6]		Mascot
1665.8	1665.9473	0.1473	88	41	55	ISDEQCKTVDEVTAK					Mascot
2225.123	2225.1399	0.0169	8	29	47	HLLDAIEALDVRISDEQCK			Carbamidomethyl (C)[18]		Mascot
2819.5212	2819.407	-0.1142	-41	1	26	MPLARFALAWVLHHPAV TCAIIGSTK			Oxidation (M)[1]		Mascot
2860.5476	2860.5505	0.0029	1	1	26	MPLARFALAWVLHHPAV TCAIIGSTK			Carbamidomethyl (C)[19]		Mascot

4	Transcription attenuation protein MtrB OS=Alicyclobacillus acidoterrestris (strain ATCC 49025 / DSM 3922 / CIP 106132 / NCIMB 13137 / GD3B) GN=N007_14590 PE=4 SV=1	tr|T0BQ55|T0BQ5 5_ALIAG	8949.6	6.4	4	27	0

Peptide Information

Calc. Mass	Obsrv. Mass	± da	± ppm	Start Seq.	End Seq.	Sequence	Ion Score	C. I. %	Modification	Rank	Result Type
1213.7052	1213.6543	-0.0509	-42	62	72	ALLYTAHGVIR					Mascot
1852.8568	1852.9092	0.0524	28	42	57	NEVMIAQFTEHTSAMK			Oxidation (M)[4]		Mascot
1852.8568	1852.9092	0.0524	28	42	57	NEVMIAQFTEHTSAMK			Oxidation (M)[4]		Mascot
2225.0576	2225.1399	0.0823	37	39	57	LDKNEVMIAQFTEHTSAM K			Oxidation (M)[7,18]		Mascot
2836.4663	2836.3979	-0.0684	-24	2	27	ADSPVLGDYFVIRALENG VQVIGMTR			Oxidation (M)[24]		Mascot

5	Uncharacterized protein OS=Alicyclobacillus acidoterrestris (strain ATCC 49025 / DSM 3922 / CIP 106132 / NCIMB 13137 / GD3B) GN=N007_06365	tr|T0C2T8|T0C2T8 _ALIAG	10293.4	8.85	4	24	0

PE=4 SV=1

Peptide Information

Calc. Mass	Obsrv. Mass	± da	± ppm	Start Seq.	End Seq.	Sequence	Ion Score	C. I. %	Modification	Rank	Result Type
999.6196	999.5724	-0.0472	-47	38	47	AKILGLAEGK					Mascot
1591.7936	1591.7142	-0.0794	-50	2	14	SSRQQSHLQTYTR					Mascot
1869.8947	1869.8901	-0.0046	-2	48	65	FIQGRENVVCMGASGTGK			Oxidation (M)[11]		Mascot
1910.9211	1910.8612	-0.0599	-31	48	65	FIQGRENVVCMGASGTGK			Carbamidomethyl (C)[10]		Mascot
2819.4714	2819.407	-0.0644	-23	70	94	SIEWTPQNGEYISSNILTLDLLSLL					Mascot

6	Uncharacterized protein OS=Alicyclobacillus acidoterrestris (strain ATCC 49025 / DSM 3922 / CIP 106132 / NCIMB 13137 / GD3B) GN=N007_04755 PE=4 SV=1	tr|T0BUV4|T0BUV4_ALIAG	39782.5	5.77	8	24	0

Peptide Information

Calc. Mass	Obsrv. Mass	± da	± ppm	Start Seq.	End Seq.	Sequence	Ion Score	C. I. %	Modification	Rank	Result Type
999.5258	999.5724	0.0466	47	155	162	YTNLAVYR					Mascot
1028.567	1028.5297	-0.0373	-36	305	313	HMVIGTSRK					Mascot
1193.6195	1193.6489	0.0294	25	190	199	MLTSKELEAR			Oxidation (M)[1]		Mascot
1300.6855	1300.6982	0.0127	10	239	249	GDVRDEVELLR					Mascot
1806.9242	1806.833	-0.0912	-50	35	51	IFDPCCGKGIALQTVGK			Carbamidomethyl (C)[5]		Mascot
1826.853	1826.8771	0.0241	13	92	106	ASMSFLWLNPPYDNR			Oxidation (M)[3]		Mascot
1863.9456	1863.9089	-0.0367	-20	35	51	IFDPCCGKGIALQTVGK			Carbamidomethyl (C)[5,6]		Mascot
2025.0288	2024.9832	-0.0456	-23	69	86	ATEATERLNFVVASPYEK					Mascot
2225.0068	2225.1399	0.1331	60	4	22	YESDSRLGFYATPETMTEK					Mascot

7	Chorismate mutase AroH OS=Alicyclobacillus acidoterrestris (strain ATCC 49025 / DSM 3922 / CIP 106132 / NCIMB 13137 / GD3B) GN=N007_16230 PE=4 SV=1	tr|T0CR07|T0CR07_ALIAG	13505.1	4.88	4	23	0

Peptide Information

Calc. Mass	Obsrv. Mass	± da	± ppm	Start Seq.	End Seq.	Sequence	Ion Score	C. I. %	Modification	Rank	Result Type
1394.8114	1394.693	-0.1184	-85	88	99	SVRVLLHVNTEK					Mascot
2821.4277	2821.405	-0.0227	-8	64	87	SLPGWQWVPLMCARELDVPNSLPR			Carbamidomethyl (C)[12]		Mascot
2829.4768	2829.594	0.1172	41	34	60	LNDIDVDDVASVLLTLTPDLSSAFPAK					Mascot

2875.457	2875.355	-0.102	-35	8	33	GATTVNEDTPEEIFAATK ELIEEIVR					Mascot

8	Uncharacterized protein OS=Alicyclobacillus acidoterrestris (strain ATCC 49025 / DSM 3922 / CIP 106132 / NCIMB 13137 / GD3B) GN=N007_07995 PE=4 SV=1	tr|T0D2A4|T0D2A4 _ALIAG	47100.4	4.92	9	22	0	

Peptide Information

Calc. Mass	Obsrv. Mass	± da	± ppm	Start Seq.	End Seq.	Sequence	Ion Score	C. I. %	Modification	Rank	Result Type
947.4064	947.3455	-0.0609	-64	321	328	EDTNSEPR					Mascot
1029.58	1029.6151	0.0351	34	2	9	QIQSERLR					Mascot
1400.7533	1400.666	-0.0873	-62	138	150	AIFGQLEPGELAR					Mascot
1413.606	1413.6772	0.0712	50	415	427	ASMETGMVSQPML			Oxidation (M)[3,7]		Mascot
1413.606	1413.6772	0.0712	50	415	427	ASMETGMVSQPML			Oxidation (M)[3,7]		Mascot
1537.7172	1537.7108	-0.0064	-4	414	427	RASMETGMVSQPML					Mascot
1553.712	1553.697	-0.015	-10	414	427	RASMETGMVSQPML			Oxidation (M)[4]		Mascot
1569.707	1569.6876	-0.0194	-12	414	427	RASMETGMVSQPML			Oxidation (M)[4,8]		Mascot
1585.7019	1585.71	0.0081	5	414	427	RASMETGMVSQPML			Oxidation (M)[4,8,13]		Mascot
1876.9004	1876.8831	-0.0173	-9	48	64	EAGMTVRIDDCGNLIGR			Carbamidomethyl (C)[11]		Mascot
1892.8954	1892.863	-0.0324	-17	48	64	EAGMTVRIDDCGNLIGR			Carbamidomethyl (C)[11], Oxidation (M)[4]		Mascot
1897.8385	1897.8943	0.0558	29	321	335	EDTNSEPRYCAEWIK			Carbamidomethyl (C)[10]		Mascot
2224.9243	2225.1399	0.2156	97	69	88	CDGPMLMMGSHLDSQP YGGR			Carbamidomethyl (C)[1], Oxidation (M)[5]		Mascot
2875.4771	2875.355	-0.1221	-42	11	36	HFVQLAEYGKIGETGVCR PTLSNVEK					Mascot

9	12-oxophytodienoate reductase OS=Alicyclobacillus acidoterrestris (strain ATCC 49025 / DSM 3922 / CIP 106132 / NCIMB 13137 / GD3B) GN=N007_14435 PE=4 SV=1	tr|T0BFD1|T0BFD 1_ALIAG	38271.4	5.89	7	22	0	

Peptide Information

Calc. Mass	Obsrv. Mass	± da	± ppm	Start Seq.	End Seq.	Sequence	Ion Score	C. I. %	Modification	Rank	Result Type
1213.6899	1213.6543	-0.0356	-29	295	304	IDGLIERLER					Mascot
1395.705	1395.6681	-0.0369	-26	179	190	TRFATEVIEACR					Mascot
1826.9423	1826.8771	-0.0652	-36	331	346	TDELVPFTSEAIKTLY					Mascot
1924.9554	1924.802	-0.1534	-80	16	32	FGNTMLSNRVVMAPMTR					Mascot
2254.0815	2254.2178	0.1363	60	244	262	FWEPEFEGSDLNLAGWT KK					Mascot
2812.4802	2812.6011	0.1209	43	262	288	KLTGKPTITVGSVGLDGE FMSLFTEGK					Mascot
2836.4111	2836.3979	-0.0132	-5	114	140	GNVNDYAESEIVAIVEAF AQAAAEAKR					Mascot

10	Branched-chain amino acid aminotransferase OS=Alicyclobacillus acidoterrestris (strain ATCC 49025 / DSM 3922 / CIP 106132 / NCIMB 13137 / GD3B) GN=N007_09700 PE=3 SV=1	tr|T0BM59|T0BM59_ALIAG	32935	5.18	7	22	0

Peptide Information

Calc. Mass	Obsrv. Mass	± da	± ppm	Start Seq.	End Seq.	Sequence	Ion Score	C. I. %	Modification	Rank	Result Type
978.5367	978.4514	-0.0853	-87	82	90	NALTGAYIR					Mascot
999.5833	999.5724	-0.0109	-11	143	151	TDALNPKIK					Mascot
1028.5483	1028.5297	-0.0186	-18	141	149	NRTDALNPK					Mascot
1660.8806	1660.7361	-0.1445	-87	38	51	VYDGNVFKLAEHIR					Mascot
1870.9164	1870.875	-0.0414	-22	274	289	ALHQAFQQCTRHVGMK			Oxidation (M)[15]		Mascot
1870.9164	1870.875	-0.0414	-22	274	289	ALHQAFQQCTRHVGMK			Oxidation (M)[15]		Mascot
1888.0361	1887.9424	-0.0937	-50	91	108	LVVTRGPGDLGISPYICK					Mascot
1907.0095	1906.8436	-0.1659	-87	109	125	GAQVFIIAEQLSMFPQK					Mascot

Gel Idx/Pos	244/J20	Instr./Gel Origin	BA2151/full sequence test 20150515	Process Status	Analysis Succeeded
Plate [#] Name	[3] full sequence test 20150515	Instrument Sample Name		Spectra	11

Rank	Protein Name	Species	Accession No.	Protein MW	Protein PI	Pep. Count	Protein Score	Protein Score C. I. %	Total Ion Score	Total Ion C. I. %
1	Uncharacterized protein OS=Alicyclobacillus acidoterrestris (strain ATCC 49025 / DSM 3922 / CIP 106132 / NCIMB 13137 / GD3B) GN=N007_08970 PE=4 SV=1		tr|T0BYX4|T0BYX4_ALIAG	114313.8	4.01	7	117	100	117	100

Peptide Information

Calc. Mass	Obsrv. Mass	± da	± ppm	Start Seq.	End Seq.	Sequence	Ion Score	C. I. %	Modification	Rank	Result Type
807.4359	807.4374	0.0015	2	121	127	FNTQVAK					Mascot
935.5309	935.5378	0.0069	7	120	127	KFNTQVAK					Mascot
1117.5848	1117.6078	0.023	21	243	253	DANGNVLSVTK					Mascot
1125.6627	1125.653	-0.0097	-9	35	45	ASQLPIVVNGK					Mascot
1238.6085	1238.6187	0.0102	8	46	56	VLSNPYEMTGK					Mascot
1254.6035	1254.621	0.0175	14	46	56	VLSNPYEMTGK			Oxidation (M)[8]		Mascot
1403.7278	1403.6982	-0.0296	-21	121	134	FNTQVAKDPAGGAK					Mascot
2254.0815	2254.0923	0.0108	5	57	76	DSGNTTGFFPIYYFNQALAK					Mascot
2254.0815	2254.0923	0.0108	5	57	76	DSGNTTGFFPIYYFNQALAK	117	100			Mascot

Rank	Protein Name	Species	Accession No.	Protein MW	Protein PI	Pep. Count	Protein Score	Protein Score C. I. %	Total Ion Score	Total Ion C. I. %
2	Aldehyde dehydrogenase OS=Alicyclobacillus acidoterrestris (strain ATCC 49025 / DSM 3922 / CIP 106132 / NCIMB 13137 / GD3B) GN=gabD PE=3 SV=1		tr|T0DTL9|T0DTL9_ALIAG	52934.1	5.74	12	42	72.267		

Peptide Information

Calc. Mass	Obsrv. Mass	± da	± ppm	Start Seq.	End Seq.	Sequence	Ion Score	C. I. %	Modification	Rank	Result Type
1117.5637	1117.6078	0.0441	39	213	221	EFLENPNVR					Mascot
1144.6508	1144.7327	0.0819	72	231	241	VGKLLMEGAAR					Mascot
1204.5555	1204.6322	0.0767	64	465	474	YAMDAFLETK			Oxidation (M)[3]		Mascot
1838.9033	1838.9519	0.0486	26	465	480	YAMDAFLETKFVSFGI					Mascot
2149.0383	2149.0007	-0.0376	-17	7	23	MFVNHEWIESESKEVLR			Oxidation (M)[1]		Mascot
2181.1445	2181.0266	-0.1179	-54	79	97	NHRDELATIIATEMGKPLR			Oxidation (M)[14]		Mascot
2266.2166	2266.1223	-0.0943	-42	139	159	QPVGVVAAITPWNFPVNMVAR					Mascot
2284.0298	2284.1628	0.133	58	404	423	MANDSTYGLAAYVYTRDNSR			Oxidation (M)[1]		Mascot
2360.1729	2360.1802	0.0073	3	24	46	VISPANGEEVGVVTFGDERDAAK					Mascot

2899.3899	2899.4663	0.0764	26	278	302	TSGQMCICTNRLFVHESVLQEFTQK					Mascot
2928.541	2928.4568	-0.0842	-29	134	159	LMVMRQPVGVVAAITPWNFPVNMVAR			Oxidation (M)[2,4]		Mascot
3260.5503	3260.594	0.0437	13	424	454	CFRMAEGLEYGIVGVNDGAPTQTQAPFGGFK					Mascot

3	ATP-dependent helicase/deoxyribonuclease subunit B OS=Alicyclobacillus acidoterrestris (strain ATCC 49025 / DSM 3922 / CIP 106132 / NCIMB 13137 / GD3B) GN=N007_15460 PE=4 SV=1	tr|T0CXP1|T0CXP1_ALIAG	134118.2	5.29	18	32	0

Peptide Information

Calc. Mass	Obsrv. Mass	± da	± ppm	Start Seq.	End Seq.	Sequence	Ion Score	C. I. %	Modification	Rank	Result Type
807.4723	807.4374	-0.0349	-43	691	697	LTISFAR					Mascot
1183.6357	1183.6669	0.0312	26	1092	1101	SELFGDLFKK					Mascot
1218.5607	1218.6188	0.0581	48	713	722	LCDACDIPVR			Carbamidomethyl (C)[2,5]		Mascot
1277.7399	1277.6279	-0.112	-88	169	178	LRDLCLLYIR					Mascot
1327.7191	1327.7316	0.0125	9	171	180	DLCLLYIRYR					Mascot
1403.7676	1403.6982	-0.0694	-49	62	73	CEVITLTRLAER					Mascot
1412.703	1412.72	0.017	12	903	916	GQHADFQGGVLGAR					Mascot
1766.8207	1766.92	0.0993	56	827	840	LESYAACPYQYFVR			Carbamidomethyl (C)[7]		Mascot
1939.9832	1939.9409	-0.0423	-22	638	654	ALPERVQSNGLLQDDER					Mascot
2248.1392	2248.1238	-0.0154	-7	876	894	AEVRFEQLQLADMTQLAER					Mascot
2254.2668	2254.0923	-0.1745	-77	1007	1025	MFYGLQLQLIVYLAVVQQK					Mascot
2254.2668	2254.0923	-0.1745	-77	1007	1025	MFYGLQLQLIVYLAVVQQK					Mascot
2270.0393	2270.074	0.0347	15	347	366	VWMAEDDEAEAHAVADEILR					Mascot
2291.0762	2291.1665	0.0903	39	1065	1085	KAYVPEGYFNANGTAISAMDR			Oxidation (M)[19]		Mascot
2292.1707	2292.0745	-0.0962	-42	1162	1181	VCHFEPLDHGASYHVLLAKR					Mascot
2317.22	2317.1582	-0.0618	-27	398	418	RYDIPHSLDAFPPLAAHALGR					Mascot
2317.22	2317.1582	-0.0618	-27	398	418	RYDIPHSLDAFPPLAAHALGR					Mascot
2350.1973	2350.2185	0.0212	9	854	874	VAADVGNLLHDTVFQLVDMHR					Mascot
2821.2886	2821.4543	0.1657	59	1066	1091	AYVPEGYFNANGTAISAMDRNFPDGK			Oxidation (M)[18]		Mascot
2899.376	2899.4663	0.0903	31	698	722	THGAAEQRPSPYVMRLCDACDIPVR			Carbamidomethyl (C)[17,20]		Mascot

4	Uncharacterized protein OS=Alicyclobacillus acidoterrestris (strain ATCC 49025 / DSM 3922 / CIP 106132 / NCIMB 13137 / GD3B) GN=N007_12105 PE=4 SV=1	tr|T0BSL1|T0BSL1_ALIAG	40660.2	5.65	9	32	0

Peptide Information

Calc. Mass	Obsrv. Mass	± da	± ppm	Start Seq.	End Seq.	Sequence	Ion Score	C. I. %	Modification	Rank	Result Type
807.3995	807.4374	0.0379	47	142	147	LDFEQR					Mascot
1125.5898	1125.653	0.0632	56	280	288	HDITEIIER					Mascot
1252.7261	1252.6338	-0.0923	-74	2	14	LPVGLDLGNGAVK					Mascot
1252.7261	1252.6338	-0.0923	-74	2	14	LPVGLDLGNGAVK					Mascot
1306.6572	1306.7213	0.0641	49	253	264	NAFPGGRMELAK			Oxidation (M)[8]		Mascot
2314.1763	2314.126	-0.0503	-22	148	168	LSGEHTVRFMSPSPWGG ITVR					Mascot
2318.2	2318.1846	-0.0154	-7	280	299	HDITEIIERHLSAAASHVY R					Mascot
2335.2114	2335.208	-0.0034	-1	350	371	WLNADGMFLTAVRLAAD TVTAV					Mascot
2335.2114	2335.208	-0.0034	-1	350	371	WLNADGMFLTAVRLAAD TVTAV					Mascot
2392.1965	2392.1858	-0.0107	-4	63	84	VFVGKLAADQGPQATYM QPNEK					Mascot
2821.4578	2821.4543	-0.0035	-1	220	246	GNINASLSDGLQYGIGTT LTSLLEDIR					Mascot

5	Uncharacterized protein OS=Alicyclobacillus acidoterrestris (strain ATCC 49025 / DSM 3922 / CIP 106132 / NCIMB 13137 / GD3B) GN=N007_17305 PE=4 SV=1	tr|T0BK78|T0BK78 _ALIAG	20274.6	5.16	6	30	0

Peptide Information

Calc. Mass	Obsrv. Mass	± da	± ppm	Start Seq.	End Seq.	Sequence	Ion Score	C. I. %	Modification	Rank	Result Type
1322.6951	1322.6172	-0.0779	-59	154	165	GIEEPPSEPVIR					Mascot
1403.6261	1403.6982	0.0721	51	37	48	ETFSNCSDVVFR					Mascot
2266.1094	2266.1223	0.0129	6	130	151	GSVTMCVDGDEAALILSV VGWK			Oxidation (M)[5]		Mascot
2333.2751	2333.219	-0.0561	-24	108	129	AVPAAQVGVVTTYNEVV EFVLK					Mascot
2863.3616	2863.5308	0.1692	59	37	61	ETFSNCSDVVFRHIVCPD AVGQGIR			Carbamidomethyl (C)[6,16]		Mascot
2888.5261	2888.5015	-0.0246	-9	62	86	MLITYVDGLVDTKHLDEA VLQVLMR			Oxidation (M)[1]		Mascot

6	Uncharacterized protein OS=Alicyclobacillus acidoterrestris (strain ATCC 49025 / DSM 3922 / CIP 106132 / NCIMB 13137 / GD3B) GN=N007_17145 PE=4 SV=1	tr|T0BBJ0|T0BBJ0 _ALIAG	10294.4	6.58	5	29	0

Peptide Information

Calc. Mass	Obsrv. Mass	± da	± ppm	Start Seq.	End Seq.	Sequence	Ion Score	C. I. %	Modification	Rank	Result Type
1125.6085	1125.653	0.0445	40	7	16	MPVQLDAPVR					Mascot

Calc. Mass	Obsrv. Mass	± da	± ppm	Start Seq.	End Seq.	Sequence	Ion Score	C. I. %	Modification	Rank	Result Type
1186.5409	1186.5804	0.0395	33	62	71	VFESESVMSR			Oxidation (M)[8]		Mascot
1252.6168	1252.6338	0.017	14	19	29	NIYAQNTSDVK					Mascot
1252.6168	1252.6338	0.017	14	19	29	NIYAQNTSDVK					Mascot
2209.1282	2209.0498	-0.0784	-35	72	89	LEEMDEQLLLLQQQIGHR			Oxidation (M)[4]		Mascot
2234.2026	2234.0935	-0.1091	-49	41	61	NGEIDSKTANAIGYLSNIL LK					Mascot

7	ATPase OS=Alicyclobacillus acidoterrestris (strain ATCC 49025 / DSM 3922 / CIP 106132 / NCIMB 13137 / GD3B) GN=N007_03055 PE=4 SV=1	tr|T0DJU4|T0DJU4 _ALIAG	97877.8	5.35	13	25	0

Peptide Information

Calc. Mass	Obsrv. Mass	± da	± ppm	Start Seq.	End Seq.	Sequence	Ion Score	C. I. %	Modification	Rank	Result Type
1117.5485	1117.6078	0.0593	53	181	190	IDVESNLGDR					Mascot
1268.6594	1268.6342	-0.0252	-20	35	46	VHGLNQLSDGTK					Mascot
1327.6965	1327.7316	0.0351	26	461	472	GAPDVLLDRSER					Mascot
1429.7281	1429.7542	0.0261	18	585	597	VLTGAELNNIDDR					Mascot
1429.7281	1429.7542	0.0261	18	585	597	VLTGAELNNIDDR					Mascot
1691.8269	1691.8392	0.0123	7	646	662	QADIGISMGQTGTDVAK					Mascot
2235.1616	2235.1423	-0.0193	-9	132	152	ELVPGDIVLLEDGDRVPA DGR					Mascot
2293.0798	2293.0598	-0.02	-9	379	400	SLIEIAGTCNNAAMVEVD VDGR			Oxidation (M)[14]		Mascot
2347.1099	2347.1843	0.0744	32	521	541	APDWESELVFVGLCGMI DPPR			Oxidation (M)[16]		Mascot
2350.1013	2350.2185	0.1172	50	379	400	SLIEIAGTCNNAAMVEVD VDGR			Carbamidomethyl (C)[9], Oxidation (M)[14]		Mascot
2360.1812	2360.1802	-0.001	0	799	819	AQTMAYATLTMAQLILVF DCR					Mascot
2379.2085	2379.1846	-0.0239	-10	623	645	ALQAREHVVAMTGDGVN DAPAIK			Oxidation (M)[11]		Mascot
2392.1709	2392.1858	0.0149	6	799	819	AQTMAYATLTMAQLILVF DCR			Oxidation (M)[4,11]		Mascot
2462.2014	2462.2166	0.0152	6	379	401	SLIEIAGTCNNAAMVEVD VDGRK			Carbamidomethyl (C)[9]		Mascot
2544.2375	2544.4099	0.1724	68	520	541	RAPDWESELVFVGLCGM IDPPR			Carbamidomethyl (C)[15]		Mascot
2899.4766	2899.4663	-0.0103	-4	374	400	RPALKSLIEIAGTCNNAA MVEVDVDGR			Carbamidomethyl (C)[14]		Mascot

8	Uncharacterized protein OS=Alicyclobacillus acidoterrestris (strain ATCC 49025 / DSM 3922 / CIP 106132 / NCIMB 13137 / GD3B) GN=N007_17850 PE=4 SV=1	tr|T0B9I1|T0B9I1_ ALIAG	15403.8	5.55	5	25	0

Peptide Information

Calc. Mass	Obsrv. Mass	± da	± ppm	Start Seq.	End Seq.	Sequence	Ion Score	C. I. %	Modification	Rank	Result Type
1147.5967	1147.6288	0.0321	28	114	124	GFGINDAARAR					Mascot

Calc. Mass	Obsrv. Mass	± da	± ppm	Start Seq.	End Seq.	Sequence	Ion Score	C. I. %	Modification	Rank	Result Type
1412.7546	1412.72	-0.0346	-24	13	24	YVVRQAHAHFGK					Mascot
1691.8712	1691.8392	-0.032	-19	2	16	ALDVTDADQARYVVR					Mascot
1838.9066	1838.9519	0.0453	25	1	16	MALDVTDADQARYVVR			Oxidation (M)[1]		Mascot
2821.4199	2821.4543	0.0344	12	72	98	QQGHLLGVSSGMGIVAMPLIGFYCASK			Carbamidomethyl (C)[24]		Mascot

9	Uncharacterized protein OS=Alicyclobacillus acidoterrestris (strain ATCC 49025 / DSM 3922 / CIP 106132 / NCIMB 13137 / GD3B) GN=N007_04235 PE=4 SV=1	tr|T0C9D1|T0C9D1_ALIAG	10637.5	6.28	4	25	0

Peptide Information

Calc. Mass	Obsrv. Mass	± da	± ppm	Start Seq.	End Seq.	Sequence	Ion Score	C. I. %	Modification	Rank	Result Type
1870.9592	1870.8739	-0.0853	-46	1	15	MPQTDFHLLSREQLR					Mascot
2209.0476	2209.0498	0.0022	1	45	63	QMNLLEGQMTSEELAEVVR			Oxidation (M)[2,9]		Mascot
2284.1252	2284.1628	0.0376	16	24	43	TDAEIAQMFGISTNTVHHRR					Mascot
2333.1587	2333.219	0.0603	26	44	63	RQMNLLEGQMTSEELAEVVR					Mascot

10	Pyruvate, phosphate dikinase OS=Alicyclobacillus acidoterrestris (strain ATCC 49025 / DSM 3922 / CIP 106132 / NCIMB 13137 / GD3B) GN=N007_15335 PE=3 SV=1	tr|T0CS45|T0CS45_ALIAG	100050.9	5.14	13	25	0

Peptide Information

Calc. Mass	Obsrv. Mass	± da	± ppm	Start Seq.	End Seq.	Sequence	Ion Score	C. I. %	Modification	Rank	Result Type
935.4614	935.5378	0.0764	82	23	31	GASLAEMTR					Mascot
1218.6113	1218.6188	0.0075	6	520	529	TLLQWADESR					Mascot
1403.7212	1403.6982	-0.023	-16	678	689	ALHELNPMLGHR			Oxidation (M)[8]		Mascot
1412.8108	1412.72	-0.0908	-64	348	360	IAVDLVNEGLITR					Mascot
1463.7166	1463.7332	0.0166	11	404	416	VVFDADDAAQWVK					Mascot
2181.1147	2181.0266	-0.0881	-40	426	446	TETTPEDIHGIQAAAGILTSR					Mascot
2207.9666	2208.085	0.1184	54	459	478	GMGKPCVCGCDALTIDPSNR			Carbamidomethyl (C)[6,8,10]		Mascot
2210.239	2210.082	-0.157	-71	656	675	VQRLNAELAELAEVETLLAK					Mascot
2237.123	2237.0991	-0.0239	-11	52	71	EHGGALLPDMVDEIAQEIRK			Oxidation (M)[10]		Mascot
2357.2646	2357.1831	-0.0815	-35	23	45	GASLAEMTRAGLPVPPGFVITTR			Oxidation (M)[7]		Mascot
2692.4202	2692.2927	-0.1275	-47	591	614	LLPMQQGDFYGILKAMDGLPVTIR			Oxidation (M)[4]		Mascot
2863.3899	2863.5308	0.1409	49	559	584	TEHMFMSADRVPVVQSMILAGSTAER					Mascot
2879.3848	2879.5305	0.1457	51	559	584	TEHMFMSADRVPVVQSM			Oxidation (M)[4]		Mascot

2888.4568	2888.5015	0.0447	15	93	121	ILAGSTAER<br>SGAPISMPGMMDTVLNL<br>GLNDSTVVGLAK	Mascot
